# Uncoupling of the allosteric signalling network in the β_1_-adrenergic receptor by a negative allosteric modulator

**DOI:** 10.1038/s41467-026-77802-8

**Published:** 2026-09-18

**Authors:** Thomas H. Harman, Timothy Noel, Ali Nokhbehzaim, Abigail Pearce, Andrew J. Y. Jones, Anjana Saji, Matthew Harris, Venkat S. Vege, Aneesh Chandran, Pierre Leroy, Suleiman Al-Sabah, Graham Ladds, Daniel Nietlispach

**Affiliations:** 1 Department of Biochemistry, University of Cambridge, Cambridge, CB2 1GA UK; 2 Department of Pharmacology, University of Cambridge, Cambridge, CB2 1PD UK; 3 Department of Biotechnology & Microbiology, Kannur University, Kannur, 670 661 Kerala India; 4 Department of Pharmacology & Toxicology, Faculty of Medicine, Kuwait University, Kuwait City, 13060 Kuwait

**Keywords:** Solution-state NMR, G protein-coupled receptors, Computational biophysics

## Abstract

Allosteric modulation is a fundamental aspect of G protein-coupled receptor (GPCR) regulation where molecules that bind to alternative sites outside the orthosteric pocket alter agonist-induced signalling responses. The advantages of allosteric modulators over traditional orthosteric agonists have positioned them as highly promising therapeutic agents. GPCRs exist in tuneable equilibria between multiple functional states, yet the ways in which allosteric modulators reshape these equilibria remain incompletely understood. Here we characterise how changes to the allosteric signalling network influence the conformational dynamics of the β_1_-adrenergic receptor (β_1_AR) through mutations at the E130^3.41^ position or using the negative allosteric modulator, AS408. Both act through a known transmembrane (TM)3-TM4-TM5 site, reducing signalling to similar extents, but through distinct molecular mechanisms as revealed by pharmacological assays, MD simulations, and solution nuclear magnetic resonance spectroscopy. E130^3.41^ mutations reduce agonist-stimulated signalling as a side chain-dependent rheostat, shifting receptor equilibria to populate states with weaker G protein affinities. In contrast, AS408 uncouples the intracellular region of β_1_AR from the influence of the orthosteric pocket and reduces G protein coupling by adoption of an alternative signalling-incompetent intracellular surface arrangement. Our investigations emphasise the relevance of mechanistic understanding in drug development and the importance of dynamics in GPCR allostery.

## Abstract (web summary)

Understanding allosteric modulation mechanisms at G protein-coupled receptors (GPCRs) is key to development of therapeutics. Here, the authors show how a negative allosteric modulator acts to uncouple the signalling network of β1-adrenergic receptor.

## Introduction

G protein-coupled receptors (GPCRs) are seven-transmembrane (TM) proteins that, following ligand binding, relay extracellular stimuli across the cell surface membrane to the cytosol to facilitate cellular responses^1^. Communication of the ligand-specific instructions across the membrane during receptor activation is mediated by highly interconnected allosteric networks. Conserved microswitches such as the P^5.50^-I^3.40^-F^6.44^ or N^7.49^-P^7.50^-x-x-Y^7.53^ motifs (superscripts refer to Ballesteros-Weinstein numbering^2^) facilitate residue side chain repacking within the hydrophobic core of class A GPCRs^3–5^. This repacking results in substantial structural rearrangements towards the intracellular surface of the GPCR^3^ and enables the population of receptor states that favour coupling to signalling effectors, which include heterotrimeric G proteins and β-arrestins^6^.

Modulation of GPCR activity is essential to the regulation of downstream signalling pathways in many disease states, and thus over 500 approved therapeutics have been developed to target GPCRs^7,8^. A majority of small molecule GPCR drugs bind into an orthosteric binding pocket, which is generally located towards the extracellular surface^9^. In contrast, allosteric modulators act at distinct sites from canonical orthosteric pockets to increase (positive allosteric modulator, PAM) or decrease (negative allosteric modulator, NAM) signalling at receptors bound to orthosteric ligands. These drugs bind commonly but not exclusively into shallow pockets formed between the transmembrane helices^10,11^. Although allosteric modulators are associated with several benefits over orthosteric ligands, including generally increased selectivity since allosteric pockets have lower sequence conservation than orthosteric sites^12^, there are far fewer allosteric modulators available in the clinic. Drugs with these modalities only represent a small (~1%^13^) proportion of approved drugs. Consequently, allosteric modulators remain a substantial area of interest for GPCR drug development.

**Fig. 1:**
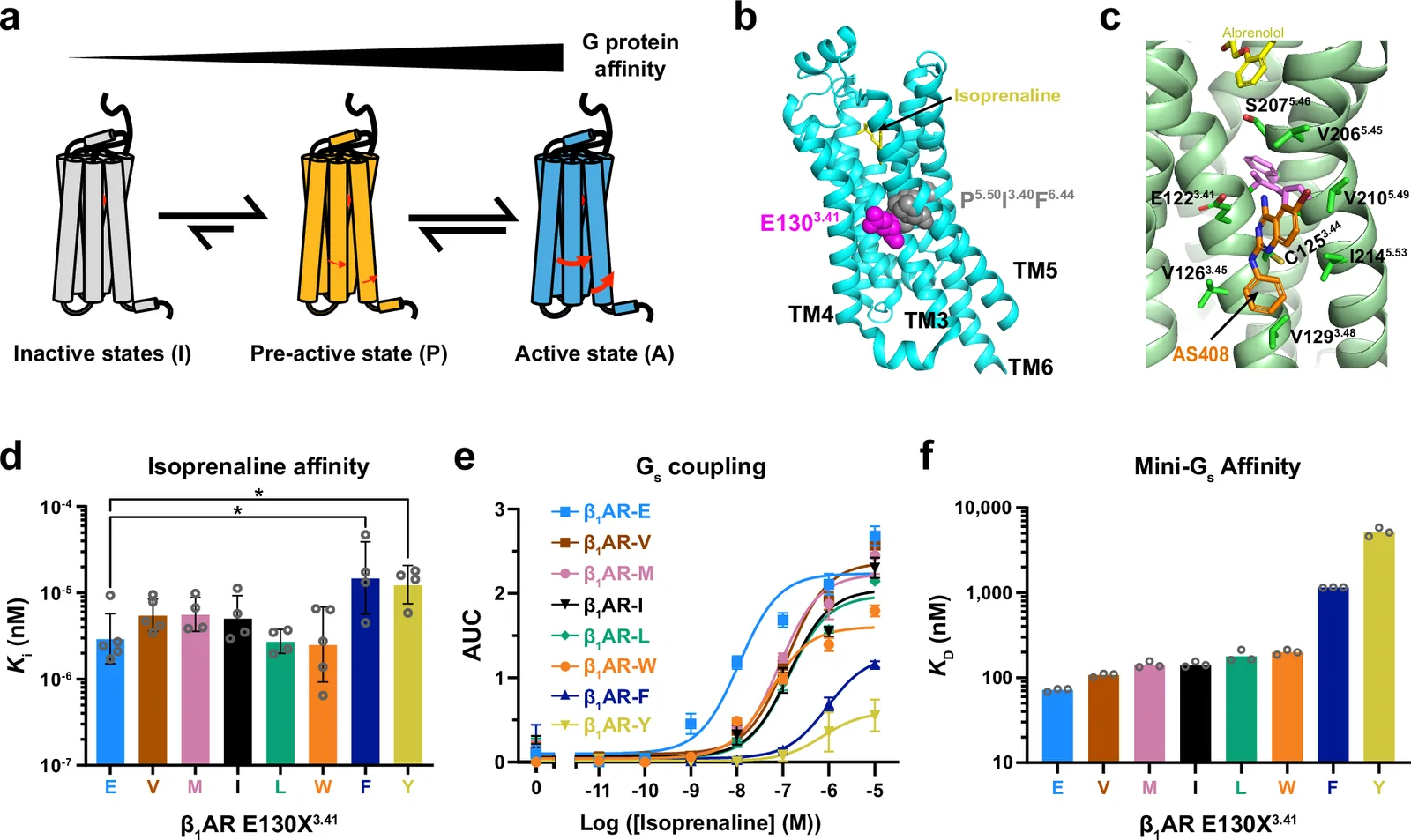
E130X^3.41^ mutations reduce β_1_AR signalling by decreasing affinity for G proteins in a rheostat fashion. **a** Cartoon illustration of β_1_AR activation showing conformational equilibria between inactive (I), pre-active (P) and active (A) states and their relative G protein affinities. The E130X^3.41^ mutations lower the activity of the agonist-stimulated receptor by shifting the equilibria to the left, favouring states with reduced G protein affinity. The equilibria are supported by previous NMR studies^14–25^. **b** The β_1_AR structure (PDB ID: 2Y03)^87^ showing the location of E130^3.41^ in the TM3-TM4-TM5 allosteric pocket. The E130^3.41^ position (purple), orthosteric agonist (isoprenaline, tan), PIF motif (grey) and transmembrane helices are labelled. **c** The AS408-β_2_AR structure (PDB ID: 6OBA)^29^ showing the binding of AS408 into the TM3-TM4-TM5 allosteric pocket. The positions of proximal side chains, including E122^3.41^, are labelled. The PIF motif side chains are shown in purple. AS408 is shown in orange, and the orthosteric ligand (alprenolol) is shown in yellow. **d** Isoprenaline affinities (equilibrium *K*_i_ values) to β_1_AR E130X^3.41^ variants expressed in HEK293T cells, determined in cell using nanoBRET signal loss through titration of isoprenaline against (S)-propranolol-red. Data shown are mean ± SEM of *n* = 4 (M,I,L,F,Y) or *n* = 5 (E,V,W) independent biological experiments, each performed in duplicate. Significance is indicated with *, analysed on log-transformed data by one-way ANOVA with Dunnet’s post-hoc tests (β_1_AR-E vs β_1_AR-F, *p* = 0.0195; β_1_AR-E vs β_1_AR-Y, *p* = 0.0074). **e** TRUPATH BRET2 biosensor G protein dissociation assay in HEK293T cells measuring activation of G_s_ by β_1_AR E130X^3.41^ variants. Responses were measured for varying isoprenaline concentrations and data fitted using the 3 parameter logistic equations. Data are mean ± SEM of *n* = 3 (V, M, I, L, F, Y) or *n* = 5 (E, W) independent biological experiments, each performed in duplicate. **f** Affinities (*K*_D_) for β_1_AR E130X^3.41^ ternary complex formation with mini-G_s_ in the presence of a saturating isoprenaline concentration (500 µM), measured by BLI assays. Means were calculated from dose-response experiments with *n* = 3 independent technical replicates.

Structural studies indicate GPCRs sample several distinct functional states connected via tuneable equilibria, including inactive, pre-active, and active states^14–25^ (Fig. 1a). The exchange between receptor states is directly mediated by the conserved microswitches in the allosteric activation network. This conformational plasticity is critical to receptor signalling as binding of orthosteric ligands modify GPCR equilibrium populations and the rates at which these receptor states interconvert^14,16,18,19,24,26–28^. Changes in the magnitude of functional responses subsequently arise as these states couple to binding partners with variable effectiveness. Some allosteric modulators have been suggested to stabilise specific receptor states within these equilibria, thereby altering the efficiency of binding partner recruitment induced by an orthosteric ligand^5,11,29–34^. Current GPCR structures with allosteric modulators represent around 2% of structures in the GPCRdb^35^, with fewer studies conducted with solution methods such as nuclear magnetic resonance (NMR) spectroscopy^30–34,36,37^. Thus, although communication between allosteric sites and the internal allosteric network of microswitches is integral to the control of receptor activity, the molecular mechanisms by which allosteric modulators impact signalling networks and energy landscapes of GPCRs^4,11,38–40^ are less well understood than for orthosteric ligands and warrant further investigation.

The β_1_-adrenergic receptor is the most prevalent βAR subtype in cardiac tissue^41^ and has been targeted with a range of orthosteric agonists^42^. The conformational equilibria of β_1_AR have been investigated through pharmacological assays and solution studies including NMR spectroscopy^14–16,22,26–28,36,43,44^, providing a useful model system to investigate modulation of receptor energy landscapes. Recently, we showed that reversal of a thermostabilising E130W^3.41^ mutation^45^ to wildtype E130^3.41^ shifted the equilibria of the turkey β_1_-adrenergic receptor (hereon referred to as β_1_AR) towards an active state^14^. The E^3.41^ position is located in a known TM3-TM4-TM5 allosteric site in GPCRs^30,31,46–52^. In our current investigation, we establish how varying the environment in this allosteric pocket through introduction of different mutations at this key position can influence the dynamics of the β_1_AR, and how this informs on the regulation of the allosteric signalling network through this site.

We then endeavour to compare these data to the effects elicited by a small molecule allosteric modulator. A recent study introduced AS408 as a NAM for the human β_2_AR (hβ_2_AR)^29^. AS408 was shown to bind hβ_2_AR at the TM3-TM4-TM5 interface, with the 3.41 position forming a key part of the allosteric pocket. The same work found that, although the affinity for the NAM was highest at the hβ_2_AR, AS408 reduced β-arrestin recruitment at several other related receptors in addition to hβ_2_AR, including the human β_1_AR (hβ_1_AR). Based on sequence and structural homology, we anticipated that AS408 would act through the same allosteric site at the β_1_AR. Consequently, AS408 could enable pharmacological and structural investigations of small molecule allosteric modulation, complementary to mutations at E130^3.41^, at the β_1_AR.

Here, we first confirm the location of the AS408 binding site at the β_1_AR and establish that AS408 acts as a NAM for G protein signalling. We then investigate the structural mechanisms by which AS408 affects β_1_AR signalling and its internal allosteric network by NMR spectroscopy and pharmacological functional assays, utilising mutations at E130^3.41^ to guide the analysis of conformational equilibria changes induced by AS408.

Our investigations reveal a similar reduction in G protein signalling at the β_1_AR by both AS408 and mutations at the E130^3.41^ position, but by differing mechanisms. Mutations at the E130^3.41^ position alter the magnitude of receptor signalling by stabilising pre-active or inactive states with lower affinities for G proteins than the agonist-stabilised active state, effectively reducing the efficacy of the agonist. In contrast, AS408 acts distinctly from this mechanism by stabilising an alternative non-productive conformation differing from currently identified receptor states. Notably, we find uncoupling of the allosteric network upon AS408 binding, as the intracellular region adopts an alternative arrangement whilst the organisation of the orthosteric pocket remains predominantly determined by the bound agonist. Overall, our study elucidates the roles of conformational dynamics and plasticity in allosteric modulation of GPCRs and offers insight into the differing signalling mechanisms that can emanate from the same allosteric site.

## Results

### E130X^3.41^ mutations reduce activity by shifting β_1_AR equilibria

Activation of the β_1_AR by orthosteric ligands arises from the repositioning of dynamic equilibria connecting receptor states of differing signalling activity (Fig. 1a), due to the communication of ligand information through the allosteric network of conserved microswitches. In our recent work with β_1_AR, we found that reverting the E130W^3.41^ thermostabilising mutation^45^ to E130^3.41^ (β_1_AR-E) (Fig. 1b) resulted in a more active receptor which occupied an active state when stimulated with a full agonist^14^. The 3.41 position (Supplementary Fig. 1) is adjacent to I^3.40^ in the PIF motif and forms part of the pocket in which the allosteric modulator AS408 binds to hβ_2_AR^29^ (Fig. 1b-c, Supplementary Fig. 2). We hypothesised that other mutations at E130^3.41^ would result in changes to the receptor energy landscape that might further our mechanistic insight into how an allosteric modulator binding at this site rewires the allosteric network in β_1_AR. Therefore, we characterised the pharmacological profiles and conformational equilibria for a set of receptor constructs with differing residues at the E130^3.41^ position (β_1_AR-X, where X is the mutation at the E130^3.41^ position).

In the work in which the W^3.41^ thermostabilising mutation was proposed for the hβ_2_AR, the authors examined a range of mutations at the 3.41 position^45^. We chose a subset of these mutations to investigate at the β_1_AR, including a group of hydrophobic residues (E130I/L/M/V^3.41^) and a second set of aromatic residues (E130W/F/Y^3.41^), selected due to the presence of aromatic ring systems in AS408 (Fig. 1c).

Ligand binding affinities for the full agonist isoprenaline to β_1_AR E130X^3.41^ variants were determined in competition assays, using receptor constructs N-terminally tagged with NanoLuc (Nluc) (Supplementary Fig. 3a) and expressed in HEK293T cells^14,53,54^. Low micromolar *K*_i_ binding affinities were observed for all β_1_AR constructs (Fig. 1d, Supplementary Fig. 3-4). Analyses of kinetic parameters revealed no significant differences in ligand dissociation or association rate constants between β_1_AR-E and the β_1_AR E130X^3.41^ variants (Supplementary Fig. 3b-e), and we found no significant difference in p*K*_i_ between β_1_AR-E (p*K*_i_ = 5.44) and hydrophobic E130X^3.41^ variants or β_1_AR-W (*p* > 0.05). β_1_AR-F and β_1_AR-Y did show significant decreases in isoprenaline equilibrium binding affinities compared with β_1_AR-E (p*K*_i_ = 4.69, *p* = 0.0195; and p*K*_i_ = 4.87, *p* = 0.0074, respectively), although still within one order of magnitude.

β_1_AR-E displays primary coupling to the G_s_ protein subtype with secondary coupling to the G_i/o_ family upon agonist stimulation^14,41^. Therefore, we assessed the effect of E130X^3.41^ mutations on isoprenaline-stimulated G protein coupling using the TRUPATH BRET2 biosensor assay^55^ in HEK293T cells (Supplementary Fig. 5a). β_1_AR-E had the greatest potency for G protein activation with respect to G_s_ (Fig. 1e) and representative G_i/o_ family members (Supplementary Fig. 5b–c). Decreases in potency were observed for hydrophobic E130X^3.41^ variants relative to β_1_AR-E, more similar to the previously characterised β_1_AR-W variant^14^, whilst β_1_AR-F and β_1_AR-Y were unable to generate full concentration-response curves.

We determined that cell-surface expression of the E130X^3.41^ variants did not influence these findings, as there was no significant difference (*p* > 0.1) between FLAG-tagged β_1_AR-E and the E130X^3.41^ variants measured by flow cytometry (Supplementary Fig. 6).

We further characterised G_s_ protein coupling through binding affinities of the engineered mini-G_s_ protein^56^ to isoprenaline-stimulated receptors using biolayer interferometry (BLI) dose-response assays^14^ (Supplementary Fig. 7a–b). Receptor affinities for mini-G_s_ protein were reduced for all E130X^3.41^ variants compared to β_1_AR-E (Fig. 1f, Supplementary Fig. 8), mirroring the trends in potency decreases obtained in the TRUPATH assays. Mini-G_s_ affinities for hydrophobic E130L/I/M/V^3.41^ variants were intermediate between β_1_AR-E and β_1_AR-W but were over an order of magnitude (~16-72 fold) lower for β_1_AR-F and β_1_AR-Y relative to β_1_AR-E. These differences in mini-G_s_ affinities resulted from decreases in *k*_on_ rate constants and increases in *k*_off_ rate constants (Supplementary Fig. 7c–d, Supplementary Table 1).

Having established that signalling through β_1_AR was significantly reduced by E130X^3.41^ mutations without substantial effects on full agonist binding, we reasoned that the decreases in potency were related to changes in communication through the allosteric network of microswitches between the ligand binding site and the intracellular G protein binding surface. Therefore, we examined the effects of E130X^3.41^ mutations on receptor conformational equilibria by NMR spectroscopy (Supplementary Fig. 9). We first investigated the impact on the intracellular surface equilibrium, most distant from the orthosteric ligand, using single site ^19^F labelling coupled with ^19^F NMR spectroscopy.

**Fig. 2:**
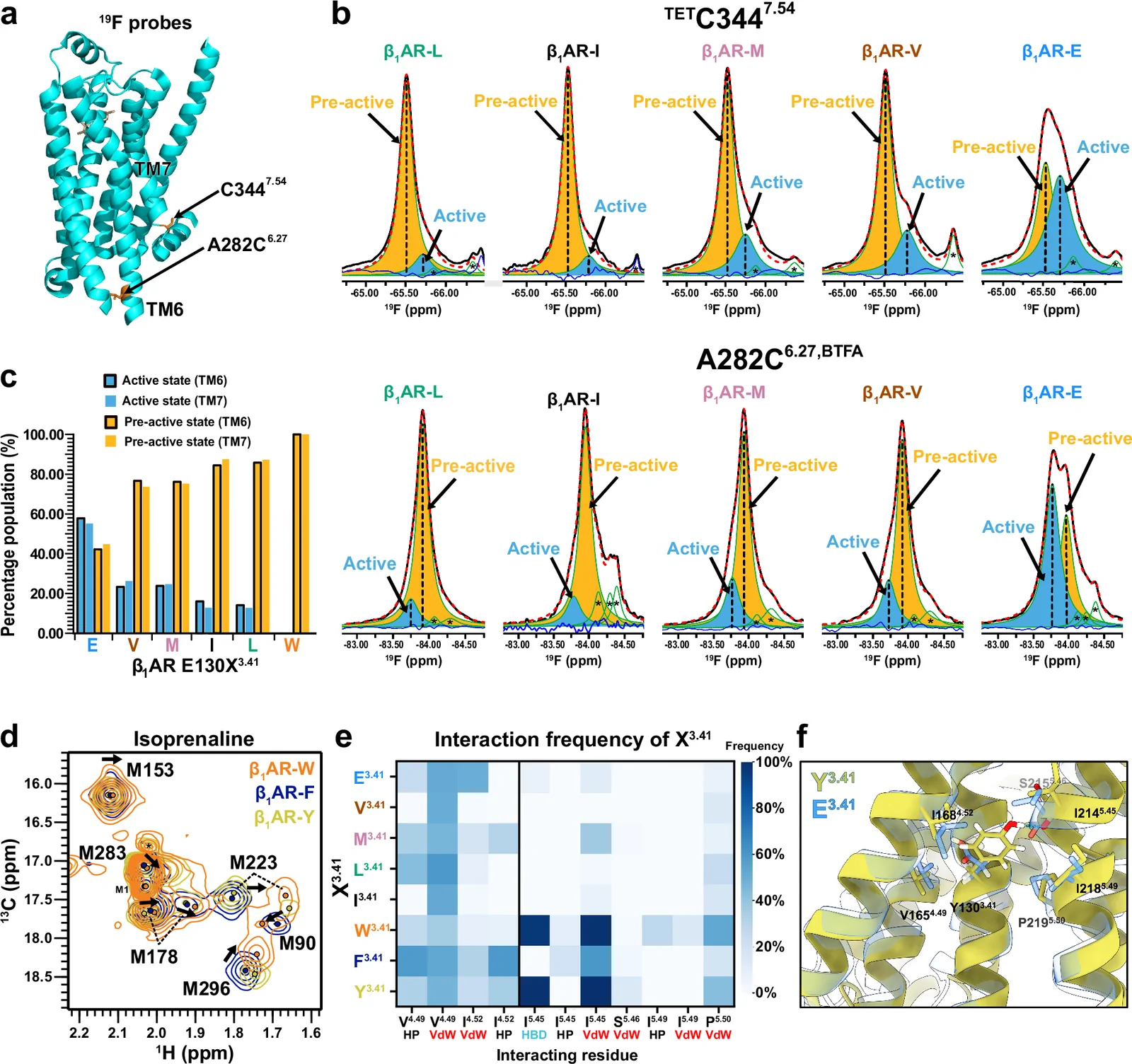
E130X^3.41^ mutations modulate β_1_AR by stabilising pre-existing states with lower affinity for G proteins. **a** The β_1_AR structure showing the location of the TM6 and TM7 ^19^F NMR probes (PDB ID: 2Y03)^87^. **b** 1D ^19^F NMR spectra of β_1_AR ^TET^C344^7.54^ (TM7) (top) and β_1_AR A282C^6.27,BTFA^ (TM6) (bottom) for the hydrophobic E130X^3.41^ variants (left-to-right: β_1_AR-L, β_1_AR-I, β_1_AR-M, β_1_AR-V and β_1_AR-E), recorded in the presence of 1 mM isoprenaline at 298 K. Signal deconvolutions are shown as filled-in areas with signal positions of the active state (blue) and pre-active state state (orange) indicated by black dashed lines. Individual deconvoluted peaks are shown as green lines. Residuals (blue lines) are the subtraction of the sum of the deconvoluted peaks (red dashed lines) from the original spectrum (black lines). * indicates degradation products. **c** Percentage of pre-active state (orange) and active state (blue) populations for β_1_AR E130X^3.41^ variants, determined from the summed AUC of the ^19^F NMR signals in (**b**). The population of the pre-active state for the β_1_AR-W variant was previously determined in Frei et al.^26^. **d** An overlay of 2D ^1^H-^13^C HMQC NMR spectra of β_1_AR aromatic E130X^3.41^ variants in the presence of 1 mM isoprenaline, recorded at 308 K. Peak maxima are indicated by dots. Sizeable chemical shift changes between variants are indicated by black arrows in direction of increasing receptor activity (Y < F < W). * indicates an artifact related to ^1^H 180˚ pulse imperfection. **e** Frequency of interactions formed by the E130X^3.41^ residue in all-atom MD simulations (*n* = *3* replicates). Interaction types are indicated (Van der Waals (VdW), hydrogen bond donor (HBD), hydrophobic (HP)). Only residues with at least one interaction exceeding 10% frequency on average are shown. Immediate neighbours within 4 residues of E130X^3.41^ are excluded. **f** Representative snapshot from MD simulations of β_1_AR-E (blue) and β_1_AR-Y (yellow), comparing the TM3–TM4–TM5 allosteric pocket. The Y130^3.41^ aromatic side chain packs more tightly against TM5 residues than E130^3.41^. The dashed blue line indicates a hydrogen bond between Y130^3.41^ and the backbone carbonyl of I214^5.45^. Only interacting residues (exceeding 10% frequency on average) are shown.

We recorded NMR experiments of detergent-purified β_1_AR-E and E130L/I/M/V^3.41^ variants, labelled with ^19^F probes at the TM7/intracellular loop (IL) 4 cysteine C344^7.54^ (^TET^C344^7.54^)^26,27^ or the introduced TM6 cysteine A282C^6.27^ (A282C^6.27,BTFA^)^26,27^ (Fig. 2a), in the presence of isoprenaline. Spectra were then analysed by signal deconvolution to establish the populations of receptor states^14^. All four hydrophobic E130X^3.41^ variants occupied two states matching the previously described pre-active and active states observed for isoprenaline-bound β_1_AR-E^14^ (Fig. 2b, Supplementary Fig. 10). We measured the populations of receptor states for each variant, as measured by the relative areas under the curve (AUC) for each signal. Unlike β_1_AR-E, which favours occupation of the active state^14^, the hydrophobic E130X^3.41^ variants predominantly occupied the pre-active state as previously observed for isoprenaline-bound β_1_AR-W^26^ (Fig. 2c). A comparison of active state:pre-active state ratios demonstrated that β_1_AR-L (^TET^C344^7.54^, 0.15; A282C^6.27,BTFA^, 0.17) and β_1_AR-I (^TET^C344^7.54^, 0.14; A282C^6.27,BTFA^, 0.19) showed lower occupation of the active state whilst the ratios for β_1_AR-M (^TET^C344^7.54^, 0.33; A282C^6.27,BTFA^, 0.31) and β_1_AR-V (^TET^C344^7.54^, 0.36; A282C^6.27,BTFA^, 0.30) were greater, though still much less than for β_1_AR-E (^TET^C344^7.54^, 1.24; A282C^6.27,BTFA^, 1.39).

We confirmed a corresponding response of the other regions in β_1_AR to the effects of the E130X^3.41^ mutations using 2D ^1^H-^13^C HMQC methionine NMR shift correlation experiments in combination with ^13^C-methyl-methionine selective isotope labelling. The ^13^C NMR spectra substantiated that hydrophobic E130X^3.41^ variants predominantly populated the pre-active state (Supplementary Fig. 11). Changes for multiple probes relative to spectra of β_1_AR-W, which populates the pre-active/inactive state equilibrium^26,27^, also corroborated the minor occupation of the active state (Supplementary Figs. 11–12).

Together, the NMR data indicated that the hydrophobic E130X^3.41^ mutations shifted the relative β_1_AR populations of the active/pre-active state equilibrium towards the lesser active pre-active state, compared to β_1_AR-E (Fig. 1a). The pre-active state has a lower affinity for G proteins than the active state^14,22^, therefore explaining the reduced signalling observed in TRUPATH and BLI assays (Fig. 1e-f).

We hypothesised that aromatic E130X^3.41^ mutations would move the agonist-bound receptor equilibrium position even further away from the active state as these variants had substantially reduced signalling capabilities. ^13^C NMR spectra of isoprenaline-bound β_1_AR-F and β_1_AR-Y were generally similar to β_1_AR-W (Fig. 2d), in agreement with the population of the pre-active/inactive state equilibrium. Previous NMR investigations of β_1_AR revealed correlations between ligand efficacy and chemical shift positions for multiple methionine probes^14,27^. These correlations were indicative of changes in the equilibrium position between the inactive and pre-active receptor states that exchanged rapidly on the NMR timescale. We observed similar chemical shift dependencies for several probes in the spectra of E130X^3.41^ aromatic variants, including for M153^34.57^, M178^4.62^, M283^6.28^, and M296^6.41^, that correlated with receptor signalling activity (Fig. 2d, Supplementary Fig. 13). Accordingly, we concluded that the populations of the pre-active state were smaller for β_1_AR-F and β_1_AR-Y than for β_1_AR-W, decreasing in the order β_1_AR-W > β_1_AR-F > β_1_AR-Y, whilst the inactive state population increased reciprocally. Thus, aromatic E130X^3.41^ mutations reduced G protein coupling by stabilising the inactive state, which results in poor signalling.

Finally, we assessed how the E130X^3.41^ mutations affected receptor dynamics in triplicate 1 µs all-atom molecular dynamics (MD) simulations of isoprenaline bound β_1_AR starting from an activated state^57^. The canonical activation microswitches^4^, such as the TM3-TM6 hydrophobic lock, the DRY motif ionic lock, the PIF motif, and the Y^5.58^-Y^7.53^ motif, generally remained consistent with an active-like state in all systems for the duration of the simulation, although the Y^5.58^-Y^7.53^ motif being most susceptible to disruption (Supplementary Fig. 14). Interactions of the agonist were also maintained and were similar across the variants (Supplementary Fig. 15). These simulations implied that the structural transitions which resulted in the differing conformations observed by NMR occurred on a longer timescale than in our simulations, and hence we focused on the local dynamics of the TM3-TM4-TM5 allosteric pocket. Analysis of molecular interactions revealed that the variants established increased interactions with TM5 residues relative to E130^3.41^. Compared to E130^3.41^, aromatic mutants E130W^3.41^ and E130Y^3.41^ formed considerably more hydrogen bonds (7.2 ± 3.6% (standard deviation) vs 96.5 ± 1.2% and 98.4 ± 0.1%, respectively), and Van der Waals (VdW) interactions (11.5 ± 2.8% vs 98.2 ± 0.9% and 99.6 ± 0.1 %, respectively) with I214^5.45^ (hydrogen bonding with the carbonyl group) and VdW interactions (7.1 ± 0.6% vs 53.0 ± 2.8% and 43.3 ± 5.7%) with P219^5.50^. These VdW interactions were also observed for E130F^3.41^ and hydrophobic mutants but to a lesser degree (Fig. 2e–f, Supplementary Figs. 16, 17).

Overall, E130X^3.41^ mutations reduced signalling efficacy at the full agonist-stimulated β_1_AR by shifting the receptor equilibria populations away from the active state in direction of the less active states, acting through the PIF motif.

### AS408 reduces β_1_AR signalling by binding the TM3-TM4-TM5 site

Our MD simulations of the E130X^3.41^ variants revealed similar interactions in the allosteric pocket, specifically involving I214^5.45^ and P219^5.50^ with the aromatic E130X^3.41^ position in β_1_AR-W and β_1_AR-Y, as those reported with the small molecule allosteric modulator AS408 at the hβ_2_AR^29^. We, therefore, utilised our pharmacological and NMR methodologies to inform on the likely mechanism by which the small molecule AS408 modulates agonist-stimulated signalling at the β_1_AR and to compare it to the E130X^3.41^ variants.

**Fig. 3:**
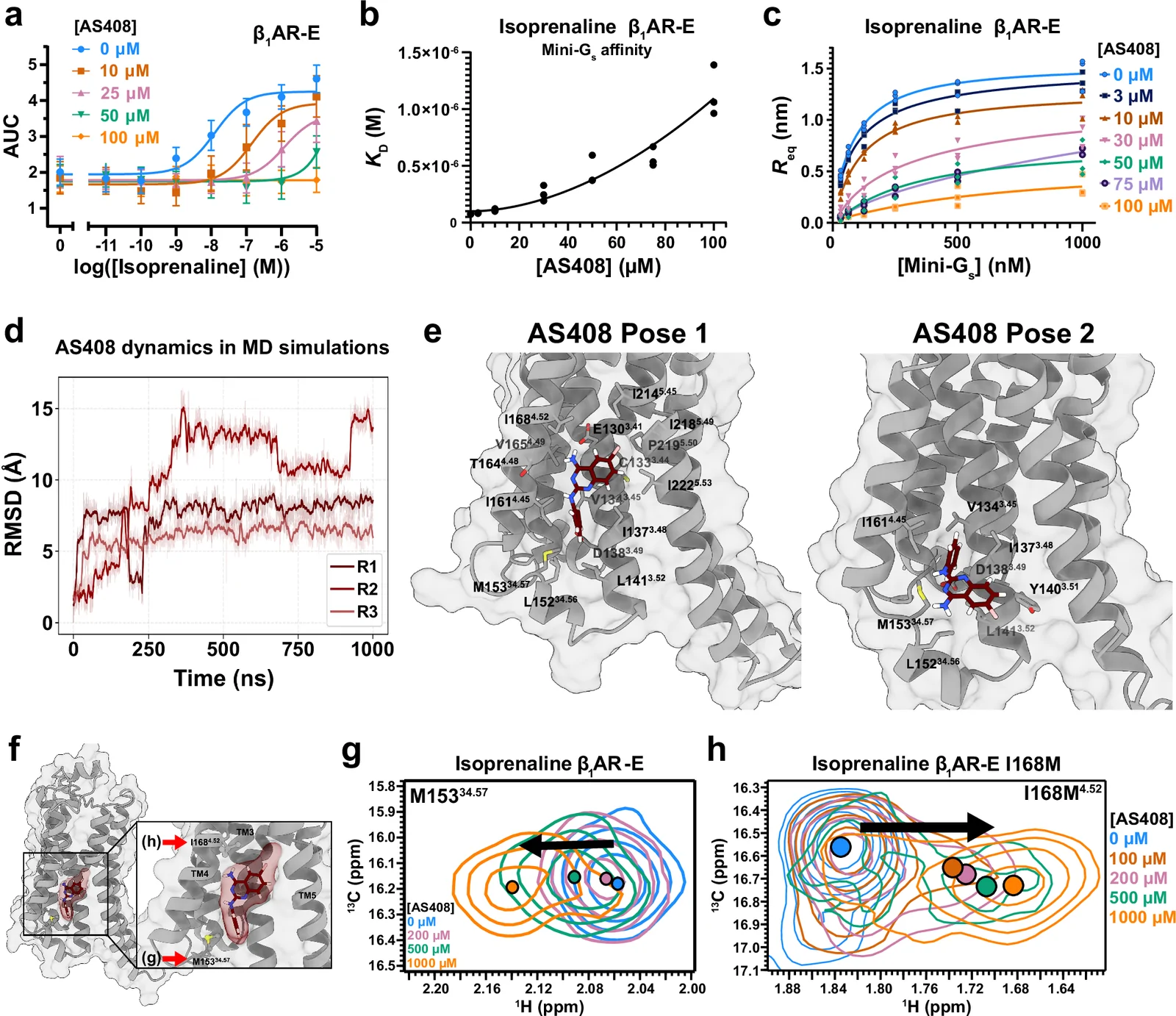
AS408 reduces G protein coupling at the β_1_AR. **a** TRUPATH BRET2 biosensor G protein dissociation assay measuring binding of G_s_ to β_1_AR-E modulated by AS408, in HEK293T cells. Responses for each AS408 concentration were fitted using the 3 parameter logistic equations. Data are mean ± SEM of *n* = 3 independent biological experiments, each performed in duplicate. **b** Affinity (*K*_D_) of mini-G_s_ binding to isoprenaline-bound (500 µM) β_1_AR-E modulated by AS408, measured by biolayer interferometry (BLI). Values for each AS408 concentration were calculated from *n* = 3 dose-response experiments (independent technical replicates), and fit with [Inhibitor] vs Response (three parameter variable slope), with a Hill constant fixed at −1. **c** Modulation by AS408 of *R*_eq_ (the equilibrium binding signal, a measure of bound analyte, obtained using a 1:1 global fitting with *R*_max_ unlinked by sensors) for mini-G_s_ binding to isoprenaline-bound (500 µM) β_1_AR-E, measured by BLI. Data are *n* = 3 independent technical replicates. **d** The root mean square deviation (RMSD) of AS408 atoms relative to the initial AS408 pose over 1 µs MD simulations for *n* = *3* replicates (R1-R3). Traces are rolling means of a 5 ns window, with the unaveraged values shown as faint traces. The sharp increases at the beginning indicate that the starting pose of AS408 in hβ_2_AR^29^ is not stable for the turkey β_1_AR. **e** Representative snapshots for binding poses of AS408 at β_1_AR-E in MD simulations. Binding pose 1 represents the major pose at the interface of TM3-TM4-TM5. Binding pose 2 shows the alternative pose in which AS408 is closer to the intracellular end of TM4 and IL2. Residues within 4 Å of AS408 are shown in stick representation. **f** Positions of NMR probes M153^34.57^ and I168M^4.52^ relative to AS408 in binding pose 1. **g** An overlay of 2D ^1^H-^13^C HMQC NMR spectra for isoprenaline-bound (1 mM) β_1_AR-E L289M^6.34^ (308 K) showing the M153^34.57^ probe region with increasing AS408 concentration. The black arrow indicates the direction of the chemical shift change. **h** An overlay of 2D ^1^H-^13^C HMQC NMR spectra of isoprenaline-bound (1 mM) β_1_AR-E I168M^4.52^ (308 K), showing the I168M^4.52^ probe region, with increasing AS408 concentration.

We established that AS408 reduced signalling through the G protein pathway at the β_1_AR in both TRUPATH (Fig. 3a) and BLI (Fig. 3b, c, Supplementary Fig. 18) assays, reminiscent of the E130X^3.41^ mutations. We applied the operational model of allostery^58^ to the TRUPATH assay data to obtain parameters for affinity cooperativity log *α* (−1.5 ± 0.4) and efficacy cooperativity log *β* (−1.7 ± 0.3) (Supplementary Fig. 19a, Supplementary Table 2). By combining these two parameters log(*αβ*) we can determine the overall effect of an allosteric modulator on agonist signalling. For AS408, the log(*αβ*) is −3.2 ± 0.5, thereby confirming AS408 as a NAM at the β_1_AR. The affinity of AS408 to β_1_AR-E (log(*K*_B_) = −6.1 ± 0.2) was similar to the hβ_1_AR when stimulated with noradrenaline (log(*K*_B_) = −5.6 ± 0.1)^29^. In the BLI assays, AS408 decreased mini-G_s_ binding through concentration-dependent reductions in the affinity for β_1_AR-E (Fig. 3b–c). These changes were primarily the result of reductions in the mini-G_s_ *k*_on_ rate constant, which decreased by an order of magnitude over the AS408 concentration range (Supplementary Fig. 18a, b). In contrast to the E130X^3.41^ variants, the *k*_off_ rate constant remained similar with increasing AS408 concentration (Supplementary Fig. 18c), indicating that AS408 had little effect on β_1_AR-E in ternary complex with mini-G_s_. We further observed AS408-dependent decreases in mini-G_s_ binding to the apo receptor (Supplementary Fig. 20a, b), consistent with inverse agonist behaviour noted for AS408 at hβ_2_AR^29^, whereas there was no observable impact of AS408 addition on the negligible coupling between mini-G_s_ and β_1_AR-E bound to the antagonist propranolol^42^ (Supplementary Fig. 20c–e).

We next assessed the structural properties of AS408 binding to β_1_AR-E in MD all-atom simulations. The allosteric pocket is highly similar between the hβ_2_AR and β_1_AR, implying AS408 would likely bind at the same interface at β_1_AR-E. Therefore, we ran 1 µs simulations in triplicate starting from the activated β_1_AR-E structure with AS408 modelled into the TM3-TM4-TM5 allosteric pocket in the same orientation as in the hβ_2_AR crystal structure^29^. The addition of AS408 did not lead to inactivation of the receptor on the 1 µs simulation timescale as indicated by the canonical activation microswitches^4^, similar to the E130X^3.41^ variant simulations (Supplementary Fig. 14). Therefore, we focused our analyses on the AS408 binding pocket.

Differences in AS408 binding were conceivable compared to β_2_AR, since the valine residues at positions 3.48, 5.45 and 5.49 in hβ_2_AR are larger isoleucine residues in β_1_AR (Supplementary Fig. 21a). Indeed, the RMSD of AS408 suggested that the initial binding pose of AS408 proved unstable (Fig. 3d), meaning that the allosteric binding pocket of β_1_AR did not accommodate AS408 in the same orientation as hβ_2_AR. The polar interaction of AS408 with the backbone of residue 5.45, which is prevalent in hβ_2_AR, was significantly reduced in AS408 binding to the β_1_AR (99.0% vs 2.6 ± 1.0%)^29^. Instead, AS408 predominantly occupied a pose at the same TM3-TM4-TM5 interface but shifted closer to TM4 with substantial rearrangements in the phenyl ring (Fig. 3e, Pose 1, Supplementary Fig. 21b). In this orientation AS408 established hydrogen bonds with E130^3.41^ alongside additional hydrophobic and VdW interactions with residues including E130^3.41^, I137^3.48^, M153^34.57^, I161^4.45^, and I168^4.52^ (Supplementary Fig. 22a). Consequently, AS408 had a smaller capacity to interact with P219^5.50^ and I214^5.45^ compared to the E130X^3.41^ variants and the equivalent AS408-hβ_2_AR interaction.

Interestingly, the simulations also revealed that AS408 reversibly sampled an alternative position closer to the intracellular end of TM4 and IL2, visiting several conformations (Fig. 3e, Pose 2, and Supplementary Fig. 22b), though only in one of the three replicates (R2 in Fig. 3d). Such fluctuations in the allosteric binding pocket of β_1_AR between several binding poses likely resulted in the attenuated affinity for the β_1_AR relative to the affinity reported for hβ_2_AR^29^.

We then experimentally substantiated that binding of AS408 at the β_1_AR occured at the same TM3-TM4-TM5 allosteric interface as at the hβ_2_AR^29^ by recording ^13^C NMR spectra of β_1_AR-E, examining signals for M153^34.57^ and an introduced I168M^4.52^ probe. Both residues were in close proximity to the AS408 in both poses in our MD simulations (Fig. 3f), meaning their NMR signals would respond to AS408 binding.

The M153^34.57^ signal gradually shifted downfield in the ^1^H dimension upon increasing addition of AS408 in the NMR spectra of both the isoprenaline-bound (Fig. 3g) and the apo β_1_AR-E (Supplementary Fig. 23a). The gradual concentration-dependent shifts were indicative of a fast exchange regime, which was consistent with the weak µM AS408 binding affinity obtained from operational model fit of the TRUPATH assay (Supplementary Table 2). Notably, nominal concentrations up to 1000 µM were necessary to approach saturation, likely due to the poor solubility of the hydrophobic AS408 and its reduced availability following incorporation into detergent micelles (Supplementary Fig. 24). In the saturated AS408-bound receptor, the major M153^34.57^ signal positions were comparable between the apo and isoprenaline-bound spectra (Supplementary Fig. 23b), suggesting similar chemical environments and therefore similar AS408 orientations. A shoulder present in the isoprenaline-bound M153^34.57^ signal (Supplementary Fig. 23b-c) implied several AS408-bound environments were populated in exchange on the µs-ms timescale, consistent with the MD simulations which also suggested several AS408 binding poses.

The signal of the I168M^4.52^ probe was generally very unresponsive towards agonist-stimulated conformational changes in the absence of AS408 (Supplementary Fig. 23d–e). In contrast, the gradual concentration-dependent changes in the I168M^4.52^ signal which occurred upon AS408 addition (Fig. 3h) further supported the µM affinity binding of the NAM within the proximity of the probe in the TM3-TM4-TM5 allosteric pocket. A similar upfield shift was also apparent for the apo receptor in the presence of AS408 (Supplementary Fig. 23f), indicating a similar binding orientation to the agonist-bound receptor. Finally, I168M^4.52^ did not respond to AS408 addition in ternary complexes of β_1_AR-E with isoprenaline and mini-G_s_ which supported the BLI-measured *k*_off_ rate constant (Supplementary Fig. 18c) in suggesting that AS408 did not interact tightly with the ternary complex at this site (Supplementary Fig. 23g).

### AS408 has minor impacts on the β_1_AR agonist pocket and core

The differences in AS408 binding orientation between the β_1_AR and hβ_2_AR, in addition to the reduced interactions with P219^5.50^ and I214^5.45^, implied that the structural mechanisms by which AS408 impacted signalling could differ between the receptors. Therefore, we next assessed the impact of AS408 on the conformational sampling of the core region of β_1_AR, inclusive of the orthosteric ligand binding pocket. We recorded NMR experiments for β_1_AR-E in the presence of increasing amounts of AS408 to obtain information on conformational changes induced by the allosteric modulator.

**Fig. 4:**
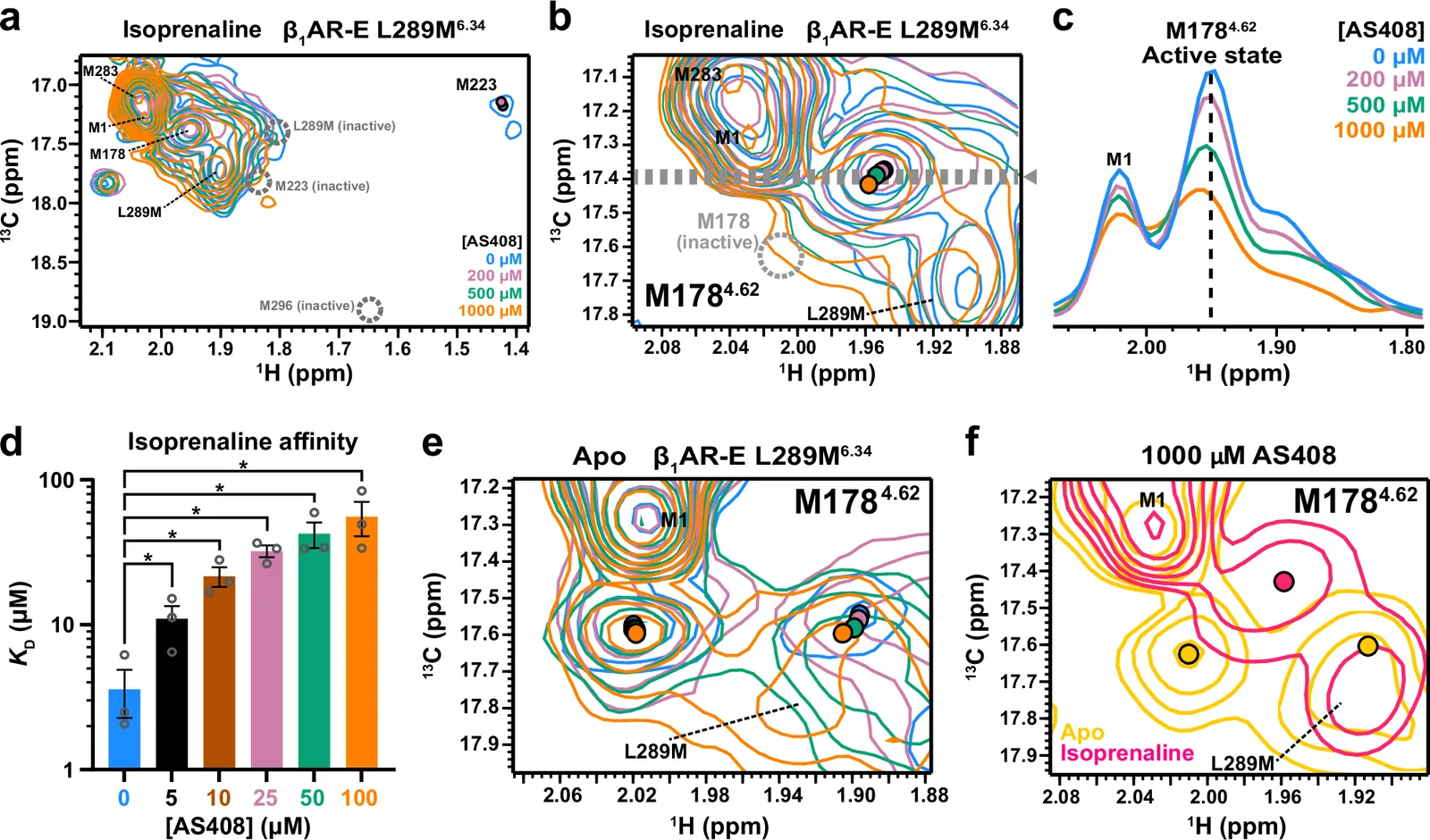
The effects of AS408 on the core and extracellular region of β_1_AR-E. **a** An overlay of 2D ^1^H-^13^C HMQC NMR spectra of isoprenaline-bound (1 mM) β_1_AR-E L289M^6.34^ with increasing AS408 concentration, recorded at 308 K. Peak assignments are indicated, with the active state signal for M223^5.54^ detectable only up to the addition of 200 µM AS408. The absence of pre-active and inactive state signals for M223^5.54^, L289M^6.34^ and M296^6.41^ during the AS408 titration series is evidenced by the empty grey dashed circles drawn at the positions taken from the apo spectrum of β_1_AR-E L289M^6.34^. **b** An overlay of 2D ^1^H-^13^C HMQC NMR spectra for isoprenaline-bound (1 mM) β_1_AR-E L289M^6.34^ with increasing concentrations of AS408, showing the M178^4.62^ probe region, recorded at 308 K. The active state M178^4.62^ position is indicated by coloured dots. The absence of an inactive state signal for M178^4.62^ is indicated by the empty grey dashed circle. **c** 1D ^1^H traces from the 2D spectra in (**b**) taken at the maxima positions of the individual M178^4.62^ active state signals as indicated by the grey line (ca. 17.4 ppm). **d** The influence of AS408 on isoprenaline affinity (*K*_D_) to β_1_AR-E, measured using the nanoBRET-based assay with Nluc-tagged β_1_AR-E. Competition assays were recorded with varying isoprenaline concentrations against bound (S)-propranolol-red at each AS408 concentration. Data are mean ± SEM of *n* = 3 independent biological experiments, each performed in duplicate. * indicates significance (*p* < 0.05), analysed on log-transformed data by one-way ANOVA with Dunnet’s post-hoc tests (5 µM, *p* = 0.0132; 10 µM, *p* = 0.0003; 25 µM, 50 µM, 100 µM, *p* < 0.0001). **e** An overlay of 2D ^1^H-^13^C HMQC NMR spectra for apo β_1_AR-E L289M^6.34^ with increasing AS408 concentration, showing the M178^4.62^ probe region. Spectra were recorded at 298 K. **f** An overlay of 2D ^1^H-^13^C HMQC NMR spectra for apo or isoprenaline-bound (1 mM) β_1_AR-E L289M^6.34^ in the presence of 1000 µM AS408, showing the M178^4.62^ probe region. Spectra were recorded at 308 K. The colours for spectra in (**b**) and (**e**) are the same as in (**a**).

In ^13^C NMR spectra of isoprenaline-bound β_1_AR-E, the methyl probes in the receptor core informing on the regions around the PIF motif remained conformationally dynamic upon increasing addition of AS408. For comparison, whilst the aromatic E130X^3.41^ variant signals for the M223^5.54^ and M296^6.41^ probes appeared in positions indicative of populating the inactive state (Fig. 2d), no signals were detected at these positions for β_1_AR-E in the presence of AS408 (Fig. 4a). Thus, the receptor core signals remained conformationally broadened, consistent with the slow-to-intermediate exchange regime sampled by this region of β_1_AR-E when occupying the pre-active/active state equilibrium^14^ (Fig. 1a). The weak signal at the active state position of M223^5.54^ was gradually lost upon increasing AS408 addition (Fig. 4a), indicating the equilibrium did shift slightly away from the active state. However, the region of agonist-bound β_1_AR-E around the PIF motif clearly did not substantially populate the inactive state when saturated with AS408.

In spectra of apo β_1_AR-E, increasing addition of AS408 gradually shifted the M223^5.54^ and M296^6.41^ signals, which was consistent with fast exchange related to the low micromolar affinity of the modulator. Notably, M296^6.41^ shifted upfield in the ^1^H dimension (Supplementary Fig. 25a) to a position not previously observed for the E130X^3.41^ variants or for β_1_AR when bound to any of a wide range of agonists^14,27^. M223^5.54^ showed smaller changes downfield in ^1^H (Supplementary Fig. 25b), indicating the region directly proximal to the PIF motif was undergoing smaller rearrangements. Therefore, these data suggested that AS408 binding impacted the conformation of the apo receptor core. We corroborated the effects of AS408 on the inactive state using propranolol-bound β_1_AR-E. Similar chemical shift changes were observed in ^13^C NMR spectra upon increasing addition of AS408 (Supplementary Fig. 25c–e), confirming that modulator binding affected the receptor core in the inactive β_1_AR-E.

The M178^4.62^ probe, reporting on the conformational sampling of the orthosteric binding pocket^14,27^, had shown a clear occupation of the inactive/pre-active state equilibrium for isoprenaline-bound β_1_AR aromatic E130X^3.41^ variants (Fig. 2d, Supplementary Fig. 13c). In contrast, the orthosteric pocket in the AS408-bound β_1_AR-E maintained the active state conformation in the presence of isoprenaline (Fig. 4b). We noted that the intensity of the M178^4.62^ active state signal decreased with increasing AS408 addition due to peak broadening (Fig. 4c), which suggested that AS408 reduced the binding affinity of isoprenaline to β_1_AR-E. In cell ligand affinity measurements corroborated this conclusion (Supplementary Figs. 26–27), in which AS408 induced a significant (*p* < 0.05) concentration-dependent reduction in the isoprenaline affinity to β_1_AR-E (Fig. 4d, Supplementary Table 3). This decrease was consistent with the affinity cooperativity value of log *α* = -1.5 obtained from analysis of the TRUPATH data (Supplementary Fig. 19a). The changes to isoprenaline affinity primarily originated from decreases in the isoprenaline association rate constant to β_1_AR-E (Supplementary Fig. 26f), whereas the dissociation rate constant did not change in a concentration-dependent manner (Supplementary Fig. 26g).

Little change was observed for the M178^4.62^ signal in the apo receptor (Fig. 4e) or the propranolol-bound receptor (Supplementary Fig. 25f) upon addition of AS408, indicating the binding pocket maintained a conformation in agreement with the inactive state under these conditions. The differences between apo and isoprenaline-bound β_1_AR-E further emphasised that AS408 exerted much less control than the full agonist over the orthosteric pocket conformation (Fig. 4f).

Finally, the M90^2.53^ probe, which reports on the dynamics of β_1_AR directly below the orthosteric binding pocket, showed small concentration-dependent chemical shift changes upon addition of AS408 to propranolol-bound β_1_AR-E (Supplementary Fig. 25g). These shifts were similar in magnitude to M223^5.54^, and further supported that AS408 had a more limited impact on the allosteric network in the region between the ligand and the PIF motif.

Overall, the NMR spectra suggested that the conformational sampling of the orthosteric pocket was little affected by AS408 binding and remained primarily determined by the properties of the orthosteric ligand, whilst AS408 exerted some influence on the receptor core arrangement below this pocket. These data contrasted with the substantial changes observed for the agonist-bound E130X^3.41^ variants which populated the inactive/pre-active state equilibrium in these regions.

### AS408 uncouples the β_1_AR intracellular surface from agonist

To understand how AS408 affected the allosteric signal propagation from the orthosteric pocket towards the G protein binding surface of the receptor, we assessed the impact of AS408 on the intracellular regions of β_1_AR-E using a combination of ^19^F and ^13^C NMR.

**Fig. 5:**
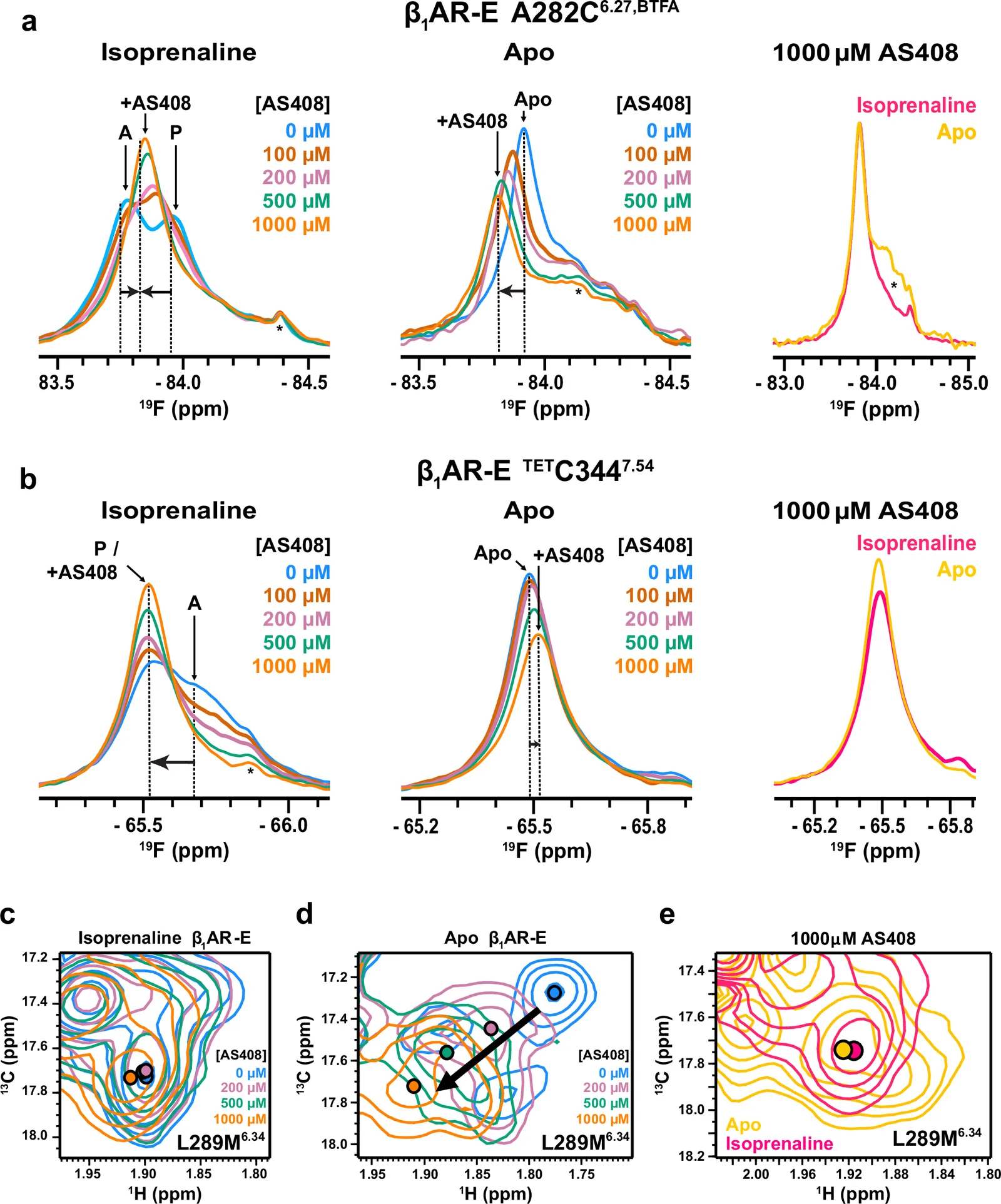
AS408 stabilises an alternative conformation on the intracellular surface of β_1_AR-E. **a** Overlay of 1D ^19^F NMR spectra for β_1_AR-E A282C^6.27,BTFA^ in the presence of increasing concentrations of AS408, recorded at 298 K. Left, isoprenaline-bound (1 mM) β_1_AR-E. Middle, apo β_1_AR-E. Right, an overlay of spectra for isoprenaline-bound and apo β_1_AR, respectively, in the presence of 1000 µM AS408. Signal positions of active state (A), pre-active state (P), apo, and AS408-bound receptor (+AS408) are indicated with dashed lines. Arrows indicate the direction of shift changes with increasing addition of AS408. * indicates degradation products. **b** Overlay of 1D ^19^F NMR spectra for β_1_AR-E ^TET^C344^7.54^. All details are as in (**a**). **c** An overlay of 2D ^1^H-^13^C HMQC NMR spectra for isoprenaline-bound (1 mM) β_1_AR-E L289M^6.34^ showing the L289M^6.34^ probe region with increasing AS408 concentration, recorded at 308 K. **d** An overlay of spectra for apo β_1_AR-E L289M^6.34^ showing the L289M^6.34^ probe region with increasing AS408 concentration, recorded at 298 K. The black arrow indicates the direction of chemical shift change induced by AS408. **e** An overlay of 2D ^1^H-^13^C HMQC NMR spectra for isoprenaline-bound (1 mM) and apo β_1_AR-E L289M^6.34^, respectively, with 1000 µM AS408, showing the similarity of the L289M^6.34^ signal. Spectra were recorded at 308 K.

For isoprenaline-bound β_1_AR-E with ^19^F labelling at A282C^6.27,BTFA^, the ^19^F signals corresponding to the active and pre-active receptor states gradually decreased in intensity with increasing addition of AS408, whilst a new peak became increasingly apparent (Fig. 5a, left, Supplementary Fig. 28a). The narrower signal linewidth suggested reduced conformational broadening (Supplementary Fig. 28a). Therefore, AS408 binding to the isoprenaline-stimulated receptor lowered the relative plasticity of the intracellular TM6 region compared to the active and pre-active states. Interestingly, the position of the signal for the AS408-bound receptor did not match with any previously observed state, including the inactive state, suggesting β_1_AR adopted an alternative conformation in the presence of AS408. The solvent exposure of this AS408-stabilised conformation was seemingly intermediate between the active and pre-active states of β_1_AR, based on the relative chemical shift positions^14,59^.

Addition of AS408 to the apo receptor shifted the signal gradually downfield, consistent with a fast exchange regime due to the weak binding of the modulator (Fig. 5a, centre). Notably, in receptors saturated with AS408, the signal position and linewidth of the apo receptor aligned well with the equivalent spectrum of the isoprenaline-bound β_1_AR-E (Fig. 5a, right). Therefore, AS408 stabilised the same alternative conformation at the cytoplasmic tip of TM6 irrespective of the ligand efficacy information encoded in the orthosteric pocket.

A similar decrease of the isoprenaline-bound active state population was also observed around the TM7/IL4 intracellular region with increasing addition of AS408, using the ^TET^C344^7.54^ probe (Fig. 5b, left). In contrast to the A282C^6.27,BTFA 19^F spectra, an increase in signal intensity was observed at a position similar to the pre-active state. Again, a gradual reduction in the signal linewidth indicated that AS408 binding reduced conformational broadening of the isoprenaline-bound receptor (Supplementary Fig. 28b). In the equivalent apo receptor spectra, the ^TET^C344^7.54^ signal gradually shifted upfield with increasing AS408 concentration, to a position that matched the isoprenaline-bound β_1_AR-E when the receptor was saturated with AS408 (Fig. 5b, centre and right). Thus, the AS408-bound receptor occupied a conformation in the TM7/IL4 region that was more similar in chemical environment to the pre-active state than the inactive state.

We extended the coverage at the cytoplasm using the introduced ^13^C methyl-methionine probe L289M^6.34^, which is a reliable reporter of the local conformational environment on the intracellular side of TM6^14^. Addition of AS408 resulted in little change to L289M^6.34^ for the isoprenaline-bound receptor even at high AS408 concentrations (Fig. 5c), where the signal remained in a position associated with the active-state (Supplementary Fig. 28c). In contrast, the apo receptor L289M^6.34^ signal strongly responded to the addition of AS408 (Fig. 5d), as did the equivalent signal in the inactive propranolol-bound receptor (Supplementary Fig. 28d). When saturated with AS408, the L289M^6.34^ signal position for apo β_1_AR and propranolol-bound β_1_AR overlaid well with the signal of the isoprenaline-bound receptor (Fig. 5e and Supplementary Fig. 28e, respectively). This similarity in chemical shifts indicated that, in the presence of AS408, this region of TM6 occupied a chemical environment that was more similar to the active state. This contrasted with our earlier ^19^F NMR observations at A282C^6.27,BTFA^, located two helical turns below L289M^6.34^, which indicated adoption of an alternative conformation differing from the active state.

Together, our NMR data strongly suggested that AS408 reduced the conformational plasticity around the G protein binding surface and stabilised alternative intracellular conformational arrangements which were not consistent with the concerted equilibrium of states typically populated during receptor activation. Importantly, the cytoplasmic organisation of β_1_AR-E had become uncoupled from the allosteric signalling network and its regulation by the orthosteric binding site, in that the alternative conformation was adopted independently of the receptor state that was occupied prior to AS408 addition.

**Fig. 6:**
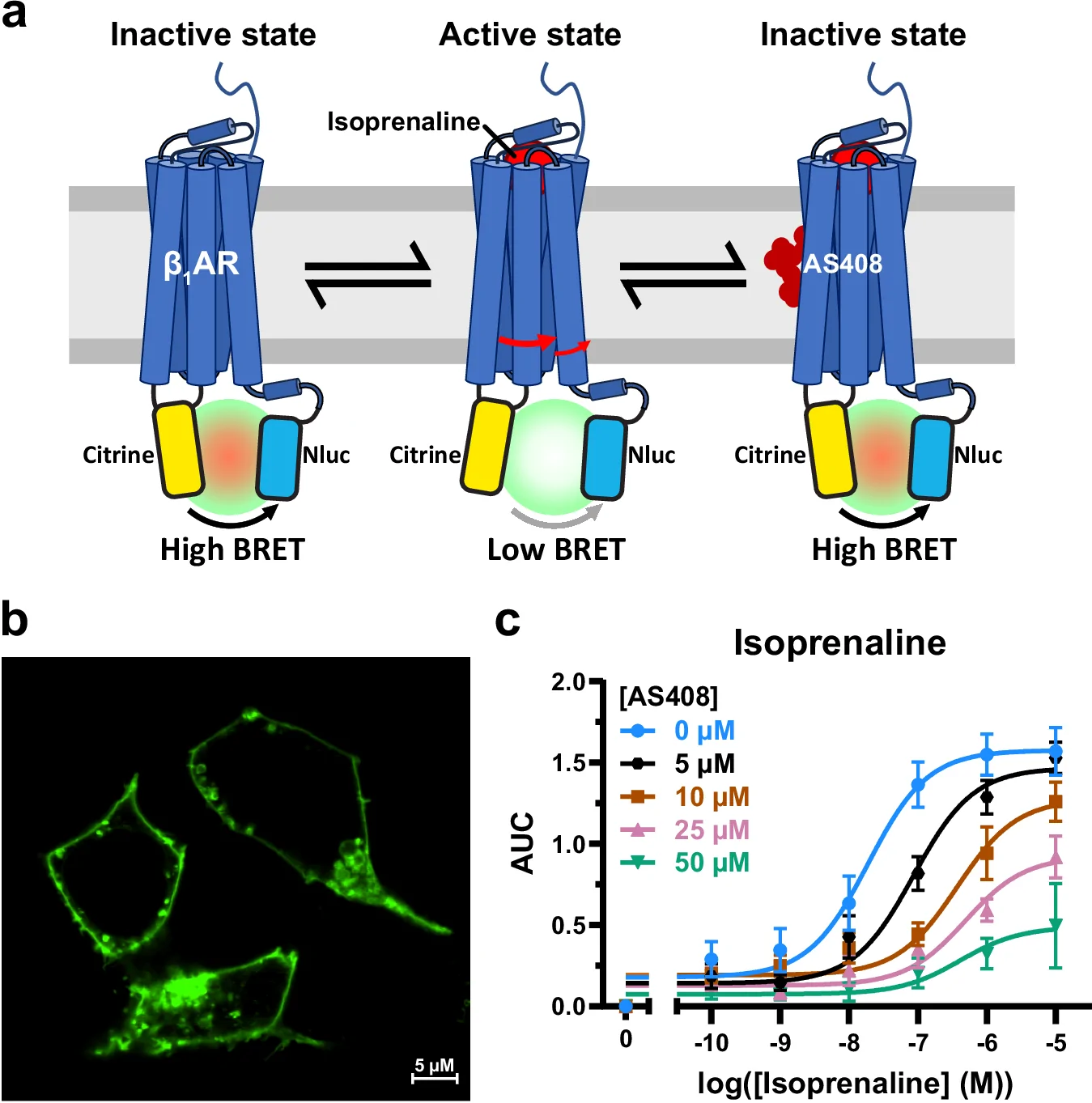
A conformational biosensor validates AS408-induced β_1_AR-E conformational rearrangements observed in vitro. **a** Schematic of the β_1_AR-E BRET conformational sensor which reports on changes in intramolecular BRET (bioluminescence resonance energy transfer) signal depending upon ligand binding. **b** Representative image of HEK293A cells stably expressing the β_1_AR-E BRET sensor in the YFP channel. **c** Concentration response curves for the β_1_AR-E BRET sensor when co-stimulated with the NAM AS408 and isoprenaline. Data fitted using the 3 parameter logistic equations. Data are mean ± SEM of *n* = 4 biological measurements, each performed in duplicate.

Based upon these observations, we sought to provide an in-cell validation of the actions of AS408 on the intracellular surface of β_1_AR-E. Previous studies have highlighted the utility of intramolecular FRET- (Förster resonance energy transfer) or BRET-based intramolecular sensors to report on conformational changes in GPCRs^60–63^ including the hβ_1_AR^64^. We therefore generated a β_1_AR-E BRET-sensor by inserting Citrine into IL3 and ligating Nluc onto the C-terminus (Fig. 6a, Supplementary Fig. 29a) and confirmed plasma membrane localisation of this receptor using confocal microscopy (Fig. 6b). We validated that the β_1_AR-E BRET-sensor reported ligand-dependent conformational changes through addition of isoprenaline and propranolol. Consistent with similar intramolecular sensors^60–62^, we observed a concentration-dependent loss of BRET signal for isoprenaline (pEC_50_: 7.74 ± 0.17) with very little change upon application with propranolol (Supplementary Fig. 29b). Propranolol was confirmed to act as an antagonist at this BRET-sensor through competition assays between the agonist and antagonist (Supplementary Fig. 30a).

We subsequently preincubated the conformational sensor with increasing concentrations of AS408 and determined the degree of activation upon addition of isoprenaline. Consistent with the NMR data, we saw decreases in β_1_AR-E conformational plasticity induced by isoprenaline (Fig. 6c). Therefore, AS408 prevented the agonist from inducing activation in TM6, without itself having substantial impacts on the BRET-sensor (Supplementary Fig. 30b–c). We were unable to detect effects of AS408 upon basal receptor activity using this β_1_AR-E BRET-sensor, possibly due to the presence of thermostabilising mutations within the construct used (Supplementary Fig. 29b). Using the same curve fitting methodology as described for the TRUPATH biosensor data, we applied the operational model of allostery to the isoprenaline concentration-response curves (Supplementary Fig. 19b). We obtained similar parameters for log *α* (−1.4 ± 0.4), log *β* (−1.2 ± 0.5), and log(*αβ*) (−2.6 ± 0.6) when compared to the TRUPATH analysis (Supplementary Table 2), again confirming AS408’s ability to reduce both the affinity and efficacy of the orthosteric ligand.

We further investigated the potential for probe dependency (a phenomenon where an allosteric modulator’s effect on a receptor differs depending on the orthosteric ligand) for AS408 at the β_1_AR using the β_1_AR-E BRET-sensor. BRET signal changes were assessed for classical and G protein/β-arrestin non-biased orthosteric ligands, relative to the reference ligand isoprenaline used in this study, from three broad classifications, including agonists (adrenaline, formoterol), a partial agonist (dobutamine), and an antagonist (propranolol) with increasing concentrations of AS408. Adrenaline, formoterol, and dobutamine all activated the receptor and we observed concentration-dependent decreases in ΔBRET signal with increasing AS408 addition (Supplementary Fig. 31), analogous to that observed for isoprenaline (Fig. 6c). Although the signal-to-noise window was narrower for the partial agonist, noticeable decreases in ΔBRET were still present. We also observed little change in the propranolol signal with increasing concentrations of AS408, as expected. Consequently, we concluded that AS408 acts similarly at the β_1_AR-E biosensor when stimulated with different non-biased orthosteric ligands tested in this study.

Overall, AS408 acted as a NAM to generate a signalling deficient receptor independently of orthosteric ligand efficacy by uncoupling ligand binding from intracellular activation.

### AS408 and E130X^3.41^ act by independent allosteric pathways

The adoption of an alternative receptor conformation, driven by uncoupling of the allosteric network relating the orthosteric site to the intracellular surface of β_1_AR, was distinct to AS408. However, the dynamic nature of agonist-bound β_1_AR-E impeded a meticulous analysis of the receptor core region upon AS408 binding as the signals for the NMR probes in this region remained unfavourably broadened by conformational exchange (Fig. 4a). Since the E130X^3.41^ mutations repositioned the conformational equilibria connecting the same states also sampled by β_1_AR-E, we questioned whether we could utilise these differences in equilibria positions inherent to the E130X^3.41^ mutations to further ascertain the impact of AS408 binding on the dynamic core region of β_1_AR by NMR.

The 3.41 position has been suggested as important in the binding of AS408 to the hβ_2_AR since E122^3.41^ mutations affected the extent to which AS408 reduced signalling by removal of a hydrogen bond between E^3.41^ and AS408^29^. The E130^3.41^ in β_1_AR also formed substantial interactions with AS408 in Pose 1 (Fig. 3e). Therefore, we first assessed the effects of AS408 on G protein coupling to β_1_AR E130X^3.41^ variants. Titration of AS408 decreased the activity and potency of E130X^3.41^ variants in TRUPATH assays with no substantial difference in the effects of AS408 between the variants and β_1_AR-E (Supplementary Fig. 32). These data implied that changing the side chain at the E130^3.41^ position did not have a major impact on AS408 binding to β_1_AR. Consequently, we leveraged the shift in receptor state equilibria positions associated with the E130X^3.41^ mutations to observe how the orthosteric ligand and AS408 simultaneously influenced the allosteric network in this region by NMR.

**Fig. 7:**
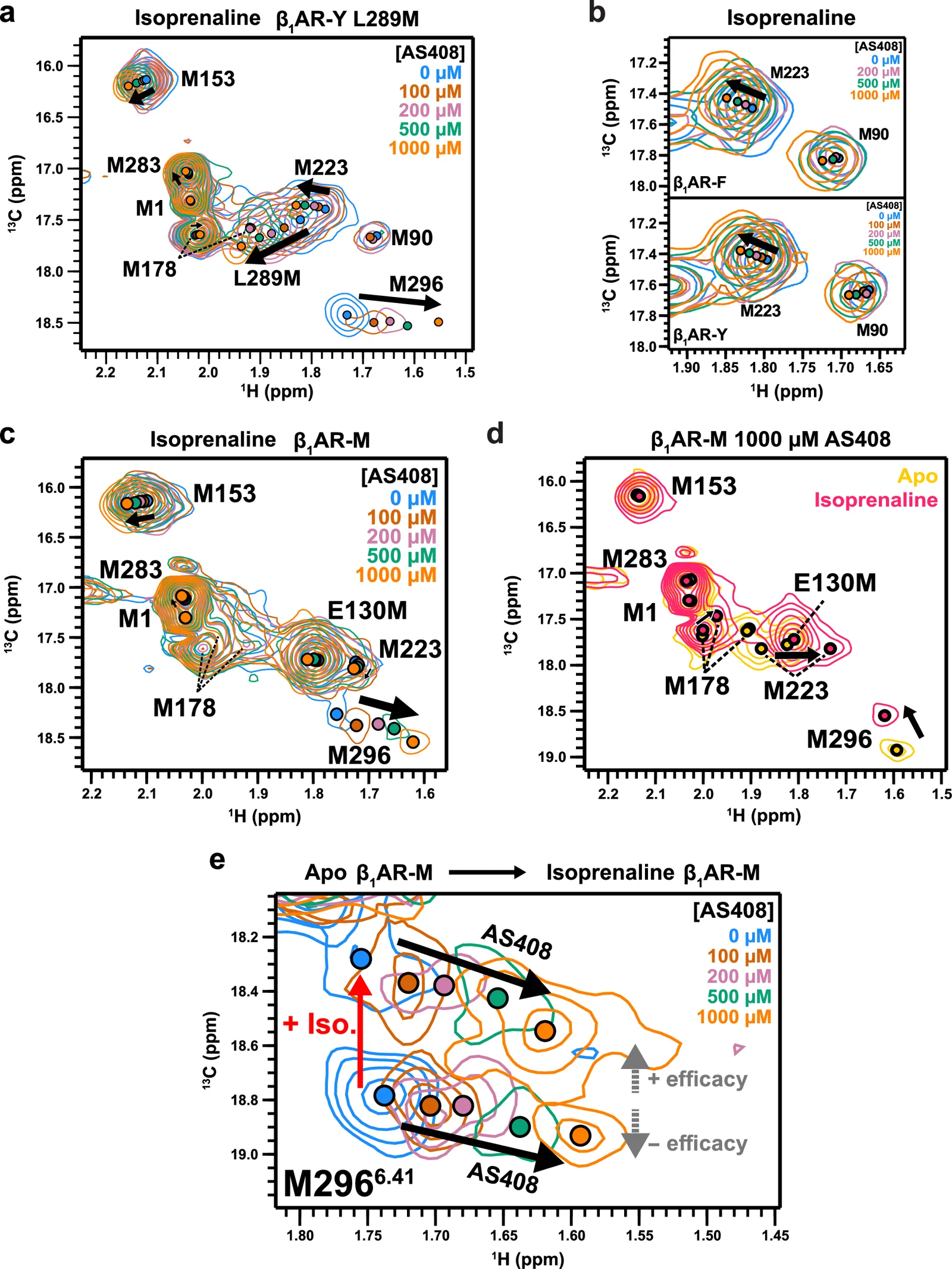
AS408 and E130X^3.41^ mutations impact β_1_AR conformational equilibria through independent pathways. **a** An overlay of 2D ^1^H-^13^C HMQC NMR spectra for isoprenaline-bound (1 mM) β_1_AR-Y L289M^6.34^ with increasing AS408 concentration, recorded at 308 K. Arrows indicate chemical shift changes with increasing addition of AS408. **b** 2D ^1^H-^13^C HMQC NMR spectra for isoprenaline-bound (1 mM) β_1_AR-F (top) or β_1_AR-Y (bottom) showing the M223^5.54^ and M90^2.43^ probe signals. Spectra were recorded with increasing AS408 concentration at 308 K. Arrows indicate chemical shift changes with increasing addition of AS408. **c** An overlay of 2D ^1^H-^13^C HMQC NMR spectra for isoprenaline-bound (1 mM) β_1_AR-M with increasing AS408 concentration, recorded at 308 K. **d** A comparison of 2D ^1^H-^13^C HMQC NMR spectra for apo or isoprenaline-bound (1 mM) β_1_AR-M, respectively, with 1000 µM AS408 added, showing the changes in receptor conformational equilibria upon stimulation with the full agonist. Arrows indicate chemical shift changes induced by isoprenaline addition. **e** An overlay of 2D ^1^H-^13^C HMQC NMR spectra for β_1_AR-M showing the M296^6.41^ probe region with increasing AS408 concentration in apo or isoprenaline (Iso., 1 mM) conditions, respectively, and recorded at 308 K. Red arrows indicate chemical shift changes for M296^6.41^ peaks between apo and isoprenaline spectra, related to activation by full agonist stimulation. Black arrows indicate the changes in signal positions related to the increasing addition of AS408. Changes in the ^1^H dimension indicate AS408-dependent alterations of the local environment, whereas changes in the ^13^C dimension relate to the efficacy-dependent repositioning of the rapidly exchanging inactive/pre-active state equilibrium towards the pre-active state^27^.

^1^H-^13^C NMR experiments indicated that the general effects of AS408 on the conformational changes of β_1_AR were maintained in the low activity aromatic E130X^3.41^ variants. The chemical shift changes observed with increasing AS408 addition for β_1_AR-Y (Fig. 7a–b, Supplementary Fig. 33a–b) and β_1_AR-F (Fig. 7b, Supplementary Fig. 33c–d) were reminiscent of those observed for apo or propranolol-bound β_1_AR-E, which further supported our earlier observations that the AS408-stabilised alternative conformation differed from the inactive state. Both variants demonstrated the effects of AS408 on the receptor core around the PIF motif in agonist-bound and apo receptors, respectively, through gradual shifts in M223^5.54^ (Fig. 7b) and M296^6.41^ (Supplementary Fig. 34). M90^2.43^ and M178^4.62^ in proximity to the orthosteric pocket were less affected by AS408 addition (Fig. 7b, Supplementary Fig. 33), which confirmed that the orthosteric pocket conformation was predominantly determined by the bound agonist.

We further utilised β_1_AR-M as a representative hydrophobic E130X^3.41^ variant as the isoprenaline-bound β_1_AR-M favoured the pre-active state whilst still also populating the inactive and active states (Fig. 2b–c), thus providing a more active receptor than β_1_AR-F or β_1_AR-Y. BLI dose-response data indicated that the β_1_AR-M and β_1_AR-E responded similarly to AS408 in detergent with respect to mini-G_s_ binding kinetics (Supplementary Fig. 35a–c).

The ^1^H-^13^C NMR spectra of β_1_AR-M with increasing addition of AS408 also revealed similar patterns in chemical shift changes compared to β_1_AR-E (Fig. 7c, Supplementary Fig. 35d) confirming the adoption of the alternative conformations for the intracellular probes in β_1_AR-M. Likewise, the conformational sampling in the orthosteric binding pocket remained little perturbed by AS408 in isoprenaline-bound β_1_AR-M, as revealed by maintaining the minor active state population (Supplementary Fig. 35e). Importantly, the core probes M223^5.54^ and M296^6.41^ were still visible and their changes upon addition of isoprenaline to AS408-bound β_1_AR-M clearly revealed the activation of the receptor core region (Fig. 7d). These spectral changes confirmed the activating conformational transitions in the PIF motif occurred in the AS408-bound receptor when stimulated by an agonist.

The signal of the M296^6.41^ probe in this region of β_1_AR-M provided clear insight into how the E130X^3.41^ mutation, the orthosteric ligand, and AS408 binding exerted separate influences on the conformational equilibria of the receptor. The gradual ^1^H upfield shift of M296^6.41^ observed upon AS408 binding to inactive β_1_AR-E (Supplementary Fig. 25a,d) was likewise present for both isoprenaline-bound and apo β_1_AR-M (Fig. 7e). Furthermore, the concurrent ^13^C upfield shifts, which were indicative of isoprenaline-induced activation^27^, suggested that the receptor core was receiving instructions from the orthosteric binding pocket but the communication network was also being rewired by AS408. The latter resulted in unproductive signal propagation further downstream of the PIF motif. Similar upfield ^13^C shifts upon agonist stimulation were observed for β_1_AR-F and β_1_AR-Y (Supplementary Fig. 34), though smaller in magnitude than for β_1_AR-M as expected for the lower activity of these variants.

We also noted small downfield shifts in ^13^C of the M296^6.41^ signal for β_1_AR-M, β_1_AR-F, and β_1_AR-Y with increasing AS408 concentration (Supplementary Fig. 34). These shifts suggested a subtle shift in the pre-active/inactive state equilibrium towards the inactive state^27^ and indicated AS408 also had minor effects on the orthosteric ligand-controlled receptor core equilibrium.

Overall, the E130X^3.41^ variant data demonstrated the separate but concurrent influences on the receptor conformational equilibria. These included the activating effect of the orthosteric agonist, the inactivating effect of E130M^3.41^ shifting equilibria towards the pre-active state, and the conformational alterations induced by AS408 binding.

## Discussion

The plasticity and conformational flexibility of GPCRs are critical properties which influence the effects of drugs on signalling. Modulating the energy landscapes of GPCRs^38–40,65^ has been rigorously applied towards a better understanding of how orthosteric ligands work^14,16,18,19,24,26–28^. Here we endeavoured to investigate the role of manipulating receptor dynamics in GPCR allostery through alterations in the chemical environment of an allosteric pocket, using a combination of pharmacological assays, MD simulations, and biophysical and NMR conformational analyses.

**Fig. 8:**
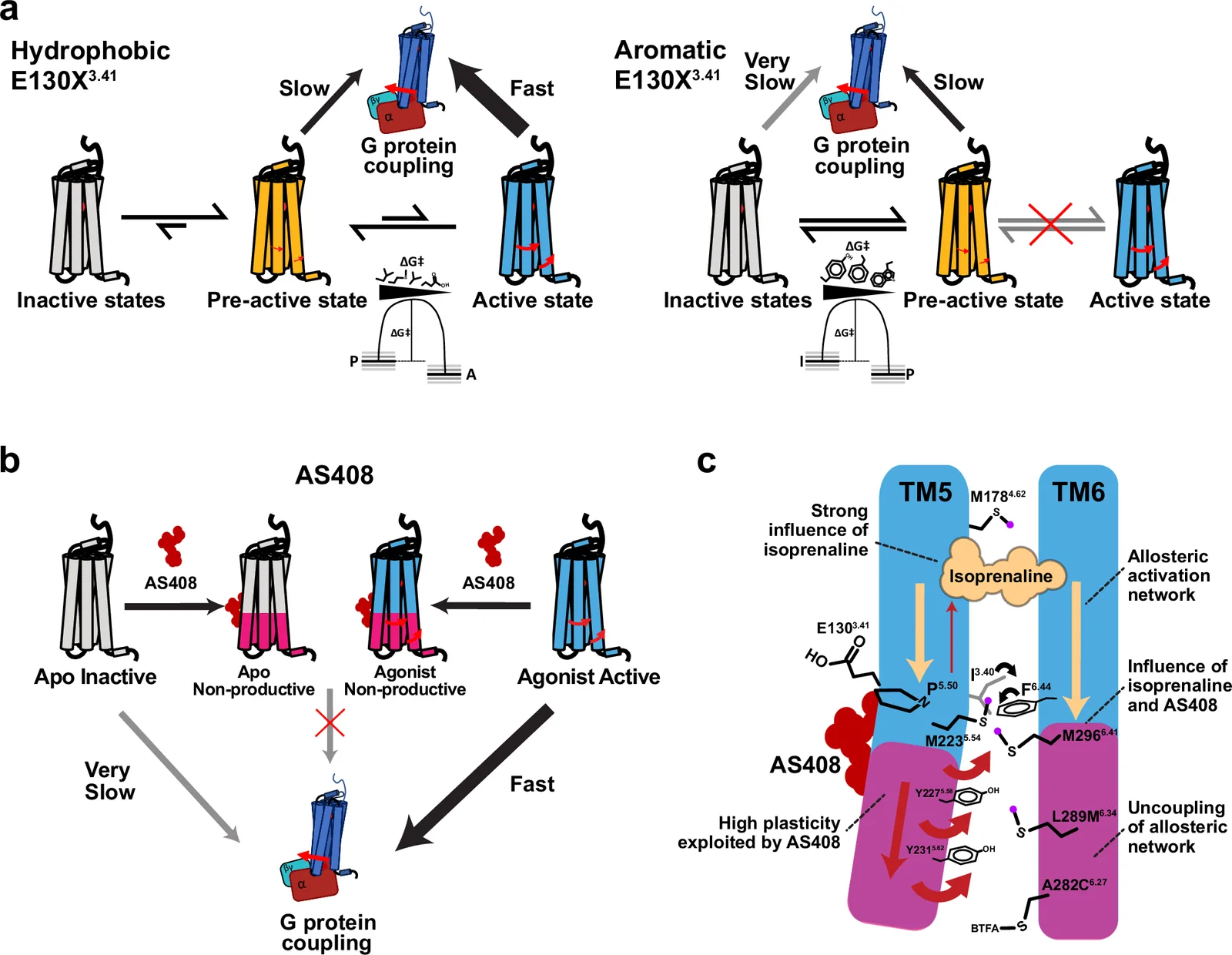
Mechanisms of allosteric modulation at the β_1_AR. **a** E130X^3.41^ mutations shift orthosteric agonist-stabilised conformational equilibria, affecting the extent to which ligand efficacy information is transduced through the allosteric network of microswitches in β_1_AR. Shifts in equilibria away from the active state (A) reduce G protein coupling efficiency in a side chain-dependent rheostat. Mutations variably stabilise receptor states, favouring stabilisation of the pre-active state (P) in receptors with hydrophobic E130X^3.41^ mutations (left) or stabilisation of inactive states (I) in receptors with aromatic mutations (right). **b** The NAM AS408 uncouples ligand efficacy information in the orthosteric pocket from the intracellular surface, resulting in a non-productive conformation (pink) with poor G protein coupling efficiency. **c** The influences of orthosteric agonist and allosteric ligand AS408 on receptor conformational dynamics. Below the PIF motif, AS408 impacts the TM5-TM6 interface and uncouples this region from the agonist efficacy information in the allosteric activation network. Above the PIF motif, the conformational dynamics are predominantly impacted by the orthosteric agonist with minor effects from AS408. Around the PIF motif, influences of both the orthosteric agonist and the NAM AS408 affect the conformational equilibrium.

Emulating the effects of allosteric drug binding through E130X^3.41^ mutations in a TM3-TM4-TM5 allosteric pocket of β_1_AR resulted in changes to the equilibrium populations of previously characterised receptor states, favouring the pre-active and inactive states (Figs. 1–2). Due to the different G protein coupling efficacies of each of these states in the β_1_AR equilibria (Fig. 1a), the E130X^3.41^ variants behaved as a rheostat for G protein signalling (Fig. 1e). The conformational equilibria positions were altered in an analogous manner to the impact of orthosteric partial agonists^14,16,26–28^, where conformations were altered through coordinated changes within the proposed framework of receptor activation^31–34,36^. Hence, the mutations at X^3.41^ regulated transduction of ligand information in a residue-type dependent manner by effectively imparting fractional efficacy effects on the full agonist to control β_1_AR signalling (Fig. 8a), resulting in the changes in maximal response and potency observed in functional data (Fig. 1e). Our data underpin the conformational selection of orthosteric ligand-stabilised functional states as a fundamental strategy by which agonist-dependent signalling can be modulated by allosteric therapeutics, supporting observations at the µ-opioid receptor^31^, the neurotensin receptor^33^, the cannabinoid receptor^32^, and the β_1_AR^36^.

Our MD simulations of the TM3-TM4-TM5 allosteric pocket indicated that the highly inactivating aromatic E130X^3.41^ variants had increased interactions between the E130X^3.41^ position and neighbouring residues including I214^5.45^ and P219^5.50^ in the conserved P^5.50^-I^3.40^-F^6.44^ microswitch (Fig. 2e–f). We inferred that the resulting changes to the receptor conformational landscape were communicated to the rest of the allosteric activation pathway via the PIF motif, to concertedly stabilise pre-active or inactive states. This mechanism was similar to the action of the small molecule modulator AS408 at the hβ_2_AR^29^.

In contrast, despite the overall similarity in the allosteric pocket’s structure and sequence between hβ_2_AR and β_1_AR, we found that AS408 acted via a discrete mechanism by stabilisation of alternative β_1_AR conformations that were distinct from those stabilised in E130X^3.41^ variants or by ligands used in previous studies^14,26,27^. Thus, we attributed the substantial decreases in G protein coupling (Fig. 3a) to the occupation of this ‘non-productive’ conformation, which had a low affinity for G proteins (Fig. 8b). Stabilisation of energy minima outside the equilibria of endogenous states has also been noted at the muscarinic M2 receptor upon binding of the PAM LY2119620^37^, supporting this as another available mechanism for allosteric modulators.

AS408 predominantly acted on the intracellular conformation with far reaching allosteric effects across the G protein binding surface (Figs. 5–6). Outward displacement of TM6 is an essential conformational rearrangement for efficient G protein coupling to Class A GPCRs^3^. The considerable structural changes in TM6 (Fig. 5) indicated AS408 disrupted these key transitions and lead to the observed reduction in β_1_AR signalling. Previous studies have found the intracellular side of β_1_AR to be more loosely coupled to the orthosteric pocket in the agonist-bound active state^14,22^. This inherent plasticity was exploited by AS408 to potentiate the uncoupling of the intracellular surface from the orthosteric pocket, as the non-productive conformation showed independence from the presence of ligand in the orthosteric pocket (Figs. 5–6). In contrast, the influence of orthosteric ligands from differing classes, including agonist (Figs. 4,7) and antagonist (Supplementary Fig. 25), remained apparent in the strongly coupled receptor core and orthosteric pocket. Accordingly, the effective magnitude of the allosteric changes induced by AS408 varied across the receptor (Fig. 8c) in contrast to the E130X^3.41^ mutations which had a coordinated effect on equilibria populations.

Although our investigations have utilised an avian GPCR, our data demonstrate the breadth in both binding poses and mechanisms available to allosteric modulators at GPCRs. The binding poses of AS408 at β_1_AR revealed in MD simulations (Fig. 3e) were distinct relative to the AS408 interaction with hβ_2_AR^29^ and likely accounted for the disparate mechanisms of allosteric modulation. The substantial reductions in interactions with residues around the PIF motif, including with I214^5.45^ and P219^5.50^ (Fig. 3e, Supplementary Fig. 22), meant that AS408 could not impart a stabilising effect which could be communicated through the allosteric activation network. Interestingly, solution studies of β_1_AR have indicated the TM5 region in the two turns below the PIF motif is amongst the most dynamic in the active state of β_1_AR^22^, and includes residues which contact AS408 in binding pose 1 (Supplementary Fig. 36). AS408 may impart its modulator activity by directly contacting this highly dynamic region on TM5 which could explain the primary localisation of AS408-induced conformational rearrangements at the intracellular surface.

The reorientation of AS408 in the TM3-TM4-TM5 allosteric pocket in MD simulations of β_1_AR resulted in altered interactions relative to AS408-bound hβ_2_AR, including between the phenyl ring and the 3.48 position. The TM3-TM4-TM5 allosteric pocket is generally well conserved between the β_1_AR, hβ_1_AR, and hβ_2_AR (Supplementary Table 4), but notably the 3.48 position has bulkier Ile and Leu residues in β_1_AR and hβ_1_AR, respectively, compared to a smaller Val residue in hβ_2_AR. This difference may favour the differing AS408 binding pose at the β_1_AR to reduce steric clashes between the AS408 phenyl ring and the larger Ile side chain (Supplementary Fig. 21a) and consequently AS408 may also reorient similarly at hβ_1_AR. However, differences in overall receptor dynamics between β_1_AR and hβ_1_AR^44^ could also contribute to differing mechanisms.

Overall, our study emphasises not only the importance of conformational variability in allosteric modulation of GPCR signalling, but also the synergy of orthogonal experimental approaches for mechanistic studies into receptor modulation. It is currently unclear if other GPCR therapeutics also exhibit several binding poses when bound to different human receptors that may increase off-target interactions. Our multi-dimensional approach presents an attractive opportunity for more effective design of subtype-selective modulators within this emerging field^13^, as information regarding the conformational plasticity of drug-receptor complexes may aid in concentrating structure guided screening efforts to improve selective targeting of allosteric sites in GPCRs.

In summary, we provide insight into the structural mechanisms of negative allosteric modulation at the β_1_AR, and the variable conformational dynamics by which the GPCR energy landscape can be altered. The mechanistic differences between E130X^3.41^ and AS408 demonstrate how varying influences in the same allosteric pocket can differentially affect conformational equilibria.

## Methods

### β_1_AR constructs—Biophysics

The β_1_AR constructs were based on the minimally thermostabilised β_1_AR construct^27^. The β_1_AR-E construct differed from wild type turkey (*Meleagris gallopavo*) β_1_AR as described previously^14,26,27^. The N-terminus (Δ3-32), C-terminus (Δ368-483) and IL3 (Δ244-271) were truncated^66^. Expression yields were improved by the single residue mutation C116L^3.27^ ^67^. The palmitoylation site C358A^8.59^ was removed to improve receptor purification. Thermostabilising mutations were introduced at R68S^1.59^, F327A^7.37^ and F338A^7.48^^68^. Additional methionine mutations M44L^1.35^, M48L^1.39^, M179L^4.63^, and M281A^6.26^ were introduced to reduce spectral crowding^27^. The E130^3.41^ position was mutated in E130X^3.41^ variants to Val, Met, Ile, Leu, Trp, Phe, or Tyr for use in BLI assays and NMR experiments. ^1^H-^13^C NMR experiments examining TM6 used the mutation L289M^6.34^, which has been previously characterised^14,17^. An additional methionine I168M^4.52^ was introduced to probe the TM3-TM4-TM5 allosteric site. See Supplementary Fig. 1a for an overview of the receptor construct.

The ^19^F NMR experiments utilised modified constructs for site specific labelling with small molecule ^19^F probes^26^. The C85V^2.48^ and C163L^4.47^ mutations were made to remove off-target labelling. ^19^F NMR experiments examining TM7 using ^TET^C344^7.54^ were labelled at the native C344^7.54^ residue and experiments examining TM6 with A282C^BTFA,6.27^ used constructs with further C344S^7.54^ and A282C^6.27^ mutations. See Supplementary Fig. 1b.

BLI experiments used receptor constructs with an avi-tag sequence (GLNDIFEAQKIEWHE) introduced at the receptor N-terminus between M1 and G2 via mutagenesis. Agonist affinity measurements used receptor constructs with a FLAG-Nluc sequence inserted between M1 and G2. Mutations were introduced using site directed mutagenesis via PCR with Q5 polymerase (New England Biolabs). PCR products were phosphorylated with PNK (Thermo Fisher Scientific), ligated with T4 ligase (Thermo Fisher Scientific) and the template was removed via Dpn1 (Thermo Fisher Scientific) digestion. Ligated PCR products were transformed into *E. coli* (DH5α, New England Biolabs) and amplified plasmids were purified by PureLink miniprep (Invitrogen). The mutation primers (Merck – Custom Oligos) used for mutagenesis in this study are listed in Supplementary Table 5.

### β_1_AR constructs—pharmacology

The β_1_AR-E and β_1_AR-W constructs expressed using a pcDNA3.1 vector and used in pharmacological experiments (TRUPATH and ligand binding) were obtained as reported in Jones et al.^14^. Mutants (Val, Met, Leu, Ile, Phe, or Tyr) of these constructs were obtained for use in pharmacological assays by site directed mutagenesis from E130W^3.41^ using the primers listed in Supplementary Table 5.

The turkey β_1_AR BRET biosensor was based on the β_1_AR-E sequence with additions as shown in Supplementary Fig. 29a. The biosensor sequence was synthesised by Twist Bioscience (San Francisco, CA) and subcloned into pcDNA3.1. The construct sequence was checked for fidelity through Sanger Sequencing.

### β_1_AR expression and purification

β_1_AR E130X^3.41^ constructs were expressed and purified in lauryl maltose neopentyl glycol (LMNG) as previously described^14,26,27^. In brief, Spodoptera frugiperda (Sf9) cells (Thermofisher, 12659017) were transfected with pBacPAK8 plasmids containing the relevant β_1_AR construct and flashBac (Oxford Expression Technologies Ltd) expression vector using CellFectin II reagent (Gibco) to generate baculoviral particles. Sequential infections of increasing volume were carried out to amplify the baculovirus. Sf9 cells in mid-log phase were diluted to 3 × 10^6^ mL^−1^ and receptor was expressed by infection with viral stocks at a 1:200 dilution. ^13^C-methyl-methionine labelled receptor was expressed in ESF 921 ∆ methionine deficient media (Expression Systems) supplemented with penicillin (50 U mL^−1^), streptomycin (50 μg mL^−1^), and amphotericin B (50 μg mL^−1^). For ^13^C NMR, ^13^C-methyl-methionine was added at 0.25 g L^−1^ 4 h post baculoviral infection and cell density was diluted to 1.5 × 10^6^ mL^−1^. Unlabelled receptor was expressed at 1.5 × 10^6^ mL^−1^ in Insect-XPRESS with L-glutamine (Lonza) supplemented with 5% heat inactivated FBS (Sigma-Aldrich), and the above antibiotics. Infected cells were incubated at 27 °C with shaking for 48 h and harvested via centrifugation at 4000 g. Cells were washed once with phosphate buffered saline (PBS) and centrifuged before storage at −20 °C until receptor purification.

All purification steps were performed at 4 °C unless otherwise stated. Cells were thawed at RT and resuspended at 4 °C in solubilisation buffer (20 mM TrisHCl, pH 8.0, 350 mM NaCl, 3 mM imidazole, 1.5% LMNG, and 1x protease inhibitor cocktail (Roche)) for 1 h with constant stirring. Lysed cells were centrifuged at 75,000 g for 45 min. The supernatant was collected and sonicated (Fisherbrand 505) on ice at 25% amplitude for a total of 2 min, with pulse on and off times of 5 s. Clarified lysates were applied at 1 mL min^−1^ to a 5 mL NiNTA column (Cytiva) and washed in a buffer containing 20 mM TrisHCl, pH 8.0, 350 mM NaCl, 50 mM imidazole, and 0.02% LMNG. Receptor was eluted from the column in elution buffer (20 mM TrisHCl, pH 8.0, 350 mM NaCl, 250 mM imidazole, and 0.02% LMNG). The elution fractions were pooled and concentrated with a 50 kDa MWCO Amicon concentrator. Receptor was applied to a Superdex 200 Increase 10/300 column (Cytiva) pre-equilibrated in gel filtration buffer (20 mM TrisHCl, pH 8.0, 150 mM NaCl and 0.02% LMNG) and the resulting peak fractions were pooled and concentrated, generating receptor samples of >95% purity.

### ^19^F labelling

Receptors were purified by NiNTA affinity chromatography as above, and then buffer exchanged 10-fold into labelling buffer (20 mM TrisHCl, pH 8.0, 350 mM NaCl, and 0.02% LMNG). A282C^6.27^ was labelled with 3-bromo-1,1,1-trifluoroacetone (BTFA) by adjusting the receptor concentration to 20 μM and incubating the sample at 4 °C overnight in the presence of 200 μM BTFA (Sigma) and 50 μM glutathione disulfide (GSSG)^14^. Labelled samples were then buffer exchanged 10-fold into labelling buffer. C344^7.54^ was labelled with 2,2,2-trifluoroethanethiol (TET)^14,26^. The receptor was buffer exchanged into labelling buffer and concentrated to 10 μM before being incubated at 4 °C with 100 μM Aldrithiol-4™ (Sigma) and 50 μM GSSG for 10 min. The receptor was buffer exchanged 10-fold into labelling buffer, and the receptor was concentrated to 10 μM and incubated at 4 °C with 100 μM TET and 50 μM GSSG for 15 min. The labelled sample was buffer exchanged once more into labelling buffer to remove excess TET label. ^19^F labelled proteins were concentrated to 1 mL and applied to a Superdex 200 Increase 10/300 column running with gel filtration buffer (20 mM TrisHCl, pH 8.0, 150 mM NaCl, and 0.02% LMNG). The resulting peak fractions containing receptor were pooled and concentrated for NMR experiments.

### Mini-G expression and purification

The mini-G_s_ protein^56^ used here was mini-G_s_ 393 with an additional C96A mutation made to reduce disulfide mediated dimerisation^14^. Mini-G_s_ was expressed and purified as previously described^14^ in BL21-RIL cells (New England Biolabs). Starter cultures were grown overnight in LB supplemented with ampicillin (50 μg mL^−1^) and used to inoculate 500 mL of LB supplemented with ampicillin and 10% glycerol in 2 L baffled flasks. Cultures were incubated at 37 °C with shaking at 220 rpm until an OD_600_ of 0.6. Expression was induced with 1 mM IPTG, and the culture was incubated at 25 °C overnight with shaking. Cells were harvested by centrifugation at 4000 g for 15 min. Cell pellets were resuspended in PBS and centrifuged before short term storage at −20 °C. All purification steps were performed at 4 °C unless otherwise stated. Cell pellets were thawed and resuspended into lysis buffer (40 mM TrisHCl, pH 7.4, 100 mM NaCl, 10 mM imidazole, 10% glycerol, 5 mM MgCl_2_, 50 μM GDP, 1 mM PMSF, 100 μM DDT, 50 μg mL^−1^ lysozyme, and 1 × Roche protease inhibitor cocktail). The cells were lysed with 3 passes through an EmulsiFlex-C5 (Avestin) operating at ~500 bar. Lysates were clarified at 75,000 g for 30 min and the supernatant was sonicated (Fisherbrand 505) on ice at 40% amplitude for a total of 2 min, pulsing with on and off times of 5 s. The lysate was passed over a 5 mL NiNTA column at 3 mL min^−1^. The column was washed in 40 mM TrisHCl, pH 7.4, 100 mM NaCl, 10 mM imidazole, 10% glycerol, 5 mM MgCl_2_, and 50 μM GDP, and then washed in 20 mM TrisHCl, pH 7.4, 500 mM NaCl, 40 mM imidazole, 10% glycerol, 1 mM MgCl_2_, and 50 μM GDP. The mini-G_s_ was eluted in 20 mM TrisHCl, pH 7.4, 100 mM NaCl, 500 mM imidazole, 10% glycerol, 1 mM MgCl_2_, and 50 μM GDP. Peak fractions were pooled and supplemented with 5 mM TCEP and His tagged tobacco etch virus protease (TEV, made in-house) at a 1:30 (mg TEV:mg mini-G_s_) dilution. The sample was placed in a 3.5 kDa cutoff dialysis membrane (Spectrum Labs) and dialysed against 1 L of dialysis buffer (20 mM Tris pH 7.4, 100 mM NaCl, 10% glycerol, 1 mM MgCl_2_, 10 μM GDP) for 20 h, and then dialysed for 4 h in 1 L of fresh dialysis buffer. The sample was supplemented with 20 mM imidazole and passed over a gravity flow column packed with 5 mL of NiNTA resin (QIAGEN). The column was washed with 5 mL of dialysis buffer plus 20 mM imidazole, and the flow-through was collected. The mini-G protein was concentrated to 2.5 mL using a 10 kDa MWCO concentrator and purified by size exclusion chromatography on a HiPrep 16/60 sephacryl S200 HR column (Cytiva) running in 20 mM TrisHCl, pH 8.0, 150 mM NaCl, 1 mM MgCl_2_, and 1 μM GDP. Peak fractions containing mini-G_s_ were pooled and concentrated to ~1.7 mM.

### NMR experiments

NMR samples containing ^13^C-methyl-methionine labelled or ^19^F labelled β_1_AR at 20–60 μM in 20 mM TrisHCl, pH 8.0, 150 mM NaCl and 0.02% LMNG were supplemented with 5% D_2_O and prepared in 5 mm Shigemi tubes. Receptor was prepared in the apo form, with 1 mM isoprenaline (Sigma), or 1 mM propranolol (Sigma) to saturate in the ligand-bound receptor population and ensure changes to NMR spectra reflected conformational exchange of the ligand-bound states. Sodium ascorbate (2 mM) was added to decrease ligand oxidation rates. Unless otherwise stated, apo spectra were recorded at 298 K due to the low stability of β_1_AR-E in the absence of orthosteric ligand. Mini-G_s_ was added at two molar equivalents with 15 mM MgCl_2_. The negative allosteric modulator AS408 (Biosynth Ltd) was dissolved in deuterated DMSO to 100 mM, see figures for concentrations used. Samples were incubated with AS408 for 20 min prior to NMR experiments.

^1^H-^13^C 2D HMQC NMR experiments were performed on a Bruker Avance III 800 spectrometer (^1^H 800.13 MHz) running TopSpin 3.1 and equipped with a 5 mm TXI HCN/z cryoprobe. NMR spectra were recorded at 308 K or 298 K using the XL-ALSOFAST sequence^69^ with experiments typically run with 64 scans (~2 h), using 100 increment pairs in the indirect dimension (t_1H_ 50 ms, t_13C_ 25 ms) and with a spectral width of 10,000 Hz (offset: 3750 Hz) in the direct dimension and 4000 Hz (offset: 3420.27 Hz) in the indirect dimension. Experiments recorded for β_1_AR-E I168M^4.52^ were recorded using the above pulse programme at 308 K or 298 K using a Bruker Avance III 600 spectrometer (^1^H 600.13 MHz) running TopSpin 3.1 and equipped with a 5 mm QCI HFCN/z cryoprobe. Experiments were typically run with 64 scans (~1.5 h) using 76 increment pairs in the indirect dimension (t_1H_ 50 ms, t_13C_ 25 ms) and with a spectral width of 10,000 Hz (offset: 2818 Hz) in the direct dimension and 3012 Hz (offset: 2565.34 Hz) in the indirect dimension.

Multiple experiments were co-added to improve the signal-to-noise ratio. Data were processed in Azara v2.8 (W. Boucher) and analysed using CCPN Analysis V2^70^.

^19^F 1D NMR spectra were recorded at 298 K using a Bruker Avance III 600 spectrometer running TopSpin 3.1 and equipped with a 5 mm QCI HFCN/z cryoprobe (^19^F 564.64 MHz). ^19^F chemical shifts were referenced against an internal standard of 2 μM trifluoroacetic acid (TFA) at −75.22 ppm. Pulse acquire experiments were run recording 2560 complex points (50 ms) with a spectral width of 50,000 Hz (offset: −50,821.80 Hz) and consisting of 5000 to 25,000 scans with a recycling time of 1 s, which lasted between 1 and 6 h. Spectra were recorded at 298 K to increase the chemical shift difference between pre-active and active states. Multiple experiments were co-added to improve the signal-to-noise ratio. All 1D FIDs were apodized with 20 Hz line broadening and zero-filled to 64k points prior to FFT using Topspin 4.4.1. All signals were deconvoluted as Lorentzian lines for the measurement of linewidths at half height and the comparison of *R*_2_ values. Overlapping signals were deconvoluted using in-house written software^14,26^. Fitted curves produced residuals that deviated from the baseline no more than the expected noise level.

### In cell ligand affinity measurements

HEK293T cells (ATCC, CRL-3216) were transiently transfected with the relevant FLAG-Nluc-β_1_AR construct. Cells were grown overnight, harvested and seeded at 10,000 cells per well onto white 96-well plates (Greiner Bio-One). Media was removed after 24 h before addition of 90 μl PBS containing 0.49 mM MgCl_2_·6H_2_O, 0.9 mM CaCl_2_·2H_2_O, 0.1% BSA and 1:20,000 Nano-Glo® Substrate (Promega). The plate was incubated for 10 min at room temperature in the dark. Cells were stimulated with varying concentrations of (S)-propranolol-red (CA200689, Hello Bio), from 0.1 nM to 300 nM, in the absence or presence of 10 μM unlabelled propranolol, and plates were read immediately using a PHERAstar microplate reader (BMG Labtech, software version 1.60 R4, firmware version 1.33) with a NanoBRET filter module. BRET ratios (Em. *λ* > 610 nm/Em. *λ* 460 nm) corrected to vehicle-treated cells were used for subsequent analysis. Specific binding of each concentration of (S)-propranolol-red was determined by subtracting the vehicle-corrected, ΔBRET values for non-specific binding (with 10 µM propranolol) from total binding. Kinetic data were fit using the ‘Association kinetics – Two or more conc. of hot’ model in GraphPad Prism 10.5 to determine *k*_on_ and *k*_off_ values. Equilibrium specific binding data, using the BRET ratio at ten minutes, were fitted to the one-site specific binding model in GraphPad Prism 10.5 to calculate the *K*_D_ for (S)-propranolol-red at each FLAG-Nluc-β_1_AR E130X^3.41^ mutant. Differences between (S)-propranolol-red binding parameters were assessed using ordinary one-way ANOVA, using Dunnett’s post-hoc test for multiple comparisons and presented as described in Curtis et al.^71^.

The p*K*_i_ for isoprenaline at each FLAG-Nluc-β_1_AR E130X^3.41^ construct was determined by transfecting cells and seeding as described above. After 10 min incubation of cells in 90 μl PBS containing 0.49 mM MgCl_2_·6H_2_O, 0.9 mM CaCl_2_·2H_2_O, 0.1% BSA and 1:20,000 Nano-Glo® Substrate, cells were stimulated with varying concentrations of isoprenaline, from 10 nM to 1 mM, in the presence of 50 nM (S)-propranolol-red. Emission was measured using the NanoBRET module of a PHERAstar microplate reader, and the BRET ratio (Em. *λ* > 610 nm/Em. *λ* 460 nm) corrected to cells treated with 100 μM isoprenaline. Kinetic ΔBRET values were fit using the ‘Kinetics of competitive binding’model in GraphPad Prism 10.5, to determine *k*_on_ and *k*_off_ values, using the mean values for CA200689. Equilibrium affinity (p*K*_i_) values were determined using the ‘One site – fit K_i_’ model and are represented as mean of three separate experiments ± SEM. Differences between isoprenaline affinity parameters were assessed using ordinary one-way ANOVA, using Dunnett’s post-hoc test for multiple comparisons and presented as described in Curtis et al.^71^.

For assays involving AS408, cells were pre-incubated with 0, 5, 10, 25, 50 or 100 µM AS408 alongside NanoGlo® for 15 minutes before the addition of 60 nM (S)-propranolol-red and isoprenaline. The kinetic parameters *k*_on_ and *k*_off_ were calculated for isoprenaline in the presence of each AS408 concentration based on the corresponding values for (S)-propranolol-red. Note, minor compound interference was observed at high concentrations of AS408 (50-100 µM).

### TRUPATH assays

HEK293T cells were transiently transfected with the relevant β_1_AR E130X^3.41^ construct, each Gα-Rluc8, and appropriate Gβ and Gγ^55^, at a ratio of 1:1:1:1. After 24 h, cells were harvested and seeded onto white 96-well plates (Greiner Bio-One) at a density of 50,000 cells per well. Media was removed after 24 h, and cells washed with HBSS before addition of 90 μl HBSS containing 20 mM HEPES, 0.1% BSA, adjusted to pH 7.4, and 11.1 μM coelenterazine 400a (Nanolight Technology, USA). The plate was then incubated for 10 min at room temperature in the dark. Cells were stimulated with varying concentrations of isoprenaline, from 10 pM to 10 μM, and emission measured at 400 nm and 515 nm for 20 min, every 60 s. Emission measurements were made using BMG Labtech PHERAstar (software version 1.60 R4, firmware version 1.33). The BRET ratio (Em. *λ* 515 nm/Em. *λ* 460 nm) was corrected to vehicle-treated cells and the area under the resulting curve used to produce the concentration-response curves shown. Concentration-response curves were fitted to the 3-parameter logistic equation to determine E_max_ and pEC_50_ values for isoprenaline at each β_1_AR E130X^3.41^ mutant.

For assays involving AS408, cells were pre-incubated with this compound alongside coelentrazine 400a for 15 minutes before the addition of isoprenaline. To account for changes in basal activity of β_1_AR, the baselines subtracted from BRET ratios were obtained from cells transfected with pcDNA3.1 containing no receptor alongside TRUPATH constructs that were subsequently treated identically to those expressing β_1_AR.

### Conformational Biosensor assay

HEK293A (a gift from Professor David Yule, Thermofisher, R70507) cells stably expressing the β_1_AR-E BRET biosensor were seeded at 30,000 cells per well in white 96-well plates (Greiner Bio-One) and grown for 24 h at 37 °C, 5% CO_2_ in humidified conditions. Assays for orthosteric ligand activity at the β_1_AR-E BRET biosensor were carried out in PBS containing 0.1% BSA, 0.5 mM MgCl_2_ and 0.9 mM CaCl_2_. Cells were incubated with AS408 and NanoGlo® substrate (1:20,000) for 15 minutes at room temperature and in the dark prior to the addition of orthosteric ligands. Immediately after orthosteric ligand addition, Nano luciferase luminescence (Em. *λ* 410-480nm) and citrine fluorescence (Em. *λ* 530-540nm) were measured every 150 seconds on a CLARIOstar Plus® microplate reader (BMG Labtech, UK, SMART control version 6.20, Mars version 4.20). Data were analysed in GraphPad Prism 10.5 by calculating the BRET ratio (fluorescence/luminescence) for each well and timepoint, then finding the area between each curve and the baseline (AUC), as demonstrated by treatment with vehicle (Supplementary Fig. 29b). The resulting plot of AUC over logarithmic isoprenaline concentration was fitted to the 3-parameter logistic equations and represented as the mean of *n* ≥ 3 independent biological replicates ± SEM. In assays including AS408, minor compound interference was observed at high concentrations of AS408 (50-100 µM).

For confocal microscopy, HEK293A cells transiently expressing the β_1_AR-E BRET biosensor were plated onto a poly-D-lysine-coated coverslip and mounted onto an “Attofluor” holder (Molecular Probes, Leiden, The Netherlands). The cellular location of the labelled receptors was monitored by live cell confocal microscopy performed on a Zeiss LSM 800 meta system (Carl Zeiss, Oberkchen, Germany). Zeiss Zen Blue 2 software (2.1) was used for data acquisition and analysis. Images were taken with an oil-immersion 63x lens using the factory settings for YFP.

### Operational model of allostery

Cooperativity and modulator binding affinities were obtained by fitting data from the TRUPATH BRET2 G_s_ assay (Fig. 3a) and β_1_AR-E BRET-sensor (Fig. 6c) isoprenaline concentration response curves (10, 25, 50, and 100 µM AS408 and 10, 25, 50 µM AS408, respectively) using the complete operational model of allostery^58^ in GraphPad Prism 10.6.1 (GraphPad, San Diego, CA). The approach was modelled on assessment of cooperativity for AS408 at the hβ_2_AR^29^. Maximal and basal responses were fixed, according to the specific assays. The Hill coefficient was set to 1.0 and τ_B_-values were fixed at near zero with the assumption that AS408 is a NAM for agonists. This enabled values for *K*_A_, *K*_B_, *α*, and *β* to be estimated. For calculation of log (*αβ*), where $\log (\alpha \beta )=\log \alpha+\log \beta$, uncertainties were propagated according to the Gaussian law of propagation of uncertainty:

1 $${\sigma }_{\log (\alpha \beta )}=\sqrt{{\sigma }_{\log \alpha }^{2}+{\sigma }_{\log \beta }^{2}}$$

Assuming independence of the parameter estimates.

### Flow cytometry

HEK293T cells were transfected with FLAG-Nluc-β_1_AR E130X^3.41^ constructs for 48 hours prior to measurement of surface expression. 500,000 cells (with viability > 85%) were harvested and washed twice with PBS supplemented with 1% BSA and 0.03% sodium azide (FACS buffer). Cells were stained in FACS buffer (APC anti-FLAG tag antibody (BioLegend, cat no. 637307) at 1 in 100 dilution) for 30 minutes at room temperature. Cells were washed and resuspended in 100 μL FACS buffer. For fixation, 3 volumes of ice cold 80% acetone were added and cells kept for 1 hour at 4 °C^72^. Cells were washed twice with FACS buffer and resuspended in 300 μL. Fluorescence was measured using an BD LSRFortessa™ Cell Analyser (Waters Biosciences), gating on single cells using BD FACSDiva™ Software v9.0. Geometric mean fluorescence intensity was calculated using OMIQ software, and normalised to the E130^3.41^ variant. Statistical significance was determined using a Kruskal-Wallis test for non-parametric data.

### Biolayer interferometry (BLI) measurements

Binding affinity assays were conducted as previously described^14^. Briefly, β_1_AR E130X^3.41^ proteins were immobilised to streptavidin (SA) coated bio-sensor tips (Octet) through an N-terminal avi-tag on the receptor. Purified avi-tagged receptors were diluted to ~40 μM. The avi-tag was labelled with biotin by 20 h incubation at 4 °C with 10 mM ATP, 10 mM Mg acetate, 50 μM biotin and 1-2 μM GST-BirA (prepared in house). Receptors were repurified and buffer exchanged via SEC using a Superdex 200 Increase 10/300 column running in 20 mM TrisHCl, pH 8.0, 150 mM NaCl, and 0.02% LMNG. BLI binding assays were performed in BLI buffer (20 mM TrisHCl, pH 8.0, 150 mM NaCl, 15 mM MgCl_2_, 0.02% LMNG, and 0.1% BSA) and assay mixtures were prepared in black 96-well plates using sample volumes of 200 μL. Experiments were run on an 8-channel Octet® R8 instrument (Sartorius) using Data Acquisition V11.0.0.64 (Pall ForteBio LLC), at 25 °C shaking at 400 rpm. SA coated biosensors were pre-hydrated in BLI buffer for a minimum of 10 min before experiments. Baseline signals were measured in BLI buffer for 120 s before the receptor (300 nM) was loaded onto the SA tips with an association time of 600 s. Non-specific binding was removed by dissociation into BLI buffer for 600 s. Receptor-loaded tips were transferred to BLI buffer containing orthosteric ligand (500 μM isoprenaline or propranolol, and 1 mM Na ascorbate) for 300 s. Association steps into BLI buffer plus agonist and varying binding partner concentrations were 600 s. Dissociation steps back in BLI buffer plus agonist were 600–900 s.

Dose-response experiments for mini-G_s_ binding affinities to E130X^3.41^ mutants were recorded using a mini-G_s_ dilution series from 31 nM to 500 nM, using individual biosensors for each concentration and referencing to a ligand only biosensor (0 nM mini-G_s_) with immobilised receptor (Supplementary Fig. 7b). Single point measurements were conducted for β_1_AR-Y, run in triplicate using *n* = 3 separate tips and a reference channel with loading steps using un-biotinylated avi-tagged receptor were used to subtract nonspecific binding.

Experiments with AS408 used agonist and NAM association steps of 1200 s to ensure ligand association reached equilibrium. All experiments were conducted with 1 mM sodium ascorbate, including apo experiments. Concentration dependence data were obtained with increasing AS408 concentrations (range of 0–100 µM AS408, see experiments for specific concentrations of AS408 used) utilising dose-response experiments with a concentration series of 31.25 nM to 500 nM mini-G_s_. Experiments used a constant corrected DMSO concentration of 0.1%, including 0 µM AS408. Experiments were referenced against a ligand and NAM only biosensor (0 nM mini-G_s_) with immobilised receptor.

BLI data was analysed on Data Analysis V11.0.0.4 (Pall ForteBio LLC) using reference channel subtraction and Savitzsky–Golay filtering. For dose-response experiments, responses were used to calculate *K*_D_, *k*_on_, and *k*_off_ values using a 1:1 global fitting with *R*_max_ unlinked by sensors. Single point measurements were analysed assuming monophasic 1:1 association and dissociation curves with local partial fitting. Concentration dependence plots for AS408 were produced using GraphPad Prism 10.5. IC_50_ values were calculated from non-linear regression curves using a Variable slope (3-parameter) fit and with Hill constants set to −1. See Jones et al.^14^, for additional information on BLI assays and control experiments.

### MD simulations

The isoprenaline and G_s_ protein-bound β_1_AR ternary complex was obtained from the Protein Data Bank (PDB ID: 7JJO)^57^. The G_s_ protein and nanobody Nb35 were removed, and Modeller^73^ was used to replace missing residues and introduce mutations according to the β_1_AR-E construct (Supplementary Fig. 1a), excluding any N-terminal tags or the C-terminal octa-His tag. The structure was mutated at E130^3.41^ via the rotamers tool in ChimeraX^74^ to generate the E130X^3.41^ variants and aligned to the orientation of the reference 7JJO structure from the Orientations of proteins in the membrane (OPM) database^75^. The receptor structures were processed using CHARMM-GUI^76^. The N- and C-termini were capped with acetyl (ACE) and amidated (CT2) patches, respectively. Hydrogen atoms were added, and amino acids were kept in their standard protonation state at pH of 7.0 with the exception of D87^2.50^, E130^3.41^, and D138^3.49^ being protonated in agreement with previous studies stating their importance in the active state^77^. The disulphide bonds (C114-C199, C192-C198) were maintained. Isoprenaline was maintained in the orthosteric pocket. In simulations with AS408, the initial AS408 pose was obtained by aligning the crystal structure of the ligand with hβ_2_AR (PDB ID: 6OBA)^29^ to the curated β_1_AR-E structure. Ligand parameterisation and force field topology were obtained via CGenFF and ParamChem through CHARMM-GUI^78^. The protein model was embedded in an in-vivo mimetic membrane^79^ (Supplementary Table 6). The embedded protein was inserted into a ~ 95 Å (*x* and *y*) rectangular simulation box with a height of ~140 Å (*z*), which was then filled with ~26,000 TIP3P^80^ water molecules. NaCl ions were added to neutralise the charge and achieve a final salt concentration of 150 mM. Simulation boxes contained ~110,000 atoms overall.

Each system was energy-minimised with the steepest descent algorithm. The system was equilibrated in six steps, gradually reducing restraints on the protein side chains, backbone, and lipid molecules, following a protocol similar to the default procedure used by the CHARMM-GUI Membrane Builder (Supplementary Table 7) for a total equilibration time of 50 ns. The first two equilibration steps were performed in the NVT ensemble with a time step of 1 fs while the third step was performed in the NPT ensemble with a 1 fs time step. The remaining steps and production run were continued in the NPT ensemble with a 2 fs time step. Bonds involving hydrogen were constrained with the LINCS algorithm^81^. A Verlet cutoff scheme was used with a neighbour list update frequency of 20 steps and a list cutoff of 1.2 nm. Van der Waals interactions were treated with a cutoff scheme using a force-switch modifier with a switch distance of 1.0 nm and a cutoff of 1.2 nm. Electrostatic interactions were calculated using the particle mesh Ewald (PME) method with a real-space cutoff of 1.2 nm. The temperature was maintained at 310.15 K using the v-rescale thermostat with a coupling time constant of 1.0 ps, applied independently to the solute, membrane, and solvent groups. In the NPT ensemble, pressure was maintained at 1.0 bar using the C-rescale barostat with semi-isotropic pressure coupling, a time constant of 5.0 ps, and a compressibility of 4.5 × 10⁻⁵ bar⁻¹. All systems were simulated using GROMACS version 2024.6^82^ and the CHARMM36/CGenFF force field combination^78,83^. Production runs were conducted for 1000 ns. Three independent MD simulation replicates were run, together generating 3 µs data for each system (27 µs of data in total). Analysis of MD trajectories was performed with MDAnalysis v2.10.0^84^, ProLIF v2.1.0^85^ was used to extract molecular interactions, *mdciao* v1.0.0^86^ was used to generate the flareplots, and molecular visualisation was performed using ChimeraX v1.10.1^74^.

### Reporting summary

Further information on research design is available in the Nature Portfolio Reporting Summary linked to this article.

## Supplementary information

Supplementary Information

Reporting Summary

Transparent Peer Review file

## Source data

Source data

## Data Availability

The NMR, BLI, in cell and TRUPATH ligand binding data generated in this study have been deposited in a Figshare repository and can be downloaded from the permanent link: https://figshare.com/projects/Data_sets_for_-_Uncoupling_of_the_Allosteric_Signalling_Network_in_the_sub_1_sub_-adrenergic_receptor_by_a_Negative_Allosteric_Modulator/267181 or by contacting the authors. The PDB accession codes for the structures used in this study were 2Y03, 7JJO, and 6OBA. Molecular dynamics trajectory files and interaction data can be found from the permanent link: https://doi.org/10.5281/zenodo.19371846. Source data are provided as a Source Data file. Source data are provided with this paper.

## Code availability

The in-house written software (R.W. Broadhurst, T. H. Harman, unpublished) used for deconvolution of ^19^F NMR spectra was custom-made and is available upon request by contacting the authors. The NMR processing package Azara v2.8 can be obtained through the link https://cambridge2000.com/azara/ where the software is available under licence directly from Dr W. Boucher. The scripts used to run the MD simulations are available at https://doi.org/10.5281/zenodo.19371846
