# Supplementary material for: Uncoupling of the allosteric signalling network in the β1-adrenergic receptor by a negative allosteric modulator
Source: Nat Commun. 2026 Sep 18;17:10952. doi: 10.1038/s41467-026-77802-8 (PMC13680373; doi:10.1038/s41467-026-77802-8)
Supplement: Supplementary file 1 [file 41467_2026_77802_MOESM1_ESM.pdf]

Supplementary Information

Uncoupling of the Allosteric Signalling Network in the β_1-adrenergic receptor by a Negative Allosteric Modulator

Thomas H. Harman[1], Timothy Noel[2], Ali Nokhbehzaim[2], Abigail Pearce[2], Andrew J. Y. Jones[1], Anjana Saji[2], Matthew Harris[2], Venkat S. Vege[2], Aneesh Chandran[2], Pierre Leroy[1], Suleiman Al-Sabah[3], Graham Ladds[2,*], Daniel Nietlispach[1,*].

[1]Department of Biochemistry, University of Cambridge, 80 Tennis Court Road, Cambridge CB2 1GA, UK.
[2]Department of Pharmacology, University of Cambridge, Tennis Court Road, Cambridge CB2 1PD, UK.
[3]Department of Pharmacology & Toxicology, Faculty of Medicine, Kuwait University, Kuwait City 13060, Kuwait.

*To whom correspondence may be addressed: dn206@cam.ac.uk, grl30@cam.ac.uk.

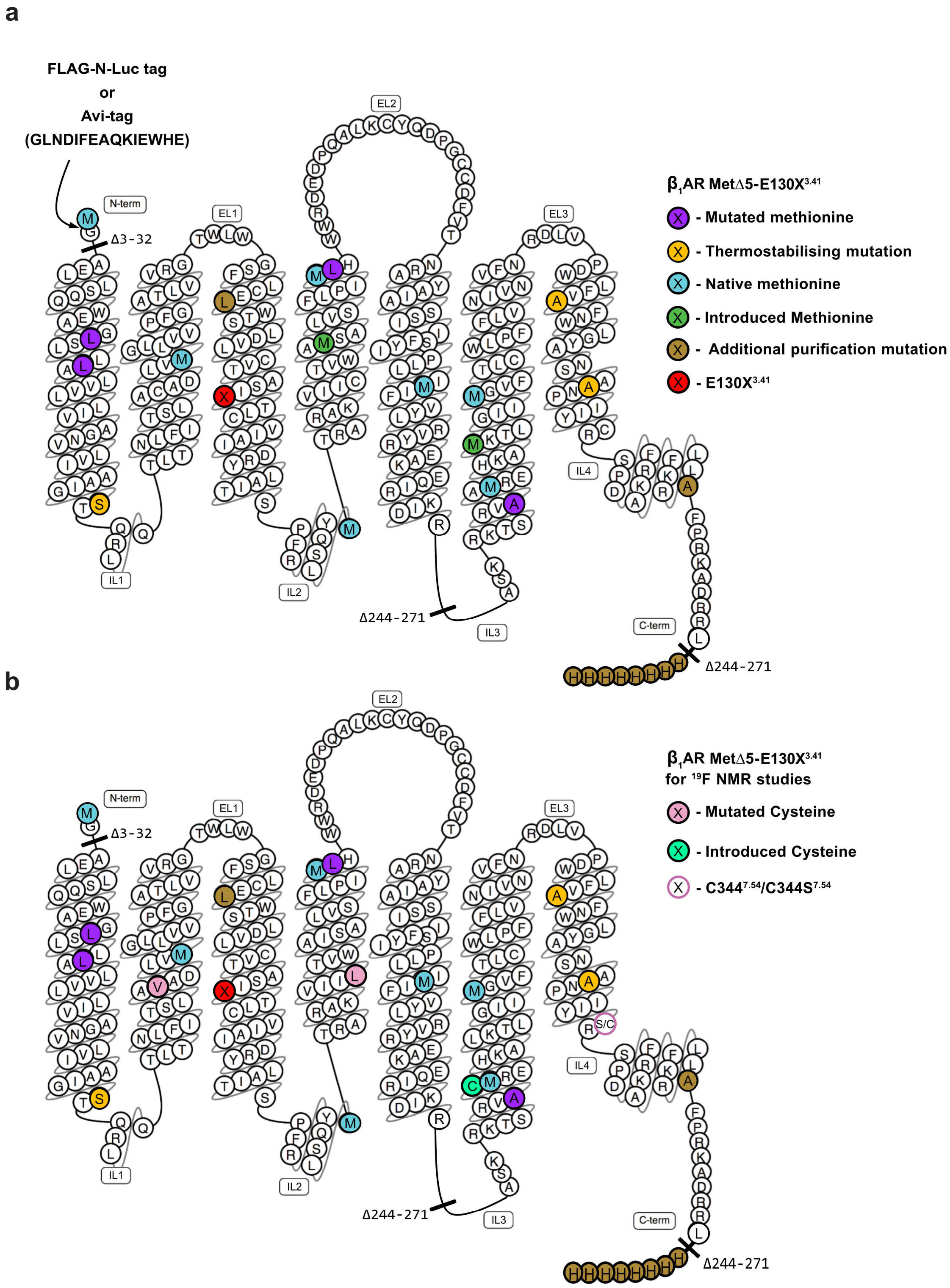

Supplementary Figure 1. Snake diagrams of turkey β$_1$AR constructs. a The β$_1$AR construct used in NanoBRET ligand binding, TRUPATH, BLI, and ^{1}H,^{13}C NMR experiments. Intracellular loop (IL) and extracellular loop (EL) regions are indicated. All mutations are relative to the native turkey (*Meleagris gallopavo*) β$_1$AR sequence (UniProt accession code P07700), see methods for explanations. Truncations to the N-terminus, C-terminus, and IL3 are shown with solid black bars. Additional mutations include mutated methionine residues (purple, MetΔ5[1]), thermostabilising mutations (orange), and purification

mutations (brown). Native methionine probes are shown in blue. Introduced methionine mutations for NMR experiments are shown in green. In cell ligand affinity studies used receptors with N-terminal FLAG-N-Luc sequence between residues M1 and G2. BLI studies used constructs with an additional N-terminal avi-tag (GLNDIFEAQKIEWHE) between residues M1 and G2. The mutated E130X$^{3.41}$ position is shown red. **b** The β_1AR construct used in ^{19}F NMR experiments. The constructs were the same as those used in (**a**) except for: TM7 labelling (^{TET}C344$^{7.54}$) used the mutated cysteines (pink, ΔC2[2]); TM6 labelling (A282C$^{BTFA,6.27}$) used the mutated cysteines plus mutated C344S$^{7.54}$ (pink ring, ΔC3[2]) and introduced cysteine A282C (light green). See methods for details.

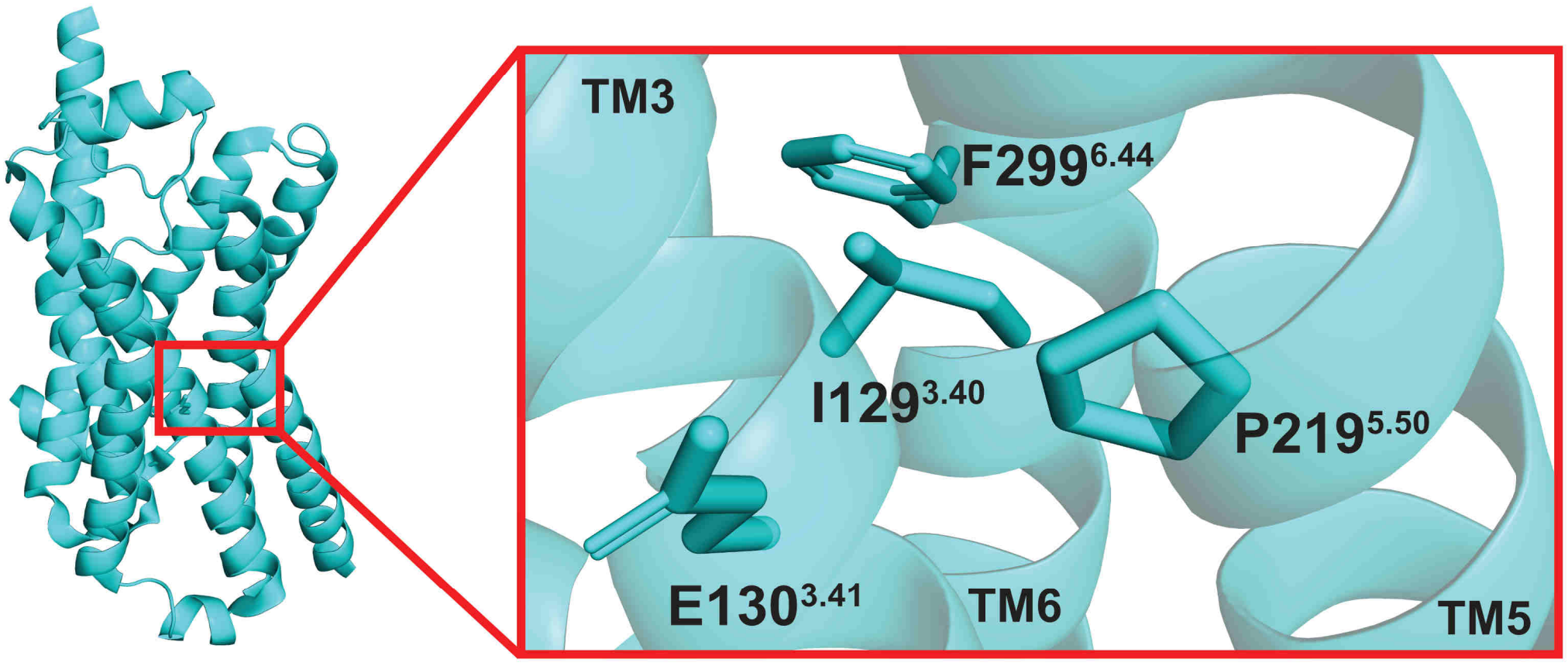

Supplementary Figure 2. The position of the E130$^{3.41}$ residue relative to the PIF motif in β_1AR. Structure overview of the isoprenaline-bound β_1AR (blue, PDB ID: 2Y03)[3] with an enlargement of the region highlighting the proximity of the P$^{5.50}$-I$^{3.40}$-F$^{6.44}$ motif side chains and the E130$^{3.41}$ position. The location of E130$^{3.41}$ is adjacent to the I129$^{3.40}$ and P219$^{5.50}$ side chains. The PIF motif is displayed in its inactive conformation.

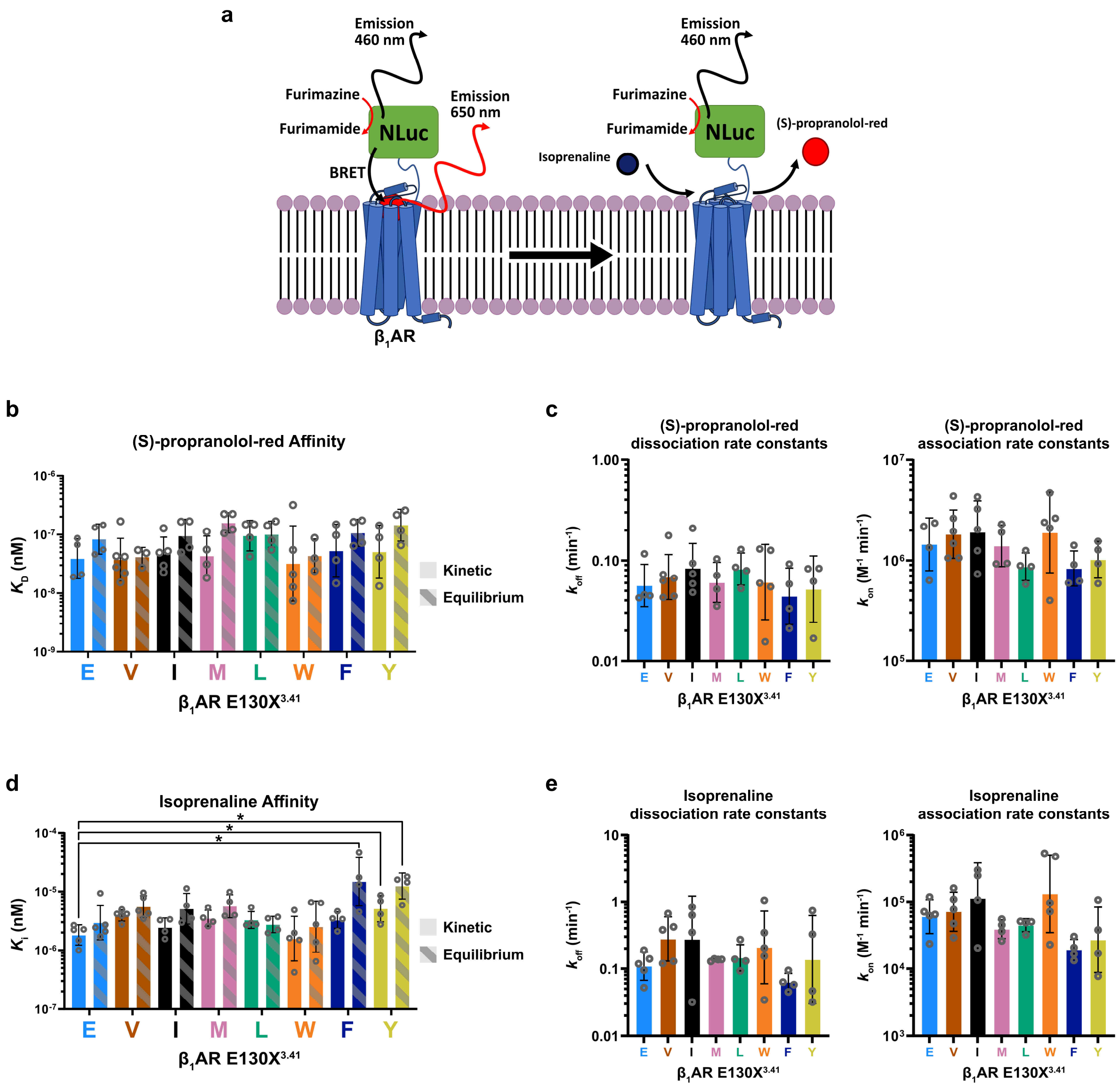

Supplementary Figure 3. The effects of E130X$^{3.41}$ mutations on ligand binding affinities to β_1AR. **a** A scheme for the luminescence-based ligand binding assay for the determination of ligand K_i to β_1AR. N-Luciferase (N-Luc) tagged β_1AR binds to the β_1AR antagonist (S)-propranolol-red[4]. Catalysis of furimazine to furimamide by NLuc generates luminescence which is transferred by BRET to (S)-propranolol-red and emitted at 650 nm. Titration with the non-BRET active ligand displaces (S)-propranolol-red and reduces the BRET signal. The NanoBRET signal loss observed upon titration of isoprenaline in competition with (S)-propanolol-red facilitates binding curve generation and K_i calculations[5]. Figure adapted with permission from Jones *et al.,* 2024[6]. **b** The K_D values for (S)-propranolol-red binding to β_1AR E130X$^{3.41}$ variants, measured by the nanoBRET signal between the bound (S)-propranolol-red and receptor N-terminally conjugated with nanoluc expressed in HEK293T cells. Data are the mean ± SEM of *n*=4 (E,M,L,F,Y) or *n*=5 (V,I,W) independent biological experiments, performed in duplicate. **c** The dissociation and association rate constants of (S)-propranolol-red binding to β_1AR E130X$^{3.41}$ variants in HEK293T cells. Data are the mean ± SEM of *n*=4 (E,M,L,F,Y) or *n*=5 (V,I,W) independent biological experiments, performed in duplicate. **d** The K_i values for isoprenaline binding to β_1AR E130X$^{3.41}$ variants, measured in a competition assay using the nanoBRET signal loss upon titration of isoprenaline against bound (S)-propranolol-red. β_1AR E130X$^{3.41}$ variants were expressed in HEK293T cells and data are the mean ± SEM of *n*=4 (M,I,L,F,Y) or *n*=5 (E,V,W) independent biological experiments, each performed in duplicate. * indicates significance

(p<0.05). **e** The dissociation and association rate constants of isoprenaline binding to β_1AR E130X$^{3.41}$ variants in HEK293T cells. Data are the mean ± SEM of n=4 (M,I,L,F,Y) or n=5 (E,W,V) independent biological experiments, performed in duplicate. The graphs in (**b-e**) were analysed for significance, using log-transformed data and by one-way ANOVA with Dunnet's post-hoc tests. No significant differences were observed in (**b**), (**c**), and (**e**) (p>0.05). In (**d**), significance was observed in comparisons of β_1AR-E with β_1AR-Y for both kinetic and equilibrium K_i values (kinetic: p = 0.0176; equilibrium: p = 0.0195) and β_1AR-E with β_1AR-F for the equilibrium K_i (p = 0.0074).

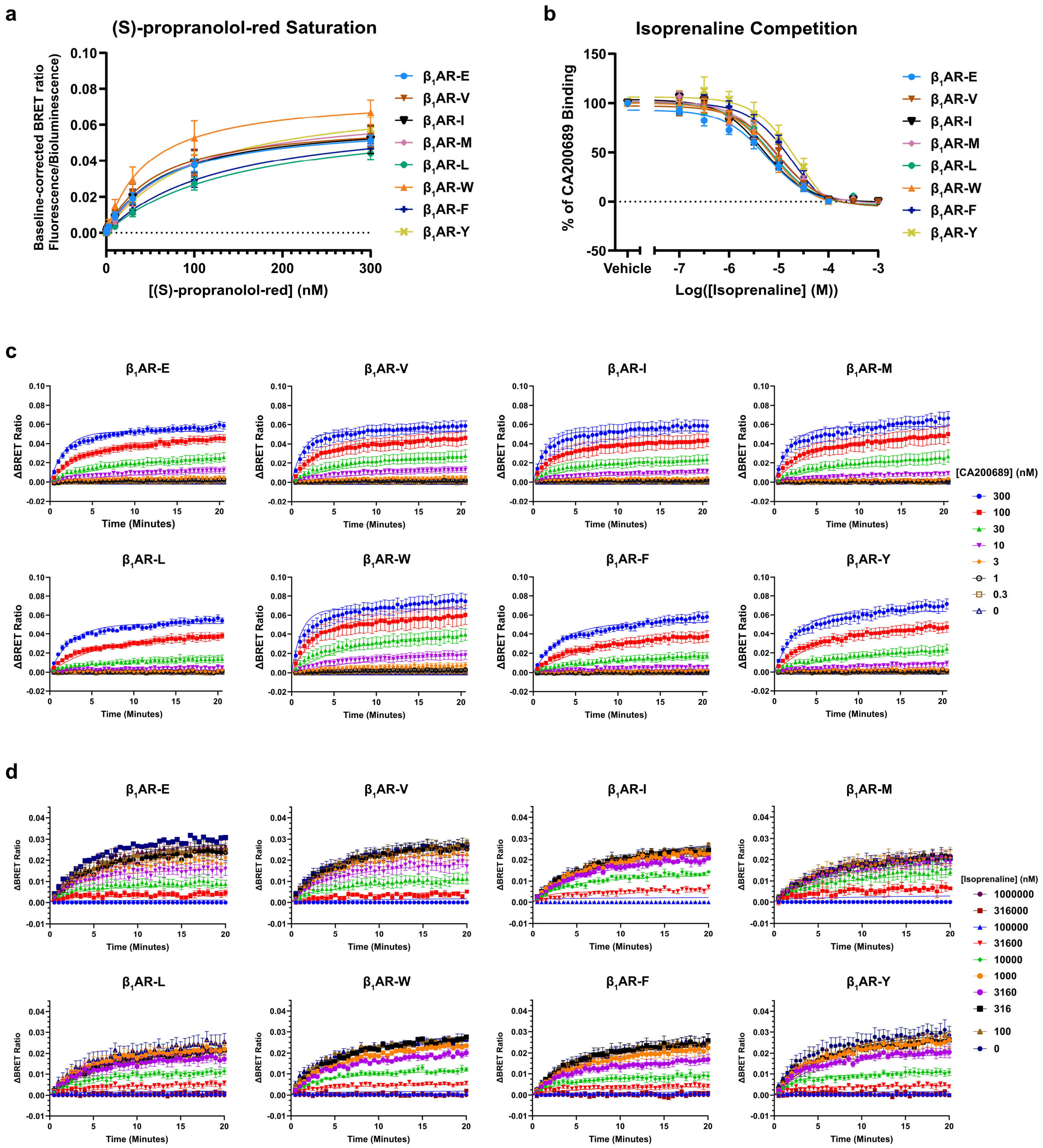

Supplementary Figure 4. Measurements of ligand binding kinetics to β$_1$AR E130X$^{3.41}$ variants. **a** Saturation equilibrium curves for binding of the fluorescently labelled β$_1$AR antagonist (S)-propranolol-red (CA200689) to β$_1$AR E130X$^{3.41}$ variants in HEK293T cells, measured using the nanoBRET-based assay shown in Supplementary Fig. 3a. Data are the mean ± SEM of *n*=4-6 independent biological replicates. **b** Competition equilibrium curves for isoprenaline affinities to β$_1$AR E130X$^{3.41}$ variants in HEK293T cells, measured using the nanoBRET-based assay. Competition assays were recorded with varying isoprenaline concentrations against bound (S)-propranolol-red. Data are mean ± SEM of *n*=4-6 independent biological experiments. **c** Kinetic traces for the saturation equilibrium measurements of (S)-propranolol-red binding to β$_1$AR E130X$^{3.41}$ variants shown in (**a**). **d** Kinetic traces for the competition equilibrium measurements of isoprenaline binding to β$_1$AR E130X$^{3.41}$ variants shown in (**b**).

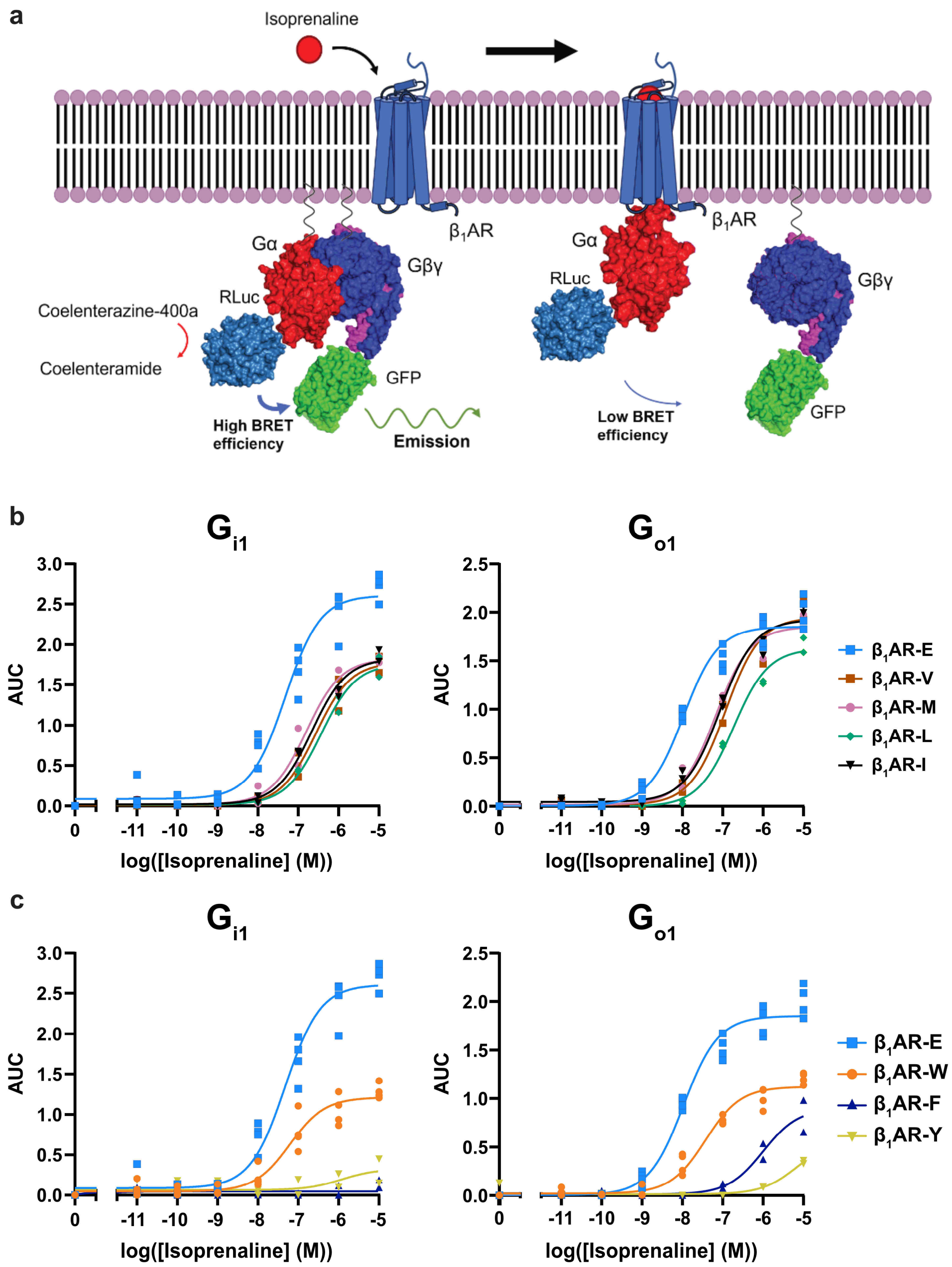

Supplementary Figure 5. E130X$^{3.41}$ mutations reduce $G_{i/o}$ protein coupling to agonist-bound β_1AR. **a** A scheme of the TRUPATH assay[7]. Gα and Gβγ proteins are labelled with R-Luc8 and GFP2, respectively. Binding of ligand to receptor induces receptor activation and G protein binding. Dissociation of the Gα and Gβγ subunits reduces the BRET efficiency, therefore reducing emission intensity. Titration of ligand facilitates generation of EC_{50} curves. Figure adapted with permission from Jones et al., 2024[6]. **b** TRUPATH assays for binding of G_{i1} (left) and G_{o1} (right) proteins to hydrophobic E130X$^{3.41}$ variants. Responses were measured for varying isoprenaline concentrations and data fitted using the 3 parameter logistic equations. Data are *n*=2 (V,M,I,L,F,Y) or *n*=4 (E,W) independent biological experiments, each performed in duplicate. **c** TRUPATH assays for binding of G_{i1} (left) and G_{o1} (right) proteins to aromatic E130X$^{3.41}$ variants. Details are as in (**b**).

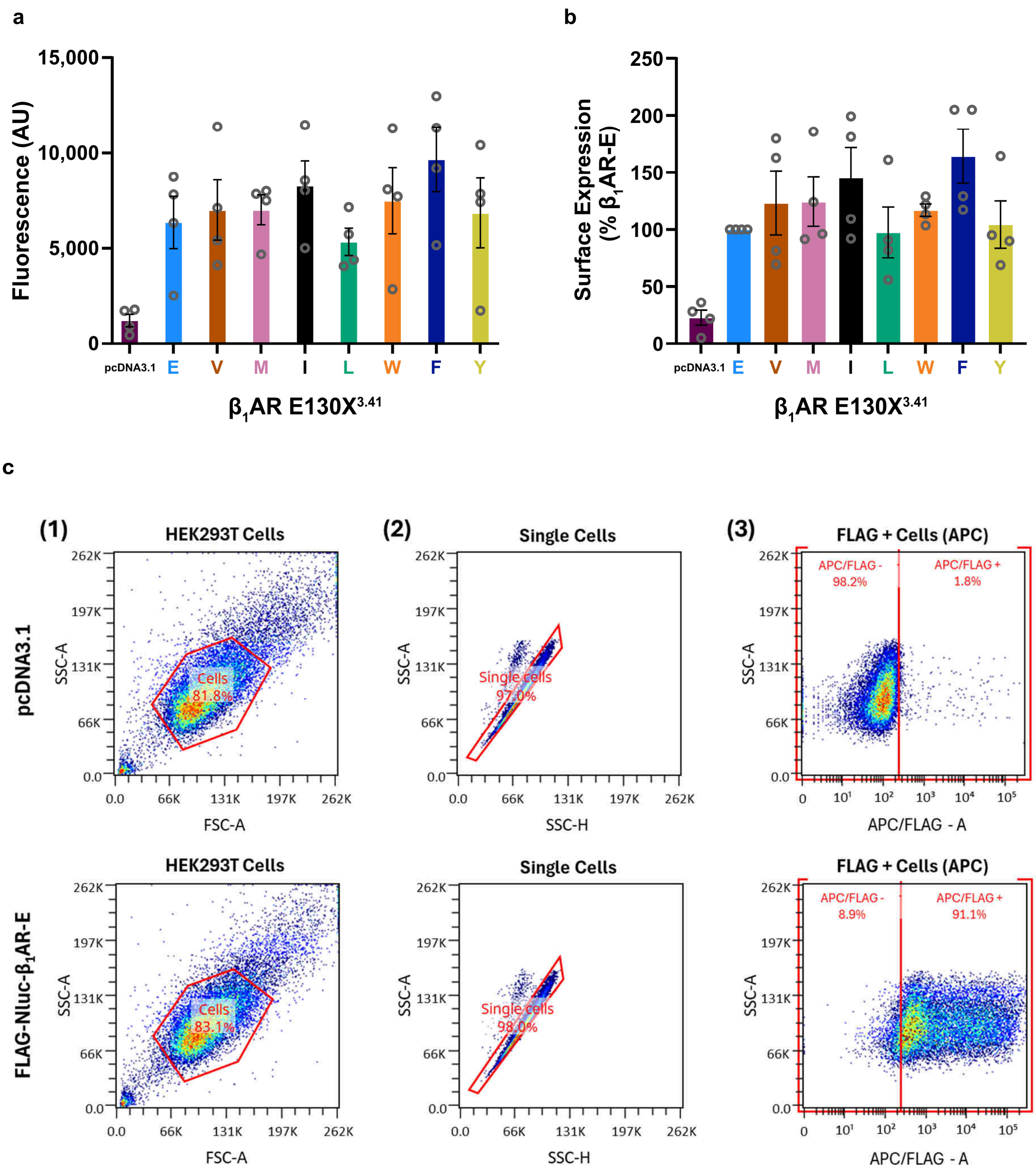

Supplementary Figure 6. Expression of β_1AR E130X$^{3.41}$ variants assessed by flow cytometry. a The surface expression of N-terminal FLAG-Nluc tagged β_1AR E130X$^{3.41}$ variants in HEK293T cells. Cells were fixed with 80% acetone, and expression was assessed with an allophycocyanin (APC) conjugated anti-FLAG antibody. Data are the mean ± SEM of *n*=4 independent biological experiments. Expression was compared to the vector control (pcDNA3.1). **b** Expression of E130X$^{3.41}$ variants in (**a**) normalised to the expression of β_1AR-E. Data are the mean ± SEM of *n*=4 independent biological experiments. Statistical significance was determined using a Kruskal-Wallis test for non-parametric data. No significant difference (p>0.3) was found for expression of any of the E130X$^{3.41}$ variants with β_1AR-E expression. **c** Gating Strategy for flow cytometry. HEK293T cells transfected with FLAG-Nluc-β_1AR E130X$^{3.41}$ variants (~5 x 10^5 cells) were pelleted (400 g for 5 min). Cells were subsequently washed in FACS Buffer (PBS, 1 % BSA) and stained using APC anti-FLAG Ab (Biolegend, 637307, 1 in 100 dilution in FACS buffer) for 30 mins at room temperature and in the dark. Cells were washed and resuspended in FACS buffer, with 3 additions of 1x volume of ice cold 80% acetone. Cells were incubated for 1 hour at 4 °C in the dark. Cells were washed again in FACS

buffer, resuspended in 300 µl FACS buffer and acquired using a BD LSRFortessa. Cells were gated for (1) HEK293T Cells, (2) Single Cells, and (3) FLAG+ based on APC fluorescence. Examples of data are shown for the vector control (pcDNA3.1) and for β_1AR-E.

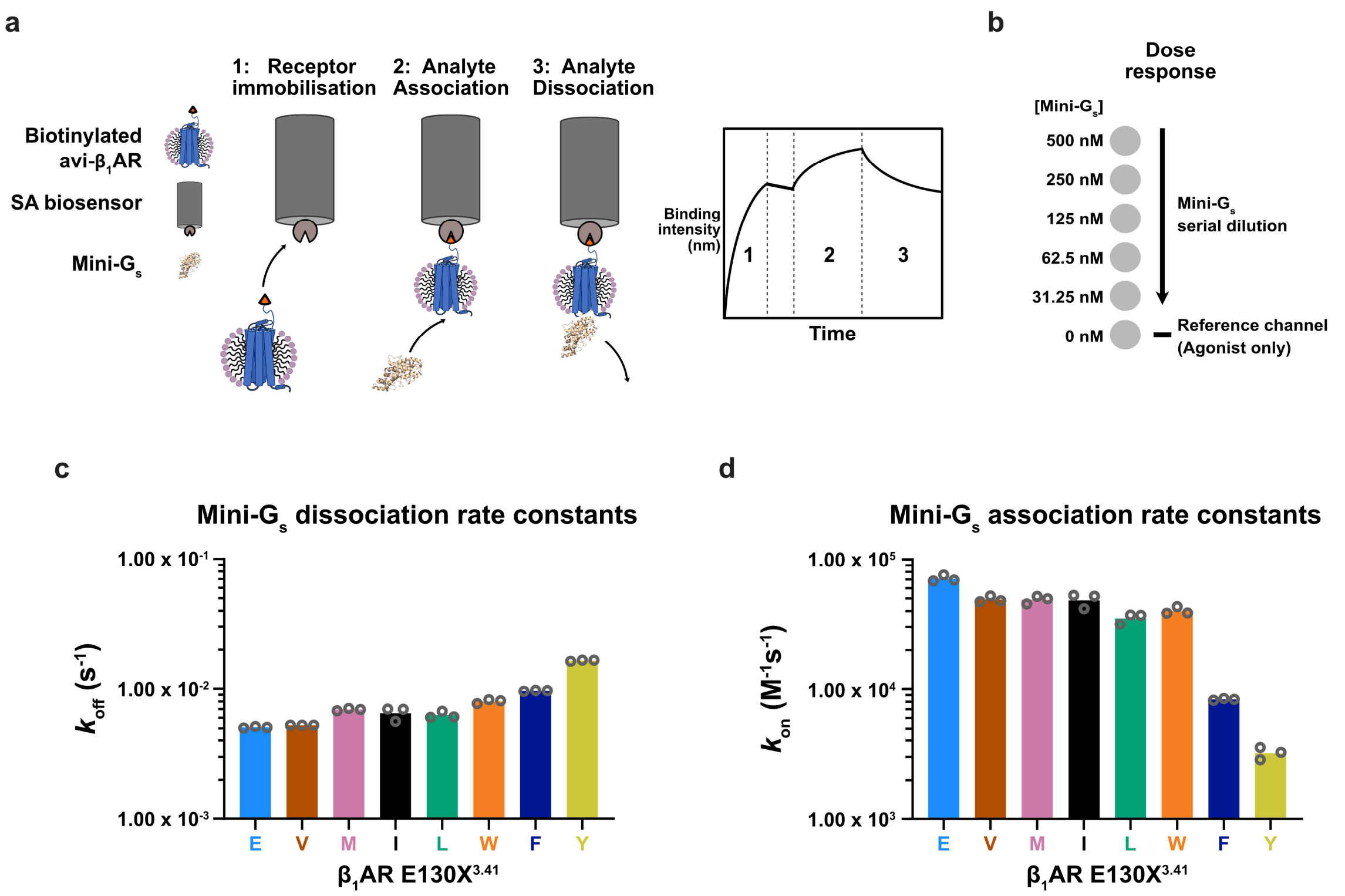

Supplementary Figure 7. E130X$^{3.41}$ mutations reduce β$_1$AR affinities for mini-G$_s$ in BLI assays. **a** A scheme for BLI experiments. Avi-tagged and biotinylated β$_1$AR E130X$^{3.41}$ variants are immobilised to Streptavidin (SA) coated biosensors. Binding of the mini-G$_s$ analyte to the immobilised β$_1$AR is measured in real time by recording changes to diffraction patterns when biosensors with immobilised receptors are dipped into wells containing mini-G$_S$, generating association curves. Dipping into buffer-only wells (without mini-G$_s$) results in dissociation of mini-G$_s$, generating dissociation curves. Kinetic parameters are calculated from fitting curves to raw data. See methods for details. **b** The experimental layout for dose response experiments for E130X$^{3.41}$ variant binding to mini-G$_s$. Analyte concentrations and reference wells are indicated. **c-d** Kinetic parameters for BLI dose-response experiments of mini-G$_s$ binding to isoprenaline-bound (500 μM) β$_1$AR E130X$^{3.41}$ variants. **c** Mini-G$_s$ dissociation (k_{off}) rate constants, **d** Mini-G$_s$ association (k_{on}) rate constants. Data points were calculated from dose-response experiments with n=3 independent technical replicates.

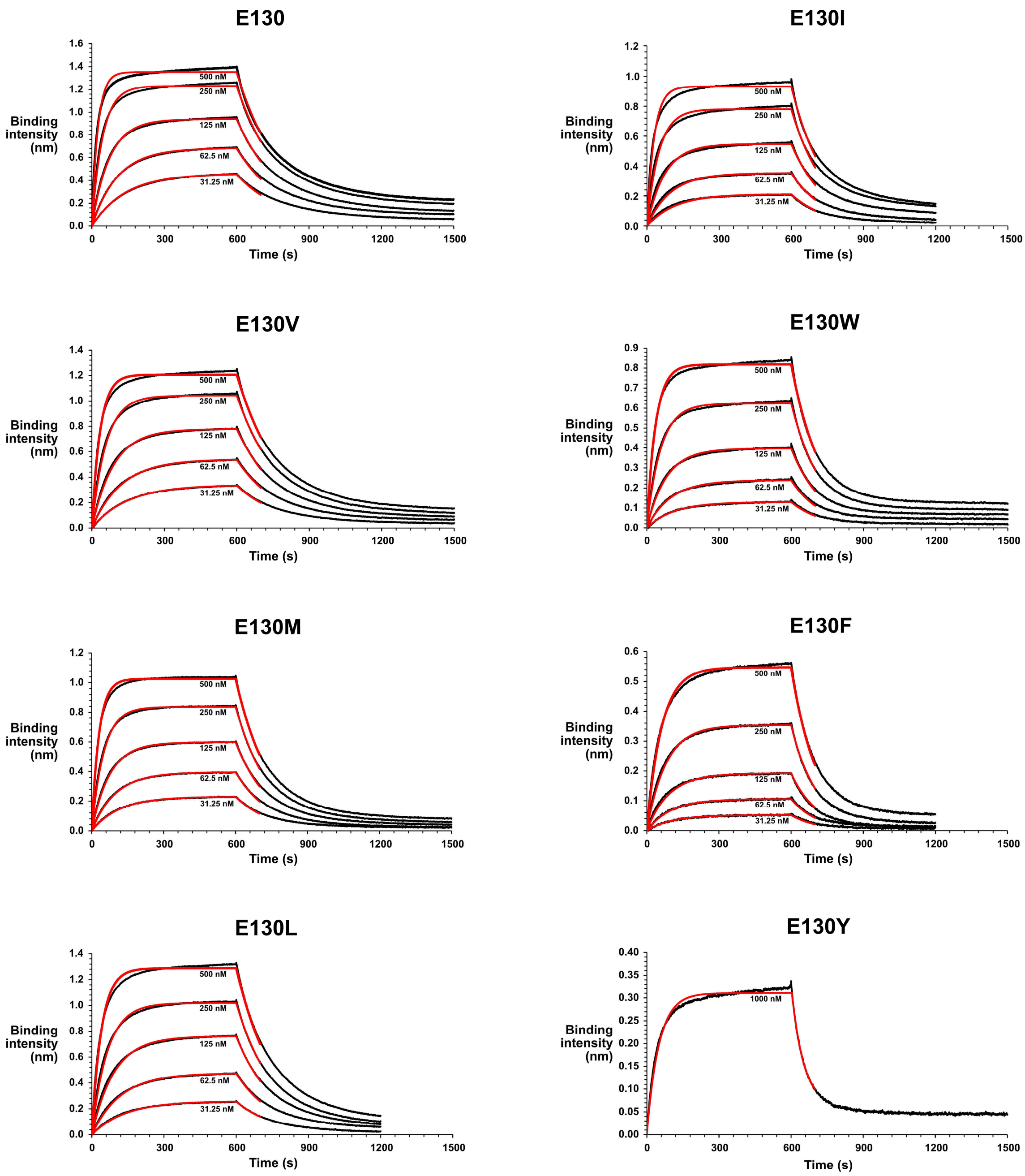

Supplementary Figure 8. Representative BLI dose-response experiments show differences in mini-G_s binding to β_1AR E130X$^{3.41}$ variants. Data are shown for each β_1AR E130X$^{3.41}$ construct, showing representative dose-response curves (from *n*=3 technical replicates), measuring mini-G_s binding kinetics in the presence of 500 µM isoprenaline (agonist). The mini-G_s concentrations utilised are indicated. Black lines show the processed binding curves. Red lines show the fitting, obtained using a 1:1 interaction stoichiometry global fitting with R_{max} unlinked by sensors. The full association window (600 s) and a partial dissociation window (100 s) were used to fit the data. The affinity of mini-G_s was significantly lower for the β_1AR-Y variant which prevented reliable dose-response experiments, and a single mini-G_s concentration (1000 nM) was used for this variant.

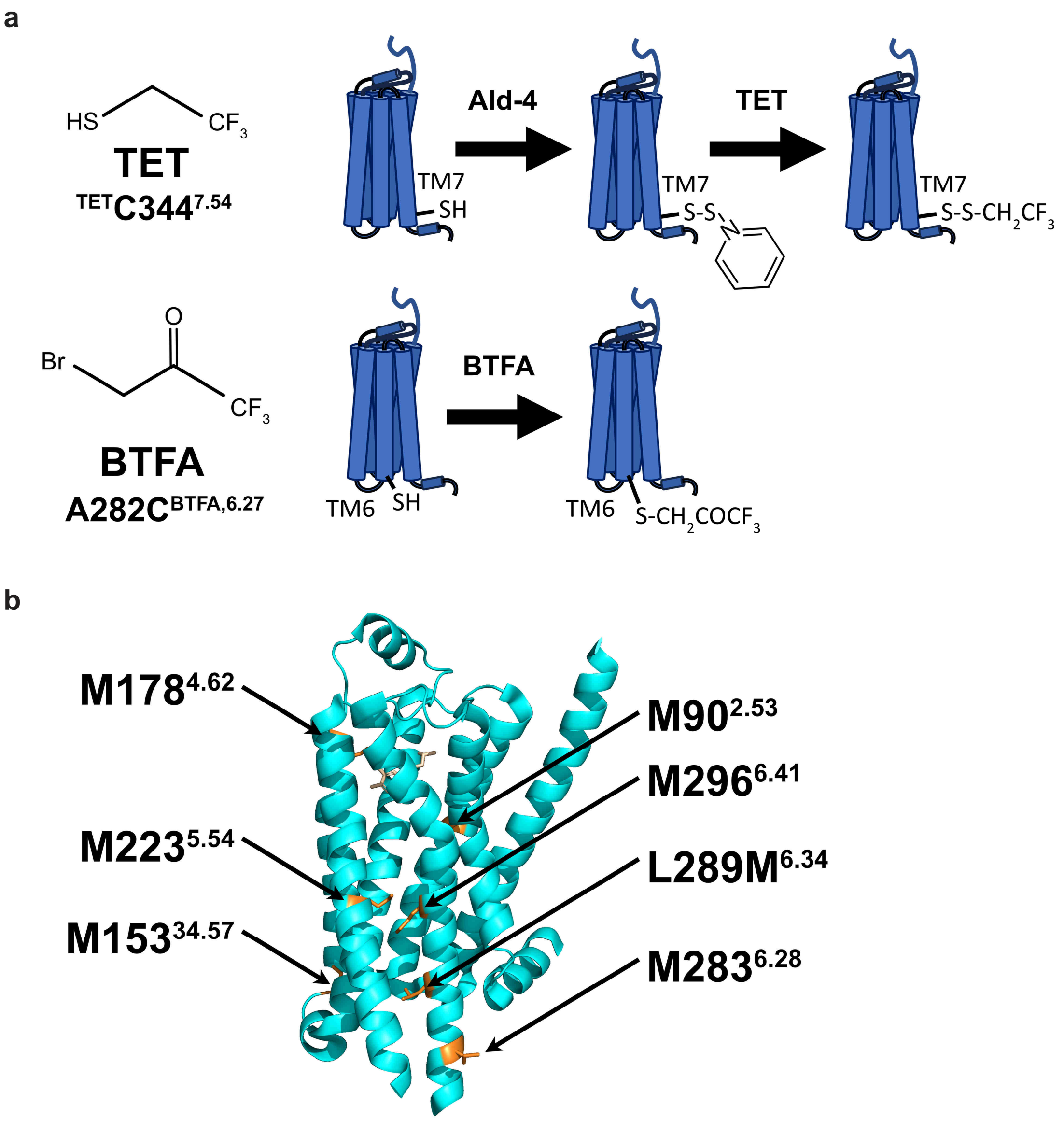

Supplementary Figure 9. Labelling of β_1AR E130X$^{3.41}$ variants for NMR studies. a A scheme for labelling of receptors with ^{19}F probes. Left, ^{19}F probes used in ^{19}F NMR experiments. Right, labelling protocols for ^{19}F probes. Ald-4, Aldrithiol-4™. See methods for experimental details. **b** Locations of ^{13}C-methyl-methionine probes in the β_1AR structure. Methionine probes (side chains in orange) are shown in the isoprenaline-bound x-ray crystal structure of turkey β_1AR (PDB ID: 2Y03)[3]. 2D ^{1}H-^{13}C HMQC NMR spectra show signals of the native methionine probes M1, M90$^{2.53}$ (TM2), M153$^{34.57}$ (IL2), M178$^{4.62}$ (extracellular loop (EL) 2), M223$^{5.54}$ (TM5), M283$^{6.28}$ (TM6), and M296$^{6.41}$ (TM6), all of which have been previously assigned[1]. The introduced probe L289M$^{6.34}$ (TM6) has also been assigned previously[6].

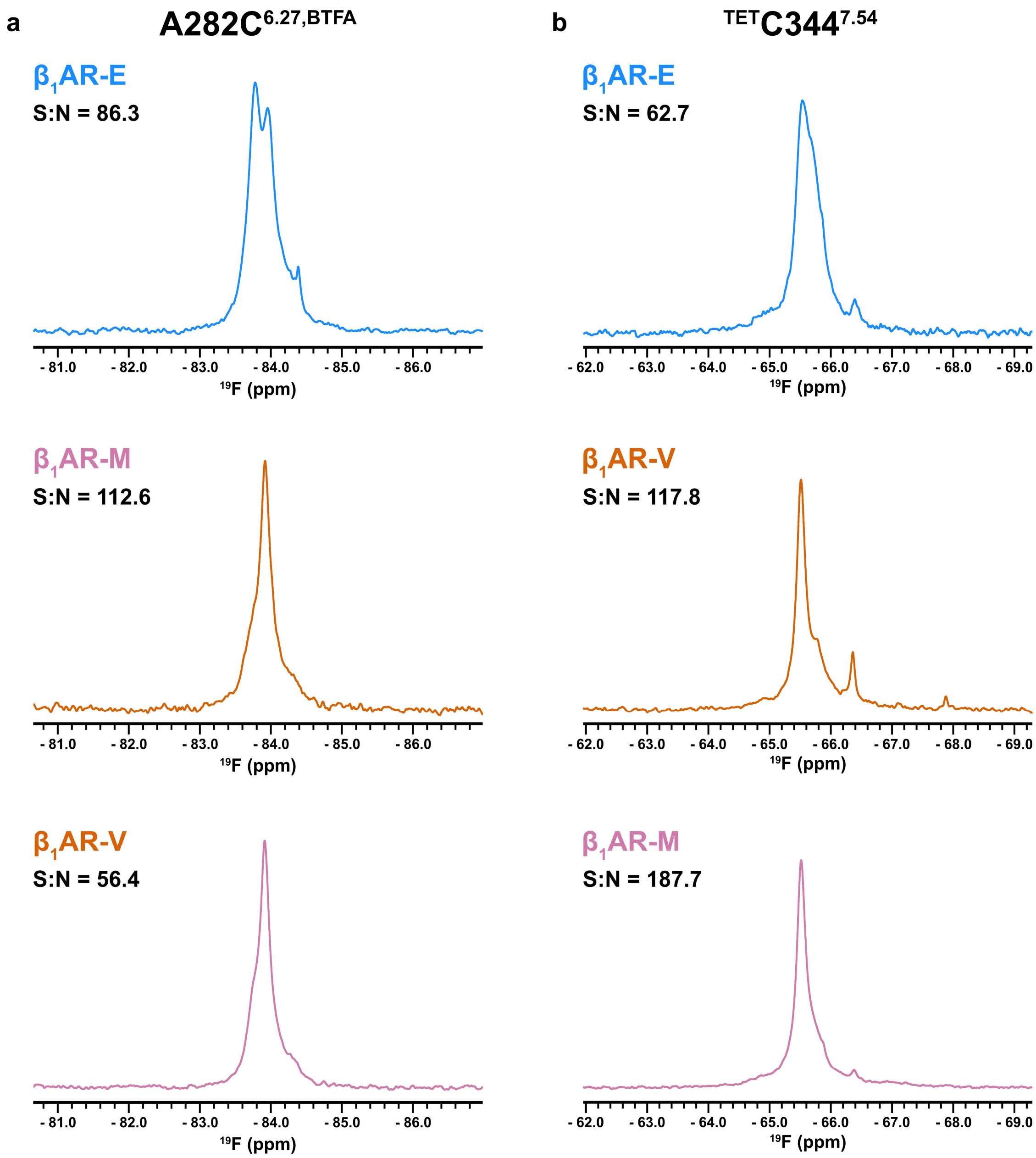

Supplementary Figure 10. ^{19}F NMR spectra of isoprenaline-bound β_1AR show differences in signals between E130X$^{3.41}$ variants. The 1D ^{19}F NMR spectra of isoprenaline-bound (1 mM) β_1AR E130X$^{3.41}$ variants with ^{19}F labelling (left, TM6 labelling at A282C$^{6.27}$ with BTFA, right, TM7 labelling at C344$^{7.54}$ with TET), showing the individual processed spectra. Deconvolutions are shown in Fig. 2b. Larger spectral windows are shown to demonstrate the high quality of the spectra. The signal-to-noise ratio (S:N) is given for each spectrum.

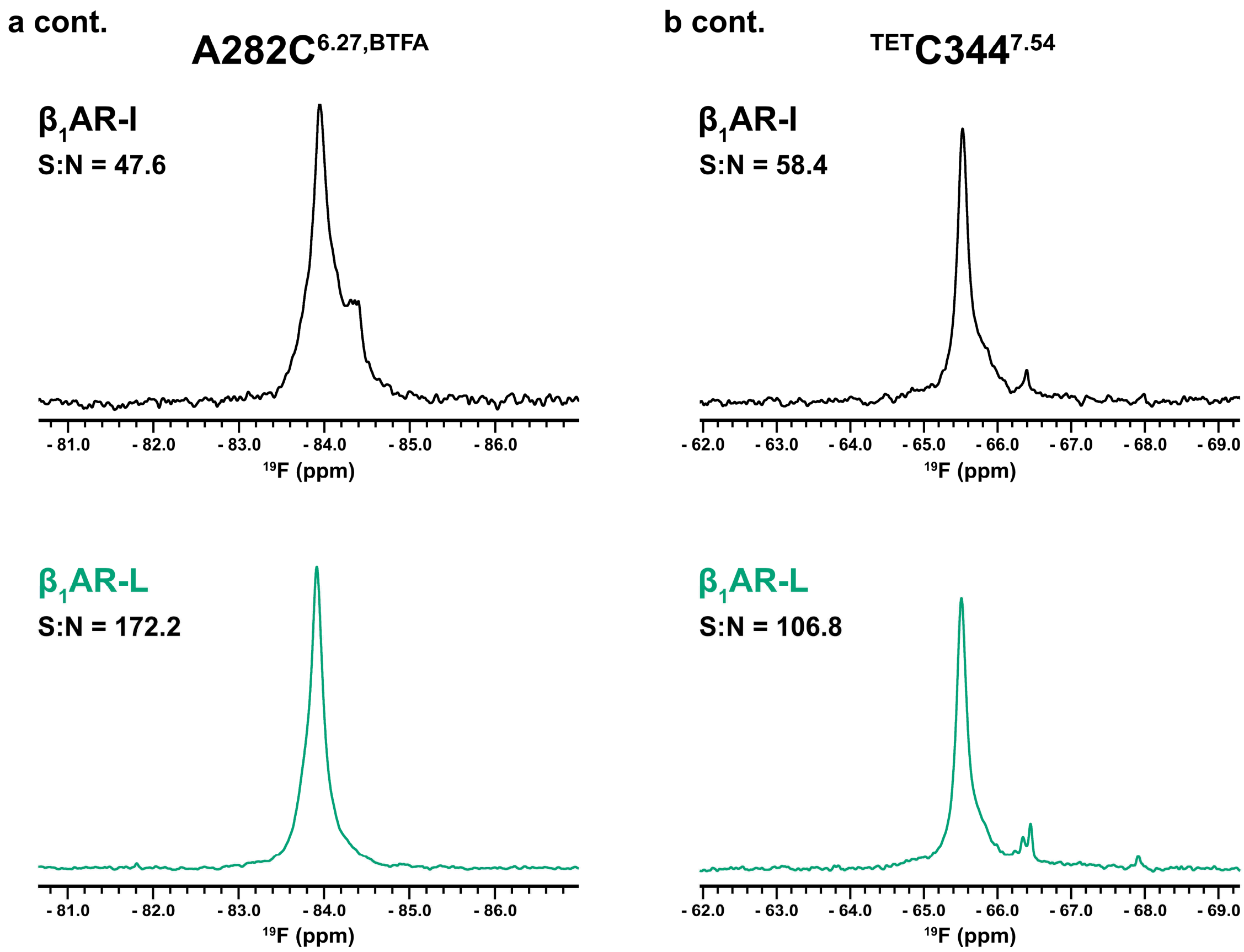

Supplementary Figure 10. Continued

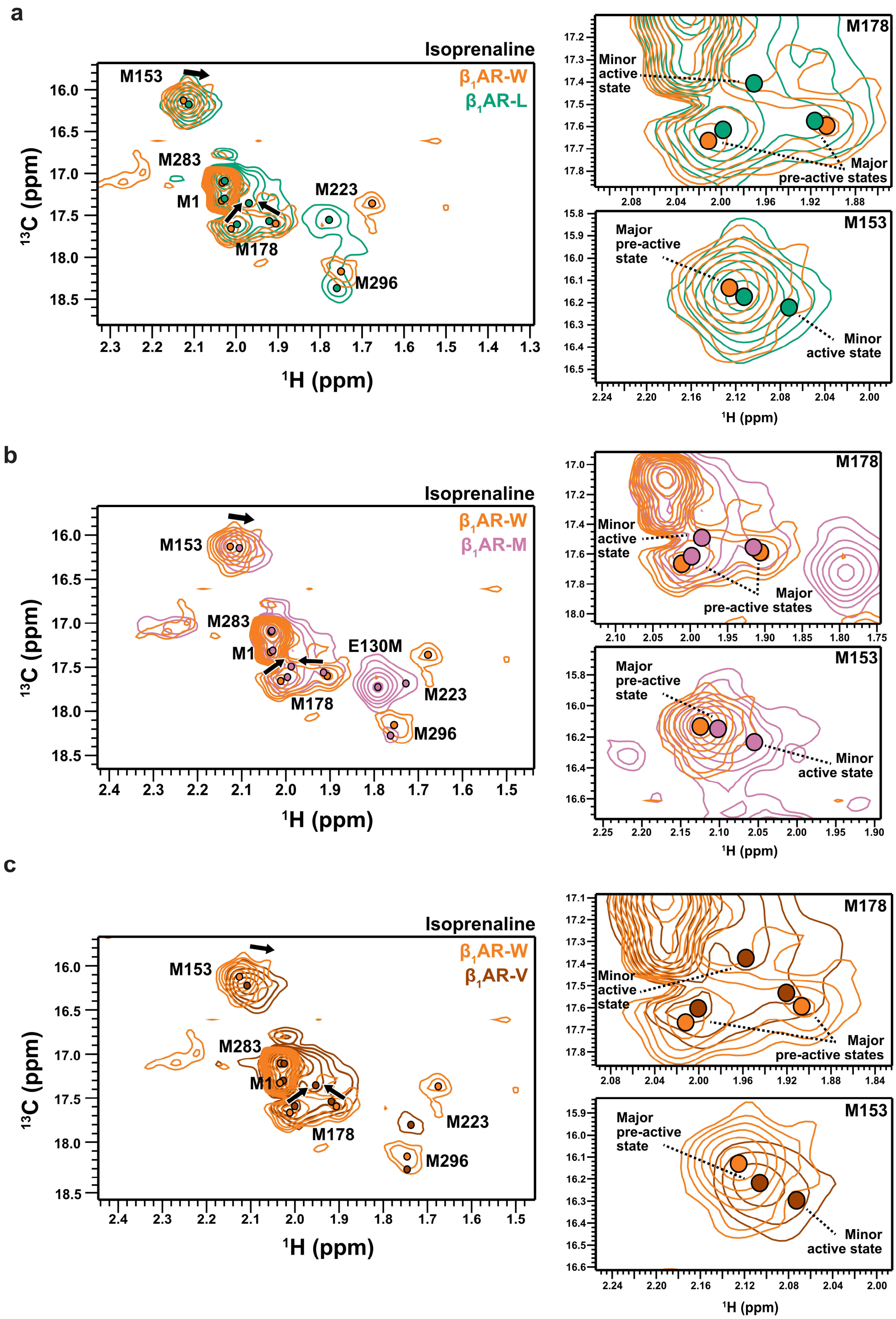

Supplementary Figure 11. E130X$^{3.41}$ hydrophobic mutations shift β$_1$AR equilibria towards the pre-active state in ^{1}H-^{13}C NMR studies. Overlays of 2D ^{1}H-^{13}C HMQC NMR spectra comparing β$_1$AR hydrophobic E130X$^{3.41}$ variants with β$_1$AR-W indicate occupation of the pre-active state with minor active

state populations in hydrophobic E130X$^{3.41}$ variants, for **a** β_1AR-L, **b** β_1AR-M, and **c** β_1AR-V, in the presence of 1 mM isoprenaline and recorded at 308 K. Left, 2D spectra overview. Right, enlargements of the M178$^{4.62}$ probe region (top) or the M153$^{34.57}$ probe region (bottom), highlighting the minor signal intensity observed at the active state positions of M153$^{34.57}$ in IL2 and M178$^{4.62}$ reporting on the extracellular orthosteric ligand binding pocket. Notably, the spectra demonstrate concerted changes in the active/pre-active state equilibrium position which affects the entire receptor, inclusive of the orthosteric pocket occupied by the full agonist.

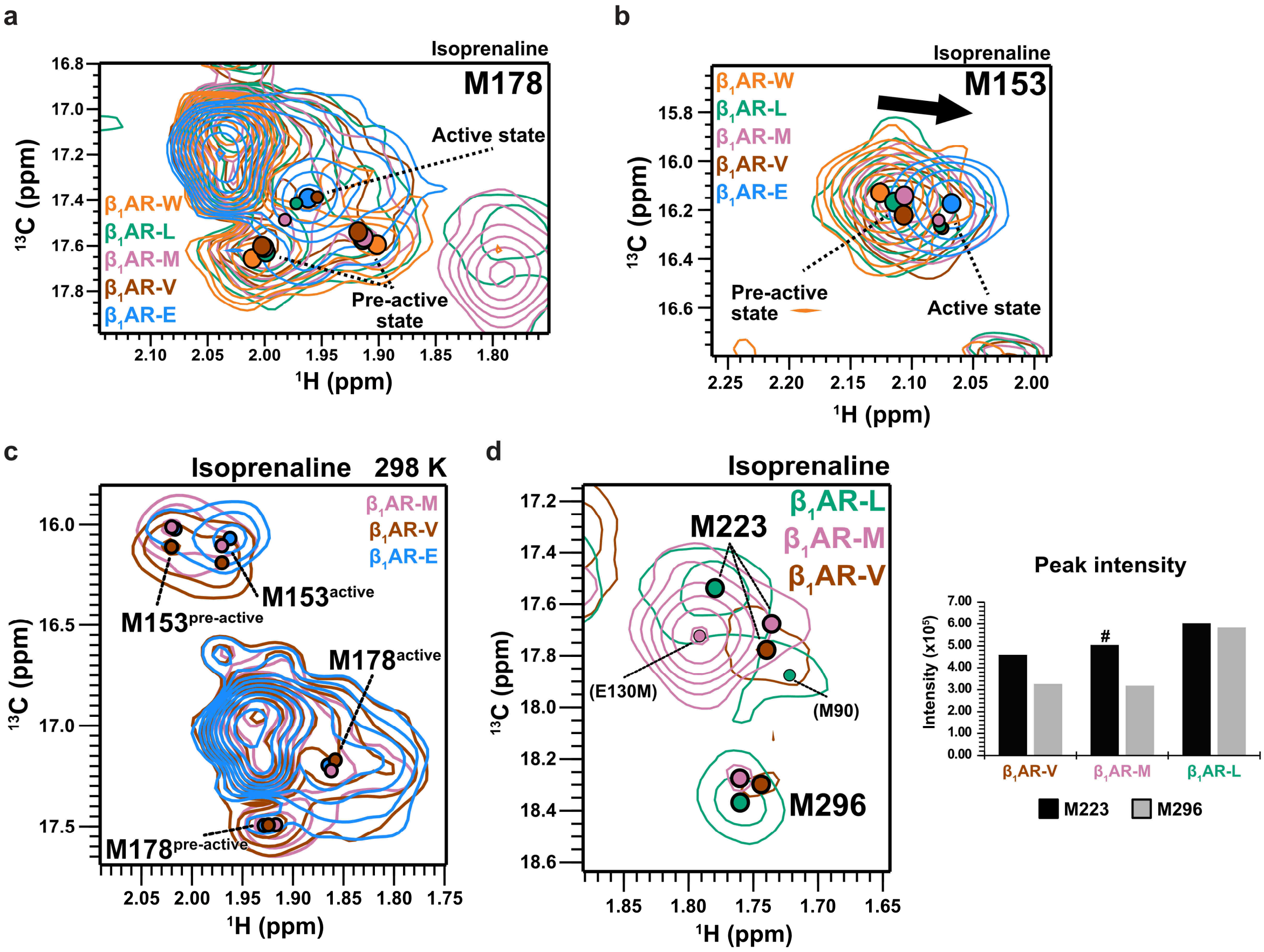

Supplementary Figure 12. E130X$^{3.41}$ hydrophobic mutations show minor active state populations in ^{1}H-^{13}C NMR studies. a An overlay of 2D ^{1}H-^{13}C HMQC NMR spectra of isoprenaline-bound (1 mM) β_1AR-W, β_1AR-L, β_1AR-M, β_1AR-V, and β_1AR-E recorded at 308 K, showing an enlargement of the M178$^{4.62}$ peak position region. Pre-active states and active state populations are indicated to illustrate the relative active state populations between the E130X$^{3.41}$ variants. β_1AR-W populates the pre-active state; β_1AR-V, β_1AR-M, and β_1AR-L show minor active state populations at similar chemical shifts to the active state position observed in β_1AR-E. **b** An overlay of 2D spectra from (**a**) showing an enlargement of M153$^{34.57}$. **c** An overlay of 2D ^{1}H-^{13}C HMQC spectra of isoprenaline-bound (1 mM) β_1AR-M, β_1AR-V, and β_1AR-E recorded at 298 K, showing the M178$^{4.62}$ and M153$^{34.57}$ regions. The lower temperature resulted in reduced exchange broadening and resolving of the active and pre-active states, which are indicated, further demonstrating the minor active populations of relative to β_1AR-M and β_1AR-V, relative to β_1AR-E. **d** An overlay of 2D ^{1}H-^{13}C HMQC spectra for isoprenaline-bound (1 mM) β_1AR-L, β_1AR-M, and β_1AR-V recorded at 308 K, showing an enlargement of the spectral region for the pre-active state signals of M223$^{5.54}$ and M296$^{6.41}$, located in the receptor core around the P$^{5.50}$-I$^{3.40}$-F$^{6.44}$ motif. The differences in pre-active state signal intensities are shown in the graph on the right. Decreases in signal intensity are indicative of conformational broadening and thus increased active state population for β_1AR-M and β_1AR-V, relative to β_1AR-L, due to the greater intermediate-to-slow exchange. # indicates M223$^{5.54}$ in β_1AR-M which partially overlapped with the E130M$^{3.41}$ signal so that there is some contribution from this peak to the signal intensity.

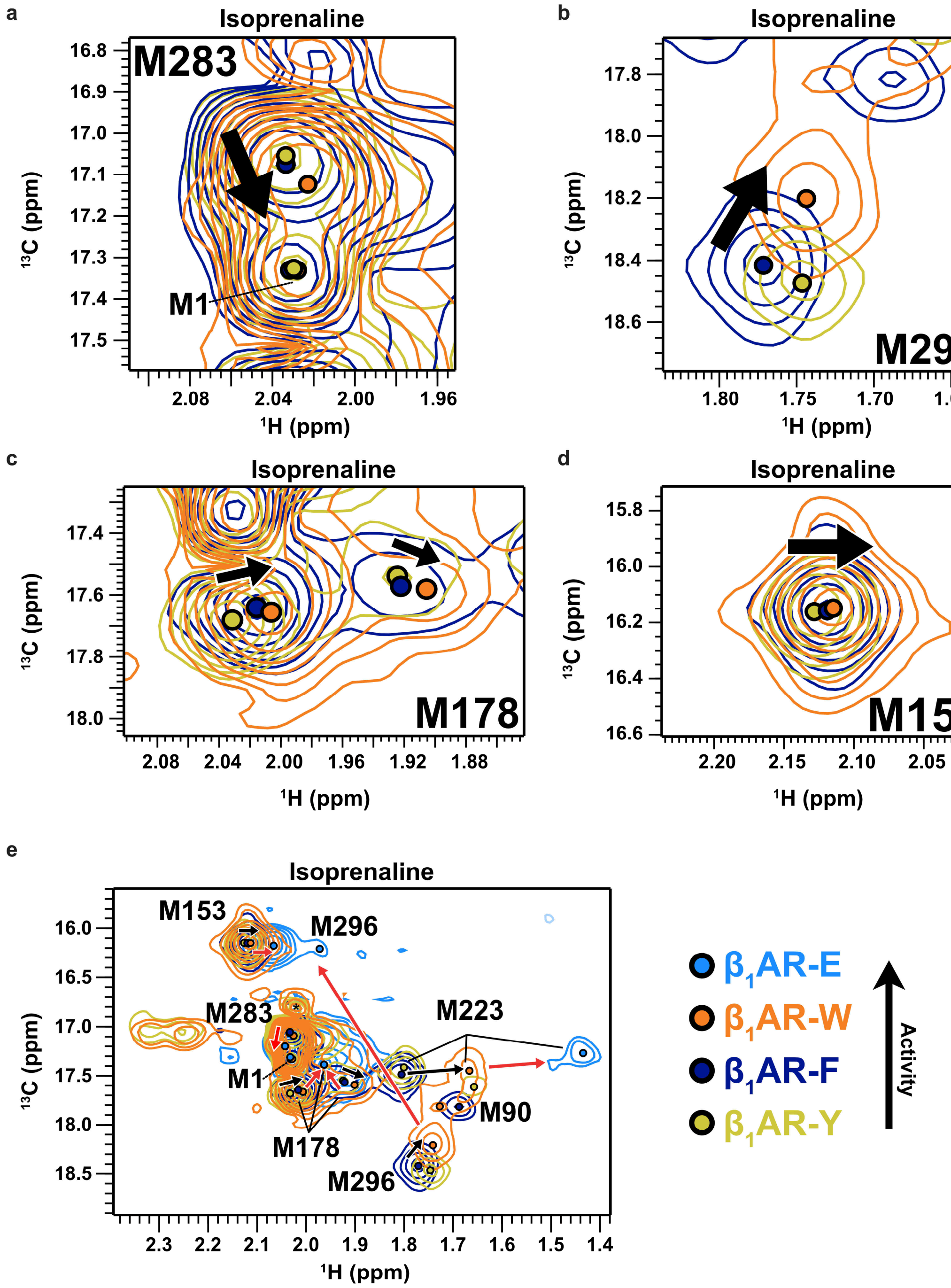

Supplementary Figure 13. E130X$^{3.41}$ aromatic mutations shift β_1AR equilibria towards the inactive state in ^{1}H-^{13}C NMR studies. Enlargements of overlays of the 2D ^{1}H-^{13}C HMQC spectra shown in Fig. 2d, for β_1AR aromatic E130X$^{3.41}$ variants in the presence of isoprenaline (1 mM), recorded at 308 K. **a**-**d** Enlargements of spectra for regions showing the M283$^{6.28}$ peak (**a**), the M296$^{6.41}$ peak (**b**), the M178$^{4.62}$ peak (**c**), or the M153$^{34.57}$ peak (**d**). Black arrows indicate changes in peak positions between E130W/F/Y$^{3.41}$ (which populate the rapidly exchanging inactive/pre-active state equilibrium) in increasing order of receptor activation. **e** An overlay of 2D ^{1}H-^{13}C HMQC spectra for β_1AR-E and E130W/F/Y$^{3.41}$ recorded in the presence of isoprenaline (1 mM) and at 308 K. Changes in peak positions for E130W/F/Y$^{3.41}$ in increasing order of receptor activation are indicated by black arrows. Changes in peak positions between E130$^{3.41}$ (active state) and E130W$^{3.41}$ (pre-active state) are indicated with red arrows. As seen for the hydrophobic residue E130X$^{3.41}$ variants, the spectra of aromatic residue E130X$^{3.41}$ variants show concerted changes affecting the entire receptor, including around the agonist-bound orthosteric pocket.

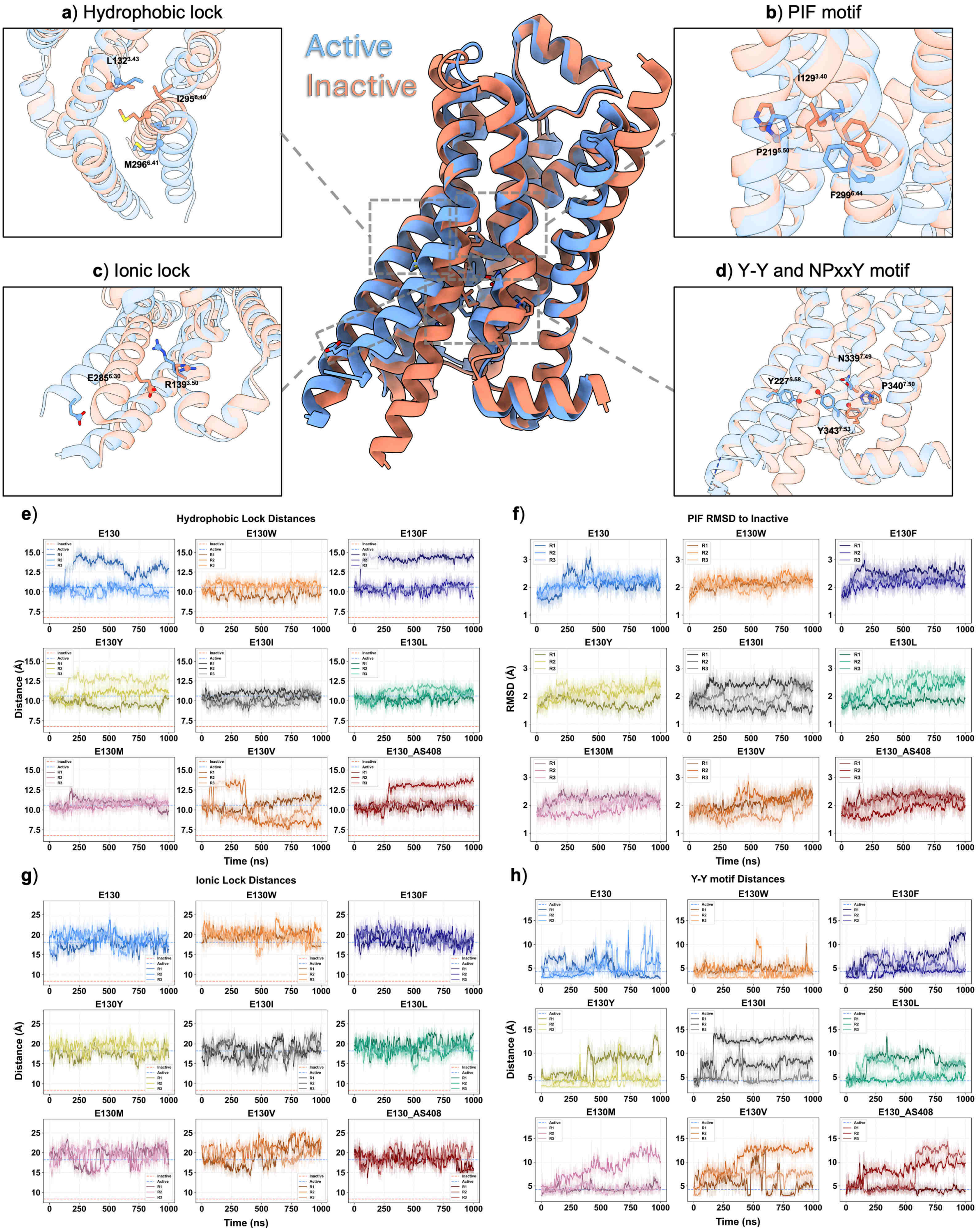

Supplementary Figure 14. Analysis of GPCR activation microswitches in MD simulations of β$_1$AR E130X$^{3.41}$ variants. a-**d** The aligned structures of the β$_1$AR active state (PDB ID:7JJO, in blue)[8] and inactive state (PDB ID:2Y03, in salmon)[3] and the activation markers examined in MD simulations of β$_1$AR E130X$^{3.41}$ variants. Atoms used for measurements in (**e**-**h**) are shown as spheres in (**a**-**d**). **e**-**h** Distances and RMSD over the 1 μs simulation windows for measurements of GPCR activation microswitch conformations. Each line in the graphs represents a single replicate (*n*=3). Reference values calculated from the active and inactive structures are shown as dashed lines with their corresponding colours in (**e**), (**g**), and (**h**) when present. The TM3-TM6 hydrophobic lock, measured as the distance between the Cα atoms of L$^{3.43}$-M$^{6.41}$ (**a**), remains open in all variants (**e**), indicating the active state is maintained. The PIF motif, measured as

the Cα root mean square deviation (RMSD) after backbone alignment to the inactive state (**b**), remains in an active-like conformation in all variants (**f**). The ionic lock, measured as the distance between the $R^{3.50}$CZ-$E^{6.30}$CD atoms (**c**), remains open in all variants (**g**). The Y-Y motif, measured as the distance between the $Y^{5.58}$OH-$Y^{7.53}$OH atoms (**d**), is the most disrupted motif, with some replicates showing signs of deactivation across all mutants (**h**). In (**e**-**h**), the traces are the rolling mean of a 5 ns window, with the unaveraged values shown as faint traces.

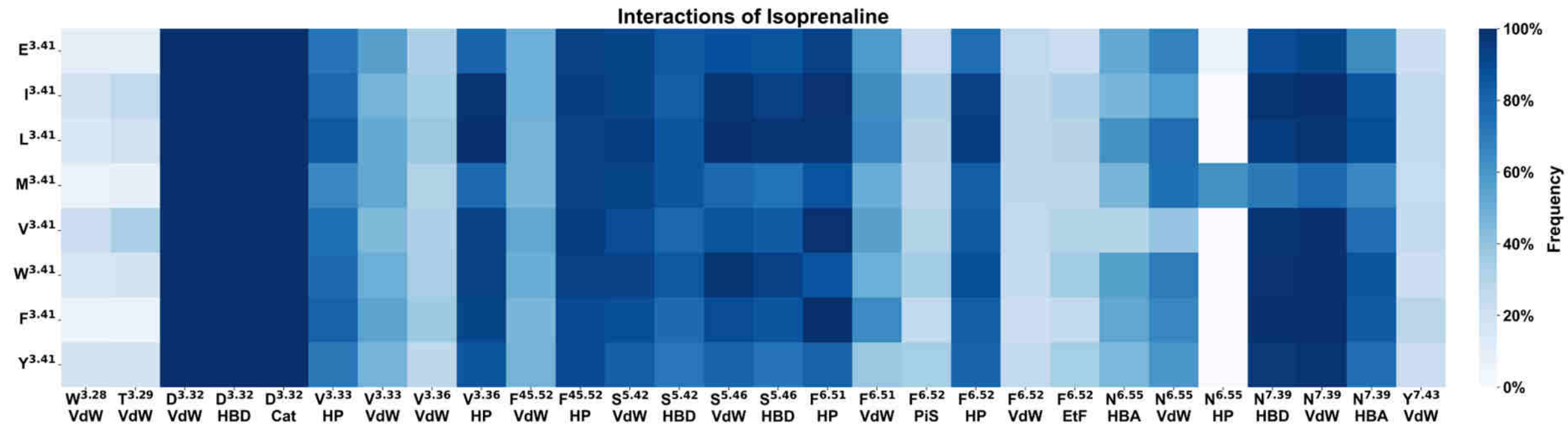

Supplementary Figure 15. MD simulations show interactions of isoprenaline in the orthosteric binding site are similar across β$_1$AR E130X$^{3.41}$ variants. Measurements of isoprenaline interactions were the average of MD simulation replicates (n=3). Interaction types are indicated below each column and include Van der Waals (VdW), hydrogen bond donor (HBD), hydrogen bond acceptor (HBA), hydrophobic (HP), π Stacking (PiS), edge-to-face (EtF) and cationic (Cat) interactions. Only residues with at least one interaction exceeding 15% frequency on average across the three 1 μs replicates are shown.

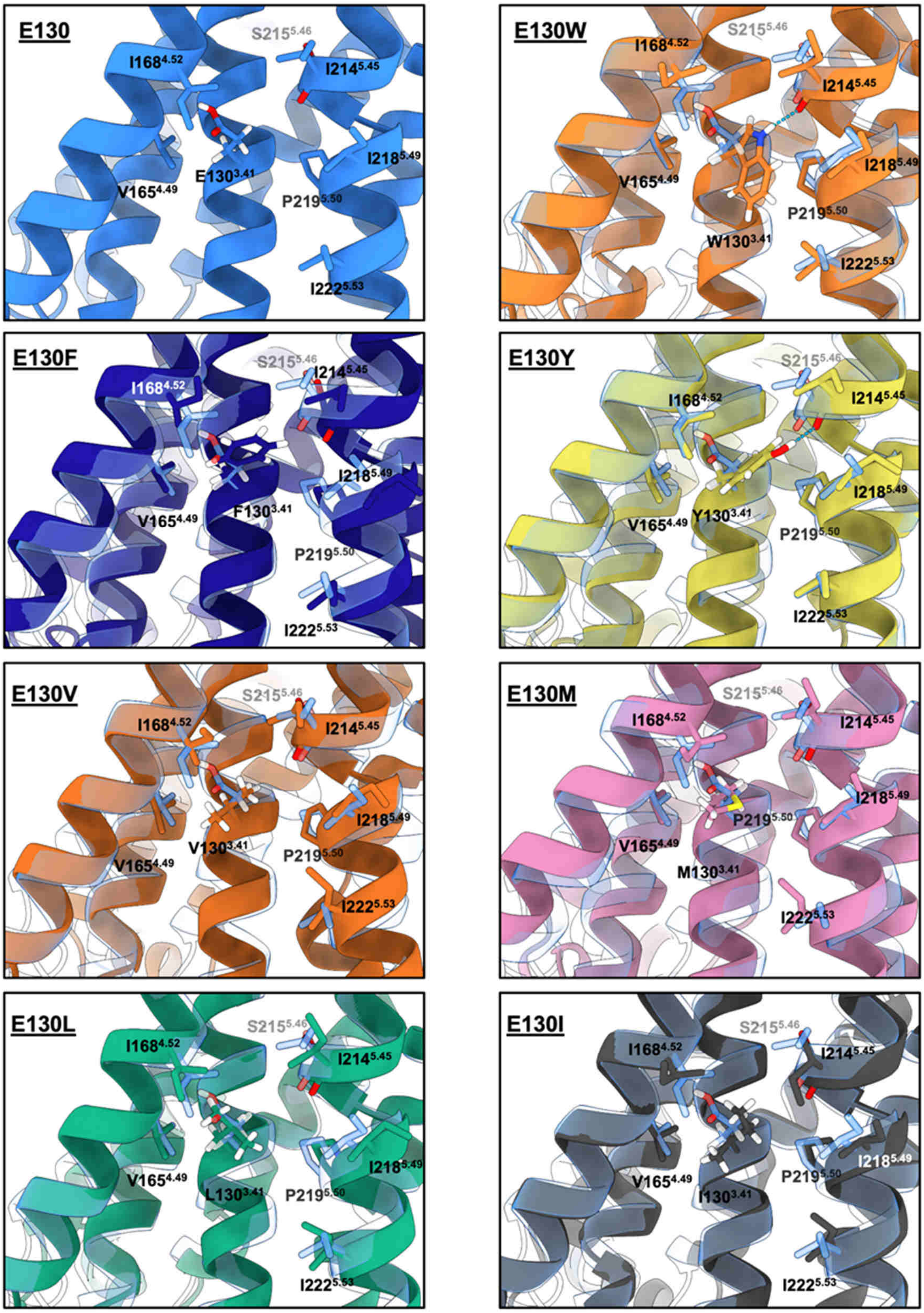

Supplementary Figure 16. Snapshots of the β_1AR-X$^{3.41}$ local pocket. Representative structural snapshots from MD simulations (*n*=3 replicates) showing the local arrangement of the 3.41 position and neighbouring residues in TM3, TM4, and TM5 for β_1AR-E, β_1AR-W, β_1AR-F, β_1AR-Y, β_1AR-V, β_1AR-M, β_1AR-L, and β_1AR-I. Only residues with at least one interaction with an average frequency over 10% are shown. Hydrogen bonds between residues are shown with a blue dashed line if present.

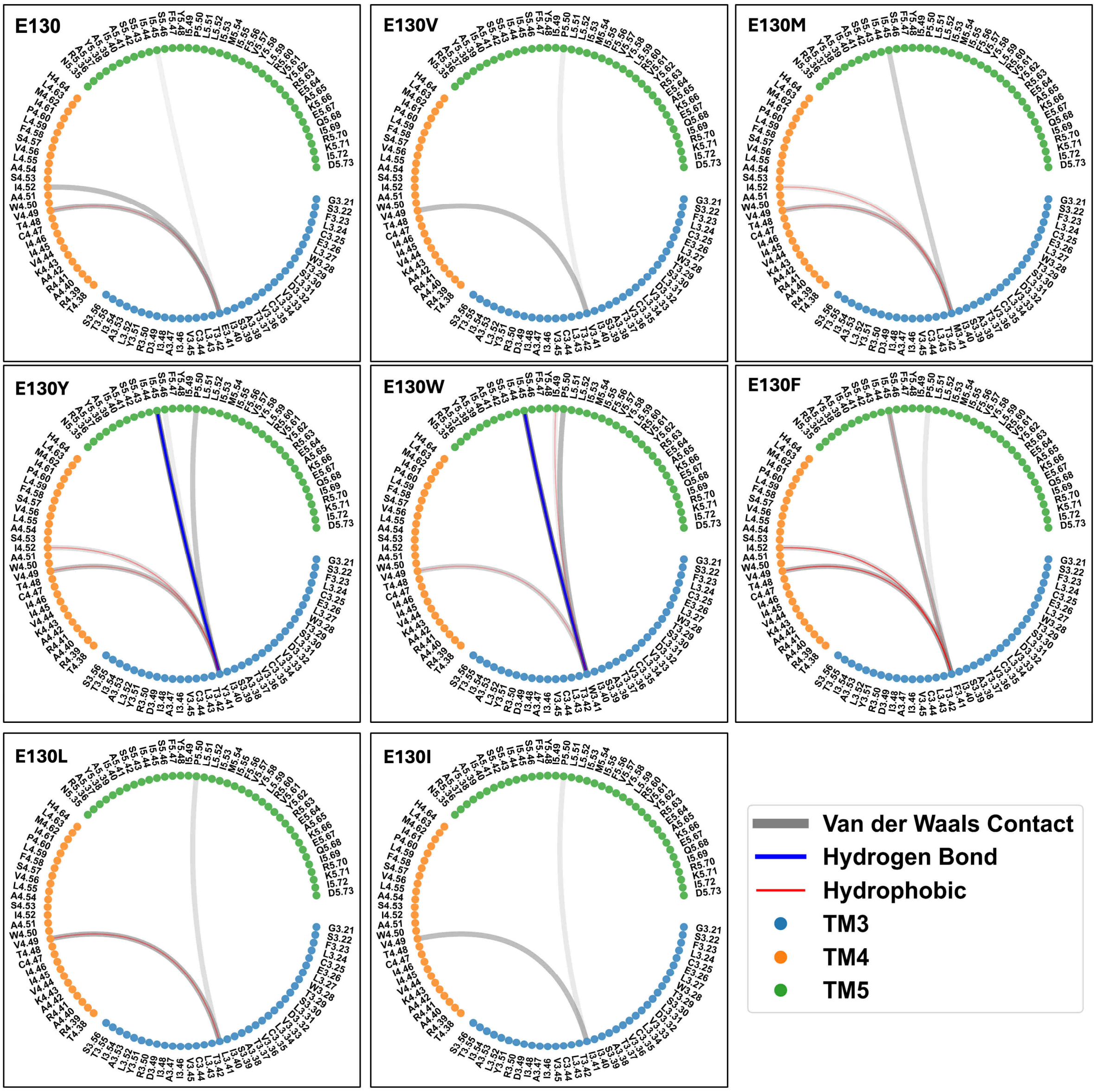

Supplementary Figure 17. Flare-plots depicting interactions of residue $X^{3.41}$ in MD simulations of β_1AR-$X^{3.41}$. Line colours indicate interaction type: Van der Waals contacts (grey), hydrogen bonds (blue), and hydrophobic interactions (red). Residues are coloured depending on the TM they belong to: TM3 (blue), TM4 (orange), and TM5 (green). Line thickness reflects contact frequency, calculated from all MD simulation replicates (*n*=3). Interactions are shown for residues that possess at least one interaction with an average frequency above 10%. Interactions with the four immediately adjacent residues on either side of the 3.41 position are excluded to highlight longer-range contacts**.** Aromatic mutants establish increased hydrogen bond and Van der Waals interactions with TM5 residues, particularly I214$^{5.45}$ and P219$^{5.50}$, compared to E130$^{3.41}$ and hydrophobic mutants.

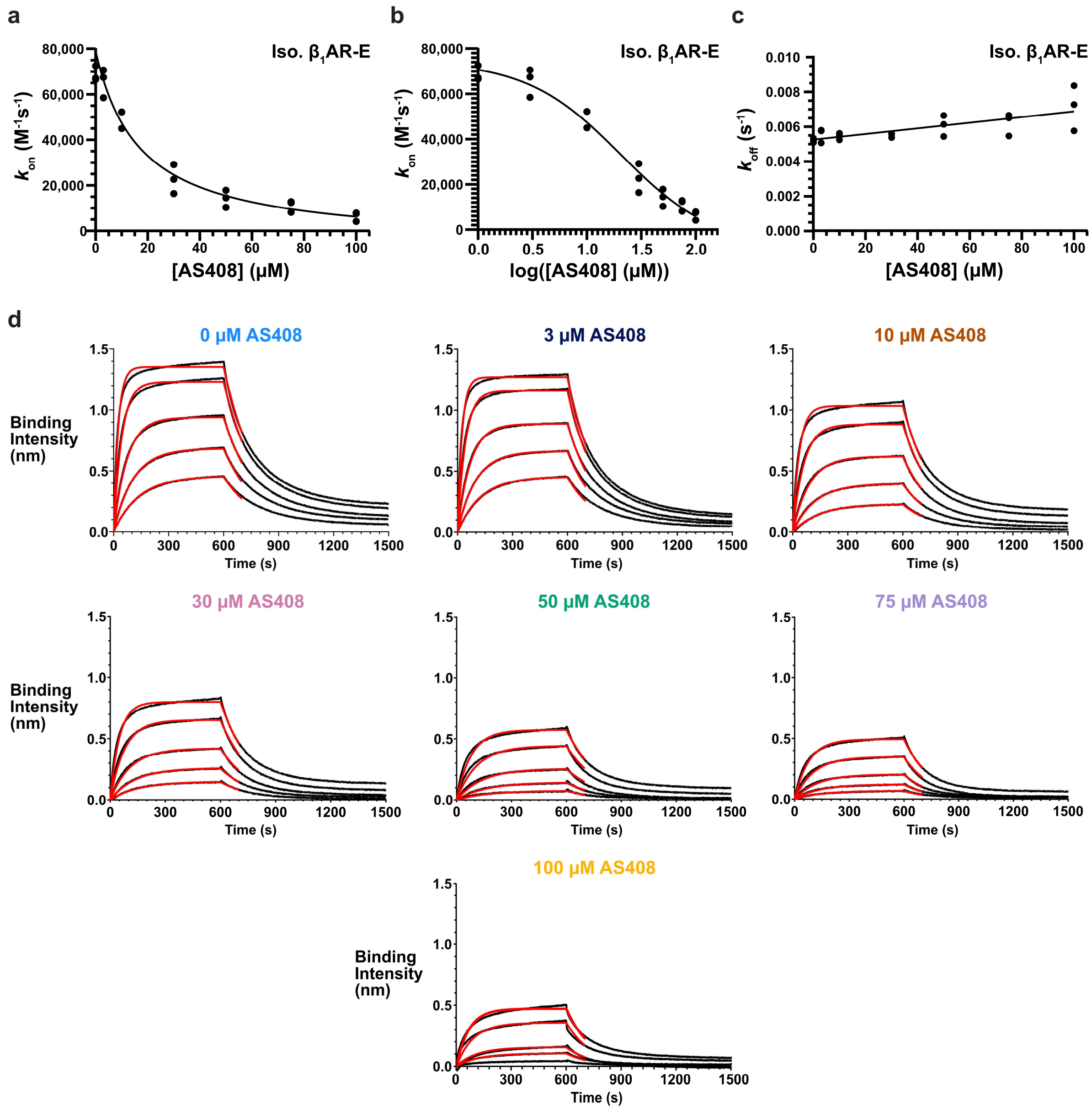

Supplementary Figure 18. AS408 decreases mini-G$_s$ coupling to isoprenaline-bound β$_1$AR-E in BLI assays. a Effects of AS408 on the mini-G$_s$ association rate constant to β$_1$AR-E in the presence of isoprenaline (500 μM), measured by BLI. For each concentration of AS408, dose-response experiments were recorded with *n*=3 independent technical replicates. Data were fit in GraphPad Prism 10.5 with an IC$_{50}$ curve using [Inhibitor] vs Response (three parameter variable slope), with a Hill constant fixed at -1. **b** Data in (**a**) plot with a log([AS408]) scale. **c** Effects of AS408 on the mini-G$_s$ dissociation rate constant from β$_1$AR-E in the presence of isoprenaline (500 μM). BLI assay details are as in (**a**). For clarity, a linear fit through the data is shown. **d** Representative (*n*=3) BLI dose-response curves showing the concentration-dependent effects of AS408 on mini-G$_s$ binding to isoprenaline-bound (500 μM) β$_1$AR-E. The mini-G$_s$ concentrations utilised were 500 μM, 250 μM, 125 μM, 62.5 μM, and 31.25 μM. Red lines show the fitting, obtained using a 1:1 interaction stoichiometry global fitting with R_{max} unlinked by sensors. The full association window (600 s) and a partial dissociation window (100 s) were used to fit the data.

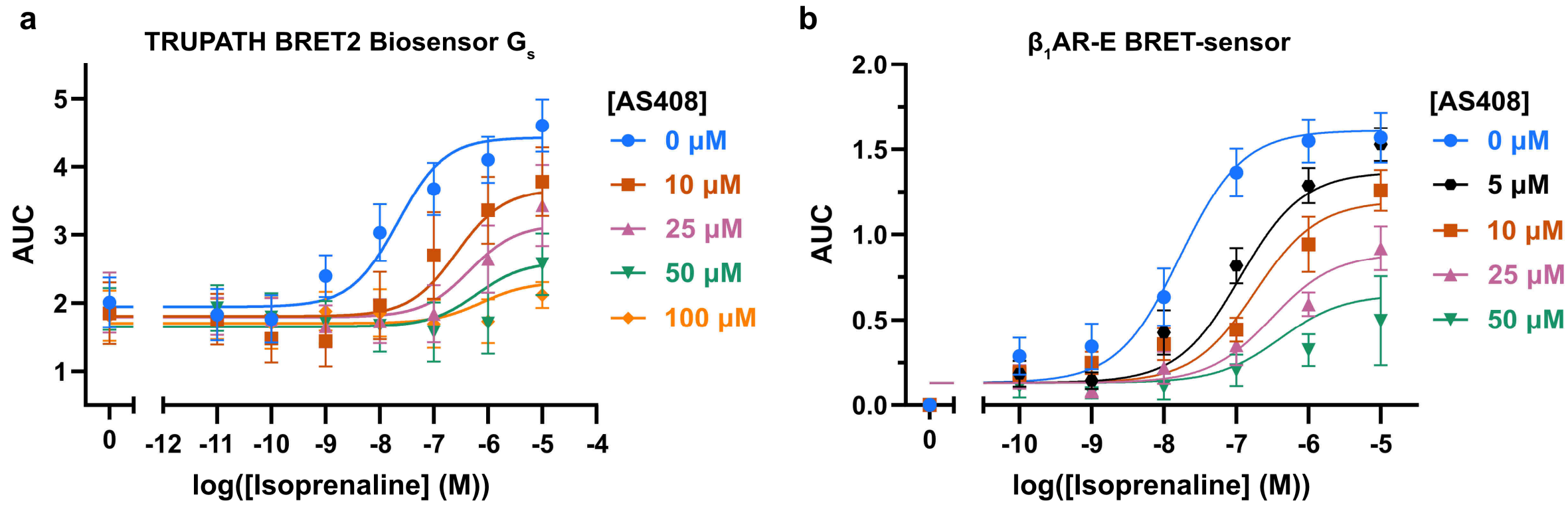

Supplementary Figure 19. Application of the operational model of allostery to pharmacological data reveals AS408 as a NAM at the turkey $β_1$AR. a Data in Fig. 3a (isoprenaline concentration response curves for the TRUPATH BRET2 biosensor G_s with varying AS408 concentrations; data are mean ± SEM of *n*=3 independent biological experiments, each performed in duplicate) were fit with the complete operational model of allostery[9]. Maximal responses were fixed. The Hill coefficient was set to 1.0 and $τ_B$- was fixed at zero with the assumption that AS408 is a NAM for agonists, following the approach established for characterising AS408 effects at the h$β_2$AR[10]. **b** Data in Fig. 6c (isoprenaline concentration response curves for the $β_1$AR-E BRET-sensor with varying AS408 concentrations; data are mean ± SEM of *n*=4 biological measurements, each performed in duplicate) were fit with the complete operational model of allostery. Maximal responses were fixed, and parameters fixed as in (**a**). For (**a**-**b**), parameters for these fits (K_A, K_B, α, and β values) can be found in Supplementary Table 2.

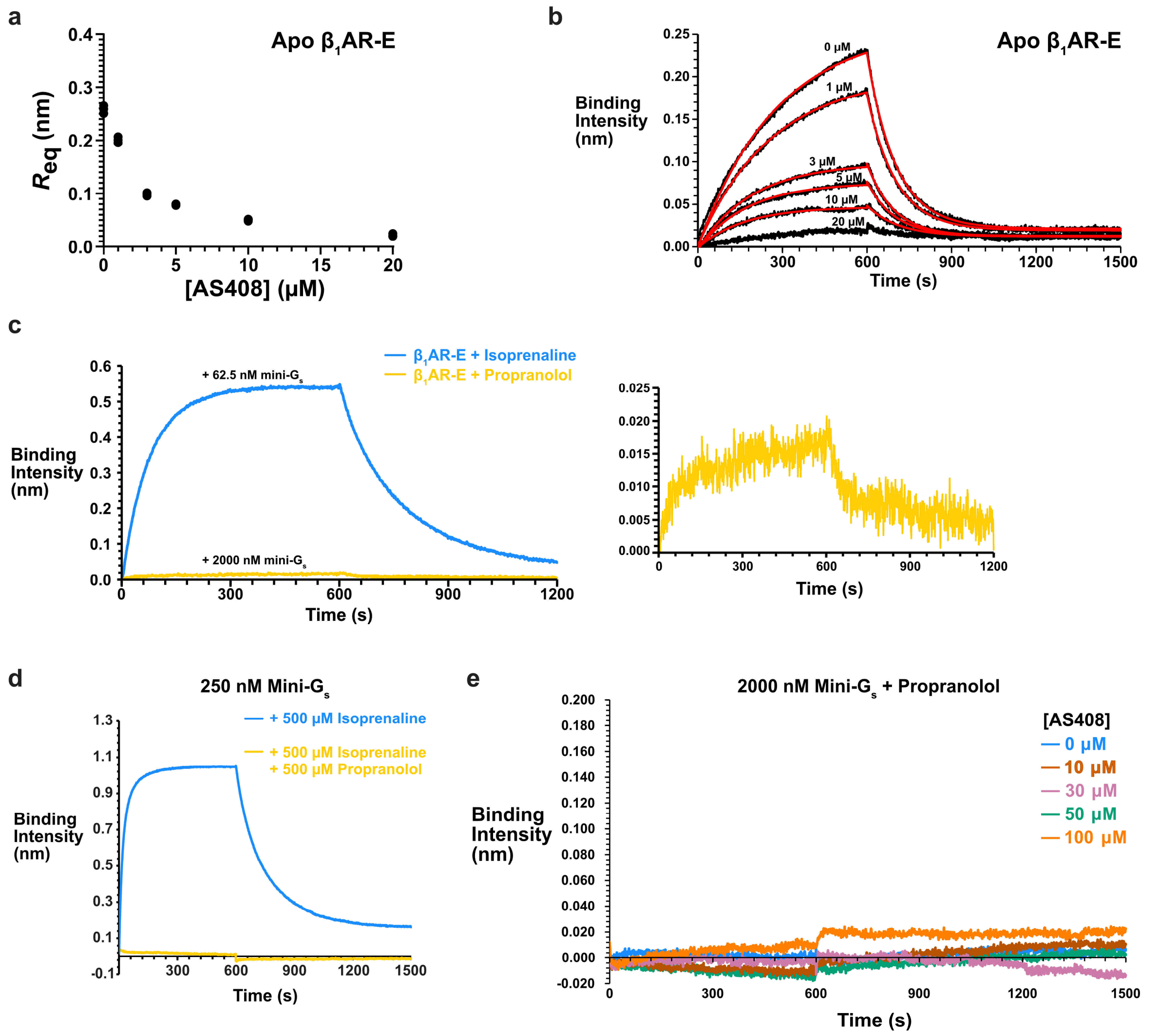

Supplementary Figure 20. AS408 has little impact on mini-G$_s$ binding to the inactive β$_1$AR-E in BLI assays. a R_{eq} of mini-G$_s$ binding to apo β$_1$AR-E modulated by AS408, measured by BLI. Values for each AS408 concentration in the titration series were calculated from single concentration (2000 nM mini-G$_s$) experiments due to low affinities in apo conditions. Data are *n*=3 independent technical replicates. **b** Representative BLI dose-response curves showing the concentration-dependent effects of AS408 on mini-G$_s$ binding to apo β$_1$AR-E. Black lines are the processed data curves, red lines are fits assuming a 1:1 interaction stoichiometry. **c** Comparisons of mini-G$_s$ coupling to β$_1$AR-E bound to either agonist isoprenaline or antagonist propranolol in BLI assays. Representative curves (*n*=3 technical replicates) of mini-G$_s$ association and dissociation are shown. β$_1$AR ligands were present in buffers at 500 μM, and the relevant mini-G$_s$ concentrations for each curve are indicated. Right, an enlargement of the curve for the binding of mini-G$_s$ to immobilised β$_1$AR-E. Very little mini-G$_s$ binding is observed to propranolol β$_1$AR-E, even at a high mini-G$_s$ analyte concentration. **d** A comparison of BLI curves for binding of mini-G$_s$ (250 μM) to immobilised β$_1$AR-E in the presence of 500 μM isoprenaline, or 500 μM isoprenaline + 500 μM propranolol. The curves demonstrate the effective competition of the high affinity antagonist for binding to β$_1$AR-E over the lower affinity isoprenaline (as shown by nanoBRET assays, Supplementary Fig. 3a) with purified receptor, and indicates high saturation of receptor with propranolol under these conditions. **e** BLI curves (representative, *n*=3 technical replicates) measuring the coupling of mini-G$_s$ to propranolol-bound (500 μM) β$_1$AR-E show that increasing AS408 has negligible impact on the protein-protein interaction. Note, the signals remain around the baseline, with minor (~ ±0.02 nm) deviations in the binding intensity, which are attributed to noise or non-specific interactions.

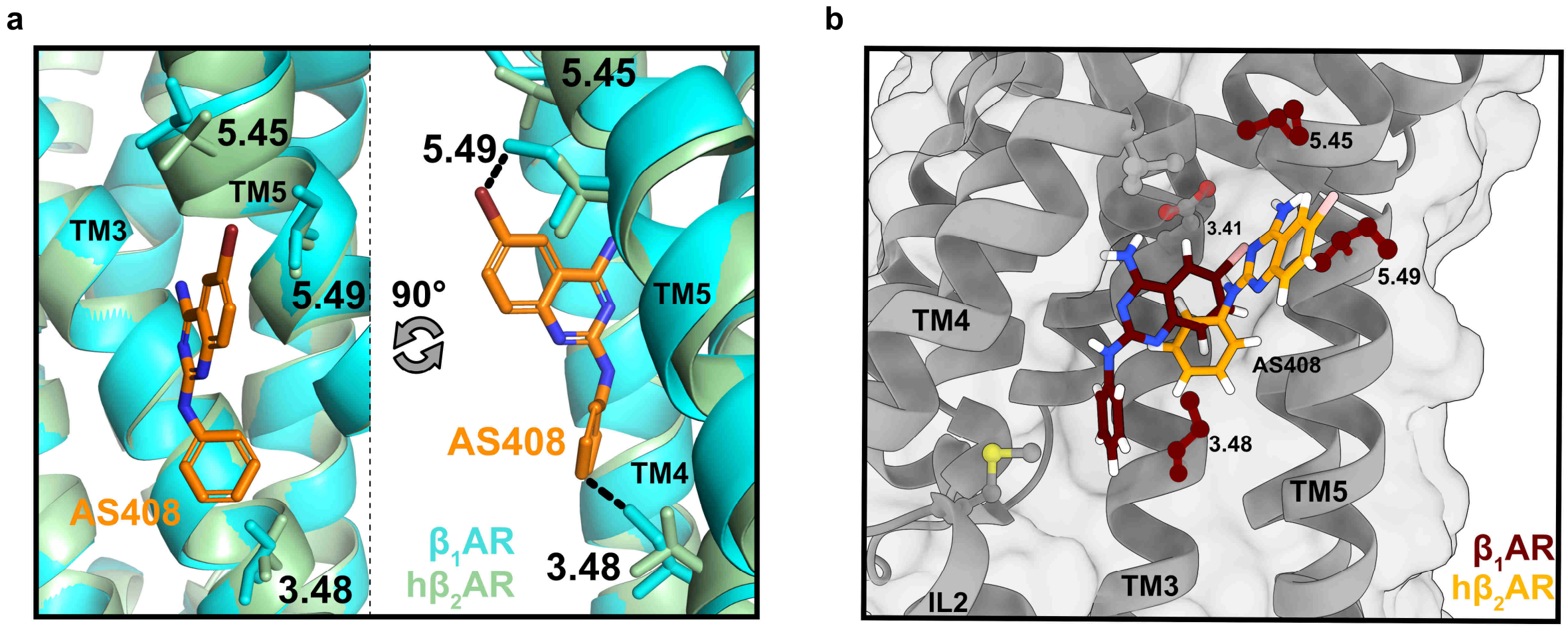

Supplementary Figure 21. Differences between the β_1AR and hβ_2AR in the TM3-TM4-TM5 AS408 binding pocket. a Structure overlay of isoprenaline-bound β_1AR crystal structure (blue, PDB ID: 2Y03)[3] and the AS408-bound hβ_2AR crystal structure (green, PDB ID: 6OBA)[10], showing the TM3-TM4-TM5 allosteric pocket into which AS408 (orange) binds at the hβ_2AR. Between the two receptors, there are three main differences in the binding pocket at positions 3.48, 5.45, and 5.49 where the shorter side chain valine residues in hβ_2AR (V129$^{3.48}$, V206$^{5.45}$, V210$^{5.49}$) consist of longer isoleucine residues in β_1AR (I137$^{3.48}$, I214$^{5.45}$, I219$^{5.49}$). The view on the right-hand side of the panel is rotated by 90° clockwise relative to the one on the left-hand side. **b** A comparison of AS408 binding mode 1 at the β_1AR (red, from MD simulations, Fig. 3e) and the binding mode of AS408 at the hβ_2AR (orange, obtained by alignment with the hβ_2AR crystal structure, PDB ID: 6OBA[10]). AS408 deviates from the hβ_2AR binding mode and interacts to a greater extent with TM4 at the β_1AR. The non-conserved 3.48, 5.45, and 5.49 positions are shown.

a

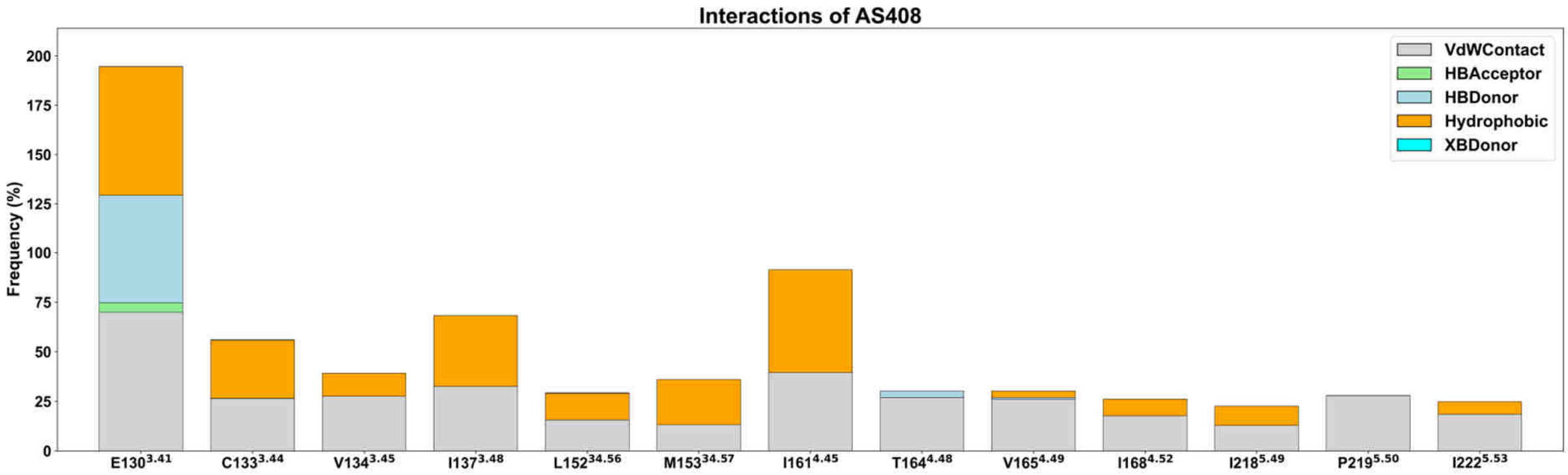

b

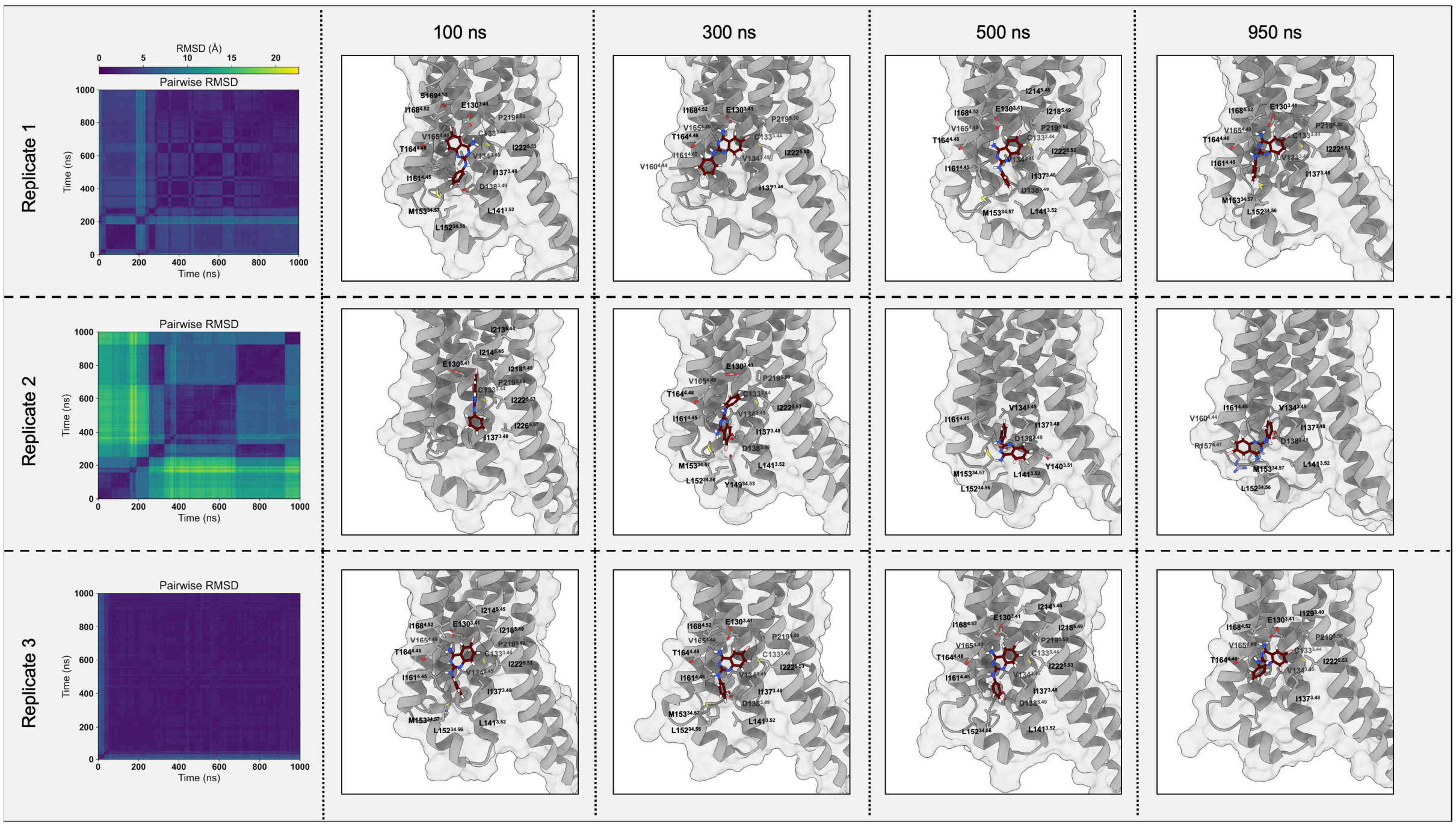

Supplementary Figure 22. The interactions and dynamics of AS408 binding to β_1AR in MD simulations. **a** All-atom simulations of isoprenaline-bound β_1AR-E with AS408 modelled into the structure were run in triplicate (*n*=3), and interactions between AS408 and residues in the TM3-TM4-TM5 allosteric pocket were measured. Stacked bars show the frequency of individual interaction types for each residue: Van der Waals contacts (grey), hydrogen bond acceptor (green), hydrogen bond donor (blue), hydrophobic (orange), and halogen bond donor (cyan). Frequencies can exceed 100% where multiple simultaneous interactions of different types occur between AS408 and a single residue. **b** Pairwise root mean square deviation (RMSD) matrices of AS408 in three replicates of isoprenaline-bound β_1AR-E, with AS408 initially modelled into the structure in the hβ_2AR orientation. Respective snapshots at different time points in each replicate are shown. Only residues within 4 Å of AS408 are shown in each frame. Replicate 2 populates AS408 Binding Pose 2, whereas Replicates 1 and 3 only populate AS408 Binding Pose 1.

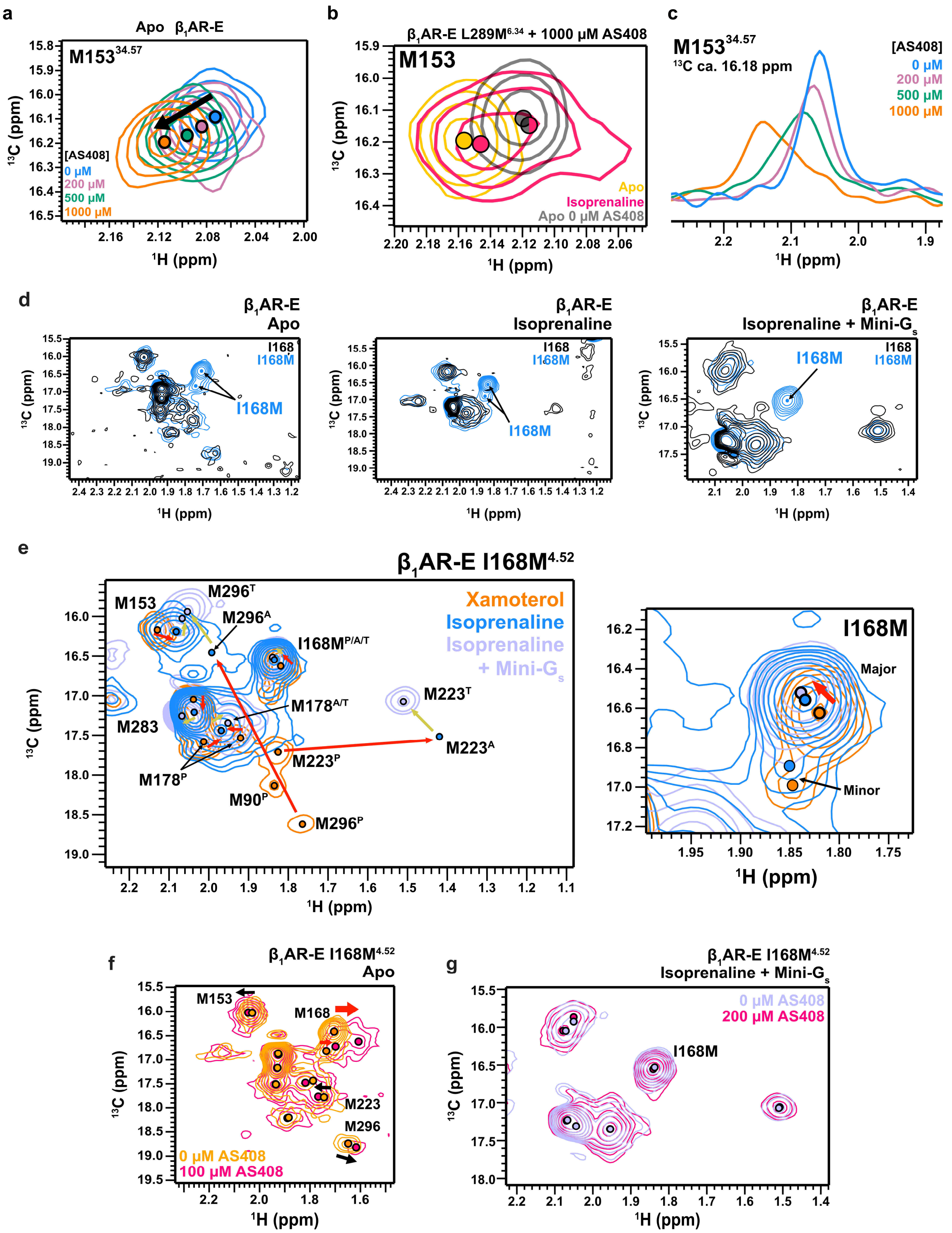

Supplementary Figure 23. Validation of the AS408 binding site at β$_1$AR-E by NMR spectroscopy. **a** An overlay of 2D ^{1}H-^{13}C HMQC NMR spectra for apo β$_1$AR-E L289M$^{6.34}$ (298 K), showing concentration-dependent chemical shift changes for M153$^{34.57}$ signals with increasing AS408 concentration. **b** An overlay of 2D ^{1}H-^{13}C HMQC NMR spectra for β$_1$AR-E L289M$^{6.34}$ (308 K) showing M153$^{34.57}$ signals for apo and isoprenaline-bound (1 mM) receptor with 1000 μM AS408. An apo (0 μM AS408) spectrum is shown to reference the inactive state signal position. The pink-and-grey circle indicates the M153$^{34.57}$ minor shoulder in the isoprenaline-bound receptor spectrum. **c** 1D traces taken from the 2D ^{1}H-^{13}C HMQC NMR spectra of

isoprenaline-bound (1 mM) β_1AR-E with increasing AS408 addition in Fig. 3g, corresponding to the ^{13}C position of the peak maxima (ca. 16.18 ppm). **d** An overlay of 2D ^{1}H-^{13}C HMQC spectra for β_1AR-E (I168$^{4.52}$) and β_1AR-E I168M$^{4.52}$ for assignment of I168M$^{4.52}$ signals. Left, apo (298 K), centre, isoprenaline-bound (1 mM, 308 K), right, isoprenaline and mini-G$_s$ (2 molar equivalents) ternary complex (308 K). **e** An overlay of 2D ^{1}H-^{13}C HMQC NMR spectra for β_1AR-E I168M$^{4.52}$ bound to xamoterol (1 mM), isoprenaline (1 mM), or the isoprenaline and mini-G$_s$ (two molar equivalents) ternary complex (308 K). Positions for pre-active state (P), active state (A), and ternary complex (T) signals are based on β_1AR-E[6]. Movements of peaks from pre-active state to active state and from active state to ternary complex are indicated with red arrows and green arrows, respectively. Right, an enlargement of I168M$^{4.52}$ signals. I168M$^{4.52}$ shows small changes, indicating this outward-facing region of TM4/TM3 undergoes little conformational rearrangements upon ligand stimulation and is insensitive to conformational rearrangements in the PIF motif during receptor activation. A minor I168M$^{4.52}$ conformation is indicated. **f** An overlay of 2D ^{1}H-^{13}C HMQC NMR spectra for apo β_1AR-E I168M$^{4.52}$ (298 K) with either 0 μM or 100 μM AS408. Black arrows indicate conformational changes; the red arrow indicates proximity effects of AS408 on I168M$^{4.52}$. **g** An overlay of 2D ^{1}H-^{13}C HMQC NMR spectra for β_1AR-E I168M$^{4.52}$ (308 K) in ternary complex with isoprenaline (1 mM) and mini-G$_s$ (two molar equivalents) and 0 μM or 200 μM AS408.

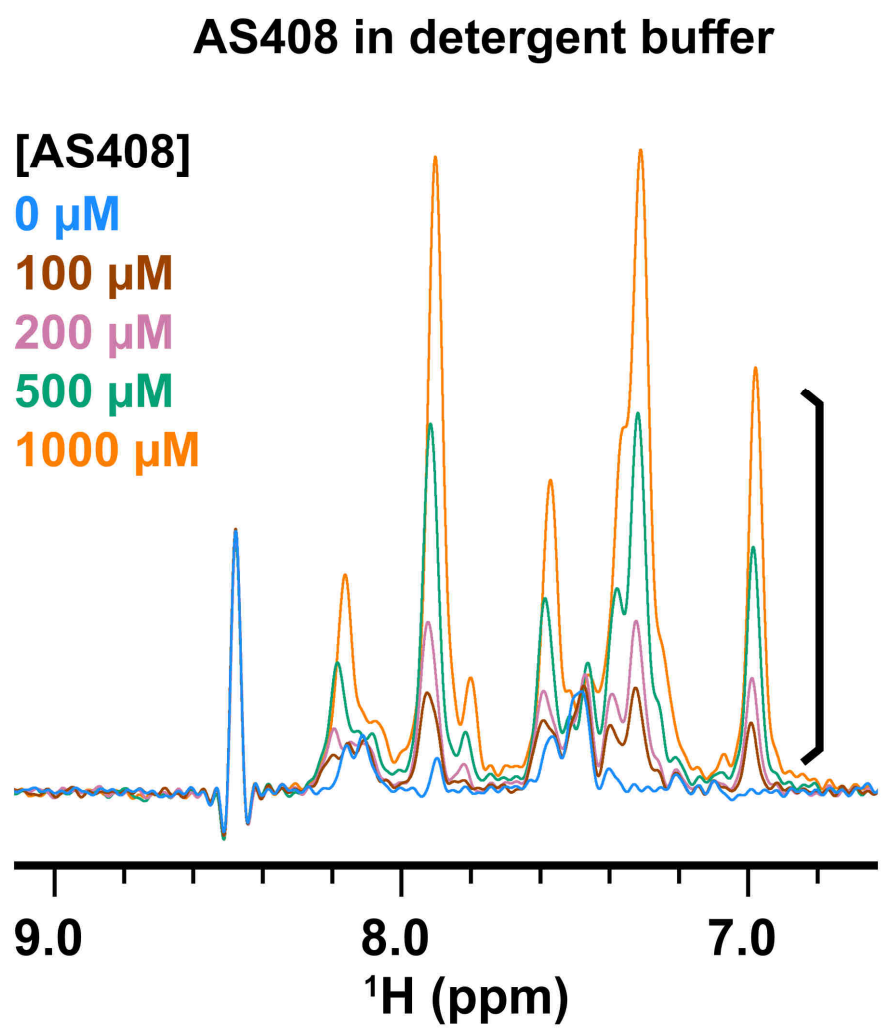

Nominal [AS408] (μM)	Scaling from ^{1}H NMR	Effective [AS408] (μM)
**100**	1.00	100
**200**	1.56	156
**500**	3.52	352
**1000**	5.79	579

Supplementary Figure 24. The effective concentrations of AS408 in NMR buffers. An overlay of 1D ^{1}H NMR spectra of AS408 in a buffer replicating β_1AR NMR conditions, to determine the amount AS408 present in the solvent under NMR conditions. The relative intensities of peaks (representative peak used for scaling indicated on the right) were measured and used to assess the concentration of AS408 in solution (estimated concentration), right. The effective concentrations in solution were reduced by at least 40% compared to the nominal concentration at 1000 μM AS408. These data suggest AS408 solubility in detergent buffers is poor relative to partitioning into lipid membranes (e.g. in TRUPATH assays) and is consistent with poor saturation of detergent-solubilised hβ_2AR (79% AS408 saturation in the AS408-hβ_2AR crystal structure)[10]. Note the assumption that 100 μM AS408 is fully solubilised.

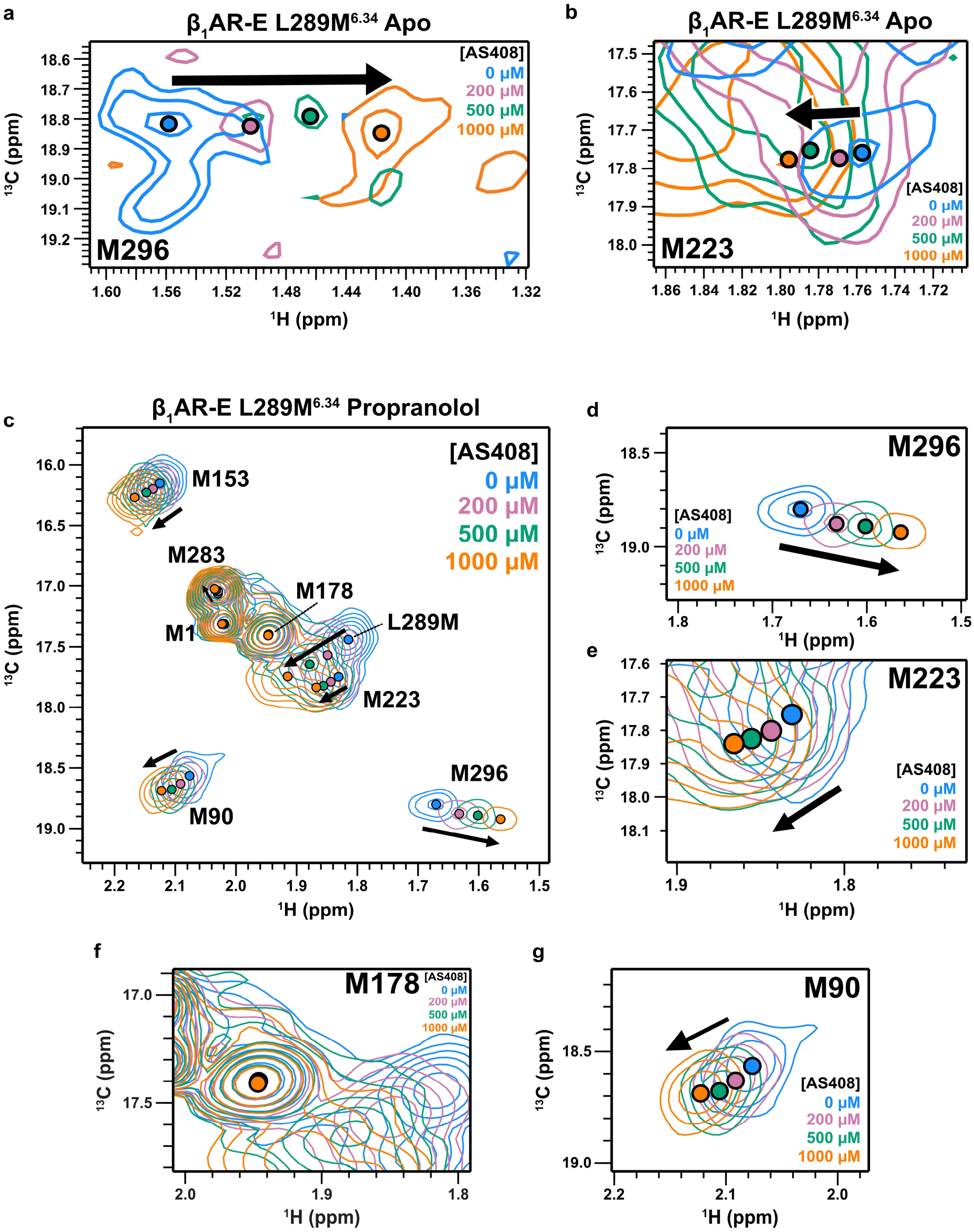

Supplementary Figure 25. The effects of AS408 on receptor core probes in ^{1}H-^{13}C NMR studies of the β_1AR inactive state. a An overlay of 2D ^{1}H-^{13}C HMQC spectra showing the M296$^{6.41}$ region for apo β_1AR-E L289M$^{6.34}$ with increasing addition of AS408. Due to the lower stability of the apo state the spectra were recorded at 298 K. Black arrows show the direction of chemical shift change with increasing AS408 concentration. **b** An overlay of 2D ^{1}H-^{13}C HMQC spectra showing the M223$^{5.54}$ region for apo β_1AR-E L289M$^{6.34}$ with increasing addition of AS408, recorded at 298 K. **c** An overlay of 2D ^{1}H-^{13}C HMQC spectra of β_1AR-E L289M$^{6.34}$ bound to the antagonist propranolol (1 mM), with increasing AS408 addition. Spectra were recorded at 308 K. **d**-**g** Enlargements of the spectra in (**c**), showing the regions associated with signals for **d** M296$^{6.41}$, **e** M223$^{5.54}$, **f** M178$^{4.62}$, and **g** M90$^{2.53}$.

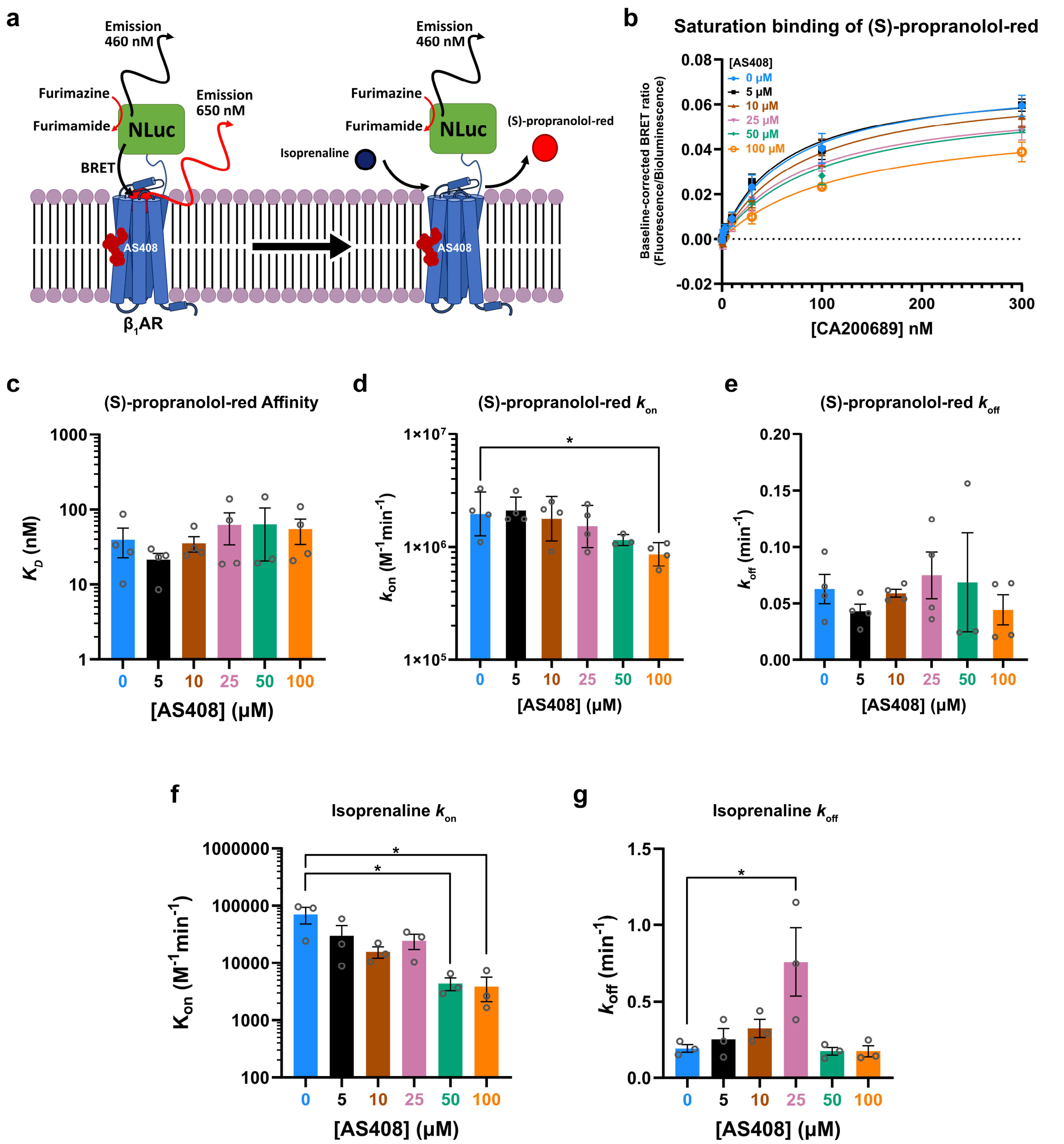

Supplementary Figure 26. The effect of AS408 on isoprenaline affinity to β$_1$AR-E. a A schematic describing the nanoBRET competition binding assay assessing the impact of AS408 on agonist binding to N-terminally tagged Nluc-β$_1$AR-E. The fluorescent tracer was β$_1$AR antagonist (S)-propranolol-red, which can be displaced by isoprenaline. **b** Saturation equilibrium curves for binding of (S)-propranolol-red (CA200689) to β$_1$AR-E with increasing AS408 concentration, measured in HEK293T cells. Data are mean ± SEM of *n*=4 (*n*=3 for [AS408] = 50 µM) independent biological replicates. Minor AS408 compound interference is observed at the maximum AS408 concentration (100 µM). **c** K_D values for (S)-propranolol-red binding to β$_1$AR-E with varying AS408 concentration, measured by the nanoBRET assay in (**a**) expressed in HEK293T cells. Data are mean ± SEM of *n*=4 (*n*=3 for [AS408] = 50 µM) independent biological experiments. **d** Association rate constants of (S)-propranolol-red binding to β$_1$AR-E with increasing AS408 concentration, in HEK293T cells. * indicates significance. Data are mean ± SEM of *n*=4 (*n*=3 for [AS408] = 50 µM) independent biological replicates. **e** Dissociation rate constants of (S)-propranolol-red binding to β$_1$AR-E with increasing AS408 concentration, in HEK293T cells. Data are mean ± SEM of *n*=4 (*n*=3

for [AS408] = 50 μM) independent biological replicates. **f** Association rate constants of isoprenaline binding to β_1AR-E with increasing AS408 concentration, in HEK293T cells. Data are mean ± SEM of n=3 independent biological experiments. **g** Dissociation rate constants of isoprenaline binding to β_1AR-E with increasing AS408 concentration, in HEK293T cells. Data are mean ± SEM of n=3 independent biological replicates. Data in (**c-g**) were analysed by one-way ANOVA with Dunnet's post-hoc tests, on log-transformed data. No significant differences were observed in (**c**) or (**e**) (p>0.05). Significance was only observed for propranolol association (**d**) at the highest AS408 concentration (0 μM AS408 vs 100 μM AS408: p = 0.0201). In (**f**), significant decreases in isoprenaline association rate constants were observed (0 μM AS408 vs 50 μM AS408: p = 0.0019; 0 μM AS408 vs 100 μM AS408: p = 0.0009). In (**g**), dissociation rate constants generally did not significantly change, aside from 25 μM AS408 (0 μM AS408 vs 25 μM AS408: p = 0.0070).

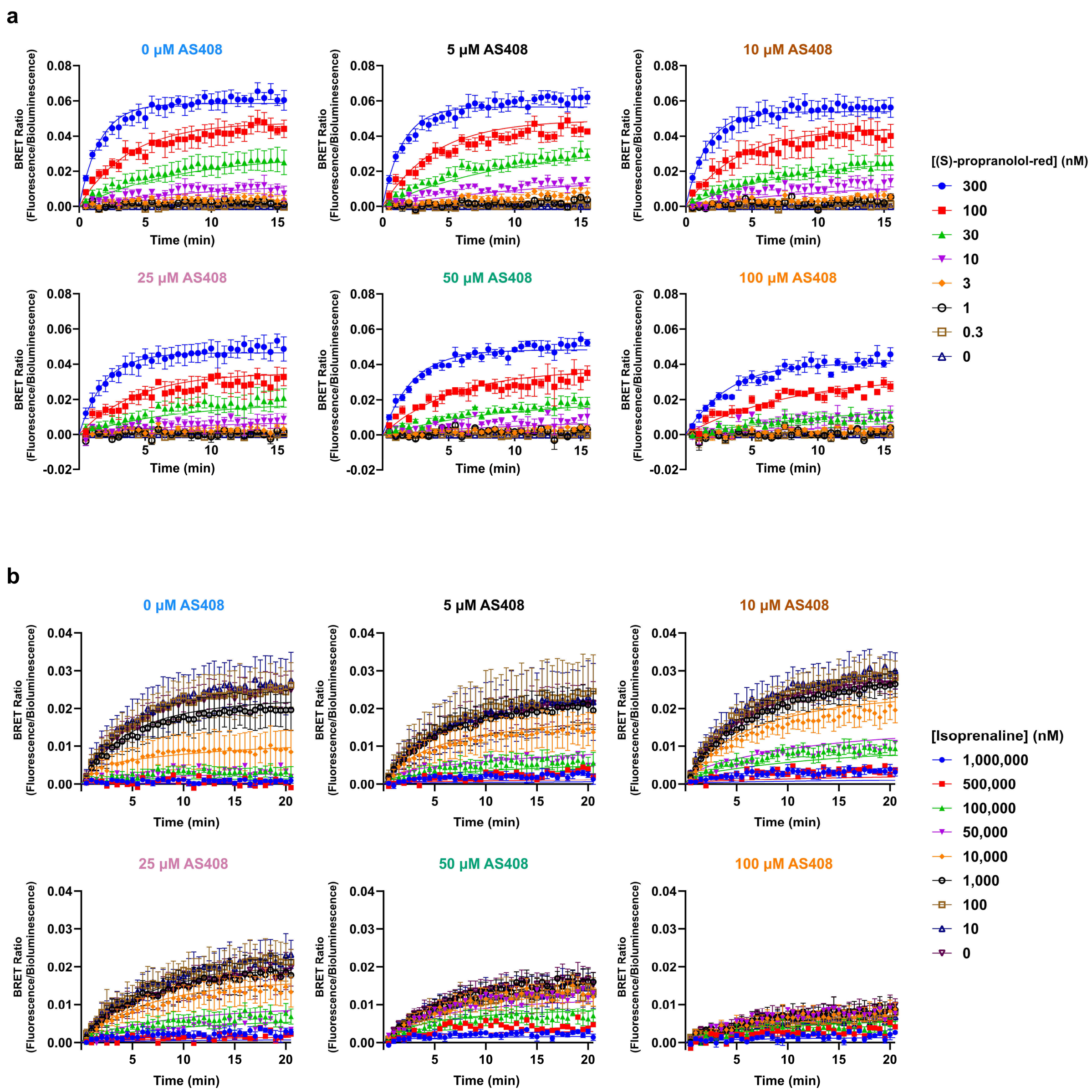

Supplementary Figure 27. Measurements of ligand binding kinetics to β_1AR-E with increasing AS408 concentration. a Kinetic traces for the saturation equilibrium measurements of the fluorescently labelled β_1AR antagonist (S)-propranolol-red to β_1AR-E in HEK293T cells incubated with increasing concentrations of AS408. Measurements were performed using the nanoBRET-based assay shown in Supplementary Fig. 3a. Data are the mean ± SEM of *n*=4 independent biological replicates. **b** Kinetic traces for the competition equilibrium measurements of isoprenaline binding to β_1AR-E in HEK293T cells incubated with increasing concentrations of AS408. Measurements were performed using the nanoBRET-based assay shown in Supplementary Fig. 3a by competition of isoprenaline with (S)-propranolol-red. Data are the mean ± SEM of *n*=3 independent biological replicates.

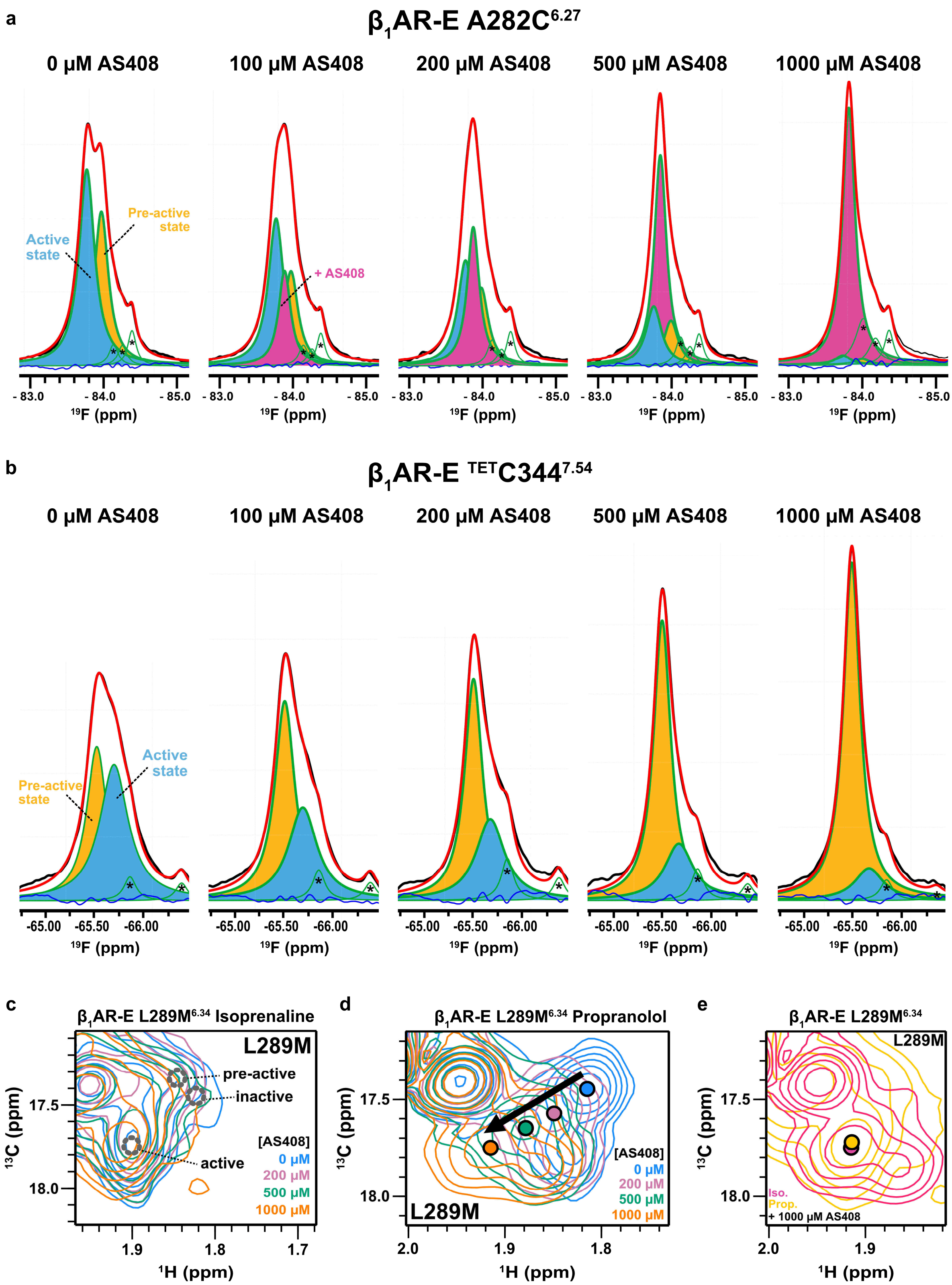

Supplementary Figure 28. Deconvolution of ^{19}F NMR spectra for AS408-bound β_1AR-E. a Spectral deconvolutions of 1D ^{19}F NMR spectra for isoprenaline-bound (1 mM) β_1AR-E A282C$^{6.27,BTFA}$ (298 K) with increasing AS408 concentrations (in Fig. 5a). The active state (teal), pre-active state (orange), and AS408-stabilised alternative conformation (+AS408, pink) are indicated. The residual (blue line) is the subtraction of the sum (red line) of the simulated peaks (green lines) from the raw data (black line).

* indicate degradation products. The AS408-bound alternative conformation signal was simulated with a smaller linewidth (95 Hz) than the signals of either the pre-active state (125 Hz) or active state (143 Hz). **b** Spectral deconvolutions of 1D ^{19}F NMR spectra for isoprenaline-bound (1 mM) β_1AR-E $^{TET}C344^{7.54}$ (298 K) with increasing AS408 concentrations (in Fig. 5b). The linewidth used to simulate the pre-active state signal decreased with increasing AS408 addition, from 160 Hz (0 µM AS408) to 120 Hz (1000 µM AS408). **c** An overlay of 2D ^{1}H-^{13}C HMQC spectra for the isoprenaline-bound (1 mM) β_1AR-E $L289M^{6.34}$ (308 K) with increasing AS408 concentration, showing an enlargement of the $L289M^{6.34}$ region. Positions of the pre-active state (a minor population present for isoprenaline-bound β_1AR-E) and the inactive state (obtained from the apo spectrum of β_1AR-E $L289M^{6.34}$) are marked with grey dashed circles. There is a decrease in minor pre-active state signal with increasing AS408 concentration. **d** An overlay of 2D ^{1}H-^{13}C HMQC spectra for propranolol-bound (1 mM) β_1AR-E $L289M^{6.34}$ (308 K) with increasing AS408 concentration (from Supplementary Fig. 25c), showing an enlargement of the $L289M^{6.34}$ region. **e** An overlay of 2D ^{1}H-^{13}C HMQC NMR spectra for isoprenaline-bound (Iso., 1 mM) and propranolol-bound (Prop., 1 mM) β_1AR-E $L289M^{6.34}$, respectively, with 1000 µM AS408, showing the similarity of the $L289M^{6.34}$ signal. Spectra were recorded at 308K.

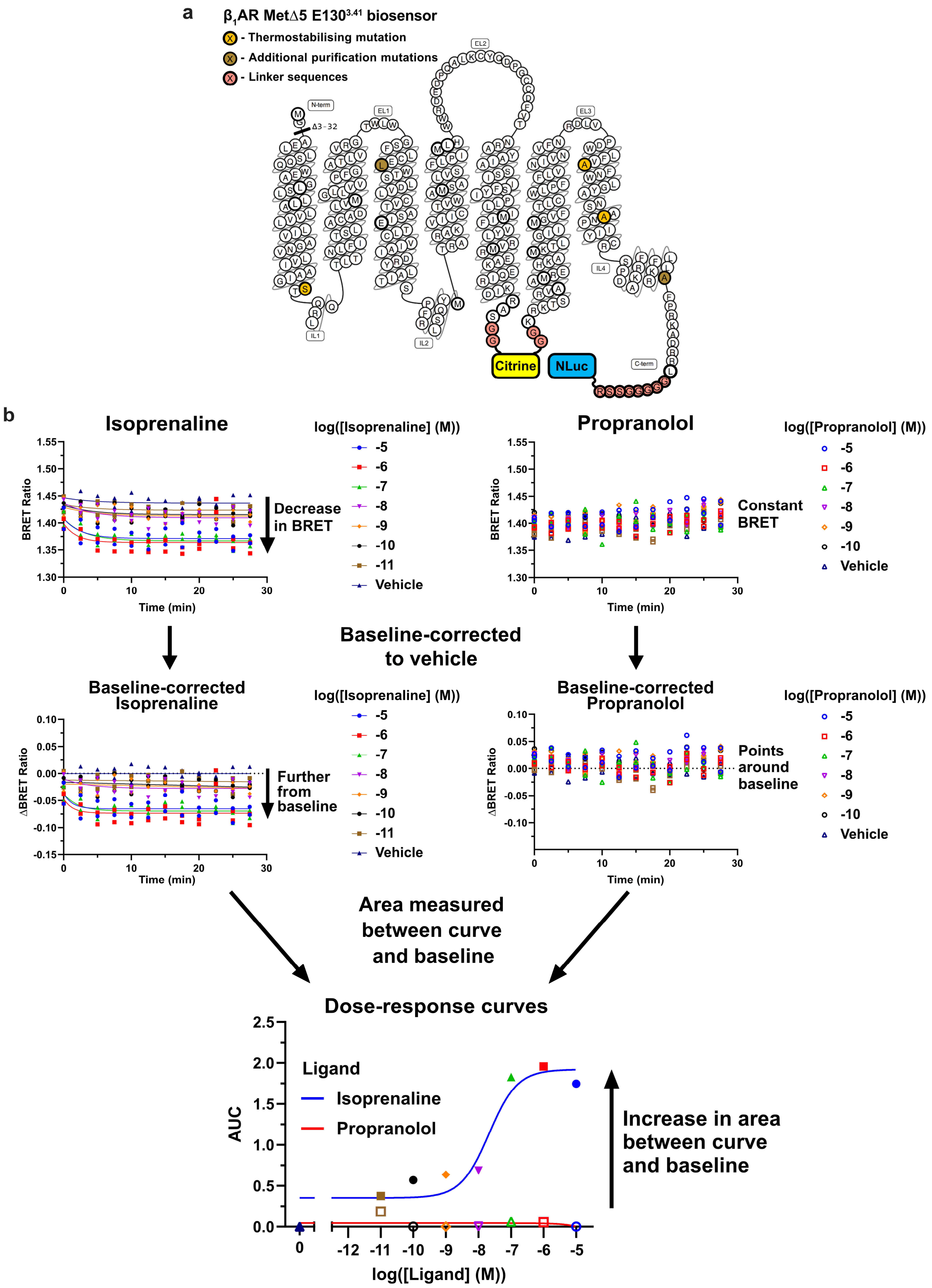

Supplementary Figure 29. Development of a BRET-based conformational biosensor for the β$_1$AR-E. **a** A snake diagram of the β$_1$AR-E construct used to generate the BRET-conformational biosensor. Using the β$_1$AR-E (Supplementary Fig. 1a) as a background, Citrine was inserted into IL3 and Nluc added to the end of a truncated C-terminus. Additional linker sequences (GG) were also added either side of the citrine insertion, and prior to the Nluc tag (GGGGGSSR), shown in light red. Note, the biosensor construct contained the same thermostabilising mutations as in β$_1$AR-E. **b** Validation of the β$_1$AR-E BRET biosensor in reporting agonist-dependent activation (isoprenaline) but not antagonist activity (propranolol). Data are

n=2 biological replicates, performed in duplicate. The biosensor was stably expressed in HEK293A cells, which were stimulated with increasing doses of either ligand. Changes in BRET ratios were detected at each concentration of ligand, and values were then normalised to the baseline for the vehicle. The area under the curves (AUC) were calculated over the 28-minute stimulation window. Concentration response curves were then fitted to examine ligand-dependent conformational changes. Data were fit using the 3 parameter logistic equations. Increases in AUC were observed for agonist stimulation, whereas propranolol had little effect on the AUC. Therefore, the β_1AR-E BRET biosensor reports on changes in receptor conformations upon agonist activation.

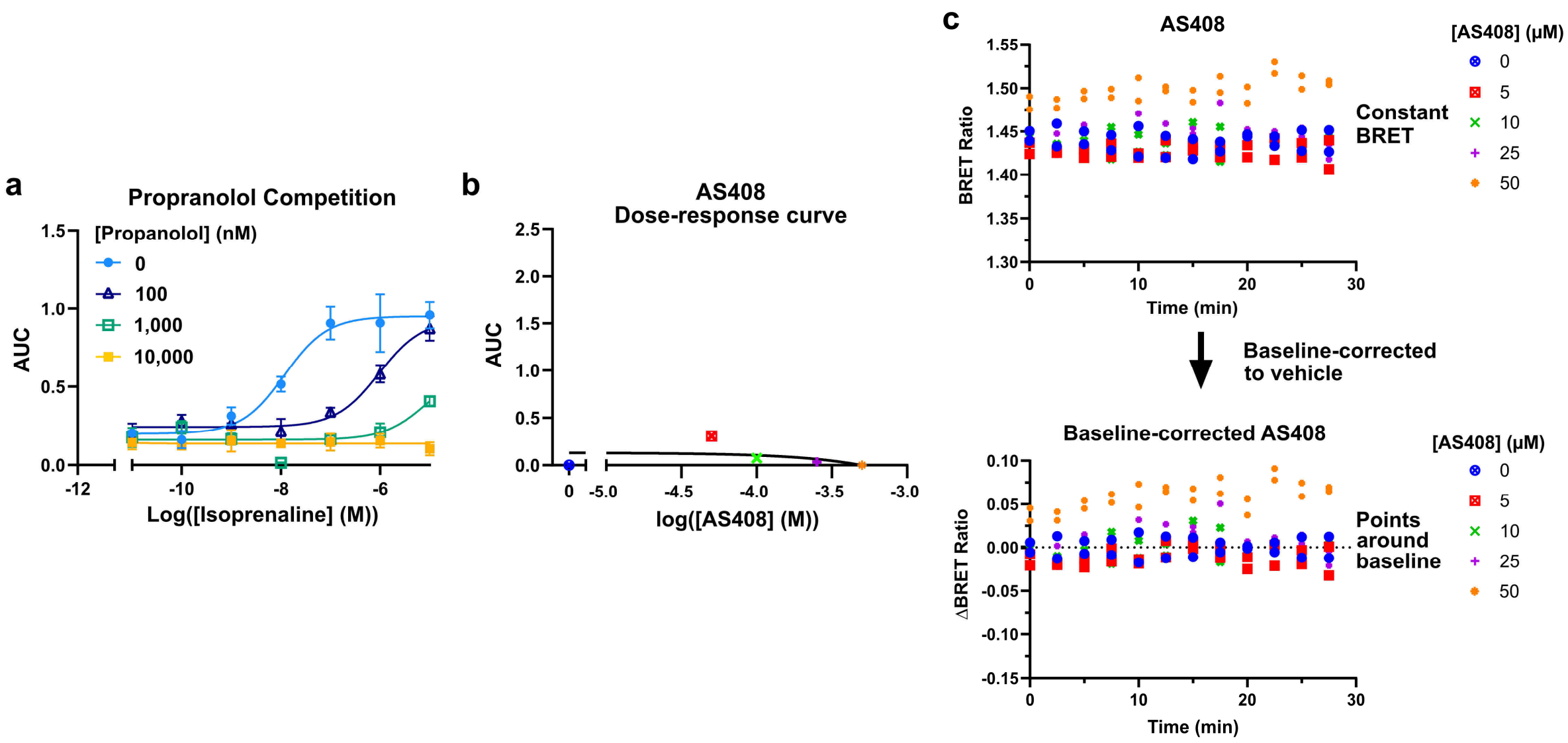

Supplementary Figure 30. Validation of the BRET-based β_1AR-E conformational biosensor for investigating ligand and allosteric modulator related conformational changes. a Validation of the β_1AR-E BRET-sensor by agonist-antagonist competition. Concentration-response curves were recorded for isoprenaline stimulation of the β_1AR-E BRET-sensor in HEK293A cells, with increasing addition of propranolol. Data are the mean ± SEM of *n*=3 biological replicates. **b** Validation of the β_1AR-E BRET-sensor with respect to AS408-induced conformational changes. The β_1AR-E BRET-sensor was stimulated with increasing doses of AS408 in HEK293A cells (*n*=3, recorded in duplicate). The concentration response curve demonstrates little conformational changes detected by the BRET-sensor upon addition of AS408 alone. **c** Changes in BRET ratios in the BRET-sensor for increasing addition of AS408. Data are *n*=2 biological replicates. BRET values were normalised to the baseline for the vehicle, and the area under the curve were measured to generate the concentration response curve in (**b**). We note that minor AS408 compound interference is observed at the maximum AS408 concentration (50 μM), which causes a small shift in values, but this effect is negligible below this concentration.

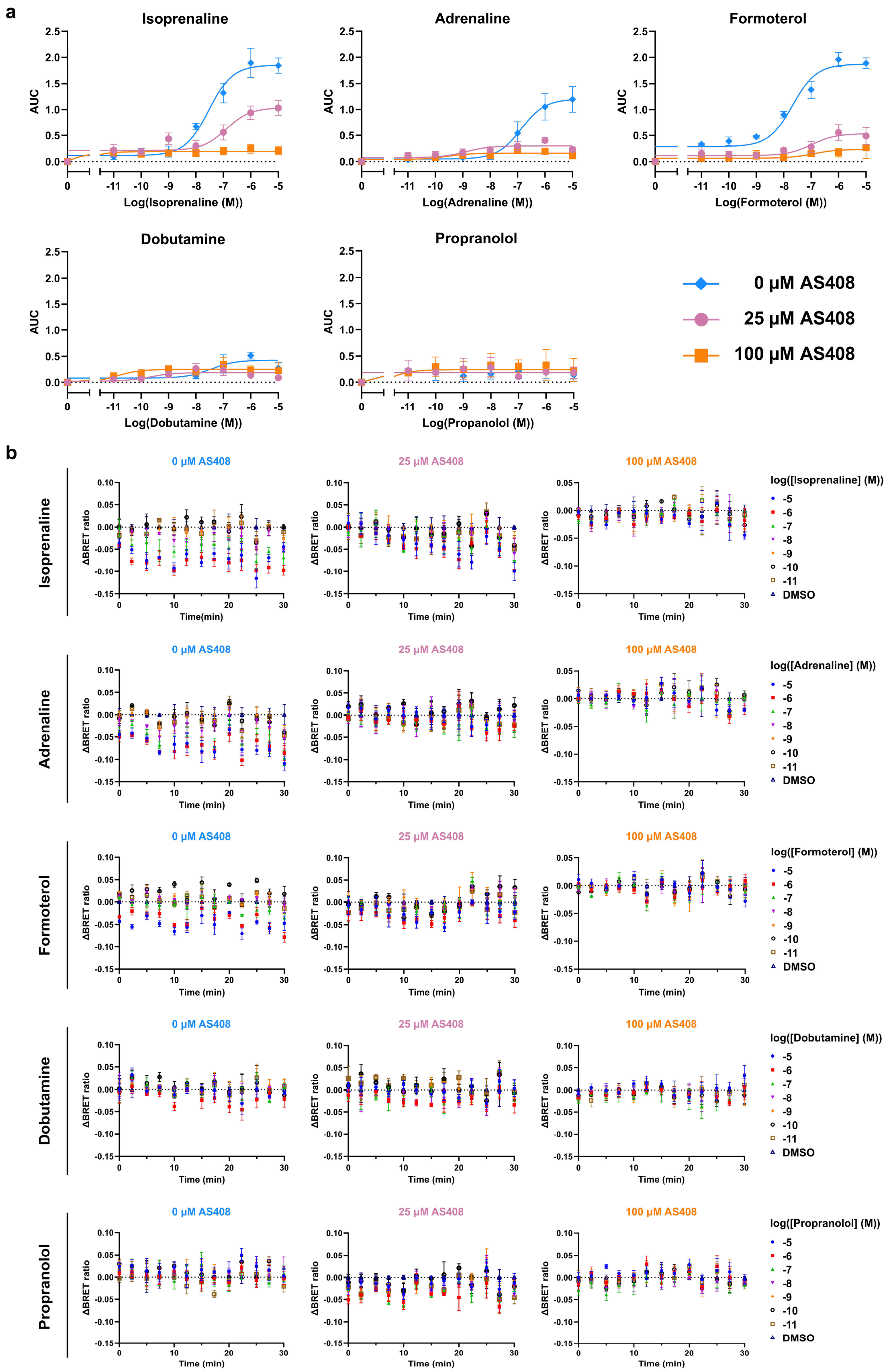

Supplementary Figure 31. The β$_1$AR-E BRET-sensor indicates there is no probe dependence for AS408 at the β$_1$AR stimulated with non-biased orthosteric ligands. a The concentration response curves for the β$_1$AR-E BRET-sensor when co-stimulated with AS408 and different G protein/β-arrestin non-biased

orthosteric ligands. Curves are shown for full agonists isoprenaline, adrenaline, and formoterol, the partial agonist dobutamine, and the antagonist propranolol. Data are the mean ± SEM of n=3 independent biological replicates, measured in HEK293A cells. Data are fitted using the 3 parameter logistic equations. **b** The kinetic traces for the BRET-sensor data shown in (**a**).

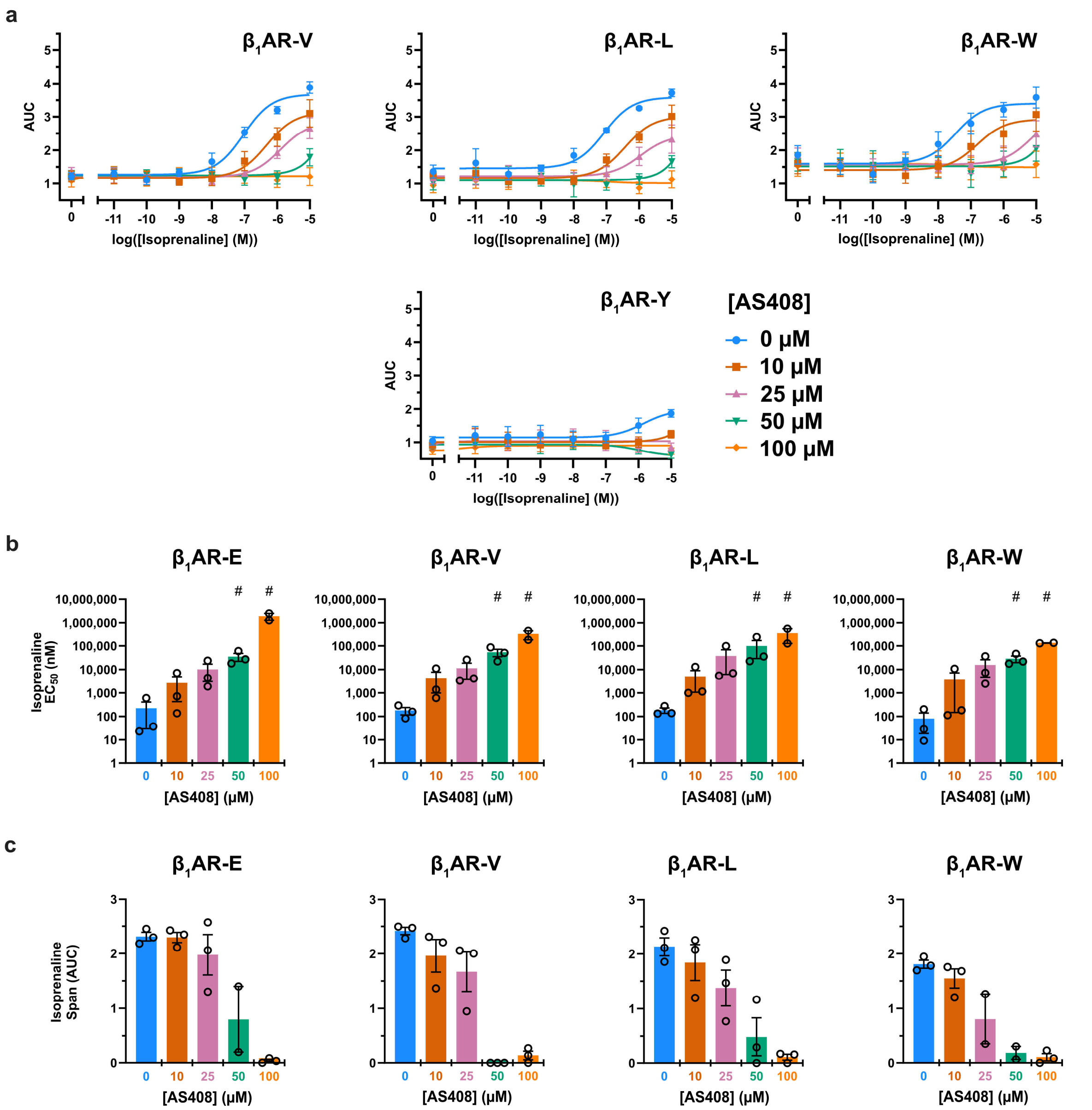

Supplementary Figure 32. β_1AR E130X$^{3.41}$ mutations have no significant impact on the effects of AS408 on β_1AR signalling in TRUPATH assays. a TRUPATH assays for binding of G_s proteins to β_1AR E130X$^{3.41}$ variants, modulated by AS408. *In cell* assays in HEK293T cells measured the dissociation of the Gβγ subunits from Gα. Responses for each AS408 concentration were measured for varying isoprenaline concentrations and data fitted using the 3 parameter logistic equations. Data are mean ± SEM of *n*=3 independent biological experiments, each performed in duplicate. **b** The effects of AS408 concentration on isoprenaline EC_{50} values, calculated for E130X$^{3.41}$ mutants from TRUPATH assay data in (**a**) and Fig. 3a (β_1AR-E). # indicates EC_{50} values taken from graphs with incomplete fits. **c** The effects of AS408 concentration on isoprenaline span, calculated for E130X$^{3.41}$ mutants from TRUPATH assay data in (**a**) and Fig. 3a (β_1AR-E).

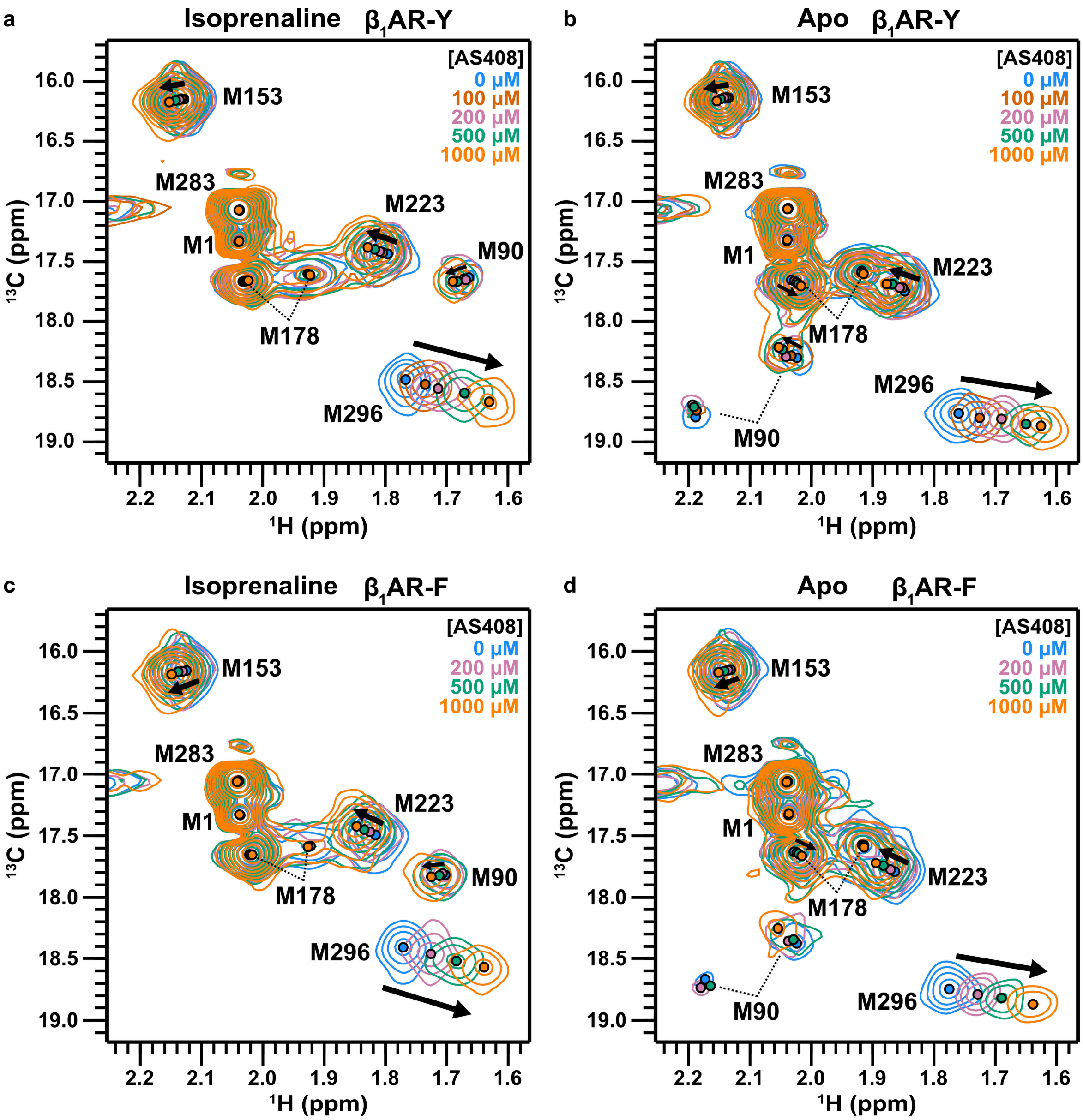

Supplementary Figure 33. The effects of AS408 on the conformational equilibria of β$_1$AR-F and β$_1$AR-Y. 2D ^{1}H-^{13}C HMQC NMR spectra for β$_1$AR E130F/Y$^{3.41}$ variants with increasing AS408 concentration, recorded at 308 K. **a** Isoprenaline-bound (1 mM) β$_1$AR-Y, **b** apo β$_1$AR-Y, **c** isoprenaline-bound (1 mM) β$_1$AR-F, **d** apo β$_1$AR-F. Black arrows indicate chemical shift changes with increasing AS408 concentration.

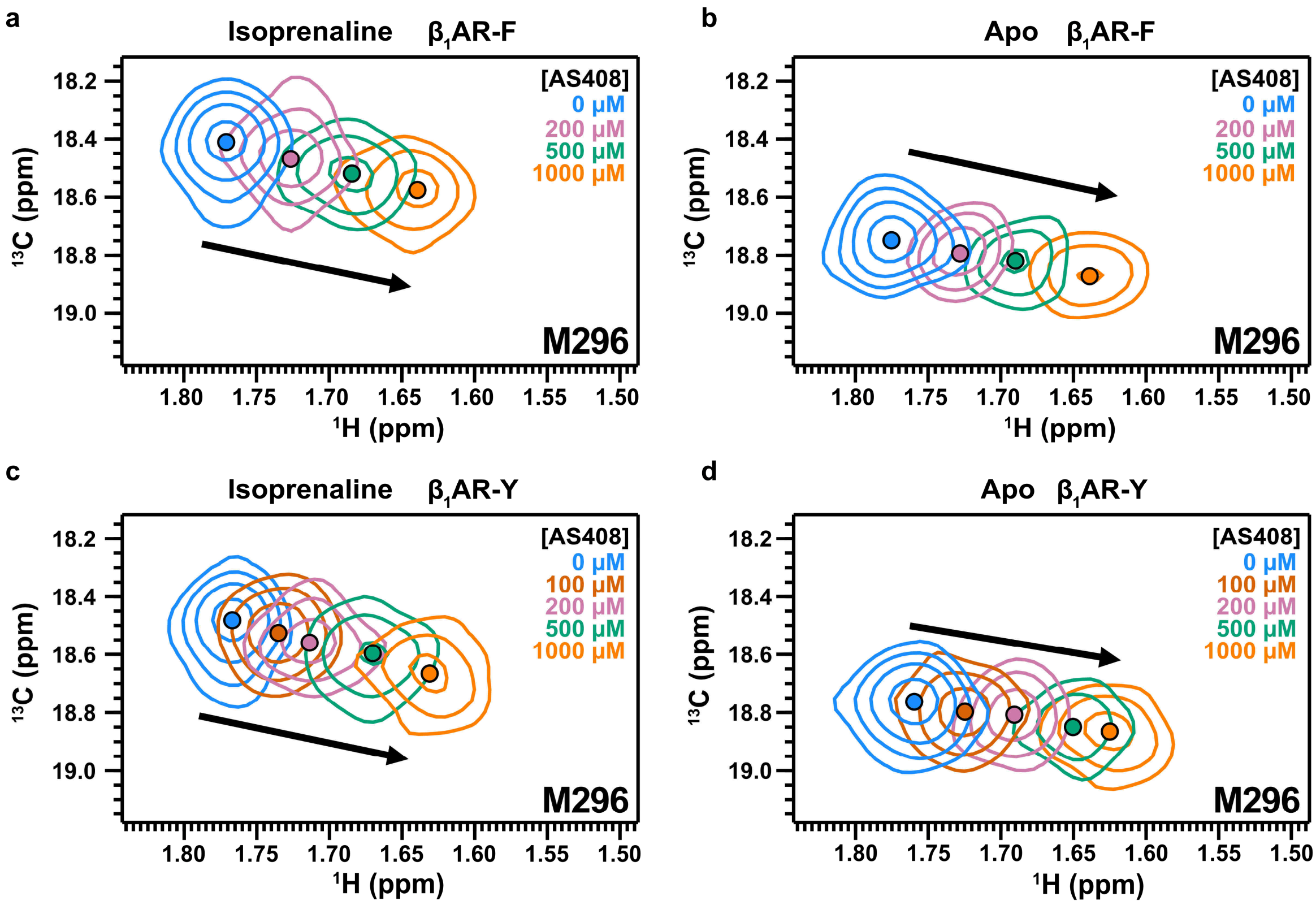

Supplementary Figure 34. Chemical shift changes in M296$^{6.41}$ upon addition of AS408 in β$_1$AR-F and β$_1$AR-Y. 2D ^{1}H-^{13}C HMQC NMR spectra for β$_1$AR E130F/Y$^{3.41}$ variants with increasing AS408 concentration, showing the M296$^{6.41}$ probe signals. Spectra were recorded at 308 K. **a** isoprenaline-bound (1 mM) β$_1$AR-F, **b** apo β$_1$AR-F, **c** isoprenaline-bound (1 mM) β$_1$AR-Y, **d** apo β$_1$AR-Y. Black arrows indicate chemical shift changes with increasing AS408 concentration.

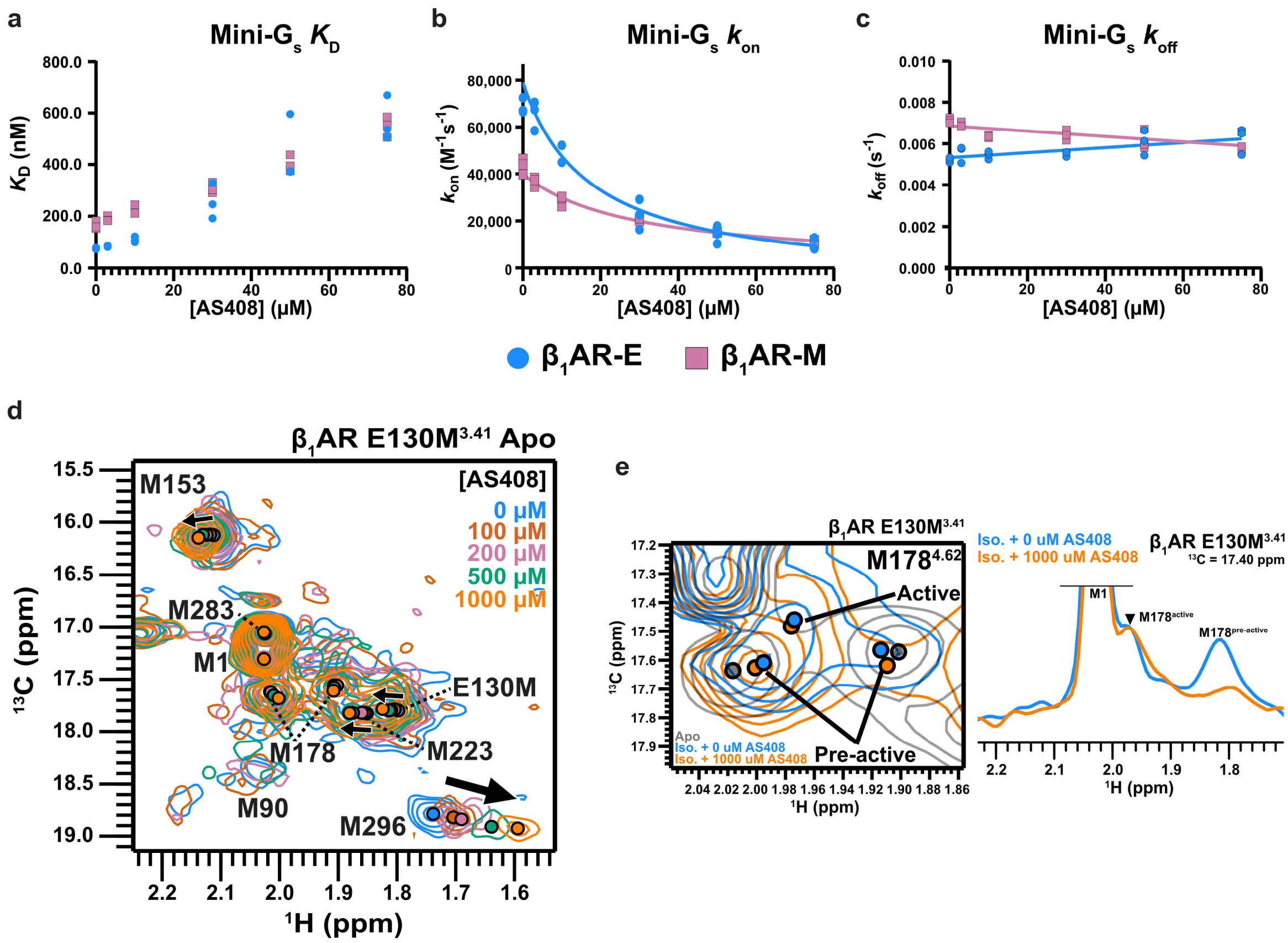

Supplementary Figure 35. The effects of AS408 on the conformational equilibria of β_1AR-M. **a-c** The binding kinetics parameters from BLI dose-response assays examining the effects of AS408 on mini-G_s interactions with isoprenaline-bound (500 μM) β_1AR-M compared to isoprenaline-bound β_1AR-E. Data are calculated from dose-response experiments with n=3 independent technical replicates. Parameters for β_1AR-M and β_1AR-E tend towards similar values with increasing AS408 concentration. **a** K_D. **b** k_{on}, data are fit with [Inhibitor] vs Response (three parameter variable slope), with a Hill constant fixed at -1. **c** k_{off} (as a guide, a linear fit through the data is shown). **d** An overlay of 2D ^{1}H-^{13}C HMQC NMR spectra for apo β_1AR-M with increasing AS408 concentration, recorded at 308 K. Black arrows indicate chemical shift changes with increasing AS408 concentration. Note, the M296$^{6.41}$ signal decreases in intensity at saturating AS408 concentration, similar to isoprenaline-bound β_1AR-Y/β_1AR-F (Supplementary Fig. 34a,c), whereas isoprenaline-bound β_1AR-M shows an increase in signal intensity (Fig. 7c). Thus, the inactive state becomes more dynamic in the receptor core when bound by AS408, and the pre-active state becomes less dynamic. **e** An overlay of spectra for isoprenaline-bound (Iso., 1 mM) β_1AR-M showing the M178$^{4.62}$ probe region for spectra with 0 μM and 1000 μM AS408 added, recorded at 308 K. Left, 2D ^{1}H-^{13}C HMQC NMR spectra. The apo spectrum for β_1AR-M is shown in faded grey for reference. Right, 1D ^{1}H slices taken from the 2D spectrum at the ^{13}C chemical shift position 17.40 ppm.

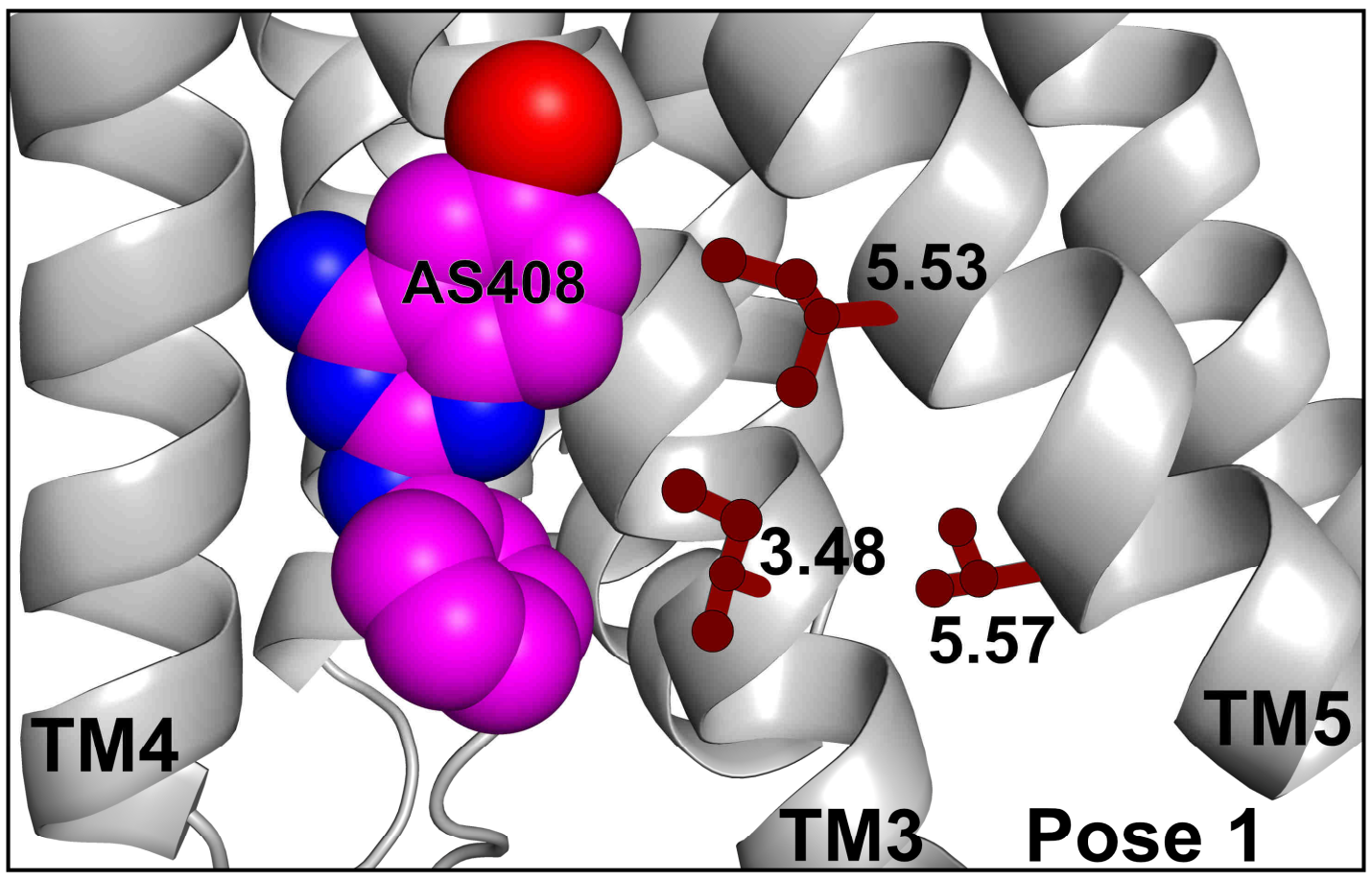

Supplementary Figure 36. The proximity of AS408 binding pose 1 to a dynamic region of TM5. Binding pose 1 of AS408 at the β_1AR (from MD simulations), showing the relative positions of highly dynamic backbone regions of TM5 and TM3 observed in the ^{15}N NMR study by Wu *et al.*, 2025[11]. The AS408 molecule is shown in purple (Br atom in red, N atoms in blue). The side chains of residues which were noted to have high backbone dynamics in the ^{15}N study, shown in dark red, are in close proximity to AS408. This interaction might impact the mechanism by which AS408 modulates β_1AR dynamics, by communicating information to the intracellular surface through the plastic region which is susceptible to modulation and thereby stabilising the alternative conformations observed for NMR probes in this study. Notably, these interactions are present in the region below the PIF motif, and thus this may explain why the PIF motif remains dynamic in the AS408-bound receptor (as reported by NMR probes, Fig. 7).

Supplementary Table 1. The binding kinetics parameters for isoprenaline-stimulated mini-G_s binding to different β_1AR E130X$^{3.41}$ receptor constructs. Data are for dose-response BLI measurements in Fig. 1f and Supplementary Fig. 7, and are the mean ± SD of three independent technical replicates.

β_1AR E130X$^{3.41}$	k_{on} ($M^{-1}s^{-1}$)	k_{off} (s^{-1})	K_D (nM)
**β_1AR-E**	7.10x10^4 ± 3.98x10^3	5.09x10^{-3} ± 0.72x10^{-4}	71.76 ± 2.99
**β_1AR-V**	4.93x10^4 ± 2.72x10^3	5.28x10^{-3} ± 0.07x10^{-4}	107.27 ± 5.63
**β_1AR-M**	4.91x10^4 ± 3.37x10^3	6.95x10^{-3} ± 1.48x10^{-4}	142.13 ± 12.27
**β_1AR-I**	4.75x10^4 ± 8.99x10^3	6.54x10^{-3} ± 7.92x10^{-4}	138.80 ± 10.83
**β_1AR-L**	3.54x10^4 ± 3.28x10^3	6.31x10^{-3} ± 3.88x10^{-4}	179.77 ± 29.07
**β_1AR-W**	4.03x10^4 ± 2.65x10^3	8.03x10^{-3} ± 2.88x10^{-4}	199.83 ± 13.28
**β_1AR-F**	0.84x10^4 ± 0.12x10^3	9.65x10^{-3} ± 0.73x10^{-4}	1153.69 ± 9.74
**β_1AR-Y**	0.32x10^4 ± 0.34x10^3	10.66x10^{-3} ± 2.08x10^{-4}	5180.91 ± 616.09

Supplementary Table 2. The allosteric parameter values describing the effects of AS408 in isoprenaline-stimulated signalling assays at the β_1AR. Data from isoprenaline concentration response curves in the presence of varying concentrations of AS408 (10, 25, 50, and 100 μM) for the TRUPATH BRET2 biosensor G_s (Fig. 3a) and (10, 25, 50 μM) for the β_1AR-E BRET conformational sensor (Fig. 6c) were fitted using the complete operational model of allostery[12] using GraphPad Prism 10.6.1 (GraphPad, San Diego, CA). Maximal responses were fixed, according to the specific assays, the Hill coefficient was set to 1.0 and τ_B was fixed at zero with the assumption that AS408 is a NAM for agonists. This approach followed the guidelines established for the study of AS408 at the hβ_2AR (see Li *et al.*, 2020[10]).

**Complete Operational Model of Allostery***	**TRUPATH biosensor (G_s)**	**β_1AR-E BRET conformational sensor**
Best-fit values		
Log (K_A)	-7.3 ± 0.4	-7.7 ± 0.6
Log (K_B)	-6.1 ± 0.2	-6.2 ± 0.4
Log (α)	-1.5 ± 0.4	-1.4 ± 0.4
Log (β)	-1.7 ± 0.3	-1.2 ± 0.5
Log ($\alpha\beta$)	-3.2 ± 0.5	-2.6 ± 0.6

* Allostery values for isoprenaline-stimulated TRUPATH BRET2 biosensor G_s and β_1AR-E BRET conformational sensor were determined using the complete operational model of allostery (GraphPad Prism v10.6.1). Maximal values were held constant within each assay, the Hill coefficient was set to 1.0, and τ_B was fixed at ~0 since AS408 is a NAM and has no agonist efficacy[10].

Supplementary Table 3. The influence of AS408 on the affinities of isoprenaline to β_1AR-E. The affinity of isoprenaline to N-terminally tagged Nluc-β_1AR-E expressed in HEK293T cells (Fig. 4d), measured using the nanoBRET-based assay. Data (Isoprenaline K_D) are the mean ± SEM of n = 3 independent biological experiments, each performed in duplicate. Significance was determined using log-transformed data (e.g. pK_D values) by one-way ANOVA with Dunnet's post-hoc tests.

[AS408] (μM)	Isoprenaline K_D (μM)	Isoprenaline pK_D	p value (compared to 0 μM AS408)
**0**	3.59 ± 1.31	5.45	-
**5**	10.97 ± 2.51	4.96	0.0132
**10**	21.57 ± 3.36	4.67	0.0003
**25**	32.23 ± 3.02	4.49	<0.0001
**50**	42.36 ± 8.51	4.37	<0.0001
**100**	55.68 ± 14.82	4.25	<0.0001

Supplementary Table 4. Conservation of the TM3-TM4-TM5 allosteric pocket between β_1AR, hβ_1AR, and hβ_2AR. The residue positions (Ballesteros-Weinstein numbering) for residues observed to interact with AS408 in the MD simulations are shown.

Residue Position	β_1AR	hβ_1AR	hβ_2AR
**3.41**	E	E	E
**3.44**	C	C	C
**3.45**	V	V	V
**3.48**	I	L	V
**34.56**	L	L	L
**34.57**	M	L	L
**4.45**	I	V	I
**4.48**	T	T	M
**4.49**	V	V	V
**4.52**	I	I	V
**5.45**	I	V	V
**5.49**	I	V	V
**5.50**	P	P	P
**5.53**	I	I	I

Supplementary Table 5. The primer sequences used for mutagenesis. 'fw', forward primer; 'rev', reverse primer.

Primer	Sequence
**Biophysics & NMR:**	
**E130V fw**	GTGACCTTGTGCGTCATCG
**E130M fw**	ATGACCTTGTGCGTCATCG
**E130I fw**	ATCACCTTGTGCGTCATCG
**E130L fw**	CTGACCTTGTGCGTCATCG
**E130F fw**	TTCACCTTGTGCGTCATCG
**E130Y fw**	TACACCTTGTGCGTCATCG
**E130X rev**	GATGCTTGCCGTCACG
**I168M fw**	GTCCGCTCTGGTCTCTTTCC
**I168M rev**	ATGGCCCAGACGGTGC
**Avi-β_1AR-X fw**	GCAGAAAATTGAATGGCATGAAGGGGCCGAGCTGC
**Avi-β_1AR-X rev**	GCTTCAAAAATATCGTTCAGGCCCATTTTGGGGGATCCGTATTTATAG
**Pharmacology:**	
**W130L fw**	GATGACGCACAAGGTCAAGATGCTTGCCGTCAC
**W130L rev**	GTGACGGCAAGCATCTTCACCTTGTGCGTCATC
**W130F fw**	GACGCACAAGGTGAAGATGCTTGCCGTCACGCAAAGC
**W130F rev**	GCTTTGCGTGACGGCAAGCATCTTCACCTTGTGCGTC
**W130I fw**	GCGATGACGCACAAGGTGATGATGCTTGCCGTCACGC
**W130I rev**	GCGTGACGGCAAGCATCATCACCTTGTGCGTCATCGC
**W130V fw**	GATGACGCACAAGGTCACGATGCTTGCCGTCACG
**W130V rev**	CGTGACGGCAAGCATCGTGACCTTGTGCGTCATC
**W130Y fw**	GACGCACAAGGTATAGATGCTTGCCGTCACGCAAAGC
**W130Y rev**	GCTTTGCGTGACGGCAAGCATCTATACCTTGTGCGTC
**W130M fw**	GATGACGCACAAGGTCATGATGCTTGCCGTCACG
**W130M rev**	CGTGACGGCAAGCATCATGACCTTGTGCGTCATC

Supplementary Table 6. The composition of the lipid membrane used in MD simulations. The distribution between the upper and lower leaflets are shown. The lipid composition was taken from Song *et al.*, 2019[13]. Abbreviations: 1-palmitoyl-2-oleoyl-sn-glycero-3-phosphocholine (POPC), 1,2-Dioleoyl-sn-glycero-3-phosphocholine (DOPC), 1-palmitoyl-2-oleoyl-sn-glycero-3-phosphoethanolamine (POPE), 1,2-dioleoyl-sn-glycero-3-phosphoethanolamine (DOPE), 1-palmitoyl-2-oleoyl-sn-glycero-3-phospho-L-serine (POPS), 1,2-dioleoyl-sn-glycero-3-phospho-L-serine (DOPS), 1-palmitoyl-2-oleoyl-sn-glycero-3-phosphoinositol (POPI), N-Palmitoylsphingomyelin (PSM), N-stearoyl–D-erythro monosialodihexosyl ganglioside (GM3).

Lipid Type	Upper leaflet		Lower leaflet		Total	
	Number	%	Number	%	Number	%
**Cholesterol**	32	25%	32	25%	64	25%
**POPC**	26	20%	6	5%	32	13%
**DOPC**	26	20%	6	5%	32	13%
**POPE**	6	5%	26	20%	32	13%
**DOPE**	6	5%	26	20%	32	13%
**POPS**	0	0%	10	8%	10	4%
**DOPS**	0	0%	9	7%	9	4%
**POPI**	0	0%	13	10%	13	5%
**PSM**	19	15%	0	0%	19	7%
**GM3**	13	10%	0	0%	13	5%
**Total**	128	-	128	-	256	-

Supplementary Table 7. Parameters for equilibration and production steps used in adjusting the turkey β_1AR model using the CHARMM36/CGenFF force field. The systems were equilibrated in six stages (50 ns total) with progressive lift of restraints followed by an unrestrained 1000 ns production run. Backbone (BB) restraints apply flexible positional constraints to the protein's backbone atoms and to the heavy atoms in the ligands; side chain (SC) restraints apply flexible positional constraints to the side chain atoms; and lipid restraints apply flexible positional constraints to the lipid components of the system and dihedral restraints apply torsional angle constraints.

Step	Step 1	Step 2	Step 3	Step 4	Step 5	Step 6	Step 7 (Production)
**Ensemble**	NVT	NVT	NPT	NPT	NPT	NPT	NPT
**Duration (ns)**	1	2	4	8	15	20	1000
**Time step (fs)**	1	1	1	2	2	2	2
**BB restraint ($kJmol^{-1}nm^{-2}$)**	4000	2000	1000	500	200	50	0
**SC restraint ($kJmol^{-1}nm^{-2}$)**	2000	1000	500	200	50	0	0
**Lipid restraint ($kJmol^{-1}nm^{-2}$)**	1000	400	400	200	40	0	0
**Dihedral restraint ($kJmol^{-1}rad^{-2}$)**	1000	400	200	200	100	0	0

Supplementary References

1. Solt, A. S. *et al.* Insight into partial agonism by observing multiple equilibria for ligand-bound and G_s-mimetic nanobody-bound β_1-Adrenergic receptor. *Nat. Commun.* **8**, 1795 (2017).

2. Frei, J. N. *et al.* Conformational plasticity of ligand-bound and ternary GPCR complexes studied by ^{19}F NMR of the β_1-adrenergic receptor. *Nat. Commun.* **11**, 669 (2020).

3. Warne, T. *et al.* The structural basis for agonist and partial agonist action on a β_1-adrenergic receptor. *Nature* **469**, 241–245 (2011).

4. Johnstone, E. K. M. *et al.* Investigation of receptor heteromers using nanoBRET ligand binding. *Int. J. Mol. Sci.* **22**, 1082 (2021).

5. Barkan, K. *et al.* Pharmacological characterisation of novel adenosine A_3 receptor antagonists. *Sci. Rep.* **10**, 20781 (2020).

6. Jones, A. J. Y. *et al.* Binding kinetics drive G protein subtype selectivity at the β_1-adrenergic receptor. *Nat. Commun.* **15**, 1334 (2024).

7. Olsen, R. H. J. *et al.* TRUPATH, an open-source biosensor platform for interrogating the GPCR transducerome. *Nat. Chem. Biol.* **16**, 841–849 (2020).

8. Su, M. *et al.* Structural basis of the activation of heterotrimeric Gs-protein by isoproterenol-bound β_1-adrenergic receptor. *Mol. Cell* **80**, 59-71 (2020).

9. Keov, P., Sexton, P. M. & Christopoulos, A. Allosteric modulation of G protein-coupled receptors: A pharmacological perspective. *Neuropharmacology* **60**, 24–35 (2011).

10. Liu, X. *et al.* An allosteric modulator binds to a conformational hub in the β_2 adrenergic receptor. *Nat. Chem. Biol.* **16**, 749–755 (2020).

11. Wu, F. J. *et al.* Activation dynamics traced through a G protein-coupled receptor by 81 ^{1}H-^{15}N NMR probes. *Science* **388**, eadq9106 (2025).

12. Leach, K., Sexton, P. M. & Christopoulos, A. Allosteric GPCR modulators: taking advantage of permissive receptor pharmacology. *Trends Pharmacol. Sci.* **28**, 382–389 (2007).

13. Song, W., Yen, H. Y., Robinson, C. V. & Sansom, M. S. P. State-dependent lipid interactions with the A_{2a} receptor revealed by MD simulations using *in vivo*-mimetic membranes. *Structure* **27**, 392-403 (2019).
