# Supplementary material for: Uncoupling of the allosteric signalling network in the β1-adrenergic receptor by a negative allosteric modulator
Source: Nat Commun. 2026 Sep 18;17:10952. doi: 10.1038/s41467-026-77802-8 (PMC13680373; doi:10.1038/s41467-026-77802-8)
Supplement: Supplementary file 2 [file 41467_2026_77802_MOESM2_ESM.pdf]

nature portfolio

Corresponding author(s): Prof. Daniel Nietlispach

Last updated by author(s): Aug 13, 2026

Reporting Summary

Nature Portfolio wishes to improve the reproducibility of the work that we publish. This form provides structure for consistency and transparency in reporting. For further information on Nature Portfolio policies, see our Editorial Policies and the Editorial Policy Checklist.

Statistics

For all statistical analyses, confirm that the following items are present in the figure legend, table legend, main text, or Methods section.

n/a	Confirmed	
☐	☒	The exact sample size (*n*) for each experimental group/condition, given as a discrete number and unit of measurement
☐	☒	A statement on whether measurements were taken from distinct samples or whether the same sample was measured repeatedly
☐	☒	The statistical test(s) used AND whether they are one- or two-sided<br>*Only common tests should be described solely by name; describe more complex techniques in the Methods section.*
☒	☐	A description of all covariates tested
☐	☒	A description of any assumptions or corrections, such as tests of normality and adjustment for multiple comparisons
☐	☒	A full description of the statistical parameters including central tendency (e.g. means) or other basic estimates (e.g. regression coefficient) AND variation (e.g. standard deviation) or associated estimates of uncertainty (e.g. confidence intervals)
☐	☒	For null hypothesis testing, the test statistic (e.g. *F*, *t*, *r*) with confidence intervals, effect sizes, degrees of freedom and *P* value noted<br>*Give P values as exact values whenever suitable.*
☒	☐	For Bayesian analysis, information on the choice of priors and Markov chain Monte Carlo settings
☒	☐	For hierarchical and complex designs, identification of the appropriate level for tests and full reporting of outcomes
☒	☐	Estimates of effect sizes (e.g. Cohen's *d*, Pearson's *r*), indicating how they were calculated

Our web collection on statistics for biologists contains articles on many of the points above.

Software and code

Policy information about availability of computer code

Data collection	The NMR data was recorded on Bruker Avance III spectrometers running Topspin 3.1 software. BioLayer Interferometry data was recorded using the Octet RED96 with Data Acquisition V 11.0.0.64 (Pall ForteBio LLC) software. TRUPATH and ligand affinity data was collected using BMG Labtech PHERAstar (software Version 1.60 R4, Firmware version 1.33) and exported as Excel files. BRET biosensor data was collected on a CLARIOstar Plus® microplate reader (SMART control version 6.20, Mars version 4.20, BMG Labtech, UK) and exported as Excel files. Flow cytometry data was recorded using a BD LSRFortessa™ Cell Analyzer (BD Biosciences) using BD FACSDiva™ Software v9.0. Molecular dynamics simulations were conducted using GROMACS version 2024.6.
Data analysis	The raw 1H-13C NMR data was processed using the processing package Azara v2.8 (W. Boucher) and then analysed using Analysis CCPN version 2.4. The 19F NMR data was processed and analysed using Bruker Topspin 4.4.1. Deconvolution of 1D NMR spectra was done using in-house written software (R.W. Broadhurst, T. H. Harman, unpublished). Ligand binding kinetics data, TRUPATH data, and BRET biosensor data was analysed using Graphpad prism 10.5 for pharmacological data. Flow cytometry data was analysed with OMIQ software and Graphpad prism 10.5. BioLayer Interferometry data was analysed using Data Analysis V 11.0.0.4 (Pall ForteBio LLC) software and Graphpad prism 10.5. The analysis of molecular dynamics trajectories was carried out with mdciao v1.0.0, MDAnalysis v2.10.0, and ProLIF v2.1.0. Snapshots were visualised using ChimeraX v1.10.1. Other structures were visualised using PyMOL 3.1.0.

For manuscripts utilizing custom algorithms or software that are central to the research but not yet described in published literature, software must be made available to editors and reviewers. We strongly encourage code deposition in a community repository (e.g. GitHub). See the Nature Portfolio guidelines for submitting code & software for further information.

Data

Policy information about availability of data

All manuscripts must include a data availability statement. This statement should provide the following information, where applicable:

- Accession codes, unique identifiers, or web links for publicly available datasets
- A description of any restrictions on data availability
- For clinical datasets or third party data, please ensure that the statement adheres to our policy

The authors declare that all relevant data supporting the findings of this study are available within the article and its Supplementary Information file, in public repositories at links below, or on request from the corresponding author. All data aside from MD simulation data are available at https://figshare.com/projects/Data_sets_for_-_Uncoupling_of_the_Allosteric_Signalling_Network_in_the_sub_1_sub_-adrenergic_receptor_by_a_Negative_Allosteric_Modulator/267181. This permanent link is accessible in the Data Availability statement. The PDB accession codes used in analysis of NMR data in this study (Y203, 7JJO, 6OBA) are also included in the Data Availability statement. Molecular dynamics trajectories (compressed to every tenth frame to reduce file sizes), are available on Zenodo at https://doi.org/10.5281/zenodo.19371846. This permanent link is accessible in the Data Availability statement.

Research involving human participants, their data, or biological material

Policy information about studies with human participants or human data. See also policy information about sex, gender (identity/presentation), and sexual orientation and race, ethnicity and racism.

Reporting on sex and gender	No data was analysed using human participants or human data.
Reporting on race, ethnicity, or other socially relevant groupings	No data was analysed using human participants or human data.
Population characteristics	No data was analysed using human participants or human data.
Recruitment	No data was analysed using human participants or human data.
Ethics oversight	No data was analysed using human participants or human data.

Note that full information on the approval of the study protocol must also be provided in the manuscript.

Field-specific reporting

Please select the one below that is the best fit for your research. If you are not sure, read the appropriate sections before making your selection.

[x] Life sciences [ ] Behavioural & social sciences [ ] Ecological, evolutionary & environmental sciences

For a reference copy of the document with all sections, see nature.com/documents/nr-reporting-summary-flat.pdf

Life sciences study design

All studies must disclose on these points even when the disclosure is negative.

Sample size	Each NMR receptor sample was produced individually and assessed for structural integrity by NMR in the apo state. This was done for every sample independently prior to addition of further reagents such as agonists or coupling partners to verify the integrity of the starting materials. BLI data was recorded using n=3 individual technical replicates. TRUPATH assays and ligand binding kinetics data were recorded using n≥3 (n≥2 for supplementary data) independent biological experiments with each performed in duplicate. BRET biosensor assays were recorded with n≥3 independent biological replicates, each performed in duplicate. Flow cytometry data were recorded using n = 4. MD simulations were recorded as three replicates. All sample sizes were determined using the guidelines from the British Journal of Pharmacology (instructions to authors and the published guidelines editorial (Curtis M. et al Br. J. Pharmacol. 2018 Apr;175(7):987-993). See individual figure and supplementary figure legends for exact n numbers.
Data exclusions	Data exclusion was not performed except for instances where control experiments failed in BLI, TRUPATH, and ligand binding data, and then the entire experiment was removed from analysis and repeated.
Replication	All TRUPATH and ligand binding experimental data are means ± SEM of n≥3 (n≥2 for supplementary data) independent biological experiments, each performed in duplicate, and analysed accordingly. BRET biosensor experimental data are the means ± SEM of n≥3 independent biological experiments, each performed in duplicate. Flow cytometry data are geometric means ± SEM of n=4 independent biological replicates. NMR experiments were repeated on independent samples to confirm that observed effects were reproducible. Reproducibility of BLI experiments was confirmed using n=3 independent experiments using three separate biosensors and separate sample wells for protein immobilisation and association/dissociation steps. Replication was successful for all experimental methodologies.
Randomization	Randomization in this study was not performed as data were not separated into experimental groups.
Blinding	Blinding was not performed in this study due to the exploratory nature of some of the experiments, and the very limited range of compounds and their concentrations used which would have made consistent blinding difficult to manage.

Reporting for specific materials, systems and methods

We require information from authors about some types of materials, experimental systems and methods used in many studies. Here, indicate whether each material, system or method listed is relevant to your study. If you are not sure if a list item applies to your research, read the appropriate section before selecting a response.

Materials & experimental systems

n/a	Involved in the study
☐	☒ Antibodies
☐	☒ Eukaryotic cell lines
☒	☐ Palaeontology and archaeology
☒	☐ Animals and other organisms
☒	☐ Clinical data
☒	☐ Dual use research of concern
☒	☐ Plants

Methods

n/a	Involved in the study
☒	☐ ChIP-seq
☐	☒ Flow cytometry
☒	☐ MRI-based neuroimaging

Antibodies

Antibodies used	APC anti-DYKDDDDK Tag Antibody - Cat number 637307 Biolegend. Dilution - 1 in 100.
Validation	Data showing validation in flow cytometry for each antibody is available on the Biolegend website (https://www.biolegend.com/en-gb/products/apc-anti-dykddddk-tag-antibody-8099)

Eukaryotic cell lines

Policy information about cell lines and Sex and Gender in Research

Cell line source(s)	Ligand binding assays and TRUPATH experiments were conducted in HEK293T cells purchased from ATCC (CRL-3216). BRET biosensor experiments were conducted in HEK293A cells (a gift from David Yule, Thermofisher, R70507). GPCR expression was performed in Spodoptera frugiperda (Sf9) cells (Thermofisher, 12659017).
Authentication	HEK293T cell line authentication was done by ATCC based on morphology. HEK293A authentication was performed based on morphology as reported by ATCC: https://www.atcc.org/products/crl-1573.
Mycoplasma contamination	HEK293T and HEK293A cells tested negative for mycoplasma contamination. Routine mycoplasma testing is performed every three months. The Sf9 cell line, used for receptor expression, tested negative for mycoplasma contamination, which conducted by Thermofisher. The Sf9 cell line was not routinely tested for mycoplasma contamination. However, the integrity of the expressed and purified proteins were routinely assessed by NMR, size exclusion chromatography and SDS PAGE gels.
Commonly misidentified lines (See ICLAC register)	No commonly misidentified cell lines were used in this study.

Flow Cytometry

Plots

Confirm that:

☒ The axis labels state the marker and fluorochrome used (e.g. CD4-FITC).

☒ The axis scales are clearly visible. Include numbers along axes only for bottom left plot of group (a 'group' is an analysis of identical markers).

☒ All plots are contour plots with outliers or pseudocolor plots.

☒ A numerical value for number of cells or percentage (with statistics) is provided.

Methodology

Sample preparation	~5 x 10^5 cells were pelleted (400 x g for 5 min). Cells were subsequently washed in FACS Buffer (PBS, 1 % BSA) and stained using APC anti-FLAG Ab (Biolegend, 637307, Dilution 1 in 100 FACS buffer) for 30 mins at room temperature and in the dark. Cells were washed and resuspended in FACS buffer, with 3 additions of 1x volume of ice cold 80% acetone. Cells were incubated for 1 hour at 4 degrees in the dark. Cells were washed again in FACS buffer, resuspended in 300 ul FACS buffer and acquired as below.
Instrument	Cells were acquired on the BD LSR-Fortessa Cell Analyzer (BD Bioscience).
Software	DB FACS Diva was used for acquisition. OMIQ software and Graphpad prism 10.5 was used for analysis.

Cell population abundance	Percentages of populations relevant to this analysis (single cell/ FLAG+) were determine as per the gating strategy below.
Gating strategy	(1) Cells, (2) Single Cells, (3) FLAG+.

☒ Tick this box to confirm that a figure exemplifying the gating strategy is provided in the Supplementary Information.
