# Supplementary material for: Uncoupling of the allosteric signalling network in the β1-adrenergic receptor by a negative allosteric modulator
Source: Nat Commun. 2026 Sep 18;17:10952. doi: 10.1038/s41467-026-77802-8 (PMC13680373; doi:10.1038/s41467-026-77802-8)
Supplement: Supplementary file 3 [file 41467_2026_77802_MOESM3_ESM.pdf]

nature portfolio

Peer Review File

Uncoupling of the Allosteric Signalling Network in the β_1-adrenergic receptor by a Negative Allosteric Modulator

Corresponding Author: Professor Daniel Nietlispach

This file contains all reviewer reports in order by version, followed by all author rebuttals in order by version.

Version 0:

Reviewer comments:

Reviewer #1

(Remarks to the Author)
Harman et al. describe a detailed mechanistic study of the effect of a negative allosteric modulator on the β1 adrenergic receptor. They first characterize in depth a range of mutants at the E130(3.41) site on the receptor, which happens to coincide with the binding site of the NAM AS408. For the mutations they can establish a clear functional distinction between aromatic and aliphatic substitutions, and the native aspartic acid. They combine F-NMR, H/C-NMR, MD and BLI to characterize the different mutants. From their data they can rationalize how the equilibria between inactive/pre-active and pre-active/active states are modulated by the different mutations. This is an extension of a previous piece of work by the same group but clearly reveals further insights into the mechanism of the reversal of this thermostabilizing mutation.
Then they turn their attention to the NAM AS408 and find that it rather uncouples the intracellular response from the extracellular agonist binding event and stabilizes a proposed novel conformation of the intracellular part of the receptor. Thus, it has a clearly different effect, than the mutations in close proximity examined in the first part of the work.

The work is absolutely sound (as usual from this group) and the reasoning and individual experiments are meticulously documented. The results on the different coupling and uncoupling mechanisms are significant enough to deserve publication in Nature Comm.

The manuscript's highly detailed and meticulous descriptions of the experiments, while thorough, make it somewhat difficult to read. Shifting some of this content to the supplementary material could improve readability. By guiding the reader through the findings with fewer explicit experimental steps in the main text, the authors would allow the narrative to flow more clearly and the key results would be highlighted more strongly.

Otherwise I have only minor comments:

Line 86: AS408 reduced arrestin recruitment to β1AR – β1 or β2?

Line 94: The advantages of AMs are praised, however, how selective is a NAM that acts on human β2AR as well as on β1AR from turkey?

Line 166: It seems that two different F-tags were used (TET and BFTA). What was the reason for this and would this have an influence on the interpretation of the extracted data?

Line 263: The I168M mutation seems to be a great probe for binding of the allosteric pocket. This is a nice tool for the field.

Line 271: Would it be possible to determine the affinity of AS408?

Line 324: > 50 nM – μM ?

Fig. 2.: Could one NMR spectrum with a wider spectral window be included? The presented signals are essentially devoid of any spectral noise. However, knowing the noise level would be instructive to judge the meaningfulness of the small active state contributions.

Discussion:
How is the conservation of residues in the allosteric pocket between turkey β1AR, human β1AR and human β2AR? Can any predictions be made on how the NAM will act on human β1AR?

It is probably out of scope, but can BLI measurements be carried out with arrestin and miniG proteins other than miniGs? It would be interesting to see, if AS408 changes the preference of β1AR towards a transducer, ie. whether the newly found conformation has an affinity towards arrestin or another G-protein.

Reviewer #2

(Remarks to the Author)
Allosteric networks are critical for transmitting signals from the extracellular orthosteric pocket to intracellular signalling pathways. The current study explored how disruption of allosteric networks within the β1AR can influence the conformational landscape sampled by the receptor. Within the study, the allosteric network is disrupted by either mutagenesis or an allosteric modulator. Authors share interesting results, however, additional evidence is required to support the different mechanisms outlined - namely, that mutations shift receptor equilibria to populate states with weaker G protein affinities, whereas the negative allosteric modulator reduces G protein coupling by adopting an alternative signalling-incompetent intracellular surface arrangement. Authors should move beyond isoprenaline to determine whether this is a global mechanism or whether probe dependence is observed. It would be useful to compare the effects of the negative allosteric modulator and orthosteric antagonist, both alone and in combination with the orthosteric agonist, to understand whether the mechanism is conserved across ligand classes. Many aspects of the pharmacological design and analyses in the current form are not correct and must be revisited to enable confidence in the pharmacological parameters determined and, as a consequence, the mechanism underpinning the findings. Specifically, mutagenesis interpretation is reliant on consistent receptor expression, which has not been shown; statistical analyses were performed on non-logarithmic data for affinity and potency values which is incorrect; design of interaction studies does not allow for determination of pharmacological parameters such as allosteric affinity and cooperativity.

Major Comments:

Can the authors clarify whether the human or turkey β1AR has been used throughout to explore the mechanism? Can the authors comment on the influence of species differences?

Figure 1: In relation to the pharmacological profiling and conformational equilibria of β1AR constructs bearing mutations at E1303.41, differences in receptor expression between assays may complicate interpretation. Although both the binding (Nluc-BRET) and TRUPATH assays use transient expression, receptor levels may vary from day to day and ideally should be normalised within each experiment. However, normalisation to the Nluc-tagged construct may not reflect the expression of the different constructs used in binding versus functional assays, as the Nluc tag itself may alter expression. As such, receptor expression must be assessed for each mutant across all assays, as expression differences can significantly influence mechanistic interpretation by altering the maximal effect and apparent potency of isoprenaline in functional assays. The affinity of the fluorescent antagonist, (S)-propranolol-red, was reported for the binding assay across different mutations (Supplementary Figure 3b & 3d). The influence of mutations on receptor expression (Bmax) must also be provided. As mentioned, receptor expression (Bmax) must be independently reported for binding and functional assays (e.g. Fig. 1e; Supplementary Figure 3, Supplementary Figure 4, Supplementary Figure 20) as differential tagging of receptor constructs may influence relative receptor expression across these contexts.

Throughout the manuscript, affinity and potency values must be reported and statistically analysed on a log scale, as only log-transformed data exhibit normally distributed errors. Affinity and potency analyses performed on linear-scale data must be reanalysed, and subsequent interpretation re-evaluated. All statistical tests and comparisons should be clearly stated in both the manuscript and figure legends. Furthermore, the text describing differences in KD values should specify whether the reported affinity refers to Mini-Gs or ligand affinity for the receptor.

Operational model of allosterism: authors must state and rationalise which parameters they have constrained and why. For example, for functional analyses, it appears that the beta value has been constrained. However, this has not been stated. Moreover, as the alpha value reflects binding cooperativity, it should be roughly equivalent across the different binding and functional assays (e.g., TRUPATH vs intramolecular). Can authors comment on the observed difference in Log alpha across assays? Authors must also rationalise the observed (but not reported) difference in beta values (efficacy modulation). Whilst AS408 causes a surmountable inhibition in isoprenaline potency for TRUPATH, insurmountable inhibition is observed for the intramolecular biosensor.

The study measures Mini-Gs binding kinetics to support mechanistic conclusions. An important missing element is the assessment of how mutations and AS408 affect the ligand-binding kinetics of orthosteric ligands. Such kinetic analyses of ligand binding are crucial for interpreting changes observed at the G-protein level and should be feasible using the NanoBRET platform.

The authors state “The ratio between the areas under the curve (AU C) for the active state (A) against the pre-active state (P) indicated that unlike β1AR-E, which primarily occupied the active state…..”. Could the authors please amend the extra space in AUC. Does the data Fig 2C support this claim? A roughly 60% active vs 40% pre-active population is shown. The

ratio between the two states is clearly different between the β1AR-E and the mutations. However, for E there is still an appreciable % of the receptor in the pre-active state, so it is unclear how the authors have determined that it primarily occupies the active state. Further to this, can the authors comment on whether it is surprising that saturating concentrations of isoprenaline did not confer greater stabilisation of active states vs pre-active?

Can the authors please clarify if a ligand was used in the MD simulations?

Figure 3a: AS408 interaction with isoprenaline appears consistent with a competitive interaction. Can the authors detail the pharmacological evidence for AS408 acting as a negative allosteric modulator at the β1AR, as currently this is unclear.

Figure 4d: The same concentration of (S)-propranolol-red must be used for the binding interaction studies evaluating the allosteric modulation of AS408 against isoprenaline and (S)-propranolol-red. This allows global fitting of data to derive AS408 affinity alongside allosteric cooperativity between isoprenaline and (S)-propranolol-red. The authors used different concentrations of (S)-propranolol-red across different concentrations of AS408, as detailed in the methods for in cell ligand affinity measurements, which means these analyses cannot be performed. Can the authors comment on why the assay was designed in this manner?

Can the authors please explain the large discrepancy between maximal AUC values for isoprenaline in Supplementary Fig 23B (value of ~7) and Fig 6C (value of ~1.5).

In supplementary figure 23, kinetic traces are shown for isoprenaline and propranolol but not AS408. Additionally, AS408 concentration response curves (in the absence of orthosteric ligand) are not shown. Both kinetic traces and curves for AS408 should be performed and shown. This will allow readers to compare across the 3 different ligands.

Figure 6: Have the authors (or others) completed full interactions between isoprenaline and propranolol with this biosensor at the β1AR? Validation of the biosensor with an orthosteric antagonist is ideal and will confirm that the sensor is capable of discerning competitive interactions.

Fig. 1e: The isoprenaline curve for β1AR-E is fit with a one-site model, but the data visually resemble a two-site fit. Can authors compare the preference for a one and two site fit?

Supplementary Fig. 5c: the association kinetic fits do not appear to pass through the data points at higher Mini-Gs concentrations (500 nM, 1000 nM, 2000 nM). The mismatch at the kinetic inflection point will influence the predicted association rate, so it should be addressed

Supplementary Fig. 5c: Mini-Gs kinetics form a central component of the mechanistic interpretation. Authors must include the kinetics for all datasets and fits, including mutations and the allosteric modulator.

In Figure 6 legend, the authors state “Data fitted using the 3 parameter logistic equations except for the 100 μM AS408 which was fitted with a simple linear regression curve due to the lack of discernible agonist activation.” Greater clarity is required with respect to this statement. There is no visible curve fit through the 100 μM AS408 condition despite the authors stating so. Furthermore, a linear regression would not be the appropriate model to fit to that data.
The authors are quantifying allosteric interactions by fitting the operational model of allosterism, but these fits are not shown in Fig 6c. The operational model of allosterism fit should be shown, as this is required for data interpretation.

Supplementary Table 2. “The p values for comparisons of binding kinetics parameters for isoprenaline-stimulated mini-Gs binding between different β1AR E130X3.41 receptor constructs. The p values were calculated using unpaired two-tailed t-tests for BLI data in Fig. 1f-g and Supplementary Fig. 5d.” Can the authors comment on why t-tests were used in this instance? Each mutant is compared to wildtype, therefore, wouldn’t a One-Way ANOVA with Dunnett’s post-hoc test be more appropriate?

Minor Comments:

To strengthen the mechanistic understanding of receptor modulation, authors could include additional datasets examining Mini-Gs kinetics and affinity in the presence of antagonist, the allosteric modulator alone, and the modulator together with isoprenaline.

Fig. 1a legend currently presents the schematic as fact, but it should be made clear whether the mechanism is known or proposed.

Figure 2: The percentage population of active/pre-active states for the hydrophobic residues is shown in panel 2C. However, for the aromatic residues, only W is shown. Can the authors comment on why W is in panel C but there are no corresponding 19F NMR spectra shown?

Figure 2: Can the authors comment on whether the stabilisation of these pre-active states for the different mutations would lead to reduction in potency and/or Emax in the TRUPATH assay? Can they link these findings to their functional data presented in Fig 1e?

Figure 2: Can the authors please include the concentration of isoprenaline (1 mM) used in these NMR experiments, either in

the text and/or figure legend.

For the E130I mutation, there is binding data (Fig 1d), functional data (Fig 2e) and MD simulation data (Supp Fig 10, 12). Why is there no corresponding NMR data for this mutation?

Supplementary Figure 10: The different shaded bars in Supplementary Figure 10 are very hard to visualise. Different colours would make them easier to see.

Figure 3c “Req (response values from 1:1 global fitting with Rmax unlinked by sensors) for mini-Gs binding to isoprenaline-bound β1AR-E modulated by AS408, measured by BLI. Data are mean ± SEM of n=3 independent technical replicates.” Can authors provide additional details as to what Req means? This is currently unclear.

Figure 3d: Authors state these data are “fit with [Inhibitor] vs Response (three parameter variable slope), with a Hill constant fixed at -1”, can the authors confirm this is correct, as the fit does not seem to be a 3-parameter sigmoidal concentration-response.

Figure 4e: Reader interpretation would benefit from reporting AS408 affinity and cooperativity values, as opposed to changes in isoprenaline affinity in the presence of AS408.

The authors state “Consistent with similar intramolecular sensors58–60, we observed a dose-dependent loss of BRET signal for isoprenaline (pEC50 – 8.16 ± 0.3) with very little change upon application with propranolol (Supplementary Fig. 23b), validating our β1AR-E BRET-sensor.” Please remove the negative value or alternatively just state the EC50. Can the authors state which data set was used to source this EC50 value? The concentration response data for isoprenaline in the absence of AS408 does not visually yield a pEC50 8.16 in Fig 6C or Supplementary Fig 23B.

Fig 1e: The colour scheme used to differentiate the V and W mutations is difficult to interpret because both are displayed in orange.

Supplementary Figure 20: It appears that an increase in Bmax occurs with increasing concentrations of AS408. Although possible, this would be an unusual finding – can authors provide a comment and explanation for this?

Results section, 3rd paragraph: The statement “We found little significant differences between wildtype β1AR-E (2.13 µM) and hydrophobic mutants or β1AR-W (p>0.05).” should instead state that no significant differences were detected.

Reviewer #3

(Remarks to the Author)
This manuscript presents a multifaceted investigation into the mechanisms of negative allosteric modulation at the β1 adrenergic receptor (β1AR), combining primarily structural and functional analyses through NMR spectroscopy and complementary cell-based assays. The authors compare the effects of targeted mutations at residue E130 (3.41, Ballesteros-Weinstein numbering) with those of the small-molecule modulator AS408, revealing distinct structural mechanisms by which both perturb receptor signalling. Overall, the experimental framework appears rigorous and the study offers a relevant contribution to the understanding of GPCR allostery. In line with my expertise, this review will focus primarily on the molecular dynamics (MD) simulation component of the work, where I have identified several key concerns that I encourage the authors to address.

1) According to the Methods, the authors started MD simulations from the agonist-bound turkey β1AR structure (PDB 2Y03), which Warne et al. (2011) showed to remain an inactive, non-signalling conformation ('None of the structures show the outward movement of the cytoplasmic end of transmembrane helix H6 by 5–6 A that is observed during light activation of rhodopsin. This indicates that the structures represent an inactive, non-signalling state of the receptor formed on initial agonist binding.'). Thus, the simulations effectively start from an inactive-state receptor. Although comparing the pocket residue behaviors among the wild-type E130 (3.41) and its mutants is informative, this analysis is inherently limited to the inactive state. Consequently, the authors’ statement that 'direct interactions with P219 (5.50) were not sufficient to stabilise β1AR in the inactive state' is not well-supported by the data, since only the inactive conformation was briefly sampled (triplicates of 300 ns) with no exploration of transitions from an active state.

2) It is clear that the authors aim to examine how various mutations (particularly involving the key residue E130) affect the receptor’s allosteric pocket. However, regardless of the starting structure conformational state, the limited trajectory length combined with the system’s size and complexity means these MD simulations are likely far from equilibrium. The authors should explicitly clarify to readers, especially those not well-versed in MD simulations, that under these conditions the results must be interpreted qualitatively rather than quantitatively.

3) Referring to the contacts as 'interactions' is potentially misleading: substituting a negatively charged glutamate (E130) with hydrophobic residues in a predominantly hydrophobic pocket yields contacts fundamentally different from the polar or charged interactions originally possible with the glutamate side chain. The analysis presented thus appears to reduce to measurements of spatial proximity rather than a comparison of equivalent interaction types, making it unclear what meaningful interactions are actually being evaluated. Furthermore, it is unsurprising that introducing a hydrophobic residue stabilises the hydrophobic core through additional hydrophobic contacts. Finally, the impact of these mutations on pocket flexibility (e.g. RMSD/RMSF of pocket residues) and on the conserved PIF motif during transitions between active and

inactive states remains unaddressed in the current analysis.

4) In addition to concerns about using an inactive-state template (PDB 2Y03), issues with the modelling arise from the fact that the template's sequence does not fully match that of the experimental constructs. According to the paragraph 'System preparation for MD simulations', only residue E130 was mutated. However, several other sequence discrepancies between the template and the constructs appear to remain unaddressed beyond the focus on residue 130. For example, the experimental constructs contain a methionine at position 90 (Supplementary Fig. 1 constructs A and B), whereas the 2Y03 template contains a valine at that position. Similarly, the constructs carry a tyrosine at position 227 (part of the conserved Y5.58-Y7.53 microswitch), whereas the 2Y03 structure contains an alanine at this position; this mutation suppresses 'the formation of the active conformation in binary agonist complexes thereby abrogating G-protein binding' (https://doi.org/10.1038/s41557-022-01009-9). At position 282, construct A contains an alanine, whereas construct B contains a cysteine for NMR labelling; however, the template contains a leucine at this site. These discrepancies between the simulation model and the actual experimental constructs raise concerns about the accuracy of the simulations and could undermine confidence in the conclusions drawn.

5) The authors state that 'Hydrogen atoms were added, and amino acids were kept in their standard protonation state at pH of 7.0.', yet they do not clarify the specific protonation state of E130. This residue, situated within a predominantly hydrophobic environment, may not remain deprotonated under physiological conditions. Indeed, protonation state predictions from tools such as Schrödinger Maestro, along with previous computational studies, suggest that E130 could be neutral at pH 7. As such, assuming a standard negatively charged glutamate side chain without justification introduces uncertainty, since this choice could meaningfully alter the interactions within the allosteric pocket and affect the conclusions drawn from the simulations.

6) The authors do not indicate whether the MD trajectories will be made publicly available. For transparency and reproducibility, the authors should consider depositing the trajectories in a publicly accessible repository such as Zenodo.

In summary, while the experimental aspects of this study are methodologically strong and provide clear mechanistic insight into β1AR allosteric modulation, the MD section falls short of this standard. Due to the use of an inactive-only starting structure, unresolved sequence mismatches with the experimental constructs, limited trajectory length, lack of equilibrium sampling, and the omission of AS408-bound systems, the simulations in their current form do not offer meaningful support for the manuscript's conclusions. As such, the manuscript would not suffer in scientific integrity or clarity were this section to be removed. Alternatively, if the authors wish to retain a computational component, a more rigorous approach would involve modelling an active-like β1AR conformation reflecting the actual experimental constructs, followed by longer simulations to evaluate how E130 mutations or AS408 binding influence deactivation pathways. In such a framework, monitoring canonical GPCR activation markers (such as the PIF motif, Y-Y distance, ionic lock formation, NPxxY motif, TM6 tilting, ion occupancy) would offer a more substantive contribution to the mechanistic narrative.

General observations:

1) In Supplementary Figure 28 (lower panels), the authors show the residue Tyr227 as part of the 2Y03 crystal structure. However, this residue is not present in the original PDB entry, which lists Ala227 in both chains A and B. This figure representation should be clarified.

2) Typo at page 7 'Supplemenatry Fig. 16a-b'.

3) Grammatical inconsistency at page 21: 'Part of the IL3 (amino acid residues 244-271) was not present in this structure and were not modelled into the β1AR structure used in simulations. '

4) Grammatical inconsistency at page 22: 'Analysis of MD trajectories were performed with mdciao'

5) The manuscript frequently refers to GPCR activation states and microswitches such as the PIF motif, which play important roles in the conformational regulation of the receptor. While these terms are familiar to GPCR specialists, a brief explanatory description summarising their relevance to receptor activation would help orient non-specialist readers and improve the manuscript's accessibility for a broader audience.

Reviewer #4

(Remarks to the Author)
I co-reviewed this manuscript with one of the reviewers who provided the listed reports. This is part of the Nature Communications initiative to facilitate training in peer review and to provide appropriate recognition for Early Career Researchers who co-review manuscripts.

Version 1:

Reviewer comments:

Reviewer #1

(Remarks to the Author)

I thank the authors for their detailed answers to my questions I originally had. They have been answered absolutely adequately, such that I have no further reservations towards publication of this manuscript.

Reviewer #2

(Remarks to the Author)
Thank you to the authors for their thoughtful responses to comments. The work presents valuable insights into the allosteric modulation of β_1AR by AS408, integrating pharmacological, biophysical, and computational approaches. The authors have addressed many of the earlier concerns, and the manuscript has improved accordingly. However, some important issues remain that should be further clarified or addressed with additional data prior to publication.

Original reviewer comment: Authors should move beyond isoprenaline to determine whether this is a global mechanism or whether probe dependence is observed.

The authors have addressed this comment. Consider mentioning the technical challenges posed by the narrow signal-to-noise window in the conformational BRET-based biosensor.

Original reviewer comment: It would be useful to compare the effects of the negative allosteric modulator and orthosteric antagonist, both alone and in combination with the orthosteric agonist, to understand whether the mechanism is conserved across ligand classes.

The authors address this comment in Supplementary Figure 30.

Original reviewer comment: Can the authors clarify whether the human or turkey β1AR has been used throughout to explore the mechanism? Can the authors comment on the influence of species differences?

The authors have clarified the species used and discussed potential differences.

Original reviewer comment: Figure 1: In relation to the pharmacological profiling and conformational equilibria of β1AR constructs bearing mutations at E1303.41, differences in receptor expression between assays may complicate interpretation.

The authors have adequately addressed this comment.

Original reviewer comment: Throughout the manuscript, affinity and potency values must be reported and statistically analysed on a log scale, as only log-transformed data exhibit normally distributed errors.

The authors have adequately addressed this comment.

Original reviewer comment: Operational model of allosterism: authors must state and rationalise which parameters they have constrained and why.

Author Response: "We rationalised our choice of parameters used in the operational model of allostery as to facilitate a direct comparison with the analysis of AS408 action at the β2AR as described in Liu et al., 2020, in that we constrained parameters in the same manner in order to replicate their analysis for the turkey β1AR. In the Supplementary Table 2 of that study, the constraints are stated: "τβ and β-values were fixed at ~0 since AS408 is a NAM and has no agonist efficacy, respectively." Consequently, we have fixed our parameters similarly. We also fix the Hill coefficient at 1.0. We have subsequently added an additional table to the Supplementary Information file (Supplementary Table 3) which includes these details, and we have added a short section to the methods (entitled "Operational model of allostery") to further detail these analyses."
New reviewer comment: The authors have provided additional documentation, but several important issues remain:
1) Nomenclature: τβ must be corrected to τB throughout the manuscript.
2) The statement "τβ and β-values were fixed at ~0 since AS408 is a NAM and has no agonist efficacy, respectively" must be reworded for accuracy. Currently, it implies the β parameter drives agonist efficacy, whereas this is driven by τB – the intrinsic efficacy of the allosteric modulator. β (efficacy cooperativity) describes how the NAM affects the agonist's ability to activate the receptor, not the NAM's own efficacy.
3) Constraining β to ~0 is appropriate, as the maximal agonist response decreases to baseline with increasing concentrations of AS408. Can authors ensure the reasoning for constraining this value is included within the manuscript?
4) The mechanism of action for AS408 needs to be described more clearly throughout the manuscript. The manuscript emphasises α (affinity cooperativity) while lacking discussion of β (efficacy cooperativity). The authors should explicitly state that AS408 NAM activity is driven by both parameters.

Original reviewer comment: Can authors comment on the observed difference in Log alpha across assays? Authors must also rationalise the observed (but not reported) difference in beta values (efficacy modulation).

Author Response: "We speculate that the variation in the α values is likely a result of the differing assays utilised to obtain these values. The biosensor directly reads out the conformational change in the receptor induced by the agonist co-stimulated with AS408, whereas the TRUPATH assay responds to G protein activation and is therefore an indirect measure of changes at the receptor.
We further note that the log(α) value was observed to vary at the β2AR: -0.7 +/- 0.5 for [35S]GTPγS binding; -0.1 +/- 0.1 for

cAMP accumulation; and -0.2 +/- 0.1 for β-arrestin recruitment (Liu et al., 2020). The differences here were attributed to "better access of the modulator to the receptor in broken cell preparations (as in the [35S]GTPγS binding assays) compared to the whole cell assays (cAMP and β-arrestin recruitment assays)". Consequently, variations in log(α) values may not be entirely unsurprising between different experimental systems.
We have now clarified in the methods section and in Supplementary Table 3 that the β values are held constant at near 0 in the operational model fits."

New reviewer comment: The author response does not appropriately address the question. Affinity cooperativity (α) should be a property of receptor-ligand interactions, independent of the system, unless there are equilibrium artifacts or NAM bias. The GTPγS dissociation example provided by the authors does not appear relevant to the current manuscript, as both assays in this study are whole-cell and measure downstream signalling or conformational changes rather than direct G protein binding.

Original reviewer comment: An important missing element is the assessment of how mutations and AS408 affect the ligand-binding kinetics of orthosteric ligands.

Author Response: "We thank the Reviewer for raising this important question and we have now conducted Nluc ligand binding assays, as suggested by the Reviewer, to address these questions. We find that there are no significant differences in either the association or dissociation rates of both (S)-propranolol-red and isoprenaline between the E130X3.41 variants. Therefore, we find that the ligand binding kinetics will have little impact on the interpretation of the BLI assays assessing mini-Gs binding to the receptor variants. These binding kinetics have been added to the manuscript in Supplementary Figure 3-4.
We have further recorded association or dissociation rate constants of both (S)-propranolol-red and isoprenaline to β1AR-E in the presence of varying AS408 concentrations. These data support our previous observations in that AS408 has little effect on the affinity to (S)-propranolol-red but increases the Ki for isoprenaline binding. This increase is a result of dose-dependent decreases in association rates for isoprenaline binding, whereas we observed no significant changes in koff rates for isoprenaline nor any significant changes in koff or kon rates for (S)-propranolol-red binding. The data for these kinetic assays have been added to the manuscript in Supplementary Figure 26-27."

New reviewer comment: The authors have added valuable kinetic data, but the interpretation requires revision. The statement that there are " These data support our previous observations in that AS408 has little effect on the affinity to (S)-propranolol-red but increases the Ki for isoprenaline binding. This increase is a result of dose-dependent decreases in association rates for isoprenaline binding, whereas we observed no significant changes in koff rates for isoprenaline nor any significant changes in koff or kon rates for (S)-propranolol-red binding" is contradicted by Supplementary Figure 27G, which shows a significant change in koff at a single AS408 concentration. Similarly, Supplementary Figure 27D shows a significant change in kon for (S)-propranolol-red at a single concentration.

Original reviewer comment: Can the authors please clarify if a ligand was used in the MD simulations?

The authors have adequately addressed this comment.

Original reviewer comment: Figure 3a: AS408 interaction with isoprenaline appears consistent with a competitive interaction. Can the authors detail the pharmacological evidence for AS408 acting as a negative allosteric modulator at the β1AR, as currently this is unclear.

Author Response: "In the TRUPATH assay data shown in Figure 3a, we agree that the limited response to isoprenaline at the highest AS408 concentration (100 µM) could be consistent with a competitive interaction. However, a likely possibility is that the full curve is not present and a higher concentration of isoprenaline would be required to obtain full dose-response curves (i.e. AS408 may shift the potency for isoprenaline further to the right). A similar effect is also observed for β1AR-F and β1AR-Y TRUPATH data (Figure 1e) in which full concentration response curves are not observed. Additional pharmacological evidence to suggest that AS408 acts allosterically, rather than competitively, includes the absence of significant changes in the affinity of the antagonist (S)-propranolol-red in the presence of the AS408. We would anticipate that at 100 µM AS408, we would observe competition of the antagonist and AS408. Consequently, the pharmacological data points towards an allosteric mode of action.
We also note that other structural data indicates that there is no competitive interaction between the orthosteric ligand and AS408 at βARs. The AS408-β2AR crystal structure in Liu et al., 2020, demonstrated binding into an allosteric binding pocket, and our complementary NMR data were consistent with binding of the AS408 into this same pocket at the turkey β1AR (Fig. 3g-h).
Furthermore, other NMR data indicates that AS408 does not bind into the orthosteric pocket. E.g. the M178 probe, which reports on the conformational dynamics and ligand identity in the orthosteric pocket, shows little to no changes in the apo spectra of β1AR-E (M178, Fig. 4e), β1AR-M (Supplementary Fig. 35d), β1AR-F (Supplementary Fig. 33d), and β1AR-Y (Supplementary Fig. 33b). Additional NMR data recorded for the propranolol-bound β1AR-E (Supplementary Fig. 25c,f) clearly demonstrated that the unique position that M178 signal occupies when bound to the antagonist is strongly maintained upon the addition of AS408, whereas other signals clearly respond in the expected manner when the receptor populates the AS408-stabilised 'non-productive' conformation. The M178 signal position remains consistent with the propranolol-stabilised chemical shift position, which notably differs from the two positions for M178 in the apo receptor with addition of AS408. Therefore, these data clearly evidence AS408 binding and related changes in conformations and dynamics whilst also distinctly demonstrating propranolol binding to the orthosteric pocket.
Finally, the supplementary 1 µs simulations (recorded in triplicate) of β1AR-E with the AS408 modelled in the β2AR binding

pose (Fig. 3d-f and Supplementary Fig. 22) further support an allosteric binding mode. In these simulations, AS408 is maintained in the TM3-TM4-TM5 binding pocket, although interacting with different regions of the allosteric pocket. Hence, these data also support AS408 as a negative allosteric modulator at the β1AR.
Overall, the data indicating that AS408 does not displace the fluorescent tracer ((S)-propranolol-red) alongside the structural dataset solidifies our conclusion that AS408 acts as an allosteric modulator at the TM3-TM4-TM5 pocket and does not compete for binding in the orthosteric pocket."

New reviewer comment: While appreciating the authors have biophysical evidence supporting an allosteric mechanism of action for AS408, the following comment on pharmacological evidence should be reconsidered "Additional pharmacological evidence to suggest that AS408 acts allosterically, rather than competitively, includes the absence of significant changes in the affinity of the antagonist (S)-propranolol-red in the presence of the AS408. We would anticipate that at 100 µM AS408, we would observe competition of the antagonist and AS408. Consequently, the pharmacological data points towards an allosteric mode of action." This is because 100 µM AS408, which has an affinity of ~250 µM, would be expected to cause a <2-fold shift in propranolol for a competitive interaction, since the concentration of AS408 is less than the affinity (KD). Therefore, the lack of effect in this binding assay is not supportive of either allosterism or competitive interactions. Authors should rephrase the interpretation.

Original reviewer comment: Can the authors please explain the large discrepancy between maximal AUC values for isoprenaline in Supplementary Fig 23B (value of ~7) and Fig 6C (value of ~1.5).

The authors have adequately addressed this comment.

Original reviewer comment: In supplementary figure 23, kinetic traces are shown for isoprenaline and propranolol but not AS408. Additionally, AS408 concentration response curves (in the absence of orthosteric ligand) are not shown. Both kinetic traces and curves for AS408 should be performed and shown.

The authors have adequately addressed this comment.

Original reviewer comment: Figure 6: Have the authors (or others) completed full interactions between isoprenaline and propranolol with this biosensor at the β1AR? Validation of the biosensor with an orthosteric antagonist is ideal and will confirm that the sensor is capable of discerning competitive interactions.
The authors have addressed this comment.

Original reviewer comment: Fig. 1e: The isoprenaline curve for β1AR-E is fit with a one-site model, but the data visually resemble a two-site fit. Can authors compare the preference for a one and two site fit?

Author Response: "We agree with the Reviewer that the data shown in Fig. 1e could be consistent with a two-site fit, we suggest that this is an artifact of the experimental system. The HEK293T cells used in TRUPATH experiments are known to endogenously express several adrenergic receptors, including the β2AR and to some extent the β1AR (e.g. Such expression is commented on for HEK293 cells in: Ferrie AM, et al., Divergent label-free cell phenotypic pharmacology of ligands at the overexpressed β_2-adrenergic receptors. Sci Rep. 4:3828. (2014)). We hypothesise that the biphasic behaviour observed in the TRUPATH data may be linked to these endogenously expressed receptors, in that additional activation of endogenously expressed receptors at the higher concentrations of isoprenaline increases the signal at the concentrations but not the lower concentrations. It is worth noting that the only curve which shows this is the β1AR-E and likely arises from experimental variation. Finally, it is well established that there is only a single binding site for isoprenaline at the β1AR. We cite the existing literature for this agonist which has been extensively characterised both pharmacologically (e.g. Baker 2010) and structurally (including PDB: 2Y03 (isoprenaline-bound β1AR crystal structure), 7JJO (isoprenaline-β1AR-Gs complex), 7S0F (isoprenaline-β1AR-Gi complex), 6H7J (isoprenaline-β1AR-Nb80 complex), 8S2T (isoprenaline-human β1AR-Gs complex)."

New reviewer comment: Agree with the authors that the only curve that shows this is the β1AR-E, and that it likely arises from experimental variation.

Original reviewer comment: Supplementary Fig. 5c: the association kinetic fits do not appear to pass through the data points at higher Mini-Gs concentrations (500 nM, 1000 nM, 2000 nM). The mismatch at the kinetic inflection point will influence the predicted association rate, so it should be addressed.

The authors have adequately addressed this comment.

Original reviewer comment: Supplementary Fig. 5c: Mini-Gs kinetics form a central component of the mechanistic interpretation. Authors must include the kinetics for all datasets and fits, including mutations and the allosteric modulator.

The authors have adequately addressed this comment.

Original reviewer comment: In Figure 6 legend, the authors state "Data fitted using the 3 parameter logistic equations except for the 100 µM AS408 which was fitted with a simple linear regression curve due to the lack of discernible agonist activation." Greater clarity is required with respect to this statement.

The authors have adequately addressed this comment.

Original reviewer comment: The authors are quantifying allosteric interactions by fitting the operational model of allosterism, but these fits are not shown in Fig 6c. The operational model of allosterism fit should be shown, as this is required for data interpretation.

The authors have adequately addressed this comment.

Original reviewer comment: Supplementary Table 2. Can the authors comment on why t-tests were used in this instance? Each mutant is compared to wildtype, therefore, wouldn't a One-Way ANOVA with Dunnett's post-hoc test be more appropriate?

The authors have adequately addressed this comment.

Original reviewer comment: To strengthen the mechanistic understanding of receptor modulation, authors could include additional datasets examining Mini-Gs kinetics and affinity in the presence of antagonist, the allosteric modulator alone, and the modulator together with isoprenaline.

The authors have adequately addressed this comment.

Original reviewer comment: Fig. 1a legend currently presents the schematic as fact, but it should be made clear whether the mechanism is known or proposed.

The authors have addressed this comment.

Original reviewer comment: Figure 2: The percentage population of active/pre-active states for the hydrophobic residues is shown in panel 2C. However, for the aromatic residues, only W is shown. Can the authors comment on why W is in panel C but there are no corresponding 19F NMR spectra shown?

The authors have adequately addressed this comment.

Original reviewer comment: Figure 2: Can the authors comment on whether the stabilisation of these pre-active states for the different mutations would lead to reduction in potency and/or Emax in the TRUPATH assay? Can they link these findings to their functional data presented in Fig 1e?

The authors have adequately addressed this comment.

Original reviewer comment: Figure 2: Can the authors please include the concentration of isoprenaline (1 mM) used in these NMR experiments, either in the text and/or figure legend.

The authors have adequately addressed this comment.

Original reviewer comment: For the E130I mutation, there is binding data (Fig 1d), functional data (Fig 2e) and MD simulation data (Supp Fig 10, 12). Why is there no corresponding NMR data for this mutation?

The authors have adequately addressed this comment.

Original reviewer comment: Supplementary Figure 10: The different shaded bars in Supplementary Figure 10 are very hard to visualise. Different colours would make them easier to see.

The authors have adequately addressed this comment.

Original reviewer comment: Figure 3c "Req (response values from 1:1 global fitting with Rmax unlinked by sensors) for mini-Gs binding to isoprenaline-bound β1AR-E modulated by AS408, measured by BLI. Data are mean ± SEM of n=3 independent technical replicates." Can authors provide additional details as to what Req means? This is currently unclear.

The authors have adequately addressed this comment.

Original reviewer comment: Figure 3d: Authors state these data are "fit with [Inhibitor] vs Response (three parameter variable slope), with a Hill constant fixed at -1", can the authors confirm this is correct, as the fit does not seem to be a 3-parameter sigmoidal concentration-response?

The authors have adequately addressed this comment.

Original reviewer comment: Figure 4e: Reader interpretation would benefit from reporting AS408 affinity and cooperativity values, as opposed to changes in isoprenaline affinity in the presence of AS408.

The authors have addressed this comment.

Original reviewer comment: The authors state "Consistent with similar intramolecular sensors58–60, we observed a dose-

dependent loss of BRET signal for isoprenaline (pEC50 – 8.16 ± 0.3) with very little change upon application with propranolol (Supplementary Fig. 23b), validating our β1AR-E BRET-sensor." Please remove the negative value or alternatively just state the EC50. Can the authors state which data set was used to source this EC50 value? The concentration response data for isoprenaline in the absence of AS408 does not visually yield a pEC50 8.16 in Fig 6C or Supplementary Fig 23B.

The authors have adequately addressed this comment.

Original reviewer comment: Fig 1e: The colour scheme used to differentiate the V and W mutations is difficult to interpret because both are displayed in orange.

The authors have adequately addressed this comment.

Original reviewer comment: Supplementary Figure 20: It appears that an increase in Bmax occurs with increasing concentrations of AS408. Although possible, this would be an unusual finding – can authors provide a comment and explanation for this?

Author Response: "We agree with the Reviewer that this observation would indeed be unusual. However, we believe that this result is likely an artifact of the experimental system. The data shown in Supplementary Fig. 20b are a ratio of fluorescence/bioluminescence, and consequently are susceptible to interference by fluorescent ligands. The AS408 molecule, which has several delocalised π systems, appears to have some low-level fluorescence which has small effects on the measurements such as absorbance or quenching effects at wavelengths relevant to this experiment. The effect on the ratio is concentration dependent and thus has the greatest effect at the 100 µM AS408. In addition, the impact is exacerbated by the ratio which can artificially inflate errors. We therefore anticipate that the result is increased variability in the fluorescence/bioluminescence ratio which can result in misleading observations such as the change in Bmax here. In support of this observation, our new dataset that was recorded to obtain the kinetic parameters for ligand binding in the presence of AS408 demonstrates that there is now observed a decrease in the Bmax at the highest concentration of AS408 (Supplementary Fig. 26b). Consequently, we put this observation down to ligand-dependent artifacts rather than any real effect."

New reviewer comment: Authors suggest the increased Bmax as an artifact arising from the intrinsic fluorescence of AS408 due to delocalised π systems. However, this raises a significant concern, as most AS408 effects in these BRET assays occur at concentrations >100 µM, where the fluorescence artifact would be greatest. If AS408 fluorescence affects the BRET ratio in a concentration-dependent manner, it could also quench or enhance donor/acceptor signals and skew concentration-response curves. Can the authors demonstrate, in control experiments using a different GPCR for which AS408 has minimal affinity, that AS408 does not cause artifacts in this system? If AS408 causes fluorescence artifacts, the authors should acknowledge and, if possible, correct these effects.

Original reviewer comment: Results section, 3rd paragraph: The statement "We found little significant differences between wildtype β1AR-E (2.13 µM) and hydrophobic mutants or β1AR-W (p>0.05)." should instead state that no significant differences were detected.

The authors have adequately addressed this comment.

Reviewer #3

(Remarks to the Author)
I thank the authors for their thorough and constructive response to my previous comments. In my opinion, the revised manuscript is substantially improved. In particular, the molecular dynamics section has been significantly strengthened and is now much better aligned with the experimental quality of the work.

The authors have addressed the main concerns by replacing the previous inactive-state simulations with a new set of simulations starting from an active-like β1AR structure, extending the simulation time, matching the receptor sequence to the experimental construct, clarifying the protonation state of key residues, adding AS408-bound simulations, analysing canonical GPCR activation markers, and making the MD trajectories publicly available. Importantly, the revised text now appropriately acknowledges that full deactivation transitions are not sampled on the simulation timescale and therefore focuses the interpretation on local dynamics within the TM3-TM4-TM5 allosteric pocket. This more cautious framing is appropriate and well supported by the data.

The inclusion of AS408-bound simulations also considerably improves the mechanistic narrative. The computational observations are now better connected to the NMR and pharmacological data, and the revised MD analysis provides useful structural context for the experimentally observed differences between the effects of E130 mutations and AS408. Overall, I consider the computational component to be now consistent with, and supportive of, the broader experimental conclusions of the manuscript.

I therefore consider that my main concerns have been satisfactorily addressed and recommend acceptance. I have only two very minor editorial/clarificatory points for the authors to consider:

In the Methods, line 894, "currated β1AR-E structure" should be corrected to "curated β1AR-E structure".

For clarity, the authors may wish to specify that the reported hydrogen bonds involving I214 are backbone-mediated, since the isoleucine side chain itself cannot participate in hydrogen bonding.

Reviewer #4

(Remarks to the Author)
I co-reviewed this manuscript with one of the reviewers who provided the listed reports. This is part of the Nature Communications initiative to facilitate training in peer review and to provide appropriate recognition for Early Career Researchers who co-review manuscripts.

Version 2:

Reviewer comments:

Reviewer #2

(Remarks to the Author)
6. Original reviewer comment: Supplementary Figure 20: It appears that an increase in Bmax occurs with increasing concentrations of AS408. Although possible, this would be an unusual finding – can authors provide a comment and explanation for this?

New Reviewer comments:
Comment 6a) In relation to results section 4, paragraph 9 "BRET signal changes were assessed for classical, G protein/β-arrestin balanced orthosteric ligands from three broad classifications, including agonists (adrenaline, formoterol), a partial agonist (dobutamine), and an antagonist (propranolol) with increasing concentrations of AS408.… …Consequently, we concluded that AS408 does not display probe dependence at the β1AR when stimulated with balanced orthosteric ligands."

Reviewers agree that AS408 decreases the response of additional agonists. However, authors should reduce their claim around at lack of probe dependence, unless they are comparing quantified allosteric modulator pharmacology. Without quantification, it is simply an observed decrease in response for all ligands tested.

Comment 6b) In relation to "Whilst we do observe a decrease in Bmax and this may be due to quenching of the luminescence signal as proposed in our previous response, there are several other possible explanations for this reduction. For example, Bmax values from NanoBRET binding assays are susceptible to receptor conformational changes, as the signal is affected by the distance between the fluorescent ligand and NanoLuc tag. We therefore would like to avoid overinterpretation of this metric and hence do not discuss the observed difference in the manuscript.

We would like to highlight that we consistently see effects in these BRET assays at much lower concentrations than 100μM (a shift in potency using the biosensor is observed at 5 μM, Fig. 6c), at which there is very little interference from any compound fluorescence or quenching."

We can appreciate that the authors do not want readers to over interpret the potential compound interference, however we strongly feel that the compound interference must be described and cannot be excluded from the manuscript. This is important information for any researchers who may use or synthesise AS408 for their work in the future.

Comment 6c) In relation to "We do appreciate these data indicate a probe dependency for AS408 with respect to a biased ligand, and consequently we have amended the manuscript to indicate we do not see differences between classical, balanced ligands of the receptor"

Can reviewers please rephrase the comment "classical, balanced ligands" to "classical non-biased ligands, relative to the reference ligand, XX, used within the study".

Reviewer #4

(Remarks to the Author)
I co-reviewed this manuscript with one of the reviewers who provided the listed reports. This is part of the Nature Communications initiative to facilitate training in peer review and to provide appropriate recognition for Early Career Researchers who co-review manuscripts.

Open Access This Peer Review File is licensed under a Creative Commons Attribution 4.0 International License, which permits use, sharing, adaptation, distribution and reproduction in any medium or format, as long as you give appropriate credit to the original author(s) and the source, provide a link to the Creative Commons license, and indicate if changes were made.
In cases where reviewers are anonymous, credit should be given to 'Anonymous Referee' and the source.
The images or other third party material in this Peer Review File are included in the article's Creative Commons license, unless indicated otherwise in a credit line to the material. If material is not included in the article's Creative Commons license and your intended use is not permitted by statutory regulation or exceeds the permitted use, you will need to obtain permission directly from the copyright holder.
To view a copy of this license, visit https://creativecommons.org/licenses/by/4.0/

Manuscript NCOMMS-25-86704

Point-by-point reply to the reviewers' comments

We would like to thank all the Reviewers for their highly valuable comments, and for the endorsement of our manuscript and work. In our point-by-point reply we have now addressed all the issues raised by the reviewers and we believe that we have satisfied their queries and comments. We have modified our manuscript accordingly and we are persuaded that the changes have substantially improved the quality of our submitted work.

As highlighted by the Nature Communications Editorial team and in line with recommendations made by Reviewer #2 and Reviewer #3, we have now included additional pharmacological and NMR experiments, in addition to revised molecular dynamics simulations, to support our findings. In brief, the significant additions or revisions to the manuscript include:

- In response to Reviewer #1, we have removed some details from the main text in order to improve the readability of the manuscript and transferred this information to the Supplementary Information where appropriate.

- The analysis of AS408 was extended to investigate the impact of orthosteric probe dependence by analysing the effects of AS408 on receptor conformations using the BRET-sensor when bound to a range of different ligands with various efficacies, in response to Reviewer #2. These studies revealed no clear probe dependence for the tested ligands, in that AS408 behaves as a negative allosteric modulator at the β_1AR when bound to different orthosteric agonists.

- Additional β_1AR BRET biosensor, BLI, and NMR experiments were performed with an antagonist to assess the impact of AS408 on a receptor bound by a ligand from a different class, in response to Reviewer #2. We observed negligible G protein coupling to propranolol-bound β_1AR-E, in agreement with the antagonist activity of the orthosteric ligand, and no observable change in coupling with increasing addition of AS408. BRET-biosensor data revealed no large-scale concentration-dependent conformational changes with addition of AS408. Importantly, NMR data revealed that conformational changes occurred in the receptor core and intracellular surface upon binding of AS408 to the propranolol-bound receptor, consistent with those observed for the apo β_1AR-E or with receptor bound to the full agonist isoprenaline. These data confirmed the adoption of the 'non-productive' conformation stabilised by AS408 for a receptor bound by a ligand from a different class.

- The binding kinetics of the orthosteric ligand isoprenaline were measured for each E130X$^{3.41}$ variant in addition to for β_1AR-E with differing concentrations of AS408, in response to Reviewer #2. We reveal that, whilst the significant differences between isoprenaline affinities for β_1AR-E and β_1AR-F or β_1AR-Y are maintained, there were no significant differences in agonist or antagonist kinetics (both association and dissociation rates) observed between the β_1AR-E and any of the E130X$^{3.41}$ variants. Consequently, we find that ligand binding kinetics have a minor impact on the interpretation of other data, including mini-G affinities measured by BLI. We also find that the change in isoprenaline binding affinity induced by AS408 is due to a reduction in the association rate whereas the ligand dissociation rates remain similar. In contrast, there were no significant dose-dependent differences in binding kinetics for the fluorescent ligand (S)-

propranolol-red, aside from the highest concentration of AS408 for propranolol association rates.

- We recorded 24 µs of additional MD simulations for the E130X$^{3.41}$ variants with improvements to the model as suggested by Reviewer #3. These data substantially improve the computational aspect of our manuscript. The simulations reveal that the E130X$^{3.41}$ variants do not have substantial impacts on the overall receptor conformation in the timescale investigated, following analyses of key receptor regions suggested by the Reviewer. We do find that the interactions of the E130X$^{3.41}$ position do vary between the variants, specifically that the aromatic mutations favour interactions with TM5.

- We recorded 3 µs of simulations for β_1AR-E bound to AS408 in response to Reviewer #3, to support our NMR and pharmacological data in classifying AS408 as an allosteric modulator acting at the turkey β_1AR. We find that AS408 occupies the TM3-TM4-TM5 allosteric pocket across the simulations, thereby supporting our experimental data, but interestingly interacts with a different region of the pocket compared to the β_2AR. We also find two distinct AS408 binding poses in the allosteric pocket. Thus, these data support our hypotheses that AS408 acts differently at the β_1AR relative to the human β_2AR due to differing interactions with the receptor.

- Validation of receptor expression levels were performed using FLAG-Nluc tagged receptor variants in HEK293T cells, to confirm differences in TRUPATH assays or ligand binding affinities were not due to differential expression of the E130X$^{3.41}$ variants. These assays confirmed that there was no significant difference in cell surface expression between β_1AR-E and the E130X$^{3.41}$ variants.

- Additional BLI and NMR experiments were performed to confirm the effects of the E130I mutation on receptor G protein coupling and conformational dynamics, in response to Reviewer #2. These data were consistent with other E130X$^{3.41}$ variants, in that reductions in signalling and G protein binding are a consequence of pre-active state population.

- Further figures and supplementary figures for MD simulations, pharmacological data, NMR data, and BLI data have been included in response to comments from all Reviewers.

Details on the experimental results described above can be found either in the updated manuscript or in the point-by-point responses to the Reviewers' comments in the following sections (#1: pg.3 ; #2, pg.10, #3, pg. 26, #4, pg. 33).

Revised or new figures/panels include:
Fig. 1d, 1f, 2b, 2c, 2e, 2f, 3d, 3e, 3f, 4d, 6c.
SI Fig. 3, 4, 6, 7c, 7d, 8, 10, 14, 15, 16, 17, 18d, 19, 20, 21, 22, 25c-g, 26, 27, 28e, 29b, 30, 31, 36.

<u>Point-by-point reply to individual Reviewers:</u>

Reviewer #1

We would like to thank the Reviewer for reviewing our manuscript. We greatly appreciate their very favourable response and strong endorsement of our manuscript and research.

The manuscript's highly detailed and meticulous descriptions of the experiments, while thorough, make it somewhat difficult to read. Shifting some of this content to the supplementary material could improve readability.

We thank the Reviewer for this comment. We agree that the manuscript was, as submitted, detailed and could benefit from a reduction in the experimental details to improve the overall flow of the paper. Consequently, we have made efforts to edit the manuscript to remove extended details in order to improve the overall flow of the main text. These details have been subsequently moved to the supplementary information file where appropriate. We believe that the manuscript now has improved readability.

Minor comments:
Line 86: AS408 reduced arrestin recruitment to β1AR – β1 or β2?

The original paper to characterise AS408 demonstrated that AS408 reduced β-arrestin recruitment to the human β_2AR and several other receptors, which included the human β_1AR (Supplementary Figure 6 in Liu *et al*., 2020). To clarify this for the reader, we have amended the text (Introduction, Paragraph 5) to reflect this: "The same work found that, although the affinity of the NAM was highest for the hβ_2AR, AS408 reduced β-arrestin recruitment at several other related receptors in addition to hβ_2AR, including the hβ_1AR."

Line 94: The advantages of AMs are praised, however, how selective is a NAM that acts on human β2AR as well as on β1AR from turkey?

We thank the Reviewer for this comment. We agree that the AS408 itself is not entirely selective for the human β_2AR, but we believe that the general consensus in the allosteric modulator development field reflects advantages of allosteric modulators with respect to generally increased selectivity for receptor subtypes (as suggested in Changeux, J. P. & Christopoulos, A., 2016).

The small molecule modulator AS408 does show off-target effects at several receptors, as reported in Liu *et al.*, 2020, which includes the human β_1AR and α_1AR as well as minor effects at several other receptors (shown in Supplementary Figure 6 of that paper). However, the authors show the selectivity of AS408 towards the human β_2AR in a physiological context would arise from the increased affinity of AS408 for this receptor.

Here we use AS408 as a tool to study the differing structural mechanisms of allosteric modulation at a GPCR, but in terms of potency AS408 acts as a poor allosteric modulator at the turkey β_1AR and shows selectivity to the human β_2AR. This selectivity is especially prominent at concentrations which could be

expected to be observed in physiological conditions (i.e. low µM values). Consequently, AS408 could be expected to effectively show selectivity towards the human β_2AR in a cellular context.

We have modified the introduction (Paragraph 5) to read as follows:

"We then endeavour to compare these data to the effects elicited by a small molecule allosteric modulator. A recent study introduced AS408 as a NAM for the human β_2AR (hβ_2AR)[29]. AS408 was shown to bind hβ_2AR at the TM3-TM4-TM5 interface, with the 3.41 position forming a key part of the allosteric pocket. The same work found that, although the affinity for the NAM was highest at the hβ_2AR, AS408 reduced β-arrestin recruitment at several other related receptors in addition to hβ_2AR, including the human β_1AR (hβ_1AR). Based on sequence and structural homology, we anticipated that AS408 would act through the same allosteric site at the β_1AR. Consequently, AS408 could enable pharmacological and structural investigations of small molecule allosteric modulation, complementary to mutations at E130$^{3.41}$, at the β_1AR."

Line 166: It seems that two different F-tags were used (TET and BFTA). What was the reason for this and would this have an influence on the interpretation of the extracted data?

We thank the Reviewer for raising an important point which requires clarification. The TET ^{19}F-tag used in the TM7 (^{TET}C344$^{7.54}$) ^{19}F NMR experiments was used because a small hydrophobic tag was necessary at this position to not interfere with the functionally relevant conformational rearrangements of the receptor. Previous experiments examining labelling at C344$^{7.54}$ with the larger and less hydrophobic BTFA tag or another alternative small molecule ^{19}F-tag BTFMA resulted in such disruptions inactivating the receptor (see Frei *et al*., 2020), necessitating the use of TET labelling at this position. Similar experiments have been performed with TET labelling at the equivalent Cys$^{7.54}$ in the β_2AR (Liu *et al*., 2012). Additionally, use of the TET label maintains the hydrophobic nature of this region of the receptor which likely contributes to the tolerance of this label at C344$^{7.54}$ relative to the less hydrophobic BTFA and BTFMA tags.

Whilst the TET label had been previously utilised at the TM6 (A282C$^{6.27}$) position for ^{19}F NMR experiments of the β_1AR-W construct in Frei *et al*., 2020, or the equivalent Cys$^{6.27}$ in β_2AR, BTFA was substituted for TET because the BTFA tag provided a more stable C-S bond (relative to the labile S-S bond in TET labelling which is more susceptible to hydrolysis). Consequently, the lifetime of the BTFA-A282C$^{6.27}$ was greater than the TET-A282C$^{6.27}$ as the former was less prone to loss of signal through hydrolysis of the label in more solvent-exposed environments, such as the active state or in ternary complex with mini-G proteins, compared to the latter. Notably, as A282C$^{6.27}$ is more exposed to the solvent than C344$^{7.53}$ due to its position on the intracellular surface (giving rise to the increased TET hydrolysis), this position is more amenable to use of less hydrophobic and larger tags such as BTFA. The use of BTFA as a TM6 label for investigating GPCR conformational dynamics was also previously validated in our research examining β_1AR-E (in Jones *et al*., 2024).
Thus, the predominant reason for the use of different ^{19}F tags was to ensure that the receptor was labelled such that the conformational dynamics were not altered by the labelling and the label remained stable over the time course of NMR experiments.

The different properties of the two tags may influence the immediate local environments around each probe and thus have the potential to impact the conformational dynamics that are reported by each probe. However, due to the small size of the tags, we do not anticipate any major impacts on the

extracted data. Indeed, the relative populations of the active and pre-active states are generally in good agreement between the two ^{19}F probes, which indicated that the tags did not have major impacts on their surrounding environments and related conformational dynamics. We cannot rule out minor differences, which might be expected due to the differing properties of the tags, but we believe that such differences do not affect our conclusions with respect to receptor population changes.

Line 263: The I168M mutation seems to be a great probe for binding of the allosteric pocket. This is a nice tool for the field.

We thank the Reviewer for their kind acknowledgement of this experimental design with respect to clarifying the allosteric ligand pocket. We believe that these data can be of substantial utility in other similar studies to support other structural data in determining allosteric modulator binding pockets.

Line 271: Would it be possible to determine the affinity of AS408?

We note that this issue has also been raised by Reviewer #2, and is also answered in detail in the reply to Reviewer #2 through pharmacological assays.

Directly measuring the affinity of AS408 binding to the turkey β_1AR is challenging in pharmacological assays, as are most allosteric modulator affinity measurements. Ligand affinities are commonly measured in cells either through direct measurement of BRET transfer between a fluorescent ligand and a tagged receptor (i.e. with NLuc as in this work), or by competition between a characterised fluorescent ligand and a non-fluorescent ligand of interest. AS408 is not sufficiently fluorescent in the appropriate wavelengths for direct measurements using Nluc tagged receptors, or similar. In the case of allosteric modulators, finding a suitable fluorescent ligand for competition assays is also challenging as far fewer ligands, if any, may target the allosteric pocket of interest compared to the orthosteric pocket; this issue is also present for AS408. Consequently, the measurement of AS408 affinity to the receptor is challenging to measure via these strategies.

However, an alternative measurement can be obtained by fitting other functional data, such as the effects of AS408 on GPCR signalling, with models such as the Operational model of allostery. This model considers the affinities of both agonists and allosteric modulators as well as α, the factor of binding cooperativity between the orthosteric agonist and the allosteric modulator. We have measured the affinity values (K_B) by fitting functional data including G protein coupling in TRUPATH assays (Fig. 3a) and conformational rearrangements in BRET-sensor assays (Fig. 6c), which are $\log(K_B) = -3.6$ and -3.1, respectively. Thus, this micromolar affinity would be consistent with the fast exchange regime observed in the NMR data. We have now added this information into the manuscript (Results, Section 2, Paragraph 2): "The affinity of AS408 to the turkey ($\log(K_B) = -3.6 \pm 0.1$) was notably lower than at the human β_1AR stimulated with noradrenaline ($\log(K_B) = -5.6 \pm 0.1$)[29].", as to reflect the lower affinity which gives rise to the fast-exchange chemical shift changes.

We have not measured the affinity of the allosteric modulator using purified protein (i.e. by NMR or BLI) as AS408 appears to partition into the detergent micelles and thus measurements in detergent will likely provide an inaccurate value for the affinity of the ligand. We have evidence for the effect of the detergent

on the availability of AS408 in ^{1}H-^{13}C HMQC NMR spectra of isoprenaline-bound β_1AR-F. This receptor provided high intensity signals for all probes (due to the population of the inactive/pre-active state equilibrium) and therefore could be used to quantify how LMNG impacted the fast-exchange chemical shift changes induced by AS408. Comparing the spectra of isoprenaline-bound β_1AR-F with increasing AS408 addition (Fig. R1a) to the equivalent spectra with high LMNG concentration in the buffer (0.04% compared to the standard 0.02%, Fig. R1b) demonstrated that the higher LMNG concentration reduced the AS408-induced chemical shift changes for signals such as M296, M153, and M223. Thus, the shift towards saturation with AS408 and towards full population of the non-productive state was reduced for the higher LMNG concentration (Fig. R1c demonstrates the similarity of conformations between the two LMNG concentrations in the absence of AS408 but the large differences, especially in the aforementioned probes' signals, in the presence of 1000 µM AS408). We interpret these data as suggesting that AS408 can associate with LMNG, such as in empty micelles that are present in addition to receptor-containing micelles, and therefore the true "available" concentrations of AS408 are likely reduced under NMR conditions relative to the nominal amount added. Consequently, we feel that any measurements of AS408 affinity using purified protein would be inaccurate and thus we only report the pharmacological measurements of AS408 affinities.

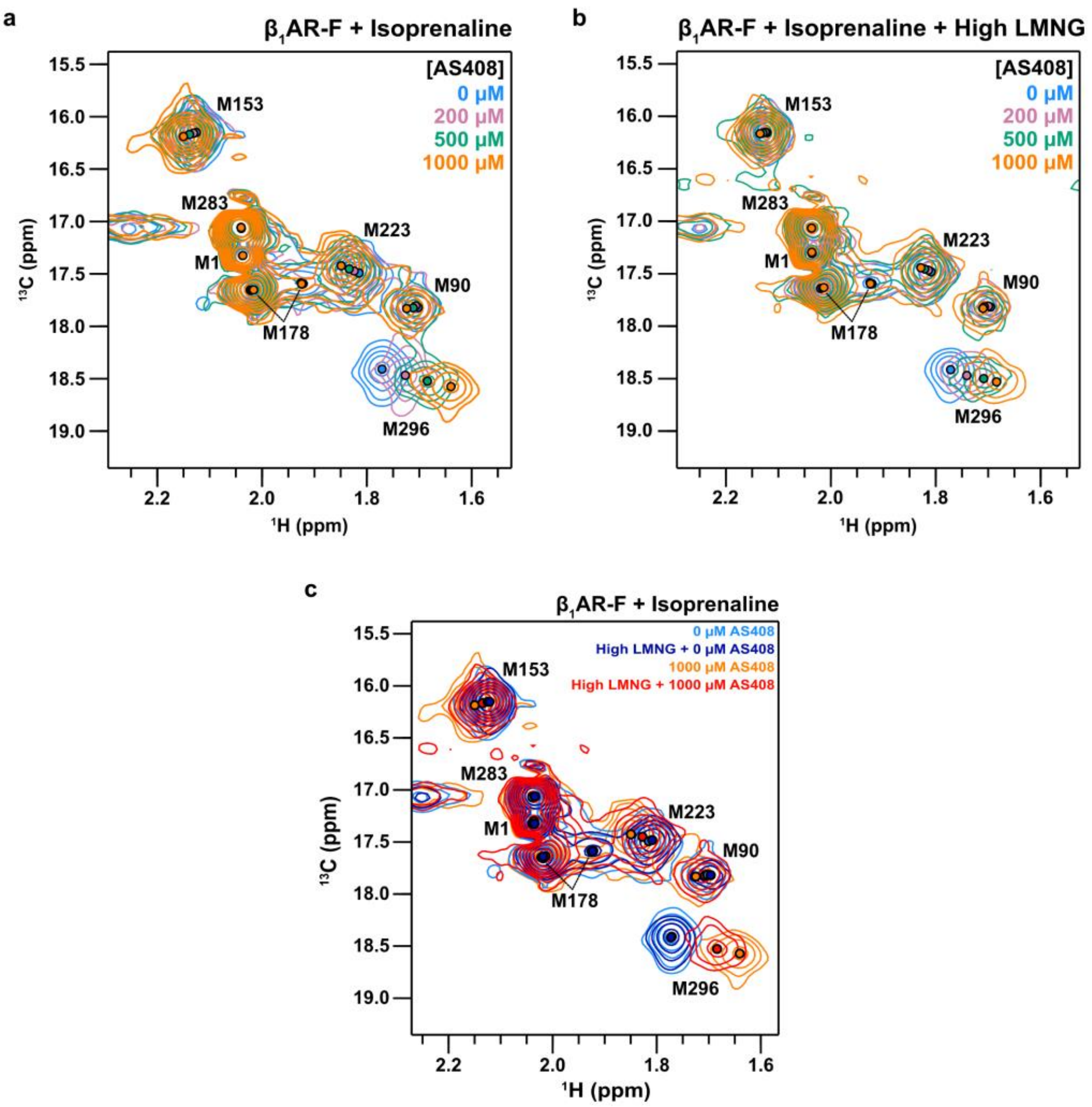

Fig. R1: The effects of LMNG concentration on AS408-dependent chemical shift changes in ^{13}C NMR spectra. a The 2D ^{1}H-^{13}C NMR spectrum of isoprenaline-bound β_1AR-F at 308 K, in 0.02% LMNG, with increasing addition of AS408. **b** The 2D ^{1}H-^{13}C NMR spectrum of isoprenaline-bound β_1AR-F at 308 K, in a higher LMNG concentration of 0.04%, with increasing addition of AS408. **c** The 2D ^{1}H-^{13}C NMR spectrum of isoprenaline-bound β_1AR-F at 308 K, comparing the spectra measured with either 0 µM or 1000 µM AS408 in buffers containing 0.02% LMNG or 0.04% LMNG (High LMNG).

We note that a similar result could be inferred from the crystal structures of AS408-bound β_2AR. Despite the much stronger binding affinity at this receptor compared to at the β_1AR, the crystal structure revealed only a 79% occupancy bound the receptor despite addition of 1 mM AS408 into crystallisation conditions. This result could indicate a general effect of detergents on measurements of binding affinities for this highly hydrophobic allosteric modulator.

Line 324: > 50 nM – µM ?

We thank the Reviewer for highlighting this error. This data has been re-measured, in response to Reviewer #2's comments, with the trend being maintained, and thus the wording in this section has been altered accordingly, correcting this error (Results, Section 3, Paragraph 4): "We noted that the intensity of the M178$^{4.62}$ active state signal decreased with increasing AS408 addition due to peak broadening (Fig. 4c), which suggested that AS408 reduced the binding affinity of isoprenaline to β_1AR-E. In cell ligand affinity measurements corroborated this conclusion (Supplementary Fig. 26-27), in which AS408 induced a significant ($p < 0.05$) concentration-dependent reduction in the isoprenaline affinity to β_1AR-E (Fig. 4d, Supplementary Table 4)."

Fig. 2.: Could one NMR spectrum with a wider spectral window be included? The presented signals are essentially devoid of any spectral noise. However, knowing the noise level would be instructive to judge the meaningfulness of the small active state contributions.

We agree with the Reviewer that a figure with a wider spectral window would be beneficial to demonstrate the relative signal to noise in the ^{19}F spectra and support the relative populations of the states. Given the limited space in Figure 2, we have decided that inclusion of the spectra with wider spectral windows would be best shown in a supplementary figure. As such, we have included this data for each spectrum shown in Figure 2 in the updated Supplementary Figure 10. Additionally, we have included the signal-to-noise ratios for each spectrum, as further evidence of the high quality of these data.

How is the conservation of residues in the allosteric pocket between turkey β1AR, human β1AR and human β2AR? Can any predictions be made on how the NAM will act on human β1AR?

The residues in the allosteric pocket are generally well conserved between the turkey β_1AR and human β_1AR, but some key differences are observed. The 5.45 and 5.49 positions (shown for turkey β_1AR and human β_2AR in Supplementary Figure 21) are isoleucine residues in the turkey β_1AR but are valine residues in human β_1AR (and thus are conserved between human β_1AR and human β_2AR). Furthermore, the 3.48 position, which forms key interactions with the phenyl ring of the AS408 in the β_2AR crystal structure in Liu *et al*., 2020, is an isoleucine residue in turkey β_1AR, a leucine residue in human β_1AR, and a smaller valine residue in human β_2AR.
Overall, the allosteric pocket in human β_1AR more closely resembles the human β_2AR than the turkey β_1AR but has a key and potentially pivotal difference at the 3.48 position. Thus, it is challenging to predict the mechanism of the AS408 at the human β_1AR without experimental investigation, but we might

anticipate that the bulky leucine at the 3.48 position may favour a mechanism closer to the action of AS408 at the turkey β_1AR which also has a bulky isoleucine relative to the smaller valine in human β_2AR.

We have recorded 3 µs of MD simulations of β_1AR-E with AS408 modelled into the simulation, initially at the same position as in β_2AR, at the request of Reviewer #3. Details on these simulations can be found in the submitted manuscript or the response to Reviewer #3. In brief, AS408 interacts with a different region of the TM3-TM4-TM5 pocket in two poses, located further towards TM4 or IL2 , respectively, which we hypothesise is the driving factor for the differences observed in the mechanisms between human β_2AR and turkey β_1AR.
The 3.48 position forms key hydrophobic and Van der Waals interactions with the phenyl ring system in AS408 (Supplementary Fig. 22a) and therefore represents a key position which could influence AS408 binding poses. Indeed, steric clashes between AS408 and the Ile at the 3.48 position (Supplementary Fig. 21a) may contribute to the reorientation of AS408 in the binding pocket in β_1AR compared to the AS408-β_2AR interaction. It is possible that the bulky Leu residue at position 3.48 in the human β_1AR may also induce population of the poses observed in our simulations of turkey β_1AR.

Both the human and turkey β_1AR have lower affinities for AS408 relative to β_2AR: the affinity of the AS408 at the turkey β_1AR obtained from fitting TRUPATH data with the operational model of allostery ($\log(K_B)$ = -3.6) was in the micromolar range, as was the affinity to human β_1AR reported in Liu *et al.*, 2020 ($\log(K_B)$ = -5.6) though bound tighter at the human β_1AR. In contrast, the affinity at the human β_2AR ($\log(K_B)$ = -6.8) was tighter, in the nanomolar range. The reduced affinity of AS408 to the human β_1AR may point towards the AS408 interacting with alternative regions of the allosteric pocket, although this alone does not constitute evidence for an AS408 binding pose at the human β_1AR similar to turkey β_1AR. Indeed, the affinity at the human β_1AR is still considerably greater than at the turkey β_1AR.

In addition to the differences in the allosteric pocket, we cannot rule out that the overall structural or dynamic differences could impact the mechanism of AS408 as the effects of this allosteric modulator on the receptor structure extend considerably from the allosteric pocket, as evidenced by ^{13}C or ^{19}F NMR spectra of intracellular probes in TM6 and TM7 (Figure 5). Differences between the human and turkey β_1AR energy landscapes may therefore impact the ability of AS408 to influence the conformation of the receptor beyond the allosteric pocket, or the relative stability or energy barrier to population of the non-productive conformation. Indeed, a recent NMR study investigating the human β_1AR thoroughly characterised the equilibria of receptor states and how their populations were modulated by the orthosteric agonist (Rößler *et al.*, 2025). Key observations in this paper included that the human β_1AR was more conformationally dynamic in its resting state (akin to our inactive/pre-active equilibrium) than its turkey β_1AR orthologue. As such, the energy landscapes between the human and turkey β_1AR likely differ slightly in relative stabilities and energy barriers between receptor states.

Consequently, we cannot make definitive conclusions on the mechanism of AS408 at the human β_1AR without experimental evidence such as NMR data. The key change at the 3.48 position could indicate that the AS408 acts via similar mechanisms at the human and turkey β_1ARs, but the lack of exact identity, other differences in the allosteric pocket, and the potential differences in overall receptor dynamics mean we cannot predict the exact mechanism with accuracy.
From our perspective, the action of AS408 differing at similar receptors with conserved mechanisms of activation and overall highly conserved allosteric pockets suggests that these modulators act with subtlety to influence receptor signalling and necessitates additional research. Investigations of modulators such as AS408 at the human β_1AR would certainly represent an intriguing and valuable follow-up investigation. Importantly we note that we use the turkey β_1AR as a model GPCR which can be characterised

structurally and pharmacologically, rather than as a proxy for the human receptor, and therefore an investigation between the human β_1AR and the human β_2AR was never the goal of this research.

We have summarised these points in the discussion (Paragraph 7) in the following text: "The reorientation of AS408 in the TM3-TM4-TM5 allosteric pocket in MD simulations of β_1AR resulted in altered interactions relative to AS408-bound hβ_2AR, including between the phenyl ring and the 3.48 position. The TM3-TM4-TM5 allosteric pocket is generally well conserved between the β_1AR, hβ1AR, and hβ_2AR (Supplementary Table 5), but notably the 3.48 position has bulkier Ile and Leu residues in β_1AR and hβ_1AR, respectively, compared to a smaller Val residue in hβ_2AR. This difference may favour the differing AS408 binding pose at the β_1AR to reduce steric clashes between the AS408 phenyl ring and the larger Ile side chain (Supplementary Fig. 21a) and consequently AS408 may also reorient similarly at hβ_1AR. However, differences in overall receptor dynamics between β_1AR and hβ_1AR[44] could also contribute to differing mechanisms."

It is probably out of scope, but can BLI measurements be carried out with arrestin and miniG proteins other than miniGs? It would be interesting to see, if AS408 changes the preference of β1AR towards a transducer, ie. whether the newly found conformation has an affinity towards arrestin or another G-protein.

We agree with the Reviewer that this is an intriguing question but is indeed beyond the scope of this study and would be well suited to complementary follow-up research.

The development of a BLI assay for β-arrestin binding to the β_1AR would require optimisation of the expression and purification of arrestin, in addition to a phosphorylated GPCR C-terminal tail mimetic to activate the arrestin, followed by optimisation of β-arrestin/β_1AR BLI assays. Similarly, addition NMR and BLI experiments with other mini-G proteins would require additional optimisation.

Reviewer #2

We would like to thank the Reviewer for reviewing our manuscript and providing detailed suggestions to improve our study. We believe that we have been able to address all the concerns raised.

Authors should move beyond isoprenaline to determine whether this is a global mechanism or whether probe dependence is observed.

We have now performed additional experiments to establish the possibility of probe dependence for the action of AS408 at the β_1AR. We achieved this by examining the impact of AS408 on a range of orthosteric ligands utilising the conformational BRET-based biosensor shown in Figure 6. We assessed the effects of AS408 on ligand-stimulated conformational changes in β_1AR-E for the following additional ligands: Full agonists isoprenaline, adrenaline, formoterol; Partial agonist dobutamine; Antagonist propranolol. These ligands were selected to assess the impact of different efficacies and structurally different orthosteric ligands on the action of AS408. In all cases, we observed similar trends in the effects of AS408, with [AS408]-dependent decreases in the BRET efficiency present for all agonists. In the case of the antagonist propranolol, no significant signal changes were observed upon orthosteric ligand addition (consistent with the known stabilisation of inactive states by this ligand) and no [AS408]-dependent decreases in the BRET efficiency changes. Therefore, we conclude that we can observe no orthosteric probe dependence in these pharmacological assays. The biosensor data have been added to the manuscript in the Supplementary Information file, in Supplementary Figure 31. We have added a short section to the manuscript to reflect this investigation (Results, Section 4, Paragraph 9): "We further investigated the potential for probe dependency (a phenomenon where an allosteric modulator's effect on a receptor differs depending on the orthosteric ligand) for AS408 at the β_1AR using the β_1AR-E BRET-sensor. BRET signal changes were assessed for orthosteric ligands from three broad classifications, including agonists (adrenaline, formoterol), a partial agonist (dobutamine), and an antagonist (propranolol) with increasing concentrations of AS408. Adrenaline, formoterol, and dobutamine all activated the receptor and we observed concentration-dependent decreases in ΔBRET signal with increasing AS408 addition (Supplementary Fig. 31), analogous to that observed for isoprenaline (Fig. 6c). We observed little change in the propranolol signal with increasing concentrations of AS408. Consequently, we concluded that AS408 does not display probe dependence at the β_1AR."

In view of limited resources with respect to research time and funding, we did not further extend our investigations to an exhaustive screen of the many β_1AR ligands available. Based on the dataset we provide in this manuscript, we feel confident that we are unlikely to observe any probe dependence. While in theory, there may be possible ligand candidates that might show probe dependence with respect to AS408, we feel screening for such an eventuality goes beyond the intent of this manuscript in which we aim to characterise two differing mechanisms of allosteric modulation observed in the same allosteric pocket.

It would be useful to compare the effects of the negative allosteric modulator and orthosteric antagonist, both alone and in combination with the orthosteric agonist, to understand whether the mechanism is conserved across ligand classes.

We have now recorded data in experiments using the conformational biosensor, BLI assays, and ^{1}H-^{13}C NMR for β_1AR-E in the presence of the antagonist propranolol in which we assess if the mechanism of AS408 is conserved across different classes of ligands. These data have been added to the updated Supplementary Information file, as indicated below. These data extend our dataset, showing the effects of AS408 at the agonist-bound receptor (isoprenaline), the apo receptor, and the antagonist-bound receptor.

We find that, as expected, propranolol stabilises the well-characterised and previously observed (Solt et al., 2017, Isogai et al., 2016, Grahl et al., 2020) inactive state of the receptor, observed by the negligible change in BRET efficiency from the apo receptor in the conformational biosensor assay (Supplementary Fig. 29) and the clear population of the inactive state signal positions in the 2D ^{1}H-^{13}C HMQC NMR spectrum of propranolol-bound β_1AR-E (Supplementary Fig. 25c). Very little signal was observed in BLI assays for interactions between β_1AR-E and the binding partner mini-G_s (Supplementary Fig. 20c), consistent with the very poor affinity of the inactive state of β_1AR-E with binding partners.

The NMR data demonstrates that the activity of this antagonist fundamentally differs from the negative allosteric modulator with respect to the stabilised conformation. Whilst the former stabilises the inactive conformation in the receptor core (around the PIF motif) and the intracellular G protein binding surface (similar to the equilibrium of conformations observed for the Apo receptor), the latter stabilises the alternative AS408-dependent conformation. We provide further evidence for this conclusion through the addition of AS408 to propranolol-stimulated receptor. In BLI assays, no mini-G_s binding was observed with AS408 addition (Supplementary Fig. 20e), and no significant changes were observed in the BRET efficiency for the conformational biosensor (Supplementary Fig. 31a). The key observation to support differences in mechanisms are the chemical shift changes observed with increasing AS408 addition in NMR spectra of propranolol-bound β_1AR-E (Supplementary Fig. 25c-g), which mirrored the changes in NMR spectra observed for apo β_1AR-E upon AS408 addition. These substantial shifts in signals clearly demonstrated that AS408 stabilised the same “non-productive” conformation when binding to antagonist-bound receptor as when AS408 interacts with apo or isoprenaline-bound receptor, and not the propranolol-stabilised inactive state. On the other hand, the extracellular region around the ligand binding pocket (informed on through the M178 NMR probe signals) is dependent on the binding of the antagonist (Supplementary Fig. 25f, the M178 signal does not change with AS408 addition and differs from the Isoprenaline-bound and apo signals for this probe in Fig. 4f). This is in agreement with our previous conclusion that the equilibrium in the orthosteric pocket is little impacted by AS408 and predominantly dependent on the orthosteric ligand. Therefore, we find no difference between ligand classes for the action of AS408, in that AS408 acts to stabilise the non-productive conformation on the intracellular surface of β_1AR-E irrespective of ligand identity in the orthosteric binding pocket whilst this pocket is predominantly dependent on the orthosteric ligand.

Can the authors clarify whether the human or turkey β1AR has been used throughout to explore the mechanism? Can the authors comment on the influence of species differences?

The turkey β_1AR has been used throughout the experiments in this manuscript, continuing on from our structural investigations published previously. The specific details of the constructs and the differences relative to the wildtype turkey β_1AR sequence are detailed in the methods section (β_1AR constructs – Biophysics and β_1AR constructs – Pharmacology). For clarity, we have amended the introduction to explicitly denote the different receptors: Introduction, Paragraph 4: "Recently, we showed that reversal of a thermostabilising E130W$^{3.41}$ mutation[45] to wildtype E130$^{3.41}$ shifted the equilibria of the turkey β_1-adrenergic receptor (hereon referred to as β_1AR) towards an active state[14]."; Introduction, Paragraph 5: "A recent study introduced AS408 as a NAM for the human β_2AR (hβ_2AR)[29]. …AS408 reduced β-arrestin recruitment at several other related receptors in addition to hβ_2AR, including the human β_1AR (hβ_1AR)." We have added references to the turkey β_1AR in the methods section, including "The turkey β_1AR BRET biosensor was based on the β_1AR-E sequence with additions as shown in Supplementary Fig. 29a." into the β_1AR constructs – Pharmacology section of the Methods.

The turkey construct was chosen to facilitate a structural analysis of the receptor by NMR spectroscopy. Due to the increased thermostability of the turkey β_1AR relative to the human β_1AR (Parker *et al.*, 1991), the turkey β_1AR has been utilised in a majority of structural characterisations for the β_1AR, including by crystallography (e.g. Warne *et al*., 2011), cryo-electron microscopy (e.g. Su *et al.*, 2020), and NMR (e.g. Isogai *et al.*, 2016) as higher yields and more stable protein can be obtained which aids with the measurements. These data have provided a wealth of knowledge and thus the turkey β_1AR is a useful model system to study GPCR signalling.

The human and turkey forms of β_1AR have ~68% sequence homology (excluding the N- and C-termini) and full conservation of microswitches including the PIF motif, CxWP toggle switch, NPxxY motif, DRY ionic lock, and Y$^{5.58}$-Y$^{7.53}$ lock. Therefore, we would anticipate that the energy landscapes of the two receptors would be relatively similar. Indeed, a recent NMR study investigating a stabilised variant of the human β_1AR demonstrated that the receptor populated the same equilibrium of inactive, pre-active, and active states, with their populations being modulated by the orthosteric agonist (Rößler *et al.*, 2025). The key observations in this paper were that the human β_1AR was more conformationally flexible and transitioned between inactive and pre-active states more readily, but the active state still exchanged slowly with the pre-active state. Consequently, the energy landscapes between the human and turkey β_1AR were comparable though differed slightly in relative stabilities and energy barriers between receptor states. As such, we anticipate that the equivalent E$^{3.41}$ mutations (E147$^{3.41}$ in human β_1AR) would have similar impacts on the conformational equilibrium of the human receptor as in the turkey receptor.

Liu *et al.*, 2020 showed AS408 acts as an allosteric modulator at the human β_1AR (Supplementary Figure 6 and Supplementary Table 4). The allosteric pocket differs at three positions (3.48, 5.45, and 5.49) between the turkey β_1AR and the human β_1AR, which are the same positions that differ between the turkey β_1AR and the human β_2AR. The 5.45 and 5.49 positions are isoleucine residues in the turkey β_1AR but are valine residues in human β_1AR. The 3.48 position, which interacts with the phenyl ring of the AS408, is an isoleucine residue in turkey β_1AR and a leucine residue in human β_1AR. We anticipate the larger residue side chain at the 3.48 position may impact the orientation of AS408, relative to the smaller valine residue at this position in the human β_2AR. However, we cannot rule out that the overall structural or dynamic differences could impact the mechanism of AS408 as differences between the human and turkey β_1AR energy landscapes may therefore impact the ability of AS408 to influence the conformation of the receptor beyond the allosteric pocket.

The primary aim of this study is to contrast different structural mechanisms by which receptor signalling can be modulated allosterically. Therefore, the turkey β_1AR has been used here as a tool to facilitate this investigation due to the wealth of pharmacological and structural data in the literature as well as how conducible this receptor is to biophysical examination, a key aspect of our investigation. Our data highlights how subtle differences in structures or conformational dynamics between receptors can have substantial impacts on the modulation of GPCR signalling and equilibria by small molecule binders. We aim to focus on the differential mechanisms of allosteric modulation that can be present in the same allosteric pocket, provided by the comparison of E130X$^{3.41}$ mutations and AS408 binding both at the turkey β_1AR, rather than a comparison between the human β_1AR and β_2AR. Further investigations into the impact of AS408 on the conformational dynamics of the human β_1AR represent an intriguing extension of the research in this manuscript but we feel are outside the scope of this study.

Figure 1: In relation to the pharmacological profiling and conformational equilibria of β1AR constructs bearing mutations at E1303.41, differences in receptor expression between assays may complicate interpretation.

We agree with the Reviewer that expression data is important to validate interpretations. We have assessed the expression of the E130X$^{3.41}$ variants via flow cytometry of FLAG-Nluc-β_1AR-X constructs expressed in HEK293T cells. We found no significant difference in expression between the E130$^{3.41}$ and the E130X$^{3.41}$ variants (Supplementary Fig. 6), indicating that the differences observed in ligand binding data or TRUPATH assays were not due to differences in receptor expression.

We are unable to determine surface expression of the untagged receptor, so instead chose to use a receptor construct used in the dataset (the FLAG-Nluc-β_1AR used to measure ligand affinities).

We further note that there is substantial precedent in the field for analysing receptor expression utilising the Nluc construct, such as in PMID: 33149304 - Marti-Solano, M., Crilly, S.E., Malinverni, D. *et al.* Combinatorial expression of GPCR isoforms affects signalling and drug responses. *Nature* **587**, 650–656 (2020), or PMID: 39073853 - Huang, X., Chorianopoulou, A., Kalkounou, P., Georgiou, M., Pousias, A.*, et al*. Hit-to-Lead Optimization of Heterocyclic Carbonyloxycarboximidamides as Selective Antagonists at Human Adenosine A3 Receptor. *J. Med. Chem.* ***67***(15), 13117–13146 (2024).

Finally, we note that the BLI data is independent of expression due to the purification of the protein required for this assay. The order of receptor activities obtained from the BLI assay is in agreement with the order obtained from the TRUPATH data, and consequently we use this data to support the conclusions made from the pharmacological data. We believe that validating our pharmacological assays with biophysical data measured with purified protein, alongside our supplementary experiments determining cell surface expression by flow cytometry, is sufficient to show that β_1AR expression in HEK293T cells is not a contributing factor to the differing activities of these receptors.

Throughout the manuscript, affinity and potency values must be reported and statistically analysed on a log scale, as only log-transformed data exhibit normally distributed errors.

We thank the Reviewer for highlighting this issue in the analysis of the data, and consequently we have reported the data in log-transformed graphs where appropriate and performed all statistical analyses on following log transformations of the data, which are reported in the text. We have also made these details clear either in figure legends or in the methods section, as appropriate.

Operational model of allosterism: authors must state and rationalise which parameters they have constrained and why.

We rationalised our choice of parameters used in the operational model of allostery as to facilitate a direct comparison with the analysis of AS408 action at the β_2AR as described in Liu *et al.*, 2020, in that we constrained parameters in the same manner in order to replicate their analysis for the turkey β_1AR. In the Supplementary Table 2 of that study, the constraints are stated: "τ_β and β-values were fixed at ~0 since AS408 is a NAM and has no agonist efficacy, respectively." Consequently, we have fixed our parameters similarly. We also fix the Hill coefficient at 1.0. We have subsequently added an additional table to the Supplementary Information file (Supplementary Table 3) which includes these details, and we have added a short section to the methods (entitled "Operational model of allostery") to further detail these analyses.

Can authors comment on the observed difference in Log alpha across assays? Authors must also rationalise the observed (but not reported) difference in beta values (efficacy modulation).

We speculate that the variation in the α values is likely a result of the differing assays utilised to obtain these values. The biosensor directly reads out the conformational change in the receptor induced by the agonist co-stimulated with AS408, whereas the TRUPATH assay responds to G protein activation and is therefore an indirect measure of changes at the receptor.

We further note that the log(α) value was observed to vary at the β_2AR: -0.7 +/- 0.5 for [^{35}S]GTPγS binding; -0.1 +/- 0.1 for cAMP accumulation; and -0.2 +/- 0.1 for β-arrestin recruitment (Liu *et al.*, 2020). The differences here were attributed to "better access of the modulator to the receptor in broken cell preparations (as in the [^{35}S]GTPγS binding assays) compared to the whole cell assays (cAMP and β-arrestin recruitment assays)". Consequently, variations in log(α) values may not be entirely unsurprising between different experimental systems.

We have now clarified in the methods section and in Supplementary Table 3 that the β values are held constant at near 0 in the operational model fits.

An important missing element is the assessment of how mutations and AS408 affect the ligand-binding kinetics of orthosteric ligands.

We thank the Reviewer for raising this important question and we have now conducted Nluc ligand binding assays, as suggested by the Reviewer, to address these questions. We find that there are no significant differences in either the association or dissociation rates of both (S)-propranolol-red and isoprenaline between the E130X$^{3.41}$ variants. Therefore, we find that the ligand binding kinetics will have little impact on the interpretation of the BLI assays assessing mini-G_s binding to the receptor variants. These binding kinetics have been added to the manuscript in Supplementary Figure 3-4.

We have further recorded association or dissociation rate constants of both (S)-propranolol-red and isoprenaline to β_1AR-E in the presence of varying AS408 concentrations. These data support our previous observations in that AS408 has little effect on the affinity to (S)-propranolol-red but increases the K_i for isoprenaline binding. This increase is a result of dose-dependent decreases in association rates for isoprenaline binding, whereas we observed no significant changes in k_{off} rates for isoprenaline nor any significant changes in k_{off} or k_{on} rates for (S)-propranolol-red binding. The data for these kinetic assays have been added to the manuscript in Supplementary Figure 26-27.

The authors state "The ratio between the areas under the curve (AU C) for the active state (A) against the pre-active state (P) indicated that unlike β1AR-E, which primarily occupied the active state…..". Could the authors please amend the extra space in AUC. Does the data Fig 2C support this claim? … Further to this, can the authors comment on whether it is surprising that saturating concentrations of isoprenaline did not confer greater stabilisation of active states vs pre-active?

We were unable to find the aforementioned extra space in the text.

As the Reviewer highlights, there are substantial differences between the populations of the active and pre-active states between the β_1AR-E and receptors with E130X$^{3.41}$ mutations, which was the essential observation to support our conclusions with respect to these NMR studies. We determined that the population of the active state is greater than the relative population of the pre-active state in β_1AR-E, as measured by the area under each signal (AUC) in the deconvoluted ^{19}F NMR spectrum. We have changed the text (Results, Section 1, Paragraph 8) to "Unlike β_1AR-E, which favours occupation of the active state…" which we now feel better reflects the data.

There has been substantial investigation into the conformational dynamics of GPCRs by NMR spectroscopy, which have revealed that binding of agonists with differing efficacies stabilise the functional states (inactive/pre-active/active states) to varying degrees. Our investigations into the turkey β_1AR have found that the receptor populates an equilibrium of active and pre-active states when saturated with a full agonist (Jones *et al.*, 2024). The populations of these states were shown to be temperature dependent, with the active state being further populated at a higher temperature (308 K) than at the reduced temperature (298 K) as shown in Figure 2 in this manuscript. We emphasise that the lower temperature was utilised in this case due to the increased separation between the ^{19}F NMR signals corresponding to the active and pre-active states relative to the spectra measured at the higher temperature. Therefore, the lower temperature aided in a more reliable assessment of the relative populations of active and pre-active states. We also emphasise that GPCR equilibria are highly sensitive to the system used: the detergent micelle composition, the degree of thermostabilisation, and addition of lipids have been demonstrated to alter the relative populations of the agonist-bound receptor states. NMR studies from other groups have also demonstrated that populations of several states are observed even at saturating concentrations of full agonists. Examples include other work at the β_1AR (Grahl *et al.*,

2020, Wu *et al.*, 2025), the β_2AR (Manglik *et al.*, 2015), the A_{2A} adenosine receptor (Sušac *et al.*, 2018, Huang *et al.*, 2021), the neurotensin receptor (Asadollahi *et al.*, 2023), the µ-opioid receptor (Kaneko *et al.*, 2022), and the cannabinoid receptor (Wang *et al.*, 2021), which are all cited in the introduction. Therefore, agonists consistently stabilise multiple populations and commonly do not alter the energy landscape to fully populate the active state. The extent to which this phenomenon is present in GPCRs is also highly dependent on the GPCR which is investigated and has been linked to differences in receptor signalling and basal activity between receptor subtypes. Notably, the stabilisation of several conformational states at equilibrium by a full agonist has been suggested as possible method to increase receptor signalling with positive allosteric modulators which would increase stabilisation and thus population of the receptor active state. This hypothesis has proven accurate in investigations by Kaneko *et al.*, 2022, in which the PAM BMS-986122 shifted the µ-opioid receptor equilibria in the presence of agonists DAMGO or morphine further towards the active state than either orthosteric ligand was able to stabilise.

Consequently, the ability of agonists to stabilise several populations at equilibrium is a well-established phenomenon with potential therapeutic applications, and hence our findings with respect to the relative active/pre-active state stabilisation for β_1AR-E are consistent with the GPCR structural biology field.

Can the authors please clarify if a ligand was used in the MD simulations?

Subject to comments from Reviewer #3, we have now performed additional MD simulations which represent a better analysis into the impacts of E130X$^{3.41}$ mutations on receptor deactivation pathways. Please see the response to Reviewer #3 for the details of these simulations. In both these revised simulations and the original submitted simulations, the agonist isoprenaline was retained in the orthosteric binding pocket.

Figure 3a: AS408 interaction with isoprenaline appears consistent with a competitive interaction. Can the authors detail the pharmacological evidence for AS408 acting as a negative allosteric modulator at the β1AR, as currently this is unclear.

In the TRUPATH assay data shown in Figure 3a, we agree that the limited response to isoprenaline at the highest AS408 concentration (100 µM) could be consistent with a competitive interaction. However, a likely possibility is that the full curve is not present and a higher concentration of isoprenaline would be required to obtain full dose-response curves (i.e. AS408 may shift the potency for isoprenaline further to the right). A similar effect is also observed for β_1AR-F and β_1AR-Y TRUPATH data (Figure 1e) in which full concentration response curves are not observed. Additional pharmacological evidence to suggest that AS408 acts allosterically, rather than competitively, includes the absence of significant changes in the affinity of the antagonist (S)-propranolol-red in the presence of the AS408. We would anticipate that at 100 µM AS408, we would observe competition of the antagonist and AS408. Consequently, the pharmacological data points towards an allosteric mode of action.

We also note that other structural data indicates that there is no competitive interaction between the orthosteric ligand and AS408 at βARs. The AS408-β_2AR crystal structure in Liu *et al.*, 2020, demonstrated

binding into an allosteric binding pocket, and our complementary NMR data were consistent with binding of the AS408 into this same pocket at the turkey β_1AR (Fig. 3g-h).

Furthermore, other NMR data indicates that AS408 does not bind into the orthosteric pocket. E.g. the M178 probe, which reports on the conformational dynamics and ligand identity in the orthosteric pocket, shows little to no changes in the apo spectra of β_1AR-E (M178, Fig. 4e), β_1AR-M (Supplementary Fig. 35d), β_1AR-F (Supplementary Fig. 33d), and β_1AR-Y (Supplementary Fig. 33b). Additional NMR data recorded for the propranolol-bound β_1AR-E (Supplementary Fig. 25c,f) clearly demonstrated that the unique position that M178 signal occupies when bound to the antagonist is strongly maintained upon the addition of AS408, whereas other signals clearly respond in the expected manner when the receptor populates the AS408-stabilised 'non-productive' conformation. The M178 signal position remains consistent with the propranolol-stabilised chemical shift position, which notably differs from the two positions for M178 in the apo receptor with addition of AS408. Therefore, these data clearly evidence AS408 binding and related changes in conformations and dynamics whilst also distinctly demonstrating propranolol binding to the orthosteric pocket.

Finally, the supplementary 1 µs simulations (recorded in triplicate) of β_1AR-E with the AS408 modelled in the β_2AR binding pose (Fig. 3d-f and Supplementary Fig. 22) further support an allosteric binding mode. In these simulations, AS408 is maintained in the TM3-TM4-TM5 binding pocket, although interacting with different regions of the allosteric pocket. Hence, these data also support AS408 as a negative allosteric modulator at the β_1AR.

Overall, the data indicating that AS408 does not displace the fluorescent tracer ((S)-propranolol-red) alongside the structural dataset solidifies our conclusion that AS408 acts as an allosteric modulator at the TM3-TM4-TM5 pocket and does not compete for binding in the orthosteric pocket.

Figure 4d: The same concentration of (S)-propranolol-red must be used for the binding interaction studies evaluating the allosteric modulation of AS408 against isoprenaline and (S)-propranolol-red.

We thank the Reviewer for this important point and have now amended the assays with new measurements in which equal concentrations of (S)-propranolol-red were used. The data are shown in Supplementary Figure 26 and demonstrate that the trend which was originally reported was maintained with this improved analysis. We also have provided the binding kinetics of (S)-propranolol-red, which further show there are no significant changes in the k_{off} rates of the antagonist with changing AS408 concentration, and only a significant decrease in k_{on} at 100 µM AS408.

Can the authors please explain the large discrepancy between maximal AUC values for isoprenaline in Supplementary Fig 23B (value of ~7) and Fig 6C (value of ~1.5).

The discrepancy between the two figures was due to differences in equipment used in measurements. The former was recorded with a BMG Labtech PHERAstar plate reader and the latter was recorded with a BMG Labtech CLARIOstar Plus® microplate reader. We have now altered Supplementary Fig. 29 so that all presented data is consistent and recorded using the CLARIOstar Plus® microplate reader.

In supplementary figure 23, kinetic traces are shown for isoprenaline and propranolol but not AS408. Additionally, AS408 concentration response curves (in the absence of orthosteric ligand) are not shown. Both kinetic traces and curves for AS408 should be performed and shown.

We have now provided the kinetic traces and curves for AS408 data in Supplementary Figure 30b-c, which demonstrate that AS408 alone has little impact on the BRET-sensor measurements.

Figure 6: Have the authors (or others) completed full interactions between isoprenaline and propranolol with this biosensor at the β1AR? Validation of the biosensor with an orthosteric antagonist is ideal and will confirm that the sensor is capable of discerning competitive interactions.

We agree with the Reviewer that the work with our biosensor, which (although modelled on previous biosensors used in the field, please see citations in the main text) was developed for this research, would benefit from showing further validation. Therefore, we show this validation through a competition assay between an agonist (isoprenaline) and an antagonist (propranolol) in Supplementary Fig. 30a. These data demonstrate the reduction in BRET changes upon addition of propranolol in concentration response curves for isoprenaline assays, and confirm that propranolol can compete for the orthosteric binding pocket to discern competitive interactions.

Fig. 1e: The isoprenaline curve for β1AR-E is fit with a one-site model, but the data visually resemble a two-site fit. Can authors compare the preference for a one and two site fit?

We agree with the Reviewer that the data shown in Fig. 1e could be consistent with a two-site fit, we suggest that this is an artifact of the experimental system. The HEK293T cells used in TRUPATH experiments are known to endogenously express several adrenergic receptors, including the β_2AR and to some extent the β_1AR (e.g. Such expression is commented on for HEK293 cells in: Ferrie AM, et al., Divergent label-free cell phenotypic pharmacology of ligands at the overexpressed β_2-adrenergic receptors. *Sci Rep*. **4**:3828. (2014)). We hypothesise that the biphasic behaviour observed in the TRUPATH data may be linked to these endogenously expressed receptors, in that additional activation of endogenously expressed receptors at the higher concentrations of isoprenaline increases the signal at the concentrations but not the lower concentrations. It is worth noting that the only curve which shows this is the β_1AR-E and likely arises from experimental variation. Finally, it is well established that there is only a single binding site for isoprenaline at the β_1AR. We cite the existing literature for this agonist which has been extensively characterised both pharmacologically (e.g. Baker 2010) and structurally (including PDB: 2Y03 (isoprenaline-bound β_1AR crystal structure), 7JJO (isoprenaline-β_1AR-G_s complex), 7S0F (isoprenaline-β_1AR-G_i complex), 6H7J (isoprenaline-β_1AR-Nb80 complex), 8S2T (isoprenaline-human β_1AR-G_s complex).

Supplementary Fig. 5c: the association kinetic fits do not appear to pass through the data points at higher Mini-Gs concentrations (500 nM, 1000 nM, 2000 nM). The mismatch at the kinetic inflection point will influence the predicted association rate, so it should be addressed.

We have now re-analysed the BLI data, excluding the 2000 and 1000 nM data points. The resulting analysis gives good fits for the data, which are shown in Supplementary Figure 8. The trends observed between the different mutations were maintained and significant differences were still observed when log-transformed values were analysed by one-way ANOVA with a post-hoc Dunnet's test. We note that the kinetic parameters were obtained using a "1:1 Global fit". In this analysis, kinetic parameters are obtained which provide the best fits for all curves, thus removing any bias from concentration dependence and providing a more robust and accurate measure of the binding affinity. As such, the kinetic fits are the most optimal for all concentrations, which can result in some small deviations in some concentrations. We believe that the re-analysed data using the range 31 nM - 500 nM is of high quality, with good fitting to the 1:1 model.

Supplementary Fig. 5c: Mini-Gs kinetics form a central component of the mechanistic interpretation. Authors must include the kinetics for all datasets and fits, including mutations and the allosteric modulator.

We have now provided example BLI curves of each dataset and their corresponding fits for each mutation (i.e. for the data in Fig. 1f-g) and the BLI data of β_1AR-E with the allosteric modulator. This data is shown in Supplementary Fig. 8 (E130X$^{3.41}$ variants) and Supplementary Fig. 18d (AS408).

In Figure 6 legend, the authors state "Data fitted using the 3 parameter logistic equations except for the 100 µM AS408 which was fitted with a simple linear regression curve due to the lack of discernible agonist activation." Greater clarity is required with respect to this statement.

The data in Figure 6c with respect to 100 µM AS408 has no appreciable change in AUC consistent with activation (due to the action of AS408) and therefore cannot be fit with a 3 parameter logistic equation. We had therefore utilised a simple linear regression (shown as a dashed orange line) to provide a guide to the reader as to the data, and not as an appropriate model to fit the data. We recognise that this has generated some confusion and, as this data cannot be accurately modelled, we have elected to remove the data for this concentration from the figure.

The authors are quantifying allosteric interactions by fitting the operational model of allosterism, but these fits are not shown in Fig 6c. The operational model of allosterism fit should be shown, as this is required for data interpretation.

The fits for the operational model of allostery for both the TRUPATH and biosensor data are now shown in Supplementary Fig. 19a and 19b, respectively.

Supplementary Table 2. Can the authors comment on why t-tests were used in this instance? Each mutant is compared to wildtype, therefore, wouldn't a One-Way ANOVA with Dunnett's post-hoc test be more appropriate?

We initially used t-tests to enable comparisons between each mutant. However, we agree with the Reviewer and have now conducted a One-Way ANOVA with a Dunnett's post-hoc test on log-transformed data to demonstrate the significant differences between the β_1AR-E and each receptor variant. Significant differences were observed, hence maintaining our conclusions. The *p* values can be found in Supplementary Table 2.

Minor Comments:

To strengthen the mechanistic understanding of receptor modulation, authors could include additional datasets examining Mini-Gs kinetics and affinity in the presence of antagonist, the allosteric modulator alone, and the modulator together with isoprenaline.

The data for mini-G_s binding kinetics to β_1AR-E in the presence of modulator with isoprenaline are shown in Supplementary Fig. 18 and Fig. 3b-c. Due to low basal activity of the β_1AR-E construct, the BLI data for binding of the apo receptor to mini-G_s in the presence of AS408 did not yield reliable kinetic data, but clear decreases in the BLI signal intensity were observed, as shown in Supplementary Fig. 20a (moved from Fig. 3d) where R_{eq} is representative of the amount of mini-G_s bound to the immobilised β_1AR-E on the biosensor.

We further recorded BLI data examining the mini-G_s kinetics to β_1AR-E in the presence of the antagonist propranolol, which demonstrates that there is negligible binding of mini-G_s to the receptor in the presence of the antagonist. The negligible binding is also observed in the presence of antagonist and increasing AS408 addition. The data are shown in Supplementary Fig. 20c-e.

Fig. 1a legend currently presents the schematic as fact, but it should be made clear whether the mechanism is known or proposed.

The mechanism shown in Fig. 1a is well-established in class A GPCRs in the literature, from a range of solution NMR studies on several receptors including the β_1AR. These studies (Ref 14-25) are cited in the introduction (Paragraph 3) in the line: "Structural studies indicate GPCRs sample several distinct states connected via tuneable equilibria, including inactive, pre-active, and active states[14–25] (Fig. 1a)." We note that additional states can also be present, but the states shown in Figure 1a are specifically relevant to the mechanism of activation at the β_1AR, as demonstrated in our previous work (Jones *et al*., 2024) as well as the work of others (e.g. Wu *et al.*, 2025). Thus, this mechanism is not being proposed in this study and has been well supported by previous research. For clarity, we have amended the figure legend: "The equilibria are supported by NMR studies[14-25]."

Figure 2: The percentage population of active/pre-active states for the hydrophobic residues is shown in panel 2C. However, for the aromatic residues, only W is shown. Can the authors comment on why W is in panel C but there are no corresponding 19F NMR spectra shown?

The ^{19}F NMR data for β_1AR-W was reported in one of our previous publications (Frei *et al.,* 2020), and therefore we have not replicated these spectral data in this work. In this work, we observed that the β_1AR-W construct populated the pre-active state, consistent with ^{13}C data published in another previous publication (Solt *et al.*, 2017). We have amended the figure legend to make this clear: "The population of the pre-active state for the β_1AR-W variant was previously determined in Frei et al., 2020[26].". We have added a further addition to the text to improve clarity: "… the hydrophobic variants predominantly occupied the pre-active state as previously observed for isoprenaline-bound β_1AR-W[26]."

Figure 2: Can the authors comment on whether the stabilisation of these pre-active states for the different mutations would lead to reduction in potency and/or Emax in the TRUPATH assay? Can they link these findings to their functional data presented in Fig 1e?

The work in this manuscript and in previous work both in our group (Jones *et al*., 2024) and others (Abiko *et al*., 2022, Wu *et al*., 2025) have demonstrated that the affinity of intracellular binding partners, including the G protein mimetic mini-G_s and the active state stabilising nanobodies Nb80 and Nb6B9, were decreased by stabilisation of the pre-active state, relative to the active state. Given the E_{max} in TRUPATH assays is dependent on the equilibrium population of G proteins which have dissociated Gα and Gβγ subunits, we anticipate that the E_{max} of receptors with E130X$^{3.41}$ variants would be reduced relative to β_1AR-E because receptors in the pre-active state would be less efficient at stimulating GDP/GTP exchange when saturated with isoprenaline than receptors populating the active state to a greater extent.

The potency is determined by both the ligand affinity for the receptor and the efficacy of the ligand-receptor complex. Although β_1AR-F and β_1AR-Y have reduced isoprenaline affinities relative to β_1AR-E, the differences are smaller than the change in potencies, which shift by several orders of magnitude (Fig. 1e). Therefore, we account for the changes in potency through the differences in the ligand-receptor efficacy. Ligand efficacy at GPCRs has been proposed to be encoded in the different receptor states populations stabilised by the ligand in a number of solution NMR studies (see Jones et al., 2024, Eddy et al., 2021, Frei et al., 2020, Grahl et al., 2020, Imai et al., 2020, Solt et al., 2017, Isogai et al., 2016, as examples, cited in the Introduction). In these studies, the receptor state populations correlate with the efficacy of the bound ligand, in that higher efficacy ligands stabilise more active receptor states to a greater extent than lower efficacy ligands. At the β_1AR-E, we have previously shown that β_1AR partial agonists (lower in efficacy than the full agonist isoprenaline) stabilise the pre-active state (published in Jones et al., 2024) which is less active with respect to G protein coupling compared to the active state. In this research, we also showed that the potency for activation of β_1AR by partial agonists is shifted to the right.

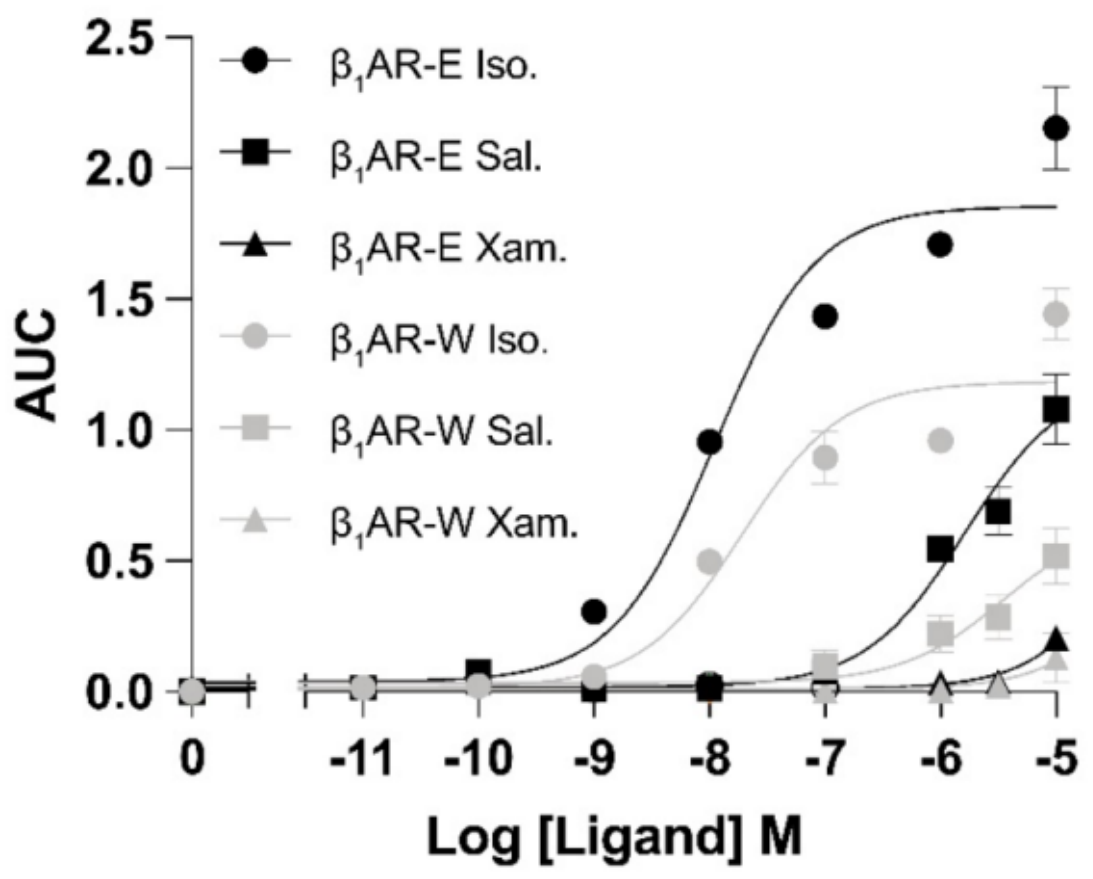

Supplementary Figure 14b from our research published in Jones et al., 2024. The TRUPATH assay system was applied for G_s activation for the partial agonists salbutamol (Sal., squares) and xamoterol (Xam., triangles) relative to full agonist isoprenaline (Iso., circles), comparing both β_1AR-E (black) and β_1AR-W (grey). Data are mean ± SEM of n=3 independent biological experiments, each performed in duplicate.

Differences in the efficacy between different low efficacy ligands can also be described in terms of the pre-active/inactive equilibrium. Our previous work has demonstrated that changes in the populations of the inactive and pre-active states correlate with partial agonist efficacy at the β_1AR**.** For example, in the panel above, the partial agonist xamoterol is a weaker partial agonist than salbutamol, and therefore the EC_{50} is shifted further to the right. This is reflected in the receptor equilibria, as xamoterol stabilises a smaller population of the pre-active state (and thus a greater population of the inactive state which has poor G protein coupling) compared to salbutamol.

As we have detailed in the main text, the E130X$^{3.41}$ variants act by stabilising different states in the existing equilibrium of β_1AR functional states, including the active, pre-active, and inactive states (Fig. 1a). The variants can therefore be described to act by altering the effective efficacy of the ligand: at the hydrophobic E130X$^{3.41}$ receptors, isoprenaline can be described as acting as a high efficacy partial agonist (relative to isoprenaline stimulation at the β_1AR-E) as the receptor population stabilised by the ligand is shifted away from the active state and predominantly stabilises the pre-active state; at the aromatic E130X$^{3.41}$ receptors, isoprenaline can be described as acting as a low efficacy partial agonist because the receptor population is shifted further towards the pre-active and inactive states. The fractional efficacy imparted on the full agonist results in the changes in potencies observed in the functional data.

We have added the following line to the Discussion (Paragraph 2) to reflect this comment: "Hence, the mutations at X$^{3.41}$ regulated transduction of ligand information in a residue-type dependent manner by effectively imparting fractional efficacy effects on the full agonist to control β_1AR signalling (Fig. 8a), resulting in the changes in E_{max} and potency observed in functional data (Fig. 1e)."

Figure 2: Can the authors please include the concentration of isoprenaline (1 mM) used in these NMR experiments, either in the text and/or figure legend.

We have now included the concentration of isoprenaline in figure legends and it is also stated in the NMR methods section.

For the E130I mutation, there is binding data (Fig 1d), functional data (Fig 2e) and MD simulation data (Supp Fig 10, 12). Why is there no corresponding NMR data for this mutation?

We had not recorded NMR data for the $E130I^{3.41}$ mutation as we found that the E130V/M/L variants were sufficient to support our mechanistic understanding of the E130X hydrophobic mutations. However, we have now recorded ^{19}F NMR and BLI data for this variant, which have been added to the figures and text. The NMR data fit the expected trend. The BLI data demonstrates a reduction in the binding affinity of E130I for mini-G_s (Fig. 1f) which corresponds to predominant population of the pre-active state (Fig. 2b-c), similar to the other E130X hydrophobic mutations and thus supports the conclusions made with the other E130X hydrophobic variants.

Supplementary Figure 10: The different shaded bars in Supplementary Figure 10 are very hard to visualise. Different colours would make them easier to see.

As we have now completed additional simulations (See reply to Reviewer #3), Supplementary Fig. 10 has been removed from the manuscript. Please see Fig. 2e-f, 3d-f and Supplementary Fig. 14-17, 22 for the updated simulations.

Figure 3c "Req (response values from 1:1 global fitting with Rmax unlinked by sensors) for mini-Gs binding to isoprenaline-bound β1AR-E modulated by AS408, measured by BLI. Data are mean ± SEM of n=3 independent technical replicates." Can authors provide additional details as to what Req means? This is currently unclear.

In the Application Note provided by Sartorius, "Biomolecular Binding Kinetics Assays on the Octet® BLI Platform" Dec 2022, the point at which the BLI signal plateaus (i.e. at equilibrium when the rate of analyte binding to the tip is equal to rate of analyte dissociation) is "the equilibrium binding signal, or R_{eq}". This value is a measure of the relative amount of analyte bound which is associated to the ligand on the biosensor. This value is taken from the modelled fit, rather than the raw data. Consequently, we have amended the figure legend as follows: "R_{eq} (the equilibrium binding signal, a measure of bound analyte, obtained using a 1:1 global fitting with R_{max} unlinked by sensors)".

Figure 3d: Authors state these data are "fit with [Inhibitor] vs Response (three parameter variable slope), with a Hill constant fixed at -1", can the authors confirm this is correct, as the fit does not seem to be a 3-parameter sigmoidal concentration-response.

We agree that the fit is not appropriate for this data and therefore we have removed the fitting, leaving the individual data points.

Figure 4e: Reader interpretation would benefit from reporting AS408 affinity and cooperativity values, as opposed to changes in isoprenaline affinity in the presence of AS408.

The NMR data in Figure 4c suggested that the orthosteric binding pocket remained in the active state when the receptor was saturated with AS408. However, the broadening of the signal suggested a change to the conformational dynamics on the µs-ms timescale. We hypothesised from this observation that this broadening was due to changes in the isoprenaline binding affinity which would directly impact the linewidth of the M178 signal in the NMR spectra. Therefore, Fig. 4d-e (now just Fig. 4d) are a direct response to this hypothesis generated from the NMR data. We find with our additional ligand binding kinetics data that there was a decrease in affinity driven primarily by decreases in ligand association rates, thereby linking the NMR and pharmacological data.
Therefore, we find that the AS408 affinity has no bearing on this specific question arising from the NMR signal M178. We do recognise that the cooperativity value is pertinent to this question, and therefore we have modified the appropriate paragraph (Results, Section 3, Paragraph 4:

"We noted that the intensity of the $M178^{4.62}$ active state signal decreased with increasing AS408 addition due to peak broadening (Fig. 4c), which suggested that AS408 reduced the binding affinity of isoprenaline to β_1AR-E. In cell ligand affinity measurements corroborated this conclusion (Supplementary Fig. 26-27), in which AS408 induced a significant ($p < 0.05$) concentration-dependent reduction in the isoprenaline affinity to β_1AR-E (Fig. 4d, Supplementary Table 4). This decrease was consistent with the cooperativity value of $\log(\alpha) = -2.1$ obtained from analysis of the TRUPATH data (Supplementary Fig. 19a)."

The authors state "Consistent with similar intramolecular sensors58–60, we observed a dose-dependent loss of BRET signal for isoprenaline (pEC50 – 8.16 ± 0.3) with very little change upon application with propranolol (Supplementary Fig. 23b), validating our β1AR-E BRET-sensor." Please remove the negative value or alternatively just state the EC50. Can the authors state which data set was used to source this EC50 value? The concentration response data for isoprenaline in the absence of AS408 does not visually yield a pEC50 8.16 in Fig 6C or Supplementary Fig 23B.

We have changed the dash to a colon to remove ambiguity. We apologise for the error in reporting the EC50 value. The value quoted here has not been updated. This value should read "pEC_{50}: 7.74 ± 0.17". We have corrected the main text with this updated value.

Fig 1e: The colour scheme used to differentiate the V and W mutations is difficult to interpret because both are displayed in orange.

We have adjusted the colours for β_1AR-V to a darker brown colour to improve the contrast between the V and W mutations. The original colours were taken from the Okabe Ito palette for colour blind friendly qualitative colour schemes.

Supplementary Figure 20: It appears that an increase in Bmax occurs with increasing concentrations of AS408. Although possible, this would be an unusual finding – can authors provide a comment and explanation for this?

We agree with the Reviewer that this observation would indeed be unusual. However, we believe that this result is likely an artifact of the experimental system. The data shown in Supplementary Fig. 20b are a ratio of fluorescence/bioluminescence, and consequently are susceptible to interference by fluorescent ligands. The AS408 molecule, which has several delocalised π systems, appears to have some low-level fluorescence which has small effects on the measurements such as absorbance or quenching effects at wavelengths relevant to this experiment. The effect on the ratio is concentration dependent and thus has the greatest effect at the 100 µM AS408. In addition, the impact is exacerbated by the ratio which can artificially inflate errors. We therefore anticipate that the result is increased variability in the fluorescence/bioluminescence ratio which can result in misleading observations such as the change in B_{max} here. In support of this observation, our new dataset that was recorded to obtain the kinetic parameters for ligand binding in the presence of AS408 demonstrates that there is now observed a decrease in the B_{max} at the highest concentration of AS408 (Supplementary Fig. 26b). Consequently, we put this observation down to ligand-dependent artifacts rather than any real effect.

Results section, 3rd paragraph: The statement "We found little significant differences between wildtype β1AR-E (2.13 µM) and hydrophobic mutants or β1AR-W ($p>0.05$)." should instead state that no significant differences were detected.

We agree with the Reviewer and have now amended the text as suggested.

Reviewer #3

We thank the Reviewer for their strong endorsement of our experimental framework and their thorough and helpful review of the MD simulations. We agree with the Reviewer that the standard of our simulations fell short compared to the other sections of our manuscript, and hence required a more rigorous approach. We decided to keep a computational component in our revised work, and therefore we have recorded a new set of simulations, guided by the recommendations of the Reviewer. In particular, we examined the deactivation pathways of the β_1AR with E130X$^{3.41}$ mutations or in the presence of AS408 based on longer simulations starting from an active state structure. These simulations utilised a receptor sequence which reflected the experimental constructs used in our manuscript, and we analysed the simulations by monitoring canonical GPCR interactions to provide a stronger basis towards a mechanistic interpretation.

Accordingly, we made amendments to the preparation of the MD simulations in line with the suggestions from Reviewer #3. The simulations were conducted using the β_1AR structure obtained from PDB ID: 7JJO (isoprenaline-bound β_1AR in complex with G_s) as a mimetic of the active state (Comment #1). The G protein and stabilising nanobody Nb35 were removed, leaving the isoprenaline-bound receptor in the simulations. Production runs were recorded for 1 µs (Comment #2), in triplicate, for each variant and totalled 24 µs (i.e. 8 variants x 3 simulations). The receptor sequence was also adjusted to match the exact β_1AR sequence as shown in Supplementary Fig. 1a (Comment #4), and thus is identical to the sequence used in BLI assays, ^{1}H-^{13}C NMR experiments, and TRUPATH/ligand affinity assays (aside from any additional N-terminal or C-terminal tags which were not included). We agree that at pH 7.0 the E130 position would likely be protonated and, accordingly, simulations were run as such (Comment #5). We also appreciate the concern raised about using distance-based measurements (Comment #3), and in the revised analysis we now report explicit chemical interactions (hydrogen bonds, Van der Waals, hydrophobic, etc) using the ProLIF package. Finally, we have made the MD trajectories publicly available in Zenodo (Comment #6), and the link can be found in the Data Availability Section in the Manuscript. We have also added Figures detailing the RMSD and new poses of AS408 in Fig. 3d-f and Supplementary Fig. 22.

Evaluation of E130X$^{3.41}$ variants

The simulations were analysed to assess the key activation markers (including the PIF motif, Y$^{5.58}$-Y$^{7.53}$ distance, ionic (DRY motif), hydrophobic (L$^{3.43}$, I$^{6.40}$, M$^{6.41}$) lock formation and NPxxY motif) as suggested by the Reviewer, as well as the differing interaction types in the allosteric pocket. Our analysis showed that in all systems, the canonical activation microswitches generally remained consistent with an active-like conformation over the 1 µs timescale of our simulations, with the hydrophobic and ionic locks remaining open and the PIF motif not sampling an inactive-state conformation. The Y$^{5.58}$–Y$^{7.53}$ motif was most susceptible to disruption, with some replicates showing transient excursions towards inactive-like distances, consistent with the known plasticity of this region. We note that full deactivation transitions are expected to occur on timescales longer than 1 µs and thus we use our simulations primarily to report on the local interactions of residue X$^{3.41}$ rather than complete conformational transitions between active and inactive states. We have reflected this use in the manuscript (Results, Section 1, Paragraph 12): “These simulations implied that the structural transitions which resulted in the differing conformations observed by NMR occurred on a longer timescale than in our simulations, and hence we focused on the local dynamics of the TM3-TM4-TM5 allosteric pocket.”

In our analysis of the differing interactions found between the $X^{3.41}$ position and the allosteric pocket, we observed a similar trend as in our previous simulations in that aromatic $E130X^{3.41}$ variants tend to interact to a much greater extent with residues in TM5 than other residues. Although we do not see the transitions between active and inactive states within the simulations, we postulate that interactions between aromatic $E130X^{3.41}$ residues and TM5 residues $P^{5.50}$ and $I^{5.45}$ would result in increased stabilisation of the inactive state once the receptor has collapsed to this resting energy level. In this sense, these mutations would act similarly to the mechanism proposed for the AS408 at the β_2AR, which strengthens our confidence in this hypothesis.

We have adapted our manuscript to include the data recorded from these 24 µs of simulations. We include an analysis of interactions between the $X^{3.41}$ residue in each simulation and the surrounding residues in the allosteric pocket in Figure 2e-f and Supplementary Fig. 16-17, and Supplementary Fig. 14-15 for the analyses of canonical activation microswitches in addition to confirmation of orthosteric ligand binding interactions.

Evalutation of AS408 binding

We further recorded 1 µs simulations in triplicate of the isoprenaline-bound β_1AR-E structure in complex with AS408. The starting structure preparation (from PDB ID: 7JJO) followed the same procedure as the isoprenaline-bound β_1AR-E simulations in the above section. AS408 was modelled into the TM3-TM4-TM5 binding pocket from the pose found in the β_2AR crystal structure (PDB ID: 6OBA), by aligning the β_1AR and β_2AR structures in ChimeraX™.

Canonical activation microswitches remained broadly active-like across all three simulations (Supplementary Fig. 14), indicating that AS408 did not induce full receptor deactivation on this timescale and consistent with the observations in MD simulations for the similarly inactivating β_1AR-F and β_1AR-Y. We found that the β_2AR-derived starting pose of AS408 was unstable in all three replicates, with the allosteric ligand RMSD increasing sharply within the first 50–100 ns (Fig. 3d). This is consistent with the sequence differences at positions 5.45 and 5.49, where bulkier isoleucine residues in β_1AR replace the valines found in β_2AR. Following departure from the initial pose, AS408 relocated to an alternative position at the same TM3–TM4–TM5 interface but shifted closer to TM4, establishing hydrogen bonds with $E130^{3.41}$ and hydrophobic/Van der Waals contacts with $I137^{3.48}$, $M153^{34.47}$, $I161^{4.45}$, and $I168^{4.52}$ (Fig. 3e, Pose 1, and Supplementary Fig. 22b, Replicates 1 and 3) This binding mode was maintained for the remainder of the simulation in two replicates. In the third, AS408 additionally sampled a secondary position closer to the intracellular end of TM4 and intracellular loop 2 (Fig. 3e, Pose 2, and Supplementary Fig. 22b, Replicate 2), likely reflecting the conformational flexibility expected given the weak binding affinity obtained experimentally. Interestingly, these data also matched observations in NMR data we had already recorded, in which patterns in the changes of NMR signals present in the allosteric pocket, including $I168M^{4.52}$ and $M153^{34.57}$, indicated the potential for multiple binding modes. Consequently, we find that these additional simulations strongly support our experimental observations for AS408 acting as a negative allosteric modulator at the turkey β_1AR. The new binding poses relative to AS408 binding at the human β_2AR also implies the differing interactions, and shift away from $I214^{5.45}$ and $P219^{5.50}$, could be responsible for the differences in mechanisms between the two receptors.

We believe that both the improved $E130X^{3.41}$ simulations and AS408-β_1AR simulations present a much more substantial contribution to the mechanistic narrative that we are presenting in this investigation. We have made the MD trajectories publicly available in Zenodo (Comment #6), and the link can be found in the Data Availability Section in the Manuscript. We have also added Figures detailing the RMSD and new poses of AS408 in Fig. 3d-f and Supplementary Fig. 22.

Specific responses to Reviewer main remarks 1 - 6

1) According to the Methods, the authors started MD simulations from the agonist-bound turkey β1AR structure (PDB 2Y03), which Warne et al. (2011) showed to remain an inactive, non-signalling conformation ('None of the structures show the outward movement of the cytoplasmic end of transmembrane helix H6 by 5–6 A that is observed during light activation of rhodopsin. This indicates that the structures represent an inactive, non-signalling state of the receptor formed on initial agonist binding.'). Thus, the simulations effectively start from an inactive-state receptor. Although comparing the pocket residue behaviors among the wild-type E130 (3.41) and its mutants is informative, this analysis is inherently limited to the inactive state. Consequently, the authors' statement that 'direct interactions with P219 (5.50) were not sufficient to stabilise β1AR in the inactive state' is not well-supported by the data, since only the inactive conformation was briefly sampled (triplicates of 300 ns) with no exploration of transitions from an active state.

We agree with the Reviewer that our previous set of simulations were limited by the use of the inactive state. Consequently, in our new set of simulations we have utilised the active state of the turkey agonist-bound turkey β_1AR, obtained from the G_s-isoprenaline-β_1AR-Nb35 structure (PDB ID: 7JJO). We removed the G protein subunits ($G\alpha_s$, Gβ, Gγ) and Nb35, leaving the β_1AR in the active state bound to the agonist isoprenaline. The model sequence was then further modified to reflect the experimental constructs (See point 4). Therefore, the model better represents the active state and agrees with the experimental work that we conducted. We recorded new simulations (with three replicates) for each of our eight E130X$^{3.41}$ variants in addition to three replicates of E130 with AS408 in the TM3-TM4-TM5 allosteric pocket, with every simulation using the active state structure that we generated from PDB:7JJO. Although we observed no transitions to the inactive state in our 1 µs simulations, our data better represents the impacts of the E130X$^{3.41}$ variants on the receptor and TM3-TM4-TM5 allosteric pocket when bound to agonist, as well as AS408 binding into this pocket at the agonist-bound receptor. We have also modified our analysis to focus on how the frequency of different interaction types in the allosteric pocket varies between the receptor variants, which can be supported by our data. Hence, we removed the sentence "direct interactions with P219 (5.50) were not sufficient to stabilise β1AR in the inactive state" which could not be supported by our data. We use our longer (1 µs, see point 2) simulations to comment on the interaction types, rather than global changes which are evident in the experimental data.

2) It is clear that the authors aim to examine how various mutations (particularly involving the key residue E130) affect the receptor's allosteric pocket. However, regardless of the starting structure conformational state, the limited trajectory length combined with the system's size and complexity means these MD simulations are likely far from equilibrium. The authors should explicitly clarify to readers, especially those not well-versed in MD simulations, that under these conditions the results must be interpreted qualitatively rather than quantitatively.

We recognise the limitations of our previous simulations which were short in length. Consequently, we have increased the trajectory length to 1 µs (with three 1 µs replicates per set, i.e. per E130X$^{3.41}$ variant). We believe that this increased trajectory length enhances the relevance of our simulations and allows our systems to evolve closer towards equilibrium. We did not observe large structural transitions over the timescale of our simulations (which is perhaps not surprising, as the inactive/pre-active state and active state are known to be exchanging slowly) although as discussed above the Y$^{5.58}$–Y$^{7.53}$ motif was susceptible to disruption towards the inactive state. Consequently, we have predominantly used our simulations to examine the local differences in interaction networks in the TM3-TM4-TM5 allosteric

pocket, and especially to examine the stability of AS408 (which was unstable in its initial conformation (Fig. 3d) in contrast to MD simulations at the β_2AR in Liu *et al*., 2020). We agree with the Reviewer that, even at 1 µs trajectory length, our results require careful interpretation. We have endeavoured to communicate to the reader that our simulations should not be used to inform on overall structural transitions (which are instead investigated by NMR) and instead we use them to investigate local dynamics changes:
In Results section 1, paragraph 12: "These simulations implied that the structural transitions which resulted in the differing conformations observed by NMR occurred on a longer timescale than in our simulations, and hence we focused on the local dynamics of the TM3-TM4-TM5 allosteric pocket.".

In Results section 2, paragraph 3: "The addition of AS408 did not lead to inactivation of the receptor on the 1 µs simulation timescale as indicated by the canonical activation microswitches[4], similar to the E130X$^{3.41}$ variant simulations (Supplementary Fig. 14). Therefore, we focused our analyses on the AS408 binding pocket.".

3) Referring to the contacts as 'interactions' is potentially misleading: substituting a negatively charged glutamate (E130) with hydrophobic residues in a predominantly hydrophobic pocket yields contacts fundamentally different from the polar or charged interactions originally possible with the glutamate side chain. The analysis presented thus appears to reduce to measurements of spatial proximity rather than a comparison of equivalent interaction types, making it unclear what meaningful interactions are actually being evaluated. Furthermore, it is unsurprising that introducing a hydrophobic residue stabilises the hydrophobic core through additional hydrophobic contacts. Finally, the impact of these mutations on pocket flexibility (e.g. RMSD/RMSF of pocket residues) and on the conserved PIF motif during transitions between active and inactive states remains unaddressed in the current analysis.

To improve the relevance and impact of our MD simulations, we analysed our new set of simulations in a more comprehensive manner as suggested by the reviewer. Firstly, we defined the different contributions of different interactions present in the E130X$^{3.41}$ allosteric pocket. These included the differing contributions of Van der Waals interactions, hydrophobic interactions, and hydrogen bond donor interactions to the differing interaction networks between the E130X$^{3.41}$ residues and the surrounding residues. This new analysis is shown in Figure 2e, and we suggest that the additional interactions with TM5, both Van der Waals and hydrogen bond donor interactions with I$^{5.45}$ and the former for P$^{5.50}$ which are observed for the aromatic variants but predominantly absent from the E130 and hydrophobic variants, would stabilise the allosteric pocket. Given the stabilisation of the inactive state is concurrent with this observation, we argue that these increased interactions between E130X and TM5 in aromatic variants likely contribute to the stabilisation of the inactive state. Consequently, we believe that our updated and more rigorous analysis of the differing interaction types in the allosteric pocket support our interpretation of our NMR and functional data.

We have further increased our analyses to include an assessment of the GPCR microswitches, including the PIF motif, the ionic and hydrophobic locks, and the Y-Y and NPxxY motif, as suggested by the reviewer. Data for these analyses, which include the changes in these motifs over the simulation windows relative to the inactive and active states, are shown in Supplementary Fig. 14. We found that only the Y$^{5.58}$-Y$^{7.53}$ motif showed signs of collapse to the inactive state, whilst other motifs remained closer to the active state over the simulation window. The measurements used were either RMSD changes, as with the PIF motif, or inter-helical distances, which are defined in the figure legend. We further recorded how the agonist interacted with the different variants, shown in Supplementary Figure 15, again exploring the

contributions of different types of interactions. We did not observe any notable changes in agonist interactions between variants.

Finally, in addition to the above analyses investigating microswitch variability, local pocket interactions, and agonist binding, we further analysed the MD simulation replicates of AS408 bound to the β_1AR-E by both the frequency of different interaction types (Supplementary Fig. 22a), RMSD (Fig. 3d) and pair-wise RMSD matrices (Supplementary Fig. 22b) of AS408 for each replicate, and recorded representative frames for two major poses observed in the MD simulations (Fig. 3e). Pose one was observed in replicates 1 and 3, whilst pose 2 was observed only in replicate 2. As in the E130X variant simulations, we did not observe substantial changes to the microswitches using interhelical distances and RMSD of the PIF motif. Overall, we believe that our analysis of these simulation trajectories informs well on the interaction of AS408 with the allosteric pocket.

4) In addition to concerns about using an inactive-state template (PDB 2Y03), issues with the modelling arise from the fact that the template's sequence does not fully match that of the experimental constructs. According to the paragraph 'System preparation for MD simulations', only residue E130 was mutated. However, several other sequence discrepancies between the template and the constructs appear to remain unaddressed beyond the focus on residue 130. For example, the experimental constructs contain a methionine at position 90 (Supplementary Fig. 1 constructs A and B), whereas the 2Y03 template contains a valine at that position. Similarly, the constructs carry a tyrosine at position 227 (part of the conserved Y5.58-Y7.53 microswitch), whereas the 2Y03 structure contains an alanine at this position; this mutation suppresses 'the formation of the active conformation in binary agonist complexes thereby abrogating G-protein binding' (https://doi.org/10.1038/s41557-022-01009-9). At position 282, construct A contains an alanine, whereas construct B contains a cysteine for NMR labelling; however, the template contains a leucine at this site. These discrepancies between the simulation model and the actual experimental constructs raise concerns about the accuracy of the simulations and could undermine confidence in the conclusions drawn.

In our new set of simulations, we have followed suggestions from the Reviewer in resolving the discrepancies between the simulation model and the experimental constructs. Specifically, the simulation model, using 7JJO as a template, was modified to reflect the β_1AR-E construct used in ^{1}H-^{13}C NMR or TRUPATH pharmacological experiments (i.e. construct A). We matched the thermostabilising mutation background to what we have in construct A (R68S, F327A, F338A, reversion of V90 to M90). 7JJO contains the Y227 residue and was maintained in our simulations. Methionine mutations (MetΔ5 mutations), which were made to reduce signal overlap in NMR spectra, were maintained for functional assays, and therefore these mutations were introduced into the simulation model. At position 282, the template was modified to an Ala residue to match construct A. Overall, our simulation model of β_1AR-E matched construct A, which was chosen over construct B as the former was used in all functional and ^{13}C NMR experiments whilst the latter was only used in ^{19}F NMR experiments. All E130X variants were then modified from this β_1AR-E template. Consequently, our model can now better reflect the experimental data and improve the accuracy of our simulations.

5) The authors state that 'Hydrogen atoms were added, and amino acids were kept in their standard protonation state at pH of 7.0.', yet they do not clarify the specific protonation state of E130. This residue, situated within a predominantly hydrophobic environment, may not remain deprotonated under physiological conditions. Indeed, protonation state predictions from tools such as Schrödinger Maestro, along with previous computational studies, suggest that E130 could be neutral at pH 7. As such, assuming a standard negatively charged glutamate side chain without justification introduces uncertainty, since this choice could meaningfully alter the interactions within the allosteric pocket and affect the conclusions drawn from the simulations.

We agree with the Reviewer that the protonation state of E130 is important to be specified in our simulations, and likely will remain protonated in the hydrophobic environment of a lipid membrane or detergent micelle. Therefore, in our new set of simulations, both simulations of the β_1AR-E and AS408-bound β_1AR-E had E130$^{3.41}$ in its protonated state. Other residues were kept in their protonation states appropriate for pH 7.0, aside from D87 and D138 which were protonated according to previous research into the active state of turkey β_1AR (Xu, X. *et al.* 2021, reference 77). We have modified the Methods section (System preparation) to reflect this: "… and amino acids were kept in their standard protonation state at pH of 7.0 with the exception of D87$^{2.50}$, E130$^{3.41}$, and D138$^{3.49}$ being protonated in agreement with previous studies stating their importance in the active state[77]."

6) The authors do not indicate whether the MD trajectories will be made publicly available. For transparency and reproducibility, the authors should consider depositing the trajectories in a publicly accessible repository such as Zenodo.

We have now indicated in the Data availability section of our manuscript that our simulations will be made publicly available. We have uploaded all of our 27x 1 µs simulations to a Zenodo repository, with data compressed to every 10th time-point in order to maintain data integrity but reduce file size. The link to this repository is also in the Data availability statement.

Responses to "General observations":

1) In Supplementary Figure 28 (lower panels), the authors show the residue Tyr227 as part of the 2Y03 crystal structure. However, this residue is not present in the original PDB entry, which lists Ala227 in both chains A and B. This figure representation should be clarified.

We have replaced this figure with a structural representation of binding pose 1 in our MD simulations. Therefore, we have altered the figure legend and removed the issue highlighted by the reviewer. The simulation contained Y227$^{5.58}$ as this residue is present in the active structure (PDB:7JJO).

2) Typo at page 7 'Supplemenatry Fig. 16a-b'.

We have now amended this typo.

3-4) Grammatical inconsistencies in the methods section:

3) Grammatical inconsistency at page 21: 'Part of the IL3 (amino acid residues 244-271) was not present in this structure and were not modelled into the β1AR structure used in simulations. '

4) Grammatical inconsistency at page 22: 'Analysis of MD trajectories were performed with mdciao'

We have now written a new methods section for the MD simulations to reflect the setup for the new simulations, which can be found in the manuscript.

5) The manuscript frequently refers to GPCR activation states and microswitches such as the PIF motif, which play important roles in the conformational regulation of the receptor. While these terms are familiar to GPCR specialists, a brief explanatory description summarising their relevance to receptor activation would help orient non-specialist readers and improve the manuscript's accessibility for a broader audience.

We have now made the following revisions in the manuscript to address this helpful comment:

Introduction, paragraph 1 - "Communication of the ligand-specific instructions across the membrane during receptor activation is mediated by highly interconnected allosteric networks, in which conserved microswitches such as the $P^{5.50}$-$I^{3.40}$-$F^{6.44}$ or $N^{7.49}$-$P^{7.50}$-x-x-$Y^{7.53}$ motifs (superscripts refer to Ballesteros-Weinstein numbering[2]) facilitate residue side chain repacking within the hydrophobic core of class A GPCRs[3-5]."

Introduction, paragraph 3 - "Structural studies indicate GPCRs sample several distinct functional states connected via tuneable equilibria, including inactive, pre-active, and active states[14–25] (Fig. 1a). The exchange between receptor states is directly mediated by the conserved microswitches in the allosteric activation network."

Results, Section 1, paragraph 1 - "Activation of the β_1AR by orthosteric ligands arises from the repositioning of dynamic equilibria connecting receptor states of differing activity (Fig. 1a), due to the communication of ligand information through the allosteric network of conserved microswitches."

Results, Section 1, paragraph 7 - "Having established that signalling through β_1AR was significantly reduced by $E130X^{3.41}$ mutations without substantial effects on full agonist binding, we reasoned that the decreases in potency were related to changes in communication through the allosteric network of microswitches between the ligand binding site and the intracellular G protein binding surface."

Reviewer #4

We would like to thank the Reviewer for their time in reviewing our manuscript alongside one of the other Reviewers as part of the Nature Communications initiative to train Early Career Researchers. As no further comments have been made, we have not provided a point-by-point response for this Reviewer.

Manuscript NCOMMS-25-86704B

Point-by-point reply to the reviewers' comments: Second revision

We would like to thank all the Reviewers for their responses to our additions or revisions, and for the endorsement of our work. We are pleased that Reviewers #1, #3, and #4 are satisfied with the revisions, and we thank Reviewer #2 for their replies to our responses and appreciate their further suggestions. We hope to provide clarity on the additional points raised and to satisfy their queries and comments.

Reviewer #1

We would like to thank the Reviewer for reviewing our manuscript. We greatly appreciate their very favourable response and strong endorsement of our manuscript and research.

Reviewer #2

We thank the Reviewer for their helpful comments and suggestions. Here, we provide responses to the New Reviewer Comments and detail the additional revisions we have made.

1. Original reviewer comment: Authors should move beyond isoprenaline to determine whether this is a global mechanism or whether probe dependence is observed.
New Reviewer comment: The authors have addressed this comment. Consider mentioning the technical challenges posed by the narrow signal-to-noise window in the conformational BRET-based biosensor.
New Author response: We have adapted the following line in the manuscript: Results section 4, paragraph 9 "… Although the signal-to-noise window was narrower for the partial agonist, noticeable decreases in ΔBRET were still present. We also observed little change in the propranolol signal with increasing concentrations of AS408, as expected."

2. Original reviewer comment: Operational model of allosterism: authors must state and rationalise which parameters they have constrained and why.
Author Response: We rationalised our choice of parameters used in the operational model of allostery as to facilitate a direct comparison with the analysis of AS408 action at the β_2AR as described in Liu *et al*., 2020, in that we constrained parameters in the same manner in order to replicate their analysis for the turkey β_1AR. In the Supplementary Table 2 of that study, the constraints are stated: "τ_β and β-values were fixed at ~0 since AS408 is a NAM and has no agonist efficacy, respectively." Consequently, we have fixed our parameters similarly. We also fix the Hill coefficient at 1.0. We have subsequently added an additional table to the Supplementary Information file (Supplementary Table 3) which includes these details, and we have added a short section to the methods (entitled "Operational model of allostery") to further detail these analyses.
New reviewer comment: The authors have provided additional documentation, but several important issues remain:

1) Nomenclature: τβ must be corrected to τB throughout the manuscript.

2) The statement "τβ and *β*-values were fixed at ~0 since AS408 is a NAM and has no agonist efficacy, respectively" must be reworded for accuracy. Currently, it implies the β parameter drives agonist efficacy, whereas this is driven by τB – the intrinsic efficacy of the allosteric modulator. β (efficacy cooperativity) describes how the NAM affects the agonist's ability to activate the receptor, not the NAM's own efficacy.

3) Constraining β to ~0 is appropriate, as the maximal agonist response decreases to baseline with increasing concentrations of AS408. Can authors ensure the reasoning for constraining this value is included within the manuscript?

4) The mechanism of action for AS408 needs to be described more clearly throughout the manuscript. The manuscript emphasises α (affinity cooperativity) while lacking discussion of β (efficacy cooperativity). The authors should explicitly state that AS408 NAM activity is driven by both parameters.

New Author response: We thank the Reviewer for their constructive comments regarding the use of the Operational Model of Allostery and we have made revisions to the manuscript accordingly:

1) τ_β has been corrected to τ_B in the method section of the manuscript and in the Supplementary Information file (Supplementary Fig. 19 and Supplementary Table 3).

2) We have reworded the text in the "**Operational model of allostery**" methods section as follows: "… The Hill coefficient was set to 1.0 and τ_B-values were fixed at near zero with the assumption that AS408 is a NAM for agonists. This enabled values for K_A, K_B, *α* and *β* to be estimated. For calculation of $\log(\alpha\beta)$, where $\log(\alpha\beta) = \log\alpha + \log\beta$, uncertainties were propagated according to the Gaussian law of propagation of uncertainty:

$$\sigma_{\log(\alpha\beta)} = \sqrt{\sigma^2_{\log\alpha} + \sigma^2_{\log\beta}}$$

assuming independence of the parameter estimates."

We have also similarly modified the text in the figure legend of Supplementary Figure 19 and in the legend for Supplementary Table 3.

3) Based on these suggestions, and assuming that point 3 refers to the model being inappropriate rather than appropriate, we have refitted the data using a model in which the *β* parameter is no longer constrained. This reanalysis has resulted in revised estimates of K_B, *α* and *β*. The updated parameter values are now provided in Supplementary Table 3. We appreciate the reviewer's recommendation, as the revised analysis yields more plausible K_B values, allowing a more meaningful comparison with those reported by Li et al. (2020). These changes have been incorporated into the revised manuscript:
Results Section 2, paragraph 2 "We applied the operational model of allostery[58] to the TRUPATH assay data to obtain parameters for affinity cooperativity log *α* (-1.5 ± 0.4) and efficacy cooperativity log *β* (-1.7 ± 0.3) (Supplementary Fig. 19a, Supplementary Table 3). By combining these two parameters log(αβ) we can determine the overall effect of an allosteric modulator on agonist signalling. For AS408, the log(*αβ*) is -3.2 ± 0.5 thereby confirming it as a NAM at the β_1AR. The affinity of AS408 to β_1AR-E (log(K_B) = -6.1 ± 0.2) was similar to the hβ_1AR when stimulated with noradrenaline (log(K_B) = -5.6 ± 0.1)[29]."
And Results Section 4, paragraph 8 "We obtained similar parameters for log *α* (-1.4 ± 0.4), log *β* (-1.2 ± 0.5), and log (*αβ*) (-2.6 ± 0.6) when compared to the TRUPATH analysis (Supplementary Table 3), again confirming AS408's ability to reduce both the affinity and efficacy of the orthosteric ligand."

4) We have revised the manuscript to include a more detailed discussion of *β*, and in addition we have included the parameter log(*αβ*) which more accurately reflects the importance of both parameters in driving NAM activity. Please see point 3) above for the changes to the text.

3. Original reviewer comment: Can authors comment on the observed difference in Log alpha across assays? Authors must also rationalise the observed (but not reported) difference in beta values (efficacy modulation).

Author Response: We speculate that the variation in the α values is likely a result of the differing assays utilised to obtain these values. The biosensor directly reads out the conformational change in the receptor induced by the agonist co-stimulated with AS408, whereas the TRUPATH assay responds to G protein activation and is therefore an indirect measure of changes at the receptor.

We further note that the log(α) value was observed to vary at the $β_2$AR: -0.7 +/- 0.5 for [^{35}S]GTPγS binding; -0.1 +/- 0.1 for cAMP accumulation; and -0.2 +/- 0.1 for β-arrestin recruitment (Liu et al., 2020). The differences here were attributed to "better access of the modulator to the receptor in broken cell preparations (as in the [^{35}S]GTPγS binding assays) compared to the whole cell assays (cAMP and β-arrestin recruitment assays)". Consequently, variations in log(α) values may not be entirely unsurprising between different experimental systems.

We have now clarified in the methods section and in Supplementary Table 3 that the β values are held constant at near 0 in the operational model fits.

New reviewer comment: The author response does not appropriately address the question. Affinity cooperativity (α) should be a property of receptor-ligand interactions, independent of the system, unless there are equilibrium artifacts or NAM bias. The GTPγS dissociation example provided by the authors does not appear relevant to the current manuscript, as both assays in this study are whole-cell and measure downstream signalling or conformational changes rather than direct G protein binding.

New author response: Following refitting of the data using the unconstrained Operational Model of Allostery, as suggested by the reviewer, the resulting Log *α* and Log *β* values are now in close agreement across both datasets. These revised values are presented in Supplementary Table 3. We thank the reviewer for this suggestion, as the updated analysis has improved the consistency of the parameter estimates and strengthens the interpretation of the allosteric effects observed.

4. Original reviewer comment: An important missing element is the assessment of how mutations and AS408 affect the ligand-binding kinetics of orthosteric ligands.

Author response: We thank the Reviewer for raising this important question and we have now conducted Nluc ligand binding assays, as suggested by the Reviewer, to address these questions. We find that there are no significant differences in either the association or dissociation rates of both (S)-propranolol-red and isoprenaline between the E130X$^{3.41}$ variants. Therefore, we find that the ligand binding kinetics will have little impact on the interpretation of the BLI assays assessing mini-G_s binding to the receptor variants. These binding kinetics have been added to the manuscript in Supplementary Figure 3-4.

We have further recorded association or dissociation rate constants of both (S)-propranolol-red and isoprenaline to $β_1$AR-E in the presence of varying AS408 concentrations. These data support our previous observations in that AS408 has little effect on the affinity to (S)-propranolol-red but increases the K_i for isoprenaline binding. This increase is a result of dose-dependent decreases in association rates for isoprenaline binding, whereas we observed no significant changes in k_{off}

rates for isoprenaline nor any significant changes in k_{off} or k_{on} rates for (S)-propranolol-red binding. The data for these kinetic assays have been added to the manuscript in Supplementary Figure 26-27.
New reviewer comment: The authors have added valuable kinetic data, but the interpretation requires revision. The statement that there are " These data support our previous observations in that AS408 has little effect on the affinity to (S)-propranolol-red but increases the Ki for isoprenaline binding. This increase is a result of dose-dependent decreases in association rates for isoprenaline binding, whereas we observed no significant changes in koff rates for isoprenaline nor any significant changes in koff or kon rates for (S)-propranolol-red binding" is contradicted by Supplementary Figure 27G, which shows a significant change in koff at a single AS408 concentration. Similarly, Supplementary Figure 27D shows a significant change in kon for (S)-propranolol-red at a single concentration.
New author response: We respectfully disagree with the Reviewer's interpretation of these data. While we acknowledge that a significant change in the k_{off} value is observed in Supplementary Figure 26g, this effect occurs at only a single concentration of AS408 (25 µM) and is not reproduced in a concentration-dependent manner as there was no significant difference observed at 50 or 100 µM AS408, and indeed are highly similar to the 0 µM AS408 value. We therefore suggest that this observation reflects experimental variability within the dataset rather than a meaningful pharmacological effect. Consequently, we have altered the text to reflect the data and this conclusion: Results Section 3, paragraph 4 "… whereas the dissociation rate constant did not change in a concentration-dependent manner (Supplementary Fig. 26g)".
For the latter ((S)-propranolol-red k_{on} in Supplementary Fig. 27d), a significant change is observed only at a single AS408 concentration (100 µM), which is approximately 100-fold higher than the estimated affinity (K_B is ~1 µM) of AS408 following our updated analysis of pharmacological data using the Operational model (please see above comments and Supplementary Table 3). Importantly, this effect is not evident at lower concentrations, and thus argues against a meaningful effect of AS408 on the association or dissociation kinetics of (S)-propranolol-red. As we have not directly commented on this reduction in the manuscript, we have not made any further revisions to the text. In the Supplementary Information file, the figure legend for Supplementary Fig. 26 reads "No significant differences were observed in (**c**) or (**e**) (p>0.05), and significance was only observed for propranolol association at the highest concentration of AS408 (0 µM AS408 vs 100 µM AS408: p = 0.0201).", which reflects this statement.

5. **Original reviewer comment:** Figure 3a: AS408 interaction with isoprenaline appears consistent with a competitive interaction. Can the authors detail the pharmacological evidence for AS408 acting as a negative allosteric modulator at the β1AR, as currently this is unclear.
Author response: In the TRUPATH assay data shown in Figure 3a, we agree that the limited response to isoprenaline at the highest AS408 concentration (100 µM) could be consistent with a competitive interaction. However, a likely possibility is that the full curve is not present and a higher concentration of isoprenaline would be required to obtain full dose-response curves (i.e. AS408 may shift the potency for isoprenaline further to the right). A similar effect is also observed for β_1AR-F and β_1AR-Y TRUPATH data (Figure 1e) in which full concentration response curves are not observed. Additional pharmacological evidence to suggest that AS408 acts allosterically, rather than competitively, includes the absence of significant changes in the affinity of the antagonist (S)-propranolol-red in the presence of the AS408. We would anticipate that at 100 µM

AS408, we would observe competition of the antagonist and AS408. Consequently, the pharmacological data points towards an allosteric mode of action.

We also note that other structural data indicates that there is no competitive interaction between the orthosteric ligand and AS408 at βARs. The AS408-β_2AR crystal structure in Liu et al., 2020, demonstrated binding into an allosteric binding pocket, and our complementary NMR data were consistent with binding of the AS408 into this same pocket at the turkey β_1AR (Fig. 3g-h).

Furthermore, other NMR data indicates that AS408 does not bind into the orthosteric pocket. E.g. the M178 probe, which reports on the conformational dynamics and ligand identity in the orthosteric pocket, shows little to no changes in the apo spectra of β_1AR-E (M178, Fig. 4e), β_1AR-M (Supplementary Fig. 35d), β_1AR-F (Supplementary Fig. 33d), and β_1AR-Y (Supplementary Fig. 33b). Additional NMR data recorded for the propranolol-bound β_1AR-E (Supplementary Fig. 25c,f) clearly demonstrated that the unique position that M178 signal occupies when bound to the antagonist is strongly maintained upon the addition of AS408, whereas other signals clearly respond in the expected manner when the receptor populates the AS408-stabilised 'non-productive' conformation. The M178 signal position remains consistent with the propranolol-stabilised chemical shift position, which notably differs from the two positions for M178 in the apo receptor with addition of AS408. Therefore, these data clearly evidence AS408 binding and related changes in conformations and dynamics whilst also distinctly demonstrating propranolol binding to the orthosteric pocket.

Finally, the supplementary 1 μs simulations (recorded in triplicate) of β_1AR-E with the AS408 modelled in the β_2AR binding pose (Fig. 3d-f and Supplementary Fig. 22) further support an allosteric binding mode. In these simulations, AS408 is maintained in the TM3-TM4-TM5 binding pocket, although interacting with different regions of the allosteric pocket. Hence, these data also support AS408 as a negative allosteric modulator at the β_1AR.

Overall, the data indicating that AS408 does not displace the fluorescent tracer ((S)-propranolol-red) alongside the structural dataset solidifies our conclusion that AS408 acts as an allosteric modulator at the TM3-TM4-TM5 pocket and does not compete for binding in the orthosteric pocket.

New reviewer comment: While appreciating the authors have biophysical evidence supporting an allosteric mechanism of action for AS408, the following comment on pharmacological evidence should be reconsidered "Additional pharmacological evidence to suggest that AS408 acts allosterically, rather than competitively, includes the absence of significant changes in the affinity of the antagonist (S)-propranolol-red in the presence of the AS408. We would anticipate that at 100 μM AS408, we would observe competition of the antagonist and AS408. Consequently, the pharmacological data points towards an allosteric mode of action." This is because 100 μM AS408, which has an affinity of ~250 μM, would be expected to cause a <2-fold shift in propranolol for a competitive interaction, since the concentration of AS408 is less than the affinity (KD). Therefore, the lack of effect in this binding assay is not supportive of either allosterism or competitive interactions. Authors should rephrase the interpretation.

New Author response: We thank the Reviewer for continuing to raise important questions regarding the nature of AS408 activity. In their original comment, the Reviewer suggested that the observed effects might be consistent with a competitive interaction at the orthosteric binding site. However, following refitting of our data using the unconstrained Operational Model of Allostery, we now estimate the affinity of AS408 to be approximately 1 μM rather than 250 μM. Consequently, the concentrations of AS408 used in our binding studies are substantially higher than those required to produce the observed changes in propranolol affinity (approximately 100-fold), making a simple competitive mechanism unlikely. In addition, we attempted Schild

regression analyses using both the TRUPATH (Fig. 3a) and conformational BRET biosensor (Fig. 6c) datasets. In neither case could the data be adequately described by a linear Schild relationship (Fig. R1), and the resulting Schild slopes were clearly different from unity. These findings are inconsistent with a simple competitive antagonism model.

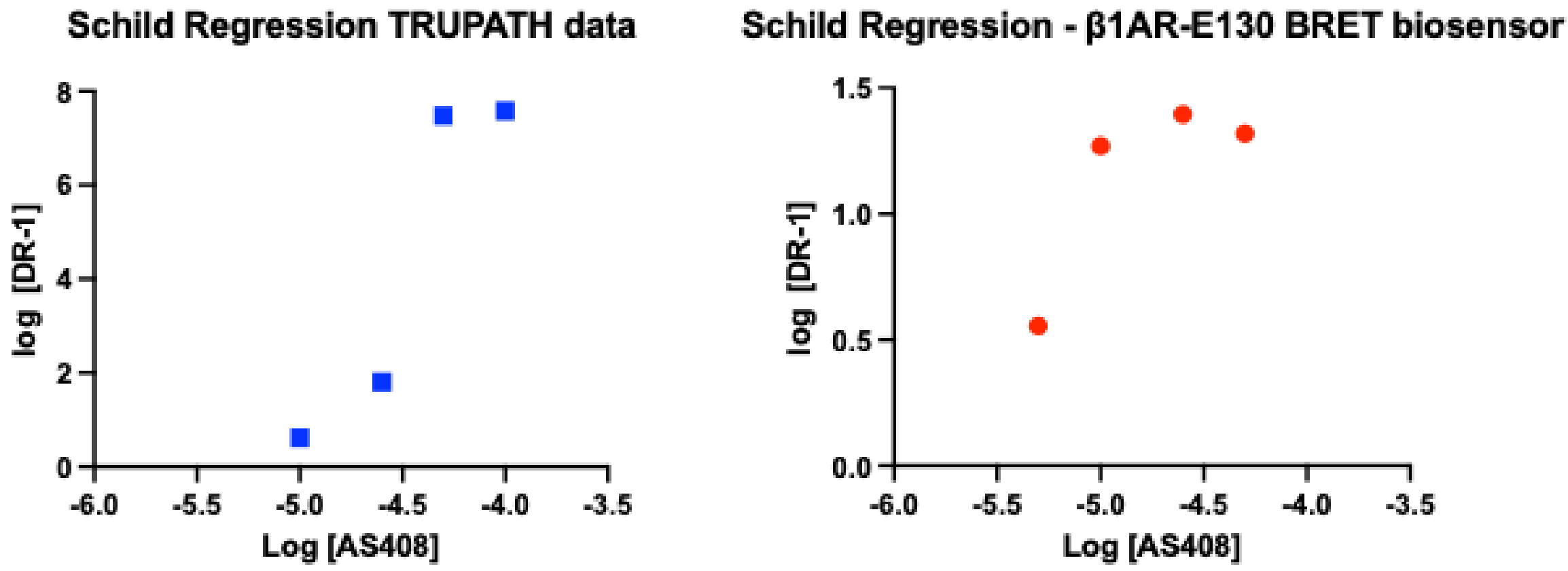

Figure R1. Schild regression for AS408 at the β_1AR from both TRUPATH (Figure 3a) and β_1AR-E BRET biosensor data (Figure 6c). In neither example is it possible to generate a linear Schild relationship. Both data sets therefore confirm that AS408 is not exhibiting competitive interactions.

Furthermore, following refitting with the Operational Model of Allostery, we obtained estimates for both the affinity cooperativity factor (α) and the efficacy cooperativity factor (β). Combining these parameters allows estimation of the overall cooperativity term, log($\alpha\beta$). For the TRUPATH dataset, log($\alpha\beta$) was −3.2, while for the β_1AR-E BRET biosensor dataset, log($\alpha\beta$) was −2.6. As both values are negative, they indicate strong negative cooperativity between AS408 and the orthosteric agonist. Taken together with the lack of a linear Schild relationship and the results of our binding and functional studies, these findings provide consistent evidence that AS408 acts as a negative allosteric modulator (NAM) rather than as a competitive orthosteric antagonist.

6. Original reviewer comment: Supplementary Figure 20: It appears that an increase in Bmax occurs with increasing concentrations of AS408. Although possible, this would be an unusual finding – can authors provide a comment and explanation for this?

Author response: We agree with the Reviewer that this observation would indeed be unusual. However, we believe that this result is likely an artifact of the experimental system. The data shown in Supplementary Fig. 20b are a ratio of fluorescence/bioluminescence, and consequently are susceptible to interference by fluorescent ligands. The AS408 molecule, which has several delocalised π systems, appears to have some low-level fluorescence which has small effects on the measurements such as absorbance or quenching effects at wavelengths relevant to this experiment. The effect on the ratio is concentration dependent and thus has the greatest effect at the 100 µM AS408. In addition, the impact is exacerbated by the ratio which can artificially inflate errors. We therefore anticipate that the result is increased variability in the fluorescence/bioluminescence ratio which can result in misleading observations such as the change in B_{max} here. In support of this observation, our new dataset that was recorded to obtain the kinetic parameters for ligand binding in the presence of AS408 demonstrates that there is now observed a decrease in the B_{max} at the highest concentration of AS408 (Supplementary Fig. 26b). Consequently, we put this observation down to ligand-dependent artifacts rather than any real effect

New Reviewer comment: Authors suggest the increased Bmax as an artifact arising from the intrinsic fluorescence of AS408 due to delocalised π systems. However, this raises a significant concern, as most AS408 effects in these BRET assays occur at concentrations >100 μM, where the fluorescence artifact would be greatest. If AS408 fluorescence affects the BRET ratio in a concentration-dependent manner, it could also quench or enhance donor/acceptor signals and skew concentration-response curves. Can the authors demonstrate, in control experiments using a different GPCR for which AS408 has minimal affinity, that AS408 does not cause artifacts in this system? If AS408 causes fluorescence artifacts, the authors should acknowledge and, if possible, correct these effects.

New Author response: We apologise for the certainty in which we proposed the cause of this reduction in B_{max} observed in our binding data. Whilst we do observe a decrease in B_{max} and this may be due to quenching of the luminescence signal as proposed in our previous response, there are several other possible explanations for this reduction. For example, B_{max} values from NanoBRET binding assays are susceptible to receptor conformational changes, as the signal is affected by the distance between the fluorescent ligand and NanoLuc tag. We therefore would like to avoid overinterpretation of this metric and hence do not discuss the observed difference in the manuscript.

We would like to highlight that we consistently see effects in these BRET assays at much lower concentrations than 100μM (a shift in potency using the biosensor is observed at 5 μM, Fig. 6c), at which there is very little interference from any compound fluorescence or quenching. Moreover, our updated Operational model analysis (see above) indicates that the K_B of AS408 is far below the 100 μM concentration of AS408. Thus, we respectfully disagree with the Reviewer that any impact of AS408 on BRET measurements will substantially skew concentration-response curves.

Whilst we are unable to test any receptors reported to not display effects with AS408 from its original publication (due to lack of technical compatibility), we have evidence using the β_1AR BRET-biosensor system. We feel this represents a good control as it maintains the identity of the biosensor system. In our previous response, we revised our manuscript to include data examining the probe dependence at AS408 using several balanced orthosteric ligands inclusive of agonists, a partial agonist, and an antagonist. In further work, we stimulated β_1AR with the β-arrestin biased ligand carvedilol, resulting in activation of the BRET-based biosensor which was unaffected by AS408 (Fig. R2), even at the highest concentration used in our previous assays (100 μM). These data were acquired more recently, for a parallel and ongoing project, and hence was not presented in the initial round of revisions. We do appreciate these data indicate a probe dependency for AS408 with respect to a biased ligand, and consequently we have amended the manuscript to indicate we do not see differences between classical, balanced ligands of the receptor: Results section 4, paragraph 9 "BRET signal changes were assessed for classical, G protein/β-arrestin balanced orthosteric ligands from three broad classifications, including agonists (adrenaline, formoterol), a partial agonist (dobutamine), and an antagonist (propranolol) with increasing concentrations of AS408.… …Consequently, we concluded that AS408 does not display probe dependence at the β_1AR when stimulated with balanced orthosteric ligands."

We prefer to not include these data showing carvedilol-stimulated response curves within our publication as differences in signalling and allosteric modulator activity between G protein/arrestin balanced agonists and β-arrestin biased agonists is of substantial interest to us, and we are actively investigating the structural mechanisms accounting for these differences. Additionally, inclusion of β-arrestin binding data, which would be necessary for further investigations of these differences, is outside the scope of this current manuscript which focuses

on allosteric networks specifically governing G protein coupling and is better suited to a follow-up investigation.

Figure R2. The carvedilol-stimulated β₁AR-E BRET-sensor shows no changes in signalling profile upon addition of AS408. a The concentration response curves for the β_1AR-E BRET-sensor when co-stimulated with

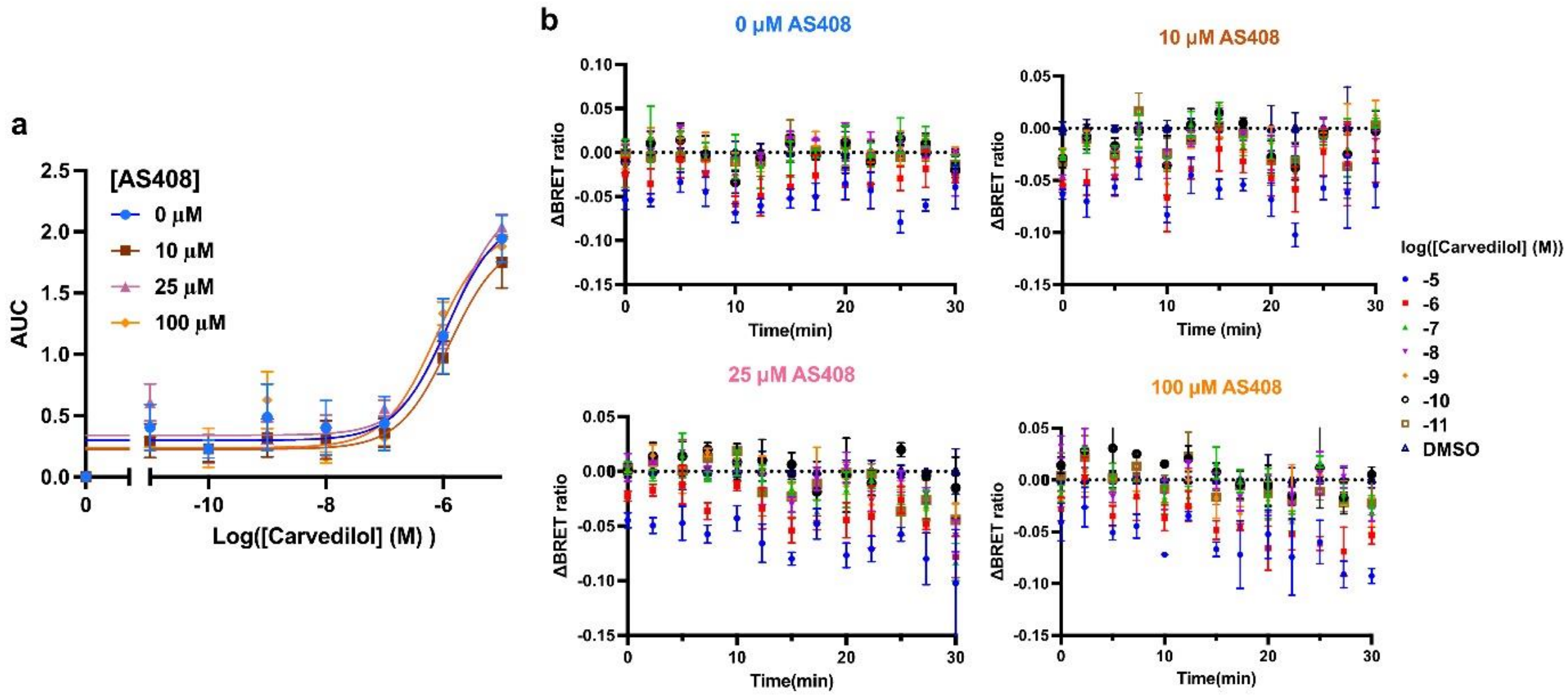

AS408 and the β-arrestin biased orthosteric ligand carvedilol. Data are the mean ± SEM of *n*=3 independent biological replicates, measured in HEK293A cells. Data are fitted using the 3 parameter logistic equations. **b** The kinetic traces for the BRET-sensor data shown in (**a**).

Reviewer #3

We thank the Reviewer for their strong endorsement of our work, and for their helpful suggestions which we agree has substantially improved our manuscript and study.

We have now made the following revisions:

1. **Reviewer:** "In the Methods, line 894, "currated β1AR-E structure" should be corrected to "curated β1AR-E structure"." **Author response:** We have corrected the error in line 894 from "currated" to "curated".
2. **Reviewer:** "For clarity, the authors may wish to specify that the reported hydrogen bonds involving I214 are backbone-mediated, since the isoleucine side chain itself cannot participate in hydrogen bonding." **Author response:** As suggested by the Reviewer, we have clarified the reported H-bonding for I241: In the figure legend for Fig. 2f, "… indicates a hydrogen bond between $Y130^{3.41}$ and the backbone carbonyl of $I214^{5.45}$."; and in the main text, "… with $I214^{5.45}$ (hydrogen bonding with the carbonyl group)…".

Reviewer #4

We would like to thank the Reviewer for their time in reviewing our manuscript alongside one of the other Reviewers as part of the Nature Communications initiative to train Early Career Researchers.

Manuscript NCOMMS-25-86704B

Point-by-point reply to the reviewers' comments: Third revision

Reviewer #2 (Remarks to the Author):

6. Original reviewer comment: Supplementary Figure 20: It appears that an increase in Bmax occurs with increasing concentrations of AS408. Although possible, this would be an unusual finding – can authors provide a comment and explanation for this?

New Reviewer comments:
Comment 6a) In relation to results section 4, paragraph 9 “BRET signal changes were assessed for classical, G protein/β-arrestin balanced orthosteric ligands from three broad classifications, including agonists (adrenaline, formoterol), a partial agonist (dobutamine), and an antagonist (propranolol) with increasing concentrations of AS408.… …Consequently, we concluded that AS408 does not display probe dependence at the β1AR when stimulated with balanced orthosteric ligands.”
Reviewers agree that AS408 decreases the response of additional agonists. However, authors should reduce their claim around at lack of probe dependence, unless they are comparing quantified allosteric modulator pharmacology. Without quantification, it is simply an observed decrease in response for all ligands tested.
Author response: We have modified our conclusions to reflect that we have examined the impact of different ligands on the conformational equilibrium stabilised by AS408, as detected by the BRET biosensor: Results Section 4, Paragraph 9: “Consequently, we concluded that AS408 acts similarly at the β_1AR-E biosensor when stimulated with different non-biased orthosteric ligands tested in this study.”

Comment 6b) In relation to "Whilst we do observe a decrease in Bmax and this may be due to quenching of the luminescence signal as proposed in our previous response, there are several other possible explanations for this reduction. For example, Bmax values from NanoBRET binding assays are susceptible to receptor conformational changes, as the signal is affected by the distance between the fluorescent ligand and NanoLuc tag. We therefore would like to avoid overinterpretation of this metric and hence do not discuss the observed difference in the manuscript. We would like to highlight that we consistently see effects in these BRET assays at much lower concentrations than 100μM (a shift in potency using the biosensor is observed at 5 μM, Fig. 6c),
at which there is very little interference from any compound fluorescence or quenching."
New Reviewer comments: We can appreciate that the authors do not want readers to over interpret the potential compound interference, however we strongly feel that the compound interference must be described and cannot be excluded from the manuscript. This is important information for any researchers who may use or synthesise AS408 for their work in the future.
Author response: We recognise the need to comment on this interference, and as such we have included the following comments in the Methods section and the legend of Supplementary Figure 26 (previously Supplementary Figure 20) and Supplementary : Methods: “In assays including AS408, minor compound interference was observed at high concentrations of AS408 (50-100 μM). This interference had only minor effects on affinity measurements.” Legend, SI 26 “Minor AS408 compound interference is observed at the maximum AS408 concentration (100 μM).” Legend, SI 30 “We note that minor AS408 compound interference is observed at the maximum AS408 concentration (50 μM), which causes a small shift in values, but this effect is negligible below this concentration.”

Comment 6c) In relation to "We do appreciate these data indicate a probe dependency for AS408

with respect to a biased ligand, and consequently we have amended the manuscript to indicate we do not see differences between classical, balanced ligands of the receptor"
New Reviewer comments: Can reviewers please rephrase the comment "classical, balanced ligands" to "classical non-biased ligands, relative to the reference ligand, XX, used within the study".
Author response: We have rephrased this sentence to the suggestion by the reviewer: Results Section 4, Paragraph 9: "BRET signal changes were assessed for classical and G protein/β-arrestin non-biased orthosteric ligands, relative to the reference ligand isoprenaline used in this study, from three broad classifications …"

Reviewer #4 (Remarks to the Author):

I co-reviewed this manuscript with one of the reviewers who provided the listed reports. This is part of the Nature Communications initiative to facilitate training in peer review and to provide appropriate recognition for Early Career Researchers who co-review manuscripts.
